# New Hydroxylactones and Chloro-Hydroxylactones Obtained by Biotransformation of Bicyclic Halolactones and Their Antibacterial Activity

**DOI:** 10.3390/molecules29122820

**Published:** 2024-06-13

**Authors:** Małgorzata Grabarczyk, Anna Duda-Madej, Fedor Romanenko, Gabriela Maciejewska, Wanda Mączka, Agata Białońska, Katarzyna Wińska

**Affiliations:** 1 Department of Food Chemistry and Biocatalysis, Wrocław University of Environmental and Life Sciences, Norwida 25, 50-375 Wrocław, Poland; 116934@student.upwr.edu.pl (F.R.); wanda.maczka@upwr.edu.pl (W.M.); katarzyna.winska@upwr.edu.pl (K.W.); 2 Department of Microbiology, Faculty of Medicine, Wroclaw Medical University, Chałubińskiego 4, 50-368 Wrocław, Poland; 3 Faculty of Chemistry, Wrocław University of Technology, Wybrzeże Wyspiańskiego 27, 50-370 Wrocław, Poland; gabriela.maciejewska@pwr.edu.pl; 4 Faculty of Chemistry, University of Wrocław, F. Joliot-Curie 14, 50-383 Wrocław, Poland; agata.bialonska@uwr.edu.pl

**Keywords:** halolactones, biotransformations, hydrolytic dehalogenation, hydroxylation, antibacterial activity, antifungal activity, multidrug resistance

## Abstract

The aim of this study was to obtain new halolactones with a gem-dimethyl group in the cyclohexane ring (at the C-3 or C-5 carbon) and a methyl group in the lactone ring and then subject them to biotransformations using filamentous fungi. Halolactones in the form of mixtures of two diasteroisomers were subjected to screening biotransformations, which showed that only compounds with a gem-dimethyl group located at the C-5 carbon were transformed. Strains from the genus *Fusarium* carried out hydrolytic dehalogenation, while strains from the genus *Absidia* carried out hydroxylation of the C-7 carbon. Both substrates and biotransformation products were then tested for antimicrobial activity against multidrug-resistant strains of both bacteria and yeast-like fungi. The highest antifungal activity against *C. dubliniensis* and *C. albicans* strains was obtained for compound **5b**, while antimicrobial activity against *S. aureus* MRSA was obtained for compound **4a**.

## 1. Introduction

Lactones are a large group of naturally occurring compounds. They are characterized by a variety of biological activities [1,2,3]. Of particular interest are lactones containing halogen atoms in their structure. Such compounds are usually obtained by chemical synthesis, although sometimes they can also be isolated from living organisms. Synthetic halolactones show antiproliferative activity [4], antimicrobial activity, cytotoxicity [5] and the ability to inhibit photosynthesis [6,7]. On the other hand, bromolactones of natural origin, isolated from the tunicates of *Pseudodistoma antinboja*, showed *antimicrobial* activity [8].

Hydroxylactones are another group of lactones characterized by biological activity, and they are often isolated from natural sources. Studies have shown that they exhibit antimicrobial [9,10,11,12,13], fungistatic [14] and cytotoxic [15] activity. Hydroxylactones have also been found to affect the pancreas [16] or liver [17].

Hydroxylactones can also be obtained by biotransformation of halolactones. Such reactions can proceed by hydrolytic dehalogenation or hydroxylation of an inactivated carbon atom leaving a halogen atom in the molecule. An example of the first reaction is the transformation of bicyclic lactones with a halogenoethylcyclohexane group catalyzed by *Absidia glauca* strain AM177 [18]. Another example is the transformation of bicyclic halolactones with one methyl group in the cyclohexane ring carried out by strains *Fusarium culmorum* AM10 and *Cunninghamella japonica* AM472 [19]. An analogous course of reactions was observed for bicyclic halolactones with two methyl groups in the cyclohexane ring when strains of the genus *Fusarium* were used for biotransformation: *F. culmorum* AM10*, F. avenaceum* AM11, *F. oxysporum* AM13, *F. scirpi* AM199*, F. solani* AM203 and *Syncephalastrum racemosum* AM105 [20].

An example of hydroxylation reactions with a halogen atom left in the molecule can be seen in biotransformations of halolactones obtained from β-citral, in which the halogen atom was located in the side chain, while the cyclohexane ring was hydroxylated [21]. Such reactions are also possible for bicyclic lactones with the halogen atom located at the secondary carbon, in the cyclohexane ring. Halo-hydroxylactones were obtained by biotransforming lactones with three methyl groups in the cyclohexane ring and a methyl group in the lactone ring. In this case, the biocatalyst was *Pleurotus ostreatus* strain PB7’96 [22]. Analogs of the above compounds containing one methyl group each in both rings (cyclohexane and lactone rings) biotransformed using *A. cylindrospora* strain AM336 yielded halo-hydroxylactones [23].

Continuing our research, we obtained further halolactones with the gem-dimethylcyclohexane system by chemical synthesis, and from them—by biotransformation—hydroxylactones. Knowing that both halo- and hydroxylactones can exhibit antimicrobial activity, we subjected the compounds we obtained both by chemical synthesis and by biotransformation to such tests.

## 2. Results

### 2.1. Obtaining of Substrates and Analysis of Their Structures

**Figure 1:**
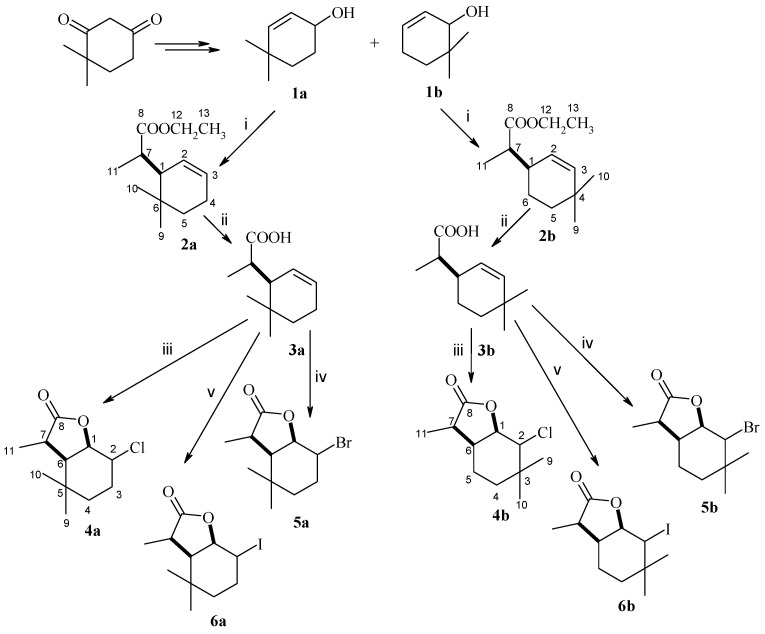
Synthesis of (±)-halolactones **4a**–**6a** and **4b**–**6b**, i: CH_3_CH_2_C(OCH_2_CH_3_)_3_, C_2_H_5_COOH, 155 °C; ii: KOH, EtOH; iii: NCS, THF; iv: NBS, THF; v: NaHCO_3_, H_2_O/(C_2_H_5_)_2_O, I_2_, KI.

In order to obtain substrates for biotransformation in organic synthesis, two known allylic alcohols **1a** and **1b** were used as substrates [24]. These alcohols were previously obtained from commercially available 4,4-dimethylcyclohes-1,3-dione. Both alcohols were subjected to Claisen rearrangement reaction with orthopropionate modification. This reaction yielded ester **2a** and ester **2b**, both as a mixture of diastereoisomers A and B in the proportions 72%:28% (compound **2a**) and 20%:80% (compound **2b**), respectively (Figures S1 and S2). The formation of each ester as two diastereoisomers was due to the presence of a methyl group in the side chain. In the next step, the esters were subjected to base hydrolysis to obtain two acids, **3a** and **3b**. These acids were also mixtures of diastereoisomers A and B in the proportions 74%:26% (compound **3a**) and 22%:78% (compound **3b**) (Figures S3 and S4). In the final step, both acids **3a** and **3b** were lactonized to obtain the corresponding halolactones, which were also mixtures of the two diastereoisomers. From acid **3a**, chlorolactone **4a** (72% A and 28% B) (Figure S5), bromolactone **5a** (76% A and 24% B) (Figure S6) and iodolactone **6a** (96% A and 4% B) (Figure S7) were obtained. Acid **3b** gave rise to chlorolactone **4b** (22% A and 78% B) (Figure S8), bromolactone **5b** (14% A and 86% B) (Figure S9) and iodolactone **6b** (23% A and 77% B) (Figure S10). A diagram of the synthesis of the above halolactones is shown in Figure 1.

**Figure 2:**
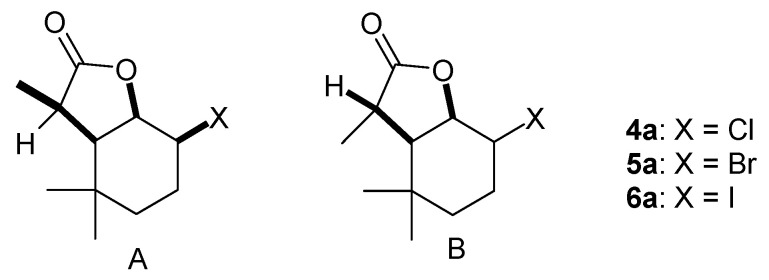
The spatial structures of diastereoisomers A and B of halolactones **4a**–**6a**.

In order to accurately analyze the structures of the obtained halolactones, they were separated into separate diastereoisomers. Analysis of the ^1^H NMR spectra of chlorolactone **4a**, bromolactone **5a** and iodolactone **6a** (Figures S31–S62) revealed that the cyclohexane ring assumes a slightly deformed chair conformation in all cases. In the case of the A isomer, the signal from the H-1 proton has a multiplet or triplet shape with a coupling constant *J* = 6.0 Hz, while the signal from the H-2 proton has a narrow multiplet shape. This indicates the axial position of proton H-1 and the equatorial position of proton H-2. In the case of isomer B, the signals coming from protons H-1 and H-2 are broad multiplets, indicating their axial orientation. A comparison of data on the structures of the lactones obtained here with the known structure of their previously obtained analog [20] made it possible to determine that the lactone ring assumes a *cis* orientation in relation to the cyclohexane ring and a *trans* orientation with respect to one of the methyl groups located at the C-5 carbon. Performing NOESY analyses, in turn, made it possible to determine the spatial orientation of the H-7 proton and the CH_3_-11 methyl group. The spectra of isomer A show couplings occurring between atoms H-1 and H-2, H-1 and H-6, and H-1 and H-7. This means that the H-7 proton is in a *cis* position relative to protons H-1, H-2 and H-6, while the CH_3_-11 group lies across the plane of the lactone ring. An analogous analysis performed for isomer B showed that disagreements occur between protons H-1 and H-6 and H-2 and H-7. This indicates that in this case, proton H-7 is in the *trans* position relative to proton H-1 and proton H-6 and cis relative to proton H-2, while the CH_3_-11 group lies in the plane of the lactone ring. The spatial structures of both stereoisomers of halolactones **4a**, **5a** and **6a** are shown in Figure 2.

**Figure 3:**
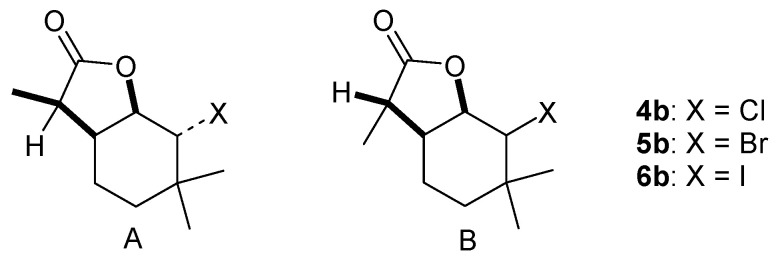
The spatial structures of diastereoisomers A and B of halolactones **4b**–**6b**.

In turn, when analyzing the ^1^H NMR spectra of chlorolactone **4b**, bromolactone **5b** and iodolactone **6b** (Figures S63–S95), it can be seen that the cyclohexane ring assumes a chair conformation. In the case of isomer A, the signal from the H-1 proton is a doublet with coupling constants of 3.6 and 3.6 Hz for compounds **4b** and **5b**, and 4.0 and 3.2 Hz for compound **6b**, while signals coming from proton H-2 are narrow multiplets. This means that both the H-1 proton and H-2 proton adopt an equatorial orientation. The signals coming from the H-1 proton of isomer B are doublets with coupling constants of 9. 6 and 6.8 Hz for compound **4b**, 10.0 and 7.6 Hz for compound **5b**, and 10.4 and 7.2 Hz for compound **6b**. The signals coming from proton H-2 are doublets (isomer B) with large coupling constants of 9.6, 10.0 and 10.4 Hz for compounds **4b**, **5b** and **6b**, respectively. This indicates their diaxial orientation. Performing NOESY analyses allowed us to confirm that two different diastereoisomers were formed in this case. In the case of isomer A, couplings are visible between protons H-1 and H-2, H-1 and H-6, and H-1 and H-7. This means that proton H-7 is in the *trans* position relative to protons H-1 and H-6, and *cis* relative to proton H-2, so the CH_3_-11 group lies across the plane of the lactone ring. In the case of isomer B, interactions are seen for protons H-1 and H-6, and H-2 and H-7. This indicates the *cis* orientation of proton H-7 relative to proton H-2, and *trans* relative to protons H-1 and H-6, and thus the positioning of the CH_3_-11 group lies in the plane of the lactone ring. The structures of halolactones **4b**, **5b** and **6b** are shown in Figure 3.

**Figure 4:**
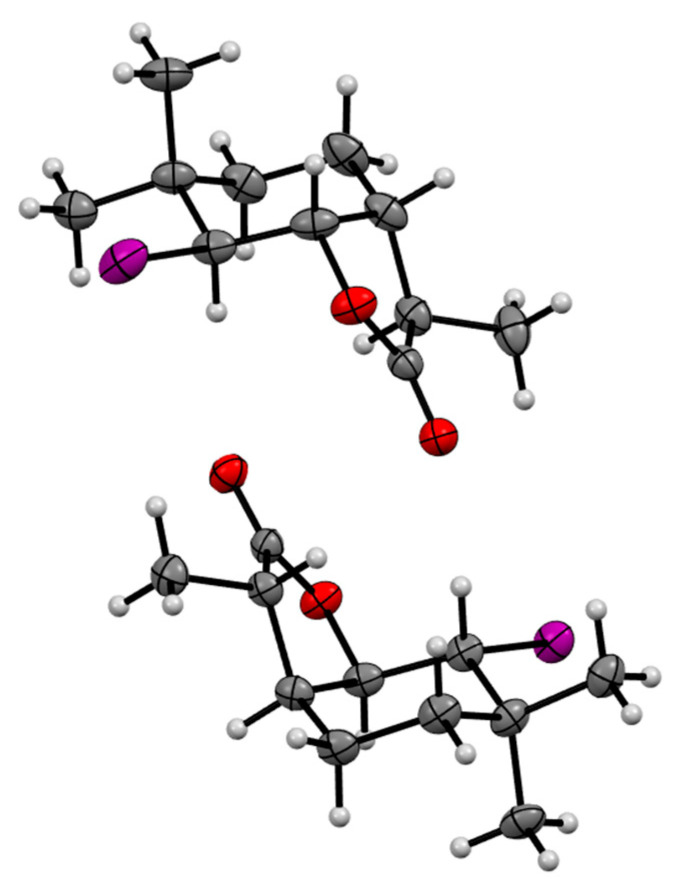
The structures of isomer B of halolactone **6b**.

For one of the isomers, specifically isomer B of iodolactone **6b**, crystallographic studies were performed. In this way, its structure was unambiguously determined, and it was found that there are two molecules in the independent part of the elementary cell (Figure 4). The absolute configurations of these two molecules were determined as 1*S*, 2*S*, 6*R*, 7*R* or 1*R*, 2*R*, 6*S*, 7*S*. Knowing the configurations of the chiral centers of one of the compounds, it was therefore possible to determine the configurations of the other halolactones obtained during the synthesis. The B isomers of chlorolactone **4b** and bromolactone **5b** have a configuration identical to that of iodolactone **6b**, described above. The A isomer of compounds **4b**–**6b** has the configuration 1*S*, 2*R*, 6*R*, 7*S* or 1*R*, 2*S*, 6*R*, 7*R*. For compounds **4a**–**6a**, isomer A has the configuration 1*S*, 2*R*, 6*R*, 7*S* or 1*R*, 2*S*, 6*S*, 7*R*, while isomer B has the configuration 1*S*, 2*S*, 6*R*, 7*R* or 1*R*, 2*R*, 6*S*, 7*S*.

### 2.2. Screening Biotransformation of Halolactones ***4a***,***b***–***6a***,***b***

The following strains of filamentous fungi from the collection of the Department of Food Chemistry and Biotechnology were used in the first stage of the study, i.e., screening biotransformation: *Fusarium culmorum* AM10, *F. avenaceum* AM12, *F. equiseti* AM16, *F. semitectum* AM20, *F. oxysporum* AM21, *F. solani* AM203, *Absidia coerulea* AM93, *A. glauca* AM177, *A. cylindrospora* AM336. The results of biotransformations of lactones **4a**–**6a** (as mixtures of diastereoisomers A and B) carried out by strains of the genus *Fusarium* and strains of the genus *Absidia* are shown in Table 1 and Table 2, respectively. Biotransformation conducted on compounds **4b**–**6b** (also as mixtures of diastereoisomers A and B) did not yield any products; in all cases, after 7 days of transformation, only substrates were present in the reaction mixture. Therefore, further studies were conducted only on compounds **4a**–**6a**.

In an analysis of the data shown in Table 1, it can be concluded that the course of biotransformation is mainly affected by the type of substituent. The presence of a substituent, which was a chlorine atom, resulted in the absence of any reaction. It was definitely different when a bromine or iodine atom was present as a substituent in the lactone molecule. Bromolactone **5a** and iodolactone **6a** were transformed by all six strains, yielding as products hydroxylactones **7a** and **8a** (or in three cases only **7a** with respect to iodolactone).

The use of strains of the genus *Absidia* (Table 2) as a biocatalyst gave different results. In this case, the course of the reaction was mainly determined by the type of biocatalyst used. The *A. coerulea* AM93 strain was not capable of transforming any of the substrates. In the case of the *A. glauca* AM177 strain, the formation of both hydroxylactones **7a** and **8a** in small amounts and the products of C-7 carbon hydroxylation, i.e., halo-hydroxylactones **9a**, **10a** and **11a**, was observed. The most efficient biotransformation was carried out by the the *A. cylindrospora* AM336 strain, which converted substrates exclusively to halo-hydroxylactones **9a**, **10a** and **11a** with good yield.

In parallel with conducting screening biotransformations, two control trials were performed. The first consisted of adding substrate (halolactones **4a**–**6b**) to a sterile medium without the addition of fungus. The second consisted of incubating the filamentous fungi used for biotransformation in a sterile medium without the addition of substrate. Both experiments were conducted for 7 days, with samples taken as in the case of the screening procedure. It was found that the reaction mixture always contained only substrate, which means that hydrolytic dehalogenation does not occur without the presence of microorganisms. It was also found that the compounds observed as biotransformation products were not secondary metabolites produced by the tested strains.

### 2.3. Preparative Biotransformation of Halolactones ***4a***–***6a***: Analysis of the Structures of the Obtained Derivatives

Conducting screening biotransformations made it possible to select strains that were used for the next step, i.e., preparative biotransformation which allows determining the structures of the resulting products. Considering the efficiency of the reaction, the best results were obtained for the *F. avenaceum* AM12 and *A. cylindrospora* AM336 strains. The first one was capable of carrying out hydrolytic dehalogenation, while the second one was able to carry out hydroxylation reactions. The results of experiments conducted using the *F. avenaceum* AM12 strain are shown in Table 3.

**Figure 5:**
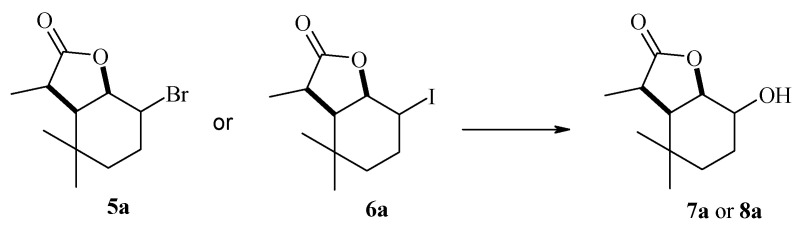
Products obtained during biotransformation of lactones **4a**–**6a** by *F. avenaceum* AM12 strain.

The use of the *F. avenaceum* AM12 strain made it possible to obtain hydroxylactone **7a** from both substrates and, in the case of iodolactone **6a**, additionally hydroxylactone **8a** (Figure 5).

**Figure 6:**
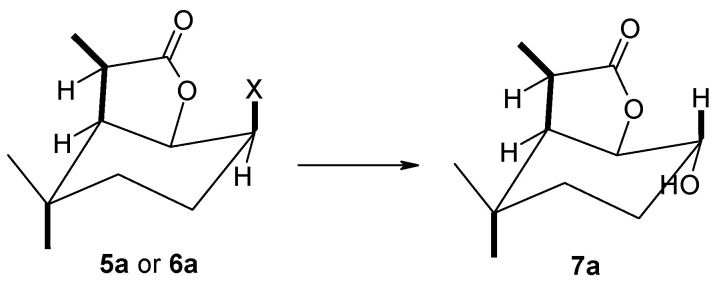
The formation of hydroxylactone **7a**.

By analyzing the ^1^H NMR spectra of both hydroxylactones (Figures S96–S105), it can be concluded that compound **7a** was formed from the A isomer of the substrate, while compound **8a** was obtained from the B isomer. The slightly deformed chair conformation of the cyclohexane ring was preserved in all cases. In the molecule of iodolactone **6a** isomer A, the iodine atom occupied the axial position, while the H-2 proton occupied an equatorial position. Thus, the hydrolytic dehalogenation reaction mechanism, which is analogous to that of SN2, resulted in the approach of the hydroxyl group from the equatorial side. Thus, the introduction of a hydroxyl group into the equatorial position of the C-2 carbon did not change the conformation of the cyclohexane ring, but only slightly deformed it. The structures of the compounds in question are shown in Figure 6.

**Figure 7:**
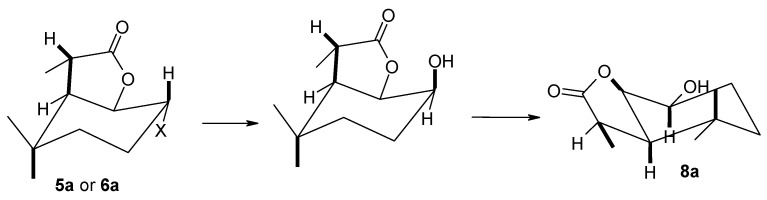
The formation of hydroxylactone **8a**.

In the case of the B isomer of iodolactone **6a**, the iodine atom was in an equatorial position, bringing the hydroxyl group into the axial position. The signal from the H-2 proton present on the NMR spectrum is a broad multiplet located in the range of 3.79–3.84 ppm, indicating its axial orientation. This means that in this case there was a conformational change in the cyclohexane ring, resulting in the hydroxyl group being in the equatorial position. This is also confirmed by changes in the position of the signals coming from the H-6 and H-1 protons. The broad multiplet coming from the H-6 proton lying in the range of 1.96–2.00 ppm has shifted towards the lower field, towards 2.44 ppm, while the narrow multiplet coming from the H-1 proton (4.78–4.81 ppm) has shifted towards the higher field, becoming a broad multiplet (4.35–4.40 ppm). The structures of the compounds in question are shown in Figure 7.

The second strain used for preparative biotransformation was the *A. cylindrospora* AM336 strain. The results of this stage of the study are shown in Table 4.

**Figure 8:**
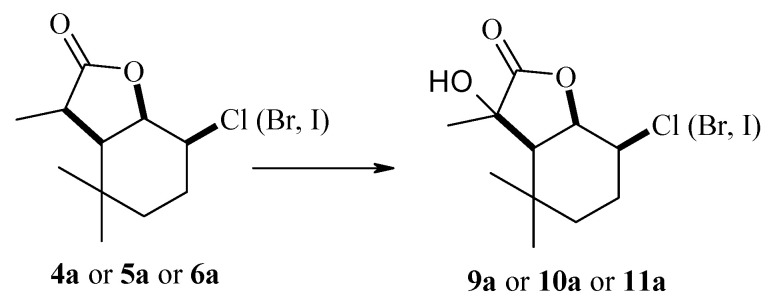
Products obtained by biotransformation of lactones **4a**–**6a** by *A. cylindrospora* AM336 strain.

The use of *A. cylindrospora* strain AM336 as a biocatalyst allowed the preparation of halo-hydroxylactones **9a**–**11a**. In all three cases, the halogen atom present in the lactone molecule remained in its position, while the hydroxyl group was introduced into the tertiary carbon C-7 (Figure 8).

**Figure 9:**
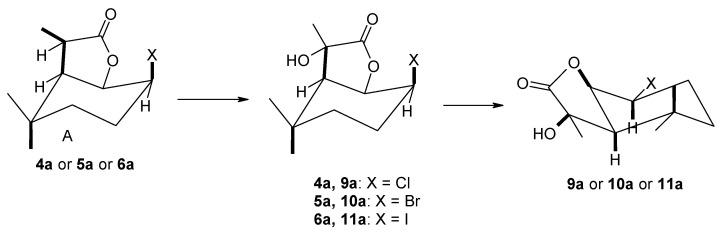
Spatial structures of halo-hydroxylactones **9a**–**11a** obtained by biotransformation of lactones **5a**–**6a** by *A. cylindrospora* AM336 strain.

By analyzing the ^1^H NMR spectra of halo-hydroxylactones **9a**–**11a** (Figures S106–S120), it can be concluded that these compounds were formed from the A isomer of the corresponding halolactones. In all cases, the cyclohexane ring assumes a chair conformation. The insertion site of the hydroxyl group is evidenced by the absence of a signal coming from the H-7 proton, as well as a shift of the signal coming from the CH_3_-11 methyl group toward the lower field. There was a definite change in the signals coming from protons H-2 and H-1, with the former shifting toward a higher field and the latter toward a lower field. In addition, the shape of the signal coming from the H-2 proton indicates a change in its orientation from equatorial to axial. These changes indicate a change in the conformation of the cyclohexane ring and the lactone ring connected to it. As a result of this change, the methyl group of CH_3_-11 was located in the plane of the lactone ring, while the newly introduced hydroxyl group was located across the plane of the lactone ring. The structures of the obtained compounds are shown in Figure 9.

All substrates used for biotransformation were racemic mixtures. Therefore, it was necessary to verify whether the products obtained by biotransformation were characterized by enantiomeric excess. Accordingly, the compounds were analyzed using a chiral column, and their optical rotation was also verified. The results are presented in Table 5.

Analyzing the data included in Table 5, it can be seen that when the hydroxyl group takes the place of the halogen atom (hydrolytic dehalogenation), the resulting products are characterized by an average enantiomeric excess (48.9–60.4%). On the other hand, when a hydroxyl group is inserted into a tertiary carbon atom (hydroxylation), the resulting products can be described as being racemic mixtures, as their enantiomeric excesses were in the range of 1.1–6.1% (Figures S121–S126).

### 2.4. Biological Tests

Both halolactones with a gem-dimethyl group in the cyclohexane ring and a methyl group in the lactone ring (tested as mixtures of diastereoisomers A and B) and their hydroxyl derivatives showed varying activity against hospital-acquired multidrug-resistant isolates. The resistance profile of strains tested during experiments is presented in Table 6.

The activity of tested lactones ranged from 64 to >512 µg/mL.

All tested compounds were inactive or very weakly active (MIC_50_ ≥ 512 µg/mL) against Gram-negative strains, i.e., *E. coli* ESBL, *P. aeruginosa* ESBL and *A. baumannii*.

Against Gram-positive strains showing MR resistance, chlorolactone **4a**, bromolactone **5a** and bromo-hydroxylactone **10a** showed the most promise, with an MIC_50_ against *S. aureus* of 128 µg/mL for the first compound and 256 µg/mL for the other two. In addition, hydroxylactone **7a** (MIC_50_ = 256 µg/mL) was active against *S. epidermidis*. Moreover, this compound also inhibited the growth of *E. faecalis* VRE, reaching MIC_50_ = 256 µg/mL, while the other tested compounds remained inactive against this bacterial strain. Furthermore, all tested lactones showed no activity and/or at very low levels against *S. haemolyticus* (MIC_50_ ≥ 512 µg/mL).

It is interesting to note that compound **4a** showed a twice lower MIC_50_ against *S. aureus* MRSA than against the reference strain *S. aureus* ATCC25923, with MIC_50_ values of 128 µg/mL and 256 µg/mL, respectively. Chlorolactone **4a** is therefore noteworthy, as it may prove to be an alternative in the fight against infections with the etiology of resistant strains.

The highest activity among the tested lactones was obtained against yeast-like fungal strains. Bromolactone **5b** showed the most interesting MIC_50_ of 64 and 128 µg/mL for *C. dubliniensis* and *C. albicans*, respectively. In addition, iodolactone **6a** also proved active against *C. albicans*, MIC_50_ = 256 µg/mL. The remaining compounds showed no (MIC_50_ > 512 µg/mL) or slightly reduced (MIC_50_ = 512 µg/mL) antifungal activity.

In an analysis of MIC_90_ values, compounds **4a** and **5a** were the most active, showing MICs of 128 µg/mL and 256 µg/mL, respectively, against *S. aureus* MRSA. In addition, compound **5b** reduced the activity of both *C. albicans* and *C. dubliniensis*, showing an MIC_50_ of 256 µg/mL.

The tested lactones did not show lethal activity against the bacterial and fungal strains used in the study. Only compound **4a** showed an MBC of 256 µg/mL against *S. aureus* MRSA.

The MIC_50_, MIC_90_ and MBC/MFC values are presented in Table 7.

Separated A and B isomers of these compounds, which showed activity against the tested strains, were taken for further antimicrobial activity studies. The A isomer of chlorolactone **4a** proved to be more active compared to the B isomer and the starting compound against the *S. aureus* MR strain. The MIC50 value was 64, >512 and 128 µg/mL for **4a**-A, **4a**-B and **4a**, respectively. Similar relationships were observed for bromolactone **5a** (starting MIC_50_ = 256 µg/mL). Isomer A showed higher activity than isomer B, MIC_50_ = 128 µg/mL vs. >512 µg/mL, respectively. Thus, for both compounds, the A isomers proved to be an order of magnitude more active than the starting compounds, 64 vs. 128 µg/mL for **4a**-A vs. **4a** and 128 vs. 256 µg/mL for **5a**-A vs. **5a**.

The same compounds also showed activity against *S. aureus* strain ATCC25923; MIC_50_ = 256 µg/mL for compounds **4a** and **5a**. After separation, the A isomers were also more active than the B isomers. For compound **4a**, the A isomer was found to be as much as twice as active as the starting compound (MIC_50_ = 64 µg/mL). In contrast, for compound **5a**, isomer A was an order of magnitude more active (MIC_50_ = 128 µg/mL) than **5a**. In both cases, the B isomers were inactive (MIC_50_ > 512 µg/mL).

The A isomer of iodolactone **6a** (only this isomer could be isolated as a pure compound) and the A and B isomers of bromolactone **5b** were taken for further studies of antifungal activity. Compound **6a** showing activity against *C. albicans* 31 strain (MIC_50_ = 256 µg/mL) proved less active for isomer A (MIC_50_ > 512 µg/mL). On the other hand, compound **5b**, which showed an MIC_50_ of 128 µg/mL against this strain, had higher activity for isomer B than for isomer A after separation into isomers (MIC_50_ = 256 µg/mL vs. MIC_50_ = 64 µg/mL, isomer A vs. isomer B, respectively). In contrast, the compound’s activity against *C. dubliniensis* 1745, which was initially 64 µg/mL, was not confirmed for any of the individual isomers. Isomer A proved to be twice as active as **5b**, while isomer B showed no activity against this strain (MIC_50_ > 512 µg/mL). 

The results obtained are shown in Table 8.

## 3. Discussion

Biotransformations carried out on halolactones make it possible to obtain hydroxyl derivatives of these compounds. These derivatives can be formed by hydrolytic dehalogenation or hydroxylation. Hydrolytic dehalogenation reactions are carried out by hydrolytic dehalogenases, enzymes responsible for replacing the halogen atom with a hydroxyl group derived from water [25,26]. The mechanism of this reaction involves the formation of a covalent bond between the substrate and the aspartate residue located in the enzyme’s active center, with the elimination of the halogen. The resulting oxoester is then hydrolyzed under the influence of water, releasing the product. In turn, hydroxylation reactions are catalyzed by cytochrome P450 monooxygenases (CYP). CYP enzymes, which come in many different variants, are capable of regioselective hydroxylation of the neutral C-H bond, especially in molecules of terpenoid compounds [27,28,29,30]. Hydroxylation reactions can also be catalyzed by unspecific peroxygenases (UPOs), enzymes often found in fungi [31].

The strains of the genus *Fusarium* used in the studies presented here were capable of hydrolytic dehalogenation to varying degrees. In the case of halolactones **4a**–**6a**, i.e., compounds with a gem-dimethyl grouping at the C-5 carbon, the formation of two hydroxylactones **7a** and **8a** (from isomers A and B, respectively) was observed. An analysis of the structures of the substrates as well as the obtained products proved that in the case of isomer A, the halogen atom occupied the axial position, so the hydroxyl group could be introduced into the equatorial position. The conformation of the cyclohexane ring did not change. In the case of isomer B, the halogen atom was in the equatorial position, and the hydroxyl group in the product also occupied the equatorial position. This means that during the biotransformation of isomer B, the conformation of the cyclohexane ring changed. Such a course of the hydrolytic dehalogenation reaction has been observed by us before [19,32].

The second type of reaction occurring during biotransformation was hydroxylation, during which strains of the genus *Absidia* converted bromolactone **5a** and iodolactone **6a** into the corresponding halo-hydroxylactones. In this case, only the formation of products was observed in which the hydroxyl group was inserted into the tertiary carbon of C-7. Moreover, it can be concluded that only the A isomer, in which the methyl group of CH_3_-11 lay in the plane of the lactone ring, underwent this reaction. It is worth noting that in this case, the conformation of the cyclohexane ring changed. Similar reactions were previously observed when the fungi *Pleurotus ostreatus* or *Absidia cylindrospora* AM336 were used as a biocatalyst [22,23].

When analyzing the course of biotransformation, attention should also be paid to the structure of the substrate, i.e., the type of substituent, which is a halogen atom, and the location of the methyl groups in the cyclohexane ring. Fungi of the genus *Fusarium* capable of conducting hydrolytic dehalogenation did not transform chlorolactone **4a**, while bromolactone **5a** and iodolactone **6a** were transformed into hydroxylactones **7a** and **8a**. This is consistent with our previous experiments, during which it was observed that chlorolactones were the least transformed compounds, while bromo- and iodolactones were more transformed [19,20,33]. These differences can be linked to the magnitudes of the ionic radii of chlorine, bromine and iodine. The enzyme’s active center adjusts better to larger ionic radii (bromine, iodine) than smaller ones (chlorine) [25]. Fungi of the genus *Absidia* were capable of transforming all three substrates **4a**–**6a** into the corresponding halo-hydroxylactones **9a**–**11a**. In the case of hydroxylation of the C-7 carbon located in the lactone ring, the type of halogen is not important.

The second element that has a significant impact on the course of biotransformation is the structure of the substrate, specifically the location of the methyl groups in the cyclohexane ring. Already at the screening stage, it was noted that only compounds with a gem-dimethyl group located at the C-5 carbon undergo biotransformation. Their analogs with two methyl groups located at the C-3 carbon did not undergo any transformation. Analogous behavior was previously observed for compounds having one methyl group at the C-5 carbon (they biotransformed) or at the C-3 carbon (they did not undergo any transformation) [33].

In an analysis of the results of the biological tests, it can be seen that the structure of the compound affects its antimicrobial activity. The presence of a gem-dimethyl moiety at the C-5 carbon promoted the inhibition of the growth of Gram-positive bacteria, while at the C-3 carbon—yeast-like fungi. The presence of a chlorine or bromine atom in the lactone molecule (compounds **4a**, **5a**) resulted in inhibition of the growth of *S. aureus* bacteria, both the reference strain and the strain showing MR-type resistance. An analogous effect was observed for compound **10a**, in which a bromine atom and a hydroxyl group were present. The introduction of a hydroxyl group in place of a halogen atom (compound **7a**) resulted in growth inhibition of the bacteria *E. faecalis* and *S. epidermidis*. In contrast, bromolactone **5b** showed the highest activity against the yeast-like fungi *C. albicans* and *C. dubliniensis*.

Further tests carried out on the separated A and B isomers showed that the A isomers of the tested compounds have higher activity against Gram-positive bacterial strains (*Staphylococcus* spp.). However, the starting compounds are more active against strains of the genus *Candida*. Separation into individual fractions did not affect the antifungal activity.

## 4. Materials and Methods

### 4.1. General Methods

The progress of the reaction, the course of biotransformation and the purity of the obtained products were determined by using thin-layer chromatography (TLC) and gas chromatography (GC). TLC plates (TLC Silica gel 60 F254, Merck, Darmstadt, Germany) and eluent in the form of a mixture of hexane/acetone in different volume ratios were used for thin-layer chromatography. The presence of the tested compounds was checked using a cerium inducer (1% Ce(SO_4_)_2_, 2% H_3_[P(Mo_3_O_10_)_4_] in 10% H_2_SO_4_). The reaction mixtures were purified using column chromatography with silica gel (Kieselgel 60, Merck, Darmstadt, Germany, mesh 230–400) and eluent in the form of a mixture of hexane/acetone at different volume ratios. GC analyses were carried out on an Agilent Technologies 6890N instrument gas chromatograph (Varian, Agilent Technologies, Santa Clara, CA, USA) using an SGE BP5 GC column (cross-linked methyl silicone gum 30 m × 0.25 mm × 0.25 µm). The injector and detector (FID) temperatures were 150 °C and 300 °C, respectively. The temperature program used to monitor the course of the synthesis (for compounds **2a**, **3a**, **2b**, **3b**) was as follows: initial column temperature 100 °C (hold for 1 min), ramp 100–220 °C at 25 °C/min, ramp 220–300 °C at 40 °C/min. For halolactones **4a**–**6a**, the program was as follows: injector 200 °C, detector (FID) 300 °C, 120 °C hold 5 min, ramp 5 °C/min to 200 °C, ramp 40 °C/min to 300 °C. On the other hand, for halolactones **4b**–**6b**, the following program was used: injector 250 °C, detector (FID) 300 °C, 160 °C hold 1 min, ramp 15 °C/min to 230 °C, ramp 30 °C/min to 300 °C. When analyzing the biotransformation progress, a different program variant was used: injector 150 °C and detector (FID) 300 °C, initial column temperature 130 °C (hold for 1 min), ramp 130–260 °C at 25 °C/min, ramp 260–300 °C at 40 °C/min. A CP-cyclodextrin-B-325 chiral column (30 m × 0.25 mm × 0.25 µm) (Supelco, Bellefonte, PA, USA) was used to determine the enantiomeric excesses of biotransformation products. For all compounds, the temperature of the injector and detector (FID) was 200 °C. The temperature program for hydroxylactone **7a** was as follows: 140 °C (hold 10 min), 140–190 °C (rate 1 °C/min), and 190–200 °C (rate 10 °C/min). For hydroxylactone **8a** it was: 140 °C (hold 20 min), 140–180 °C (rate 1 °C/min), and 180–200 °C (rate 20 °C/min). In turn, for halo-hydroxylactones, the following conditions were used: 140 °C (hold 1 min), 140–200 °C (rate 1 °C/min). All compounds were subjected to HR-MS analysis using a Waters LCT Premier XE instrument (ESI ionization, Waters, Milford, MA, USA). A JEOL DeltaTM 400 MHz spectrometer (JEOL USA, Inc., Peabody, MA, USA) and a Bruker AvanceTM 600 MHz spectrometer (Bruker, Rheinstetten, Germany) were used to obtain NMR spectra, using CDCl_3_ as a solvent. The optical rotation of biotransformation products was determined using a Jasco P-2000 polarimeter (Jasco, Easton, PA, USA); measurements were performed for chloroform solutions, at concentrations expressed in g/100 mL.

### 4.2. Organic Synthesis

*(1R, 7S or 1S, 7R)-Ethyl ester of (4,4,7-trimethylcyclohex-2-en-1-yl)acetic acid (isomer A) and (1R, 7R or 1S, 7S)-Ethyl Ester of (4,4,7-trimethylcyclohex-2-en-1-yl)acetic acid (isomer B)* (**2a**), The known allylic alcohol **1a** [24] was subjected to Claisen rearrangement with orthopropionate modification. Briefly, a mixture of alcohol (3.5 g), triethyl orthopropionate (20 cm^3^) and a catalytic amount of propionic acid was heated at 138 °C for 30 h. The progress of the reaction was monitored by TLC and GC. The obtained ester was purified by column chromatography using a 19:1 mixture of hexane and acetone as eluent. Ester **2a** (3.9 g, 68%) was obtained as a pair of diastereoisomers A and B in the ratio 72%:28%. The spectral data of the obtained compound **2a** are as follows: ^1^H NMR (600 MHz, CDCl_3_): 0.86 (s, 3H, CH_3_-9B), 0.87 (s, 3H, CH_3_-9A), 0.94 (s, 3H, CH_3_-10B), 0.96 (s, 3H, CH_3_-10A), 1.10–1.14 (M, 2H, CH_2_-5B), 1.09 (d, *J* = 7.2 Hz, 3H, CH_3_-11B), 1.20–1.26 (m, 9H, CH_3_-13A, CH_3_-13B, CH_3_-11A), 1.33–1.42 (m, 2H, CH_2_-4B), 1.58 (m, 2H, CH_2_-5A), 1.96–2.00 (m, 2H, CH_2_-4A), 2.27–2.37 (m, 2H, H-1A and H-1B), 2.58 (qd, *J* = 6.8 and 4.8 Hz, 1H, H-7B), 2.71 (qd, *J* = 7.2 and 3.6 Hz, 1H, H-7A), 4.06 (q, *J* = 7.2 Hz,2H, CH_2_-12A), 4.11 (q, *J* = 6.8 Hz,2H, CH_2_-12B), 5.49–5.54 (m, 2H, H-3A and H-3B), 5.69–5.74 (m, 2H, H-2A and H-3B), ^13^C NMR (151 MHz, CDCl_3_): 9.26 (C-11B), 14.19 (C-11A), 14.61 (C-13B), 18.71 (C-13A), 22.78 (C-4A), 23.07 (C-9A), 24.74 (C-9B), 25.56 (C-4B), 28.79 (C-10B), 29.04 (C-10A), 29.18 (C-6B), 29.62 (C-6A), 34.82 (C-5B), 36.03 (C-5A), 39.86 (C-7B), 39.95 (C-7A), 45.90 (C-1B), 49.85 (C-1A), 60.02 (C-12A), 60.36 (C-12B), 126.34 (C-2A), 126.77 (C-2B), 139.02127.03 (C-3A), 127.55 (C-3B), 176.20 (C-8A), 177.55 (C-8B), ESIHRMS: calculated for C_13_H_22_O_2_, 211.1698 (M + H)^+^, found 211.1700.

*(1R, 7S or 1S, 7R)-(4,4,7-trimethylcyclohex-2-en-1-yl)acetic acid (isomer A) and (1R, 7R or 1S, 7S)-(4,4,7-trimethylcyclohex-2-en-1-yl)acetic acid (isomer B)* (**3a**), Ester **2a** (3.9 g) was given alkaline hydrolysis according to a known procedure [32]. Briefly, the ester and 5% potassium hydroxide solution in ethanol were heated for 6 h under reflux conditions. After the reaction was completed, the ethanol was evaporated, the residue was dissolved in water, acidified with 1 mol hydrochloric acid, and then extracted with diethyl ether. As a result, 1.5 g (44%) of acid **3a** was obtained, as a pair of isomers A and B in the ratio 74%:26%. The spectral data of compound **3a** are as follows: ^1^H NMR (400 MHz, CDCl_3_): 0.89 (s, 3H, CH_3_-9A), 0.92 (s, 3H, CH_3_-9B), 0.96 (s, 3H, CH_3_-10A), 0.98 (s, 3H, CH_3_-10B), 1.11 (d, *J =* 7.2 Hz, 3H, CH_3_-11A), 1.27 (d, *J =* 7.6 Hz, 3H, CH_3_-11B), 1.29–1.34 (m, 1H, CH_2_-5B), 1.98–2.00 (m, 2H, CH_2_-4A), 2.04–2.06 (m, 1H, H-1B), 2.38–2.40 (m, 1H, H-1A), 2.64–2.70 (qd, *J =* 7.2 and 4.4 Hz, 1H, H-7a), 2.73–2.76 (qd, *J =* 7.2 and 4.0 Hz, 1H, H-7B), 5.49–5.50 (m, 1H, H-3B), 5.51–5.53 (m, 1H, H-3A), 5.69–5.70 (m, 1H, H-2B), 5.73–5.77 (m, 1H, H-2A), ^13^C NMR (151 MHz, CDCl_3_): 14.10 (C-11A), 17.82 (C-11B), 22.75 (C-9A), 23.32 (C-4A), 24.70 (C-9B), 29.12 (C-10A), 29.60 (C-10B), 32.31 (C-6A), 32.60 (C-6B), 34.93 (C-5A), 35.79 (C-5B), 39.45 (C-7A), 40.11 (C-7B), 45.80 (C-1B), 45.80 (C-4B), 49.50 (C-1A), 126.30 (C-2A), 126.72 (C-2B), 127.03 (C-3B), 127.90 (C-3B), 182.36 (C-8B), 183.870 (C-8A), ESIHRMS: calculated for C_11_H_18_O_2_, m/z 183.1385 (M + H)^+^, found 183.1378.

*(1S, 2R, 6R, 7S or 1R, 2S, 6S, 7R)-2-Chloro-3,3,7-trimethyl-9-oxabicyclo[4.3.0]nonan-8-one (isomer A) and (1S, 2S, 6R, 7R or 1R, 2R, 6S, 7S)-2-Chloro-3,3,7-trimethyl-9-oxabicyclo[4.3.0]nonan-8-one (isomer B)* (**4a**), Chlorolactone was prepared according to a known procedure [32]. Briefly, acid **3a** (0.5 g) and N-chlorosuccinimide (0.9 g) dissolved in THF (30 mL) were stirred for 24 h at room temperature. The reaction mixture was diluted with water, and the product was extracted with diethyl ether. After purification of the reaction product by column chromatography, using a mixture of hexane/acetone 3:1 as eluent, 0.35 g (59%) of chlorolactone **4a** was obtained as a pair of isomers A and B, occurring in the ratio 72%:28%. The spectral data of compound **4a** are as follows: Isomer A: ^1^H NMR (600 MHz, CDCl_3_): 1.09 (s, 3H, CH_3_-9), 1.11 (s, 3H, CH_3_-10), 1.41–1.44 (m, 1H, one of CH_2_-4), 1.46 (d, *J =* 7.2 Hz, 3H, CH_3_-11), 1.59–1.62 (m, 1H, one of CH_2_-4), 1.88-1.94 (m, 1H, one of CH_2_-3), 2.15–2.20 (m, 1H, CH_2_-3), 2.47 (dd, *J =* 7.2 and 6.0 Hz, 1H, H-6), 2.76 (q, *J =* 7.8 Hz, 1H, H-7), 4.23–4.28 (m, H-2), 4.49–4.52 (m, 1H, H-1), ^13^C NMR (151 MHz, CDCl_3_): 14.07 (C-11), 24.67 (C-9), 27.83 (C-3), 30.80 (C-5), 32.38 (C-10), 34.22 (C-4), 39.64 (C-7), 47.58 (C-6), 58.11 (C-2), 82.26 (C-1), 178.93 (C-8). Isomer B: ^1^H NMR (400 MHz, CDCl_3_): 1.04 (s, 3H, CH_3_-10), 1.08 (s, 3H, CH_3_-9), 1.33 (d, *J =* 6.8 Hz, 3H, CH_3_-11), 1.42–1.53 (m, 1H, CH_2_-4), 1.84–1.92 (m, 1H, one of CH_2_-3), 2.01–2.04 (m, 1H, H-6), 2.06–2.11 (m, 1H, one of CH_2_-3), 2.36–2.44 (m, 1H, H-7), 3.62–3.69 (m, 1H, H-2), 4.97 (dd, *J =* 9.2 and 7.2 Hz, 1H, H-1), ^13^C NMR (100 MHz, CDCl_3_): 17.29 (C-11), 28.15 (C-9), 29.481 (C-10), 29.85 (C-3), 32.70(C-5), 34.29 (C-4), 35.90 (C-7), 54.62 (C-6), 60.75 (C-2), 81.62 (C-1), 178.85 (C-8). ESIHRMS: calculated for C_11_H_17_ClO_2_, m/z 217.0995 and 219.0969 (M + H)^+^, found 217.0999 and 219.0970, calculated for C_11_H_17_ClO_2_Na, m/z 239.0815 and 241.0788 (M + H)^+^, found 239.0823 and 241.0790.

*(1S, 2R, 6R, 7S or 1R, 2S, 6S, 7R)-2-Bromo-3,3,7-trimethyl-9-oxabicyclo[4.3.0]nonan-8-one (isomer A) and (1S, 2S, 6R, 7R or 1R, 2R, 6S, 7S)-2-Bromo-3,3,7-trimethyl-9-oxabicyclo[4.3.0]nonan-8-one (isomer B)* (**5a**), Bromolactone was obtained according to a known procedure [32]. Briefly, acid **3a** (0.5 g) and N-bromosuccinimide (0.7 g) dissolved in THF (30 mL) were stirred for 24 h at room temperature. Water was added to the reaction mixture and the product was extracted with diethyl ether. After purification of the reaction product by column chromatography, using a mixture of hexane/acetone 3:1 as eluent, 0.54g (75%) of bromolactone **5a** was obtained as a pair of isomers A and B, occurring in the ratio 76%:24%. The spectral data of compound **5a** are as follows: Isomer A: ^1^H NMR (600 MHz, CDCl_3_): 1.09 (s, 3H, CH_3_-9), 1.12 (s, 3H, CH_3_-10), 1.43–1.46 (m, 1H, one of CH_2_-4), 1.47 (d, *J* = 7.2 Hz, 3H, CH_3_-11), 1.62 (m, 1H, one of CH_2_-4), 2.00–2.06 (m, 3H, one of CH_2_-3), 2.21–2.27 (m 1H, one of CH_2_-3), 2.49 (dd, *J* = 7.2 and 6.6 Hz, 1H, H-6), 2.77 (q, *J* = 7.2 Hz, 1H, H-7), 4.36 (m, H-2), 4.61–4.65 (t, *J* = 6.0 Hz, 1H, H-1), ^13^C NMR (151 MHz, CDCl3): 17.18 (C-11), 28.08 (C-3), 29.47 (C-5), 30.81 (C-9), 32.45 (C-10), 35.69 (C-4), 39.82 (C-7), 49.70 (C-6), 50.12 (C-2), 82.47 (C-1), 178.92 (C-8). Isomer B: ^1^H NMR (600 MHz, CDCl_3_): 1.06 (s, 3H, CH_3_-10), 1.12 (s, 3H, CH_3_-9), 1.37 (d, *J* = 6.6 Hz, 3H, CH_3_-11), 1.42–1.44 (m, 1H, one of CH_2_-4), 1.49–1.54 (m, 1H, one of CH_2_-4), 2.02–2.10 (m, 2H, one of CH_2_-3, H-6), 2.16–2.19 (m, 1H, one of CH_2_-3), 2.45–2.51 (m, 1H, H-7), 3.74–3.79 (m, 1H, H-2), 4.66 (dd, *J* = 9.6 and 7.2 Hz, 1H, H-1), ^13^C NMR (151 MHz, CDCl3): 17.14 (C-11), 28.08 (C-9), 29.047 (C-10), 30.72 (C-3), 32.64 (C-5), 35.22 (C-4), 35.62 (C-7), 51.72 (C-2), 54.84 (C-6), 81.70 (C-1), 178.65 (C-8). ESIHRMS: calculated for C_11_H_17_BrO_2_, *m*/*z* 261.0490 and 263.0473 (M + H)^+^, found 261.0475 and 263.0453, calculated for C_11_H_17_BrO_2_Na, *m*/*z* 283.0310 and 285.0290 (M + H)^+^, found 283.0304 and 285.0284.

*(1S, 2R, 6R, 7S or 1R, 2S, 6S, 7R)-2-Iodo-3,3,7-trimethyl-9-oxabicyclo[4.3.0]nonan-8-one (isomer A) and (1S, 2S, 6R, 7R or 1R, 2R, 6S, 7S)-2-Iodo-3,3,7-trimethyl-9-oxabicyclo[4.3.0]nonan-8-one (isomer B)* (**6a**), Iodolactone was prepared according to a known procedure [32]. Briefly, acid **3a** (0.5 g) was dissolved in 25 mL diethyl ether and stirred for 1 h with 25 mL of 0.5 mol sodium bicarbonate solution. Then, 1 g of iodine and 2 g of potassium iodide in 20 mL of water were added to the solution, and the whole mixture was stirred for 24 h. After the reaction was completed, the product was extracted with diethyl ether. After purification of the compound by column chromatography, using a mixture of hexane/acetone 3:1 as eluent, 0.48g (57%) of iodolactone **6a** was obtained as a pair of isomers A and B, occurring in the ratio 96%:4%. The spectral data of compound **6a** are as follows: Isomer A: ^1^H NMR (400 MHz, CDCl_3_): 1.04 (s, 3H, CH_3_-9), 1.09 (s, 3H, CH_3_-10), 1.41–1.48 (m, 2H CH_2_-4), 1.45 (d, *J* = 7.2 Hz, 3H, CH_3_-11), 2.03–2.17 (m, 2H, CH_2_-4), 2.09–2.14 (m, 2H, CH_2_-3), 2.43 (dd, *J* = 7.2 and 6.0 Hz, 1H, H-6), 2.71 (q, *J* = 7.8 Hz, 1H, H-7), 4.42 (m, H-2), 4.70 (m, 1H, H-1), ^13^C NMR (100 MHz, CDCl_3_): 14.42 (C-11), 25.18 (C-9), 28.58 (C-2), 30.87(C-3), 32.66 (C-10), 37.34 (C-4), 40.09 (C-7), 47.73 (C-6), 54.77 (C-5), 84.06 (C-1), 179.06 (C-8). Isomer B: ^1^H NMR (400 MHz, CDCl_3_): 1.04 (s, 3H, CH_3_-9), 1.09 (s, 3H, CH_3_-10), 1.41–1.49 (m, 2H, CH_2_-4), 1.45 (d, *J* = 7.6 Hz, 3H, CH_3_-11), 1.92–1.97 (m, 2H, one of CH_2_-3), 2.11–2.15 (m, 1H, CH_2_-6), 2.20–2.22 (m, 1H, one of CH_2_-3), 2.48–2.55 (qm, 1H, H-7), 3.74–3.81 (m, H-2), 4.69–4.72 (m, H-1), ^13^C NMR (100 MHz, CDCl_3_): 17.10 (C-11), 25.18 (C-9), 28.12 (C-2), 29.74 (C-10), 30.87 (C-5), 33.02 (C-3), 37.34 (C-4), 40.01 (C-7), 47.69 (C-6), 82.91 (C-1), 179.06 (C-8). ESIHRMS: calculated for C_11_H_17_IO_2_, *m*/*z* 309.0352 (M + H)^+^, found 309.0352, calculated for C_11_H_17_IO_2_Na, *m*/*z* 331.0171 (M + H)^+^, found 331.0161.

*(1R, 7S or 1R, 7R)-Ethyl ester of (4,4,7-trimethylcyclohex-2-en-1-yl)acetic acid (isomer A) and (1R, 7R or 1S, 7S)-Ethyl ester of (4,4,7-trimethylcyclohex-2-en-1-yl)acetic acid (isomer B)* (**2b**), The known allylic alcohol **1b** [24] (1.5 g) was subjected to Claisen rearrangement with orthopropionate modification analogously as described for ester **1a**. Ester **2b** (1.6 g) was obtained as a mixture of two diastereoisomers A and B in the ratio 20%:80% in 65% yield. The spectral data of compound **2b** are as follows: ^1^H NMR (400 MHz, CDCl_3_): 0.93 (s, 3H, CH_3_-9), 0.94 (s, 3H, CH_3_-10), 1.09 (d, *J* = 6.8 Hz, 3H, CH_3_-11A), 1.11 (d, *J* = 6.8 Hz, 3H, CH_3_-11B), 1.24 (t, *J* = 7.6 Hz, 3H, CH_3_-12), 1.36–1.42 (m, 2H, one of CH_2_-5, one of CH_2_-6), 1.47–1.52 (m, 1H, one of CH_2_-6), 1.54–1.53 (m, 1H, one of CH_2_-5), 2.29–2.37 (m, 2H, H-1 and H-7), 4.12 (q, 2H, CH_2_-12), 5.28–5.31 (dm, *J* = 10.0 Hz, 1H, H-3), 5.43–5.45 (dm, *J* = 8.4 Hz, 1H, H-2),^13^C NMR (100 MHz, CDCl_3_): 13.63 (C-11A), 13.92 (C-11B), 14.39 (C-13A and C-13B), 22.39 (C-5A), 24.07 (C-5B), 28.77 (C-9A), 29.00 (C-9B), 30.52 (C-10B), 30.71 (C-10A), 31.66 (C-4A), 31.74 (C-4B), 36.27 (C-6B), 36.39 (C-6A), 38.48 (C-1A), 38.51 (C-1B), 44.06 (C-7B), 44.21 (C-7A), 60.22 (C-12A and C-12B), 125.60 (C-3A), 126.80 (C-3B), 139.02 (C-2A), 139.31 (C-2B), 176.07 (C-8B), 176.16 (C-8A), ESIHRMS: calculated for C_13_H_22_O_2_, *m*/*z* 211.1698 (M + H)^+^, found 211.1700.

*(1R, 7S or 1R, 7R)-(4,4,7-trimethylcyclohex-2-en-1-yl)acetic acid (isomer A) and (1R, 7R or 1S, 7S)-(4,4,7-trimethylcyclohex-2-en-1-yl)acetic acid (isomer B)* (**3b**), Ester **2b** (1.6g) was given an alkaline hydrolysis, analogously as described for acid **2a**. Acid **3b** (0.7g) was obtained as a mixture of two diastereoisomers A and B in the ratio 22%:78% in 50% yield. The spectral data of compound **3b** are as follows: ^1^H NMR (400 MHz, CDCl_3_): 0.90 (s, 3H, CH_3_-10B), 0.94 (s, 3H, CH_3_-9A), 0.96 (s, 3H, CH_3_-10A,), 0.96(s, 3H, CH_3_-9B), 1.12 (d, *J* = 6.8 Hz, 3H, CH_3_-11A), 1.16 (d, *J* = 6.8 Hz, 3H, CH_3_-11B), 1.38–1.43 (m, 4H, one of CH_2_-5A, one of CH_2_-6A, one of CH_2_-5B, one of CH_2_-6B), 1.49–1.53 (m, 2H, one of CH_2_-6A, one of CH_2_-6B), 1.63–1.68 (m, 2H, one of CH_2_-5A, one of CH_2_-5B), 2.37–2.44 (m, 4H, H-1A, H-1B, H-7A and H-7B), 5.33–5.35 (dm, *J* = 10.4 Hz, 2H, H-3A, H-3B), 5.46–5.49 (dm, *J* = 10.0 Hz, 2H, H-2A, H-2B), ^13^C NMR (100 MHz, CDCl_3_): 13.19 (C-11A), 13.62 (C-11B), 22.05 (C-5A), 24.19 (C-5B), 28.77 (C-9A), 29.00 (C-9B), 30.49 (C-10B), 30.69 (C-10A), 31.65 (C-4A), 31.75 (C-4B), 43.90 (C-6B), 43.00 (C-6A), 38.19 (C-1A), 38.19 (C-1B), 43.90 (C-7B), 44.00 (C-7A), 125.12 (C-3A), 126.63 (C-3B), 139.36 (C-2A), 139.71 (C-2B), 182.31 (C-8B), 182.39 (C-8A), ESIHRMS: calculated for C_11_H_18_O_2_, *m*/*z* 183.1385 (M + H)^+^, found 183.1376. 

*(1S, 2R, 6R, 7S or 1R, 2S, 6R, 7R)-2-Chloro-3,3,7-trimethyl-9-oxabicyclo[4.3.0]nonan-8-one (isomer A) and (1S, 2S, 6R, 7R or 1R, 2R, 6S, 7S)-2-Chloro-3,3,7-trimethyl-9-oxabicyclo[4.3.0]nonan-8-one (isomer B)* (**4b**), Acid **3b** (0.2g) was subjected to chlorolactonization in the same manner as given for compound **3a**. The product **4b** was obtained as a mixture of two diastereoisomers A and B in the ratio 22%:78% with a yield of 0.19 g (80%) with the following spectral data: Isomer A: ^1^H NMR (400 MHz, CDCl_3_): 1.07 (s, 3H, CH_3_-9), 1.07 (s, 3H, CH_3_-10), 1.18 (d, *J* = 7.2 Hz, 3H, CH_3_-11), 1.24–1.30 (m, 2H, CH_2_-4), 1.56–1.70 (m, 2H, CH_2_-5), 2.60–2.67 (m, 1H, H-6), 2.75 (q, *J* = 7.2 Hz, 1H, H-7), 4.10 (m, 1H, H-2), 4.57 (dd, *J* = 3.6 and 3.6 Hz, 1H, H-1), ^13^C NMR (100 MHz, CDCl3): 9.56 (C-11), 18.95 (C-4), 25.64 (C-9), 30.17 (C-10), 30.57 (C-5), 34.10 (C-3), 35.49 (C-6), 40.97 (C-7), 65.22 (C-2), 80.83 (C-1), 178.31 (C-8). Isomer B: ^1^H NMR (400 MHz, CDCl_3_): 1.00 (s, 3H, CH_3_-9), 1.07 (s, 3H, CH_3_-10), 1.20 (d, *J* = 6.4 Hz, 3H, CH_3_-11), 1.46 (ddd, *J* = 14.4, 4.4 and 4.4 Hz, 1H, one of CH_2_-4), 1.55 (ddd, *J* = 14.4, 4.8. and 2.4 Hz, 1H, one of CH_2_-4), 1.64–1.69 (m, 1H, one of CH_2_-5), 1.85–1.94 (m, 1H, one of CH_2_-5), 2.36–2.48 (m, 2H, H-6, H-7), 3.56 (d, *J* = 9.6 Hz, 1H, H-2), 4.48 (dd, *J* = 9.6 and 6.8 Hz, 1H, H-1), ^13^C NMR (100 MHz, CDCl3): 13.48 (C-11), 18.54 (C-9), 19.89 (C-5), 29.60 (C-10), 34.00 (C-4), 35.74 (C-6), 36.97 (C-3), 43.53 (C-7), 70.83 (C-2), 81.59 (C-1), 178.30 (C-8). ESIHRMS: calculated for C_11_H_17_ClO_2_, *m*/*z* 217.0995 and 219.0969 (M + H)^+^, found 217.0999 and 219.0970, calculated for C_11_H_17_ClO_2_Na, *m*/*z* 239.0815 and 241.0788 (M + H)^+^, found 239.0823 and 241.079.

*(1S, 2R, 6R, 7S or 1R, 2S, 6R, 7R)-2-Bromo-3,3,7-trimethyl-9-oxabicyclo[4.3.0]nonan-8-one (isomer A) and (1S, 2S, 6R, 7R or 1R, 2R, 6S, 7S)-2-Bromo-3,3,7-trimethyl-9-oxabicyclo[4.3.0]nonan-8-one (isomer B)* (**5b**), Acid **3b** (0.2 g) underwent bromolactonization analogously as described for compound **3a**. The resulting product **5b** was formed as a mixture of two diastereoisomers A and B in the ratio 14%:86% with a yield of 0.26 g (90%) with the following spectral data: Isomer A: ^1^H NMR (600 MHz, CDCl_3_): 1.14 (s, 3H, CH_3_-9), 1.18 (s, 3H, CH_3_-10), 1.22 (d, *J* = 7.2 Hz, 3H, CH_3_-11), 1.29–1.34 (m, 2H, one of CH_2_-4, one of CH_2_-5), 1.61–1.66 (m, 1H, CH_2_-4), 1.71–1.76 (m, 1H, CH_2_-5), 2.74–2.76 (m, 1H, H-6), 2.80 (q, *J* = 7.2 Hz, 1H, H-7), 4.32 (m, 1H, H-2), 4.78 (dd, *J* = 3.6 and 3.6 Hz, 1H, H-1), ^13^C NMR (151 MHz, CDCl_3_): 9.51 (C-11), 19.03 (C-4), 25.69 (C-10), 31.07 (C-5), 32.48 (C-9), 33.96 (C-3), 36.45 (C-6), 41.17 (C-7), 60.33 (C-2), 81.21 (C-1), 178.31 (C-8). Isomer B: ^1^H NMR (600 MHz, CDCl_3_): 1.08 (s, 3H, CH_3_-9), 1.12 (s, 3H, CH_3_-10), 1.24 (d, *J* = 6.8 Hz, 3H, CH_3_-11), 1.52 (ddd, *J* = 14.0, 4.8 and 4.4 Hz, 1H, one of CH_2_-4), 1.63–1.67 (m, 1H, one of CH_2_-4), 1.70–1.74 (m, 1H, one of CH_2_-5), 1.92–1.99 (m, 1H, one of CH_2_-5), 2.36–2.40 (m, 1H, H-6), 2.50 (q, *J* = 6.8 Hz, 1H, H-7), 3.74 (d, *J* = 10.0 Hz, 1H, H-2), 4.67 (dd, *J* = 10.0 and 7.6 Hz, 1H, H-1), ^13^C NMR (151 MHz, CDCl_3_): 13.31 (C-11), 19.69 (C-9), 19.92 (C-5), 30.98 (C-10), 33.60 (C-4), 35.41 (C-7), 36.78 (C-3), 43.69 (C-6), 65.34 (C-2), 81.77 (C-1), 178.05 (C-8). ESIHRMS: calculated for C_11_H_17_BrO_2_, *m*/*z* 261.0490 and 263.0471 (M + H)^+^, found 261.0485 and 263.0465, calculated for C_11_H_17_BrO_2_Na, *m*/*z* 283.0310 and 285.0290 (M + H)^+^, found 283.0305 and 285.0285.

*(1S, 2R, 6R, 7S or 1R, 2S, 6R, 7R)-2-Iodo-3,3,7-trimethyl-9-oxabicyclo[4.3.0]nonan-8-one (isomer A) and (1S, 2S, 6R, 7R or 1R, 2R, 6S, 7S)-2-Iodo-3,3,7-trimethyl-9-oxabicyclo[4.3.0]nonan-8-one (isomer B)* (**6b**), Acid **3b** (0.3 g) was submitted to iodolactonization in the same way as compound **3a**. The product **6b** was formed as a mixture of two diastereoisomers A and B in the ratio 23%:77% with a yield of 0.24 g (48%). The spectral data are as follows: Isomer A: ^1^H NMR (400 MHz, CDCl_3_): 1.13 (s, 3H, CH_3_-9), 1.17 (d, *J* = 6.8 Hz, 3H, CH_3_-11), 1.22 (s, 3H, CH_3_-10), 1.24–1.29 (m, 1H, one ofCH_2_-4), 1.60–1.69 (m, 2H, one of CH_2_-4 and CH_2_-5), 2.76 (q, *J* = 6.8 Hz, 1H, H-7), 2.80–2.85 (m, 1H, H-6), 4.53–4.54 (m, 1H, H-2), 4.94 (dd, *J* = 4.0 and 3.2 Hz, 1H, H-1), ^13^C NMR (100 MHz, CDCl_3_): 9.62 (C-11), 19.35 (C-5), 22.73 (C-3), 24.85 (C-10), 32.07 (C-4), 35.57 (C-6), 36.99 (C-9), 41.69 (C-7), 45.00 (C-2), 83.48 (C-1), 178.67 (C-8). Isomer B: ^1^H NMR (400 MHz, CDCl_3_): 1.05 (s, 3H, CH_3_-9), 1.06 (s, 3H, CH_3_-10), 1.19 (d, *J* = 6.8 Hz, 3H, CH_3_-11), 1.48–1.57 (m, 1H, one of CH_2_-4), 1.64–1.73 (m, 2H, one of CH_2_-4 and one of CH_2_-5), 1.88–1.98 (m, 1H, one of CH_2_-5), 2.19–2.24 (m, 1H, H-6), 2.52 (q, *J* = 6.8 Hz, 1H, H-7), 3.84 (d, *J* = 10.4 Hz, 1H, H-2), 4.77 (dd, *J* = 10.4 and 7.2 Hz, 1H, H-1), ^13^C NMR (100 MHz, CDCl_3_): 13.24 (C-11), 20.21 (C-5), 21.92 (C-10), 31.81 (C-4), 33.84 (C-9), 34.99 (C-7), 36.62 (C-3), 43.43 (C-6), 48.49 (C-2), 83.00 (C-1), 177.92 (C-8). ESIHRMS: calculated for C_11_H_17_IO_2_, *m*/*z* 309.0352 (M + H)^+^, found 309.0352.

### 4.3. X-ray Diffraction Data

Single-crystal X-ray diffraction data were collected at 100 K on Rigaku XtaLAB Synergy R DW system (HyPix-Arc 150, Rigaku, Wrocław, Poland) κ-geometry diffractometers using Cu Kα radiation. Data reduction and analysis were carried out with the CrysAlis Pro program [33]. The structures were solved by direct methods and refined with the full-matrix least-squares technique using the SIR2019 version 19.02 [34] and Shelxl-2018/3 [35] programs. Hydrogen atoms were placed at calculated positions, and before the last cycle of refinement, all H atoms were fixed and were allowed to ride on their parent atoms. Anisotropic displacement parameters were refined for all non-hydrogen atoms.

Crystal data for B isomer of *2-iodo-3,3,7-trimethyl-9-oxabicyclo[4.3.0]nonan-8-one*: C_11_H_17_IO_2_, M = 308.14, monoclinic, P2_1_/c, a = 7.384(2) Å, b = 21.772(4) Å, c = 14.745(4) Å, β = 90.93(2)°, V = 2370.2(10) Å^3^, Z = 8, D_c_ = 1.727 Mg m^−3^, T = 100(2) K, R = 0.0360, *wR* = 0.0957 (3978 reflections with I > 2σ(*I*)) for 253 variables, CCDC 2359086.

### 4.4. Biotransformation

#### 4.4.1. Microorganisms

Biotransformation reactions were carried out using strains of filamentous fungi from the collection of the Department of Food Chemistry and Biocatalysis of Wrocław University of Life Sciences. There were the strains of the genus *Fusarium*: *F. culmorum* AM10, *F. avenaceum* AM12, *F. equiseti* AM16, *F. semitectum* AM20, *F. oxysporum* AM21, *F. solani* AM203, and strains from the genus *Absidia*: *A. coerulea* AM93, *A. glauca* AM177, *A. cylindrospora* AM336. These strains were cultured on Sabouraud Agar (0.5 g of aminobac, 0.5 g of peptone, 4 g of glucose and 1.5 g of agar dissolved in 100 mL of water) at 28 °C and stored at 4 °C after growth.

#### 4.4.2. Screening Biotransformation

Biotransformation was carried out in 300 mL Erlenmeyer flasks containing 100 mL of Sabouraud medium, consisting of 3 g of glucose and 1 g of peptone dissolved in 100 mL of water. After inoculation of the medium with the given microorganism, the mycelium was allowed to grow for three days. After this time, 10 mg of halolactone **4a**,**b**–**6a**,**b** dissolved in 1 mL of acetone was added to each flask. Shaken culture was continued for another seven days, and after three, five and seven days, about 30 mL of medium was taken with the mycelium. This mixture was extracted with dichloromethane (15 mL), dried with magnesium sulfate and analyzed by GC using a SGE BP5 column.

#### 4.4.3. Preparative Biotransformation

To 10 Erlenmeyer flasks, each containing 100 mL of Sabouraud medium with overgrown mycelium, 10 mg each (for a total of 100 mg) of bromo-**5a** or iodolactone **6a** dissolved in 10 mL of acetone was added. Shaken culture was carried out for 7 days, after which the combined contents of ten flasks were extracted with dichloromethane (3 × 30 mL). The combined organic fractions were dried with anhydrous magnesium sulfate. After evaporation of the solvent in vacuo, the products were purified by column chromatography using a mixture of hexane/acetone 6:1 as eluent. The spectral data of the compounds obtained are shown below.

*(1S, 2S, 6R, 7S or 1R, 2R, 6S, 7R)-2-Hydroxy-3,3,7-trimethyl-9-oxabicyclo[4.3.0]nonan-8-one* (**7a**), ^1^H NMR (400 MHz, CDCl_3_): 1.01 (s, 6H, CH_3_-9 and CH_3_-10), 1.19–1.27 (m, 1H, one of CH_2_-4), 1.39–1.41 (m, 1H, one of CH_2_-4), 1.43 (d, *J* = 7.2 Hz, 3H, CH_3_-11), 1.61–1.71 (m, 1H, one of CH_2_-3), 1.76–1.83 (m, 1H, one of CH_2_-3), 2.15 (s, 1H, OH), 2.18 (dd, *J* = 6.8 and 4.4 Hz, 1H, H-6), 2.82 (q, *J* = 7.2 Hz, 1H, H-7), 3.74 (ddd, *J* = 10.4, 5.2 and 5.2, 1H, H-2), 4.42 (t, *J* = 4.4 Hz, 1H, H-1), ^13^C NMR (151 MHz, CDCl_3_): 11.87 (C-11), 22.78 (C-9), 26.11 (C-3), 31.53 (C-10), 31.92 (C-5), 38.81 (C-4), 42.20 (C-7), 47.07 (C-6), 69.30 (C-2), 80.52 (C-1), 179.27 (C-8), ESIHRMS: calculated for C_11_H_18_O_3_, *m*/*z* 199.1334 (M + H)^+^, found 199.1330, calculated for C_11_H_18_O_3_Na, *m*/*z* 221.1154 (M + H)^+^, found 221.1134.

*(1S, 2R 6R, 7R or 1R, 2S, 6S, 7S)-2-Hydroxy-3,3,7-trimethyl-9-oxabicyclo[4.3.0]nonan-8-one* (**8a**), ^1^H NMR (400 MHz, CDCl_3_): 1.06 (s, 3H, CH_3_-9), 1.09 (s, 3H, CH_3_-10), 1.24–1.26 (m, 1H, one of CH_2_-4), 1.40 (d, *J* = 7.2 Hz, 3H, CH_3_-11), 1.45–1.53 (m, 1H, one of CH_2_-4), 1.56–1.65 (m, 1H, one of CH_2_-3), 1.86–1.93 (m, 1H, one of CH_2_-3), 1.99 (s, 1H, OH), 2.44 (dd, *J* = 8.4 and 7.2 Hz, 1H, H-6), 2.69 (q, *J* = 8.0 Hz, 1H, H-7), 3.85–3.91 (m, 1H, H-2), 4.35–4.40 (m, 1H, H-1), ^13^C NMR (151 MHz, CDCl_3_): 15.19 (C-11), 23.09 (C-5), 26.35 (C-3), 30.38 (C-10), 32.86 (C-4), 34.22 (C-9), 38.35 (C-7), 48.33 (C-6), 71.55 (C-2), 84.28 (C-1), 180.24 (C-8), ESIHRMS: calculated for C_11_H_18_O_3_, *m*/*z* 199.1334 (M + H)^+^, found 199.1330.

*(1S, 2R, 6R, 7R or 1R, 2S, 6S, 7S)-2-Chloro-7-hydroxy-3,3,7-trimethyl-9-oxabicyclo[4.3.0]nonan-8-one* (**9a**), ^1^H NMR (400 MHz, CDCl_3_): 1.10 (s, 3H, CH_3_-9), 1.22 (s, 3H, CH_3_-10), 1.40–1.44 (m, 1H, one of CH_2_-4), 1.59 (d, *J* = 7.2 Hz, 3H, CH_3_-11), 1.66 (td, *J* = 14.0 and 3.6 Hz, 1H, one of CH_2_-4), 1.92 (qd, *J* = 14.0 and 4.0 Hz, 1H, one of CH_2_-3), 2.12–2.17 (m, 1H, one of CH_2_-3), 2.54 (d, *J* = 8.4 Hz, 1H, H-6), 2.76 (s, 1H, OH), 3.92 (ddd, *J* = 12.8, 10.0, 5.2 Hz, 1H, H-2), 4.61 (dd, *J* = 10.0 and 8.4 Hz, 1H, H-1), ^13^C NMR (151 MHz, CDCl_3_): 24.08 (C-11), 26.56 (C-10), 30.08 (C-3), 30.31 (C-9), 32.84 (C-5), 35.06 (C-4), 55.09 (C-6), 59.71 (C-2), 73.88 (C-7), 81.16 (C-1), 179.60 (C-8), ESIHRMS: calculated for C_11_H_17_ClO_3_, *m*/*z* 233.0945 and 235.0918 (M + H)^+^, found 233.0933 and 235.0893.

*(1S, 2R, 6R, 7R or 1R, 2S, 6S, 7S)-2-Bromo-7-hydroxy-3,3,7-trimethyl-9-oxabicyclo[4.3.0]nonan-8-one* (**10a**), ^1^H NMR (400 MHz, CDCl_3_): 1.10 (s, 3H, CH_3_-9), 1.21 (s, 3H, CH_3_-10), 1.24–1.29 (m, 1H, one of CH_2_-4), 1.60 (d, *J* = 7.2 Hz, 3H, CH_3_-11), 1.66 (td, *J* = 14.0 and 3.6 Hz, 1H, one of CH_2_-4), 2.09 (qd, *J* = 13.2 and 4.0 Hz, 1H, one of CH_2_-3), 2.22–2.25 (m, 1H, one of CH_2_-3), 2.50 (dd, *J* = 8,4 and 1.2 Hz, 1H, H-6), 2.72 (s, 1H, OH), 4.00 (ddd, *J* = 12.8, 10.0, 5.6 Hz, 1H, H-2), 4.74 (dd, *J* = 10.0 and 8.4 Hz, 1H, H-1), ^13^C NMR (151 MHz, CDCl_3_): 24.35 (C-11), 26.67 (C-10), 30.07 (C-9), 31.23 (C-3), 32.84 (C-5), 35.03 (C-4), 50.59 (C-2), 55.39 (C-6), 73.86 (C-7), 81.30 (C-1), 179.44 (C-8), ESIHRMS: calculated for C_11_H_17_BrO_3_, *m*/*z* 277.0439 and 279.0420 (M + H)^+^, found 277.0425 and 279.0387.

*(1S, 2R, 6R, 7R or 1R, 2S, 6S, 7S)-2-Iodo-7-hydroxy-3,3,7-trimethyl-9-oxabicyclo[4.3.0]nonan-8-one* (**11a**), ^1^H NMR (400 MHz, CDCl_3_): 1.10 (s, 3H, CH_3_-9), 1.20 (s, 3H, CH_3_-10), 1.24 (m, 1H, one of CH_2_-4), 1.64 (d, *J* = 7.2 Hz, 3H, CH_3_-11), 1.65 (td, *J* = 14.0 and 4.0 Hz, 1H, one of CH_2_-4), 2.25 (qd, *J* = 13.6 and 4.0 Hz, 2H, CH_2_-3), 2.38 (d, *J* = 8.4 Hz, 1H, H-6), 2.58 (s, 1H, OH), 3.95 (ddd, *J* = 12.8, 10.8, 5.6 Hz, 1H, H-2), 4.61 (dd, *J* = 10.4 and 8.0 Hz, 1H, H-1), ^13^C NMR (151 MHz, CDCl_3_): 24.85 (C-11), 26.93 (C-10), 26.98 (C-2), 30.04 (C-9), 32.83 (C-5), 33.50 (C-3), 37.30 (C-4), 55.24 (C-6), 73.88 (C-7), 82.42 (C-1), 179.12 (C-8), ESIHRMS: calculated for C_11_H_17_IO_3_, *m*/*z* 325.0301 (M + H)^+^, found 325.0309.

### 4.5. Biological Tests

#### 4.5.1. Microbial Strains

Multidrug-resistant (MDR) clinical bacterial strains, namely methicillin-resistant (MR) *Staplylococcus aureus*, *S. epidermidis* and *S. haemolyticus*; vancomycin-resistant *Enterococcus faecalis* (VRE); beta-lactamase-producing (ESBL) *Eschericha coli* and *Pseudomonas aeruginosa*; and *Acinetobacter baumannii*, as well as two strains of yeast-like fungi—*Candida albicans* and *C. dubliniensis*—were used to test antimicrobial activity. In addition, a reference strain of *S. aureus* ATCC25923 was used in the study. All strains needed for the determinations can be found in the Museum of Microorganisms of the Department of Microbiology of the Medical University of Wrocław.

The bacterial strains were stored at −80 °C. They were revived in overnight culture in Tryptic Soy Broth (TSB) media (OXOID, Hampshire, UK) or Sabouraud Broth (SB) using shaking (MaxQTM 6000 incubator shaker, Thermo Scientific, Waltham, MA, USA) at 125 rpm and 37 °C. For each experiment, a fresh 18–20 h culture was prepared on Tryptic Soy Agar (TSA, OXOID, UK) or Sabouraud Agar (SA), which was then transferred to 3 mL of fresh Mueller Hinton broth (MHB, OXOID, Hampshire, UK) or SB for bacteria and fungi, respectively. The optical density of each culture was set at 10^8^ CFU/mL and then diluted to a concentration representing the primary inoculum, 10^6^ CFU/mL.

#### 4.5.2. Determination of Antimicrobial Activity

##### Determination of Inhibitory Concentration

MIC (minimum inhibitory concentration) values for the tested compounds were determined using the broth microdilution method, according to the standards of the European Committee for Antimicrobial Susceptibility Testing, (EUCAST) [36,37]. Stock solutions (20 mg/mL) of the tested lactones were prepared in DMSO (Chempur, Piekary Śląskie, Poland). Doubly concentrated compounds were diluted at concentrations ranging from 2 µg/mL to 1024 µg/mL in 96-well titration plates.

Culture in MHB or SB (10^5^ CFU/mL) was added to an appropriate liquid medium with twice-concentrated compounds. The following controls were set for the assays: blank (MHB/SB medium); negative/background compound (MHB/SB medium with the halolactones under study); growth (bacterial culture in MHB/SB) and solvent (bacterial/fungal culture in MHB/SB with the appropriate concentration of DMSO). Each experiment was performed as 3 independent experiments, each in 3 replicates.

The contents of the titration plate were stirred for 20 min (2000 rpm) and incubated for 18 h at 37 °C. The optical density (OD_600nm_) was then read (ASYS UVM340 reader, BIOCHROM Ltd., Cambridge, UK). The MIC value was calculated from the optical density counted for the test compound (taking into account the blank and background of the compound) relative to the growth control. On this basis, MIC_50_ and MIC_90_ were determined, i.e., the concentration of the modified lactone that contributed to killing the test strain at 50% and 90%, respectively.

##### Determination of the Killing Concentration

In order to determine the MBC (minimum bactericidal concentration) and MFC (minimum fungicidal concentration) values of the tested compounds, 10 µL of the reaction mixture was seeded from each well of the titer plate onto TSA or SA and incubated for 18–20 h at 37 °C.

## 5. Conclusions

As a result of several stages of organic synthesis, two groups of racemic halolactones with two methyl groups in the cyclohexane ring (at the C-5 carbon or C-3 carbon) and a methyl group in the lactone ring (C-7 carbon) were obtained. Halolactones with substituents at the C-5 carbon underwent biotransformation to hydroxylactones or halo-hydroxylactones. Their analogs with substituents at the C-3 carbon did not undergo any transformation under the influence of filamentous fungi. The biological activity tests showed that halolactones containing a chlorine or bromine atom with a gem-dimethyl moiety at the C-5 carbon in their structure are capable of inhibiting the growth of bacteria, while those containing bromolactone with two methyl groups at the C-3 carbon are capable of inhibiting yeast-like fungi. Among the biotransformation products, bromo-hydroxylactone with a gem-dimethyl moiety at the C-5 carbon was the only one to show antimicrobial activity.

## Figures and Tables

**Table 1:** Results of screening biotransformation of lactones **4a**–**6a** (mixtures of diastereoisomers A and B) carried out for 7 days by strains of the genus *Fusarium* (in % according to GC).

Strain	Chlorolactone 4a	Product	Bromolactone 5a	Hydroxylactone 7a	Hydroxylactone 8a	Iodolactone 6a	Hydroxylactone 7a	Hydroxylactone 8a
*F. culmorum* AM10	100	-	69.6	26.3	4.1	81.2	12.6	6.2
*F. avenaceum* AM12	100	-	42.2	50.9	6.9	50.4	32.9	16.7
*F. equiseti* AM16	100	-	69.2	24.2	6.4	75.6	24.4	-
*F. semitectum* AM20	100	-	87.4	9.4	3.2	91.3	8.7	-
*F. oxysporum* AM21	100	-	78.0	15.3	6.7	90.0	10.0	-
*F. solani* AM203	100	-	79.2	17.4	3.4	77.4	15.0	7.6

**Table 2:** Results of screening biotransformation of lactones **4a**–**6a** (mixtures of diastereoisomers A and B) carried out for 7 days by strains of the genus *Absidia* (in % according to GC).

Strain	Chlorolactone 4a	Chloro-Hydroxylactone 9a	Bromolactone 5a	Hydroxylactone 7a	Hydroxylactone 8a	Bromo-Hydroxylactone 10a	Iodolactone 6a	Hydroxylactone 7a	Hydroxylactone 8a	Iodo-Hydroxylactone 11a
*A. coerulea* AM93	100	-	100	-	-	-	100	-	-	-
*A. glauca* AM177	80.2	19.8	58.3	5.8	10.5	25.4	50.8	13.4	13.0	22.8
*A. cylindrospora* AM336	59.0	41.0	36.4	-	-	63.6	30.7	-	-	69.3

**Table 3:** Results of the preparative-scale biotransformation of lactones **5a** and **6a** (mixtures of diastereoisomers A and B) by *F. avenaceum* AM12 after 7 days of incubation (in % according to GC).

Substrate	Unreacted Substrate (%)	Lactone 7a (%)	Isolated Yield (g/%)	Lactone 8a (%)	Isolated Yield (g/%)
Bromolactone **5a**	50.4	44.6	0.02/31.1	5.0	-
Iodolactone **6a**	42.3	39.1	0.017/26.4	18.6	0.0066/10.3

**Table 4:** Results of the preparative-scale biotransformation of lactones **4a**, **5a** and **6a** (mixtures of diastereoisomers A and B) by *A. cylindrospora* AM336 after 7 days of incubation (in % according to GC).

Substrate	Unreacted Substrate (%)	Product			Isolated Yield (g/%)
		Lactone 9a (%)	Lactone 10a (%)	Lactone 11a (%)	
Chlorolactone **4a**	51.1	48.9	-	-	0.0326/30.4
Bromolactone **5a**	41.4	-	58.6	-	0.026/24.5
Iodolactone 6a	36.3	-	-	63.7	0.03/28.5

**Table 5:** The values of enantiospecificity and optical purity of lactones **7a**–**11a**.

Substrate	Product	ee (%)	${\left[a\right]}_{20}^{D}$
Bromolactone **5a**	Hydroxylactone **7a**	48.9	−23.12 (C = 0.95, CHCl_3_)
Iodolactone **6a**	Hydroxylactone **7a**	56.3	−23.88 (C = 0.80, CHCl_3_)
Iodolactone **6a**	Hydroxylactone **8a**	60.4	+12.86 (C = 0.31, CHCl_3_)
Chlorolactone **4a**	Chloro-hydroxylactone **9a**	1.6	−1.12 (C = 1.55, CHCl_3_)
Bromolactone **5a**	Bromo-hydroxylactone **10a**	1.1	−1.62 (C = 1.25, CHCl_3_)
Iodolactone **6a**	Iodo-hydroxylactone **11a**	6.1	−2.57 (C = 0.78, CHCl_3_)

**Table 6:** Resistance profile of the strains used in the study.

Strain	Resistance Profile	Sensitivity Profile
***Staphylococcus aureus* S16**	FOX; ERY; CLI; CIP	VAN; GM; SXT; NET; TEC; LZD
***Enterococcus faecalis* 37VRE**	VAN; GM	AMP; IMP; TEC
***Staphylococcus epidermidis* S22**	FOX; ERY; CLI; SXT	CIP; VAN; GM; NET; TEC; TET
***Staphylococcus haemolyticus* 9**	GM; ERY; MY	VAN
***Escherichia coli* 1471**	CIP; GM; TOB; CTX; CAZ; CXM; AMC; AZM; FEP; TZP; MEM; AMK; SAM	SXT; IMP; ETP; DOR
***Pseudomonas aeruginosa* 5894p**	IMP; MEM; DOR; CIP; GM; TOB; AMK; NET; FEP; TZP; CAZ; AZM	CST
***Acinetobacter baumannii* 2800**	CIP; GM; SXT; TOB; CAZ; FEP; TZP; MEM; LVX; PIP; AMK; IMP	CST
***Candida albicans* 31**	ITC (I)	5FC; AMB; KTC; FLC; MIC
***Candida dubliniensis* 1745**	5FC	AMB, MIC; KTC; ITC; FLC

AMB (amphotericin B); AMC (amoxicillin/clavulanic acid); AMK (amikacin); AMP (ampicillin); AZM (aztreonam); CAZ (ceftazidime); CIP (ciprofloxacin); CLI (clindamycin); CST (colistin); CTX (cefotaxim); CXM (cefuroxime); DOR (doripenem); ETM (ertapenem); ERY (erythromycin); FEP (cefepime); FLC (fluconazole); FOX (cefoxitin); GM (gentamicin); IMP (imipenem); ITC (itraconazole); KET (ketoconazole); LVX (levofloxacin); LZD (linezolid); MEM (meropenem); MIC (miconazole); MY (lincomycin); NET (netilmicin); PIP (piperacillin); SAM (ampicillin/sulbactam); SXT (trimethoprim-sulfamethoxazole); TEC (teicoplanin); TET (tetracycline); TOB (tobramycin); VAN (vancomycin); VRC (voriconazole); TZP (piperacillin/tazobactam); 5FC (fluorocytozine); I—intermediate.

**Table 7:** Antibacterial and antifungal activity of tested strains of clinical origin.

The Values of MIC_50_/MIC_90_ (MBC/MFC) [µg/mL]										
Lactone	*S. aureus* MRSA	*E. faecalis* VRE	*S. epidermidis* MRCNS	*S. aureus ATCC25923*	*S. haemolyticus* MRCNS	*C. albicans*	*C. dubliniensis*	*E. coli ESBL*	*P. aeruginosa* ESBL	*A. baumannii*
**4a**	128/128 (256)	>512/>512 (>512)	>512/>512 (>512)	256/256 (>512)	512/>512 (>512)	>512/>512 (>512)	>512/>512 (>512)	>512/>512 (>512)	>512/>512 (>512)	>512/>512 (>512)
**5a**	256/256 (>512)	512/>512 (>512)	>512/>512 (>512)	256/512 (512)	512/512 (512)	512/512 (512)	512/512 (512)	>512/>512 (>512)	>512/>512 (>512)	512/>512 (>512)
**6a**	512/512 (>512)	512/>512 (>512)	512/>512 (>512)	512/>512 (>512)	512/>512 (>512)	256/>512 (>512)	512/>512 (>512)	>512/>512 (>512)	>512/>512 (>512)	>512/>512 (>512)
**7a**	512/512 (>512)	256/512 (>512)	256/512 (>512)	>512/>512 (>512)	>512/>512 (>512)	>512/>512 (>512)	>512/>512 (>512)	>512/>512 (>512)	>512/>512 (>512)	>512/>512 (>512)
**8a**	>512/>512 (>512)	>512/>512 (>512)	>512/>512 (>512)	>512/>512 (>512)	>512/>512 (>512)	>512/>512 (>512)	>512/>512 (>512)	>512/>512 (>512)	>512/>512 (>512)	>512/>512 (>512)
**9a**	>512/>512 (>512)	>512/>512 (>512)	512/>512 (>512)	>512/>512 (>512)	>512/>512 (>512)	>512/>512 (>512)	>512/>512 (>512)	>512/>512 (>512)	>512/>512 (>512)	>512/>512 (>512)
**10a**	256/>512 (>512)	>512/>512 (>512)	512/>512 (>512)	>512/>512 (>512)	>512/>512 (>512)	>512/>512 (>512)	>512/>512 (>512)	>512/>512 (>512)	>512/>512 (>512)	>512/>512 (>512)
**11a**	512/>512 (>512)	512/>512 (>512))	>512/>512 (>512)	>512/>512 (>512)	>512/>512 (>512)	>512/>512 (>512)	>512/>512 (>512)	>512/>512 (>512)	>512/>512 (>512)	>512/>512 (>512)
**4b**	>512/>512 (>512)	>512/>512 (>512)	>512/>512 (>512)	>512/>512 (>512)	>512/>512 (>512)	>512/>512 (>512)	>512/>512 (>512)	>512/>512 (>512)	>512/>512 (>512)	>512/>512 (>512)
**5b**	512/>512 (>512)	512/512 (>512)	512/>512 (>512)	>512/>512 (>512)	512/>512 (>512)	128/256 (512)	64/256 (512)	>512/>512 (>512)	>512/>512 (>512)	512/>512 (>512)
**6b**	512/>512 (>512)	512/>512 (>512)	512/>512 (>512)	>512/>512 (>512)	512/>512 (>512)	>512/>512 (>512)	512/>512 (>512)	>512/>512 (>512)	>512/>512 (>512)	>512/>512 (>512)

**Table 8:** Antibacterial and antifungal activity of selected strains of clinical origin.

Values MIC_50_ [µg/mL]				
Lactone	*S. aureus* MRSA	*S. aureus* ATCC 25923	*C. albicans* 31	*C. dubliniensis* 1745
**4a**-A	64	64	NT	NT
**4a**-B	>512	>512	NT	NT
**5a**-A	128	128	NT	NT
**5a**-B	>512	>512	NT	NT
**6a**-A	NT	NT	>512	NT
**5b**-A	NT	NT	256	256
**5b**-B	NT	NT	64	>512

NT—not tested.

## Data Availability

Data are contained within the article and Supplementary Materials.

## Supplementary Materials

The following supporting information can be downloaded at https://www.mdpi.com/article/10.3390/molecules29122820/s1: Figures S1–S10: Chromatograms of substrates, Figures S11–S30: ^1^H NMR, COSY, HMQC, ^13^C NMR, HRMS spectrum of esters and acids, Figures S31–S95: ^1^H NMR, COSY, HMQC, ^13^C NMR, NOESY, HRMS spectrum of halolactones, Figures S96–S105: ^1^H NMR, COSY, HMQC, ^13^C NMR, HRMS spectrum of hydroxylactones, Figures S106–S120: ^1^H NMR, COSY, HMQC, ^13^C NMR, HRMS spectrum of halo-hydroxylactones, Figures S121–S126: Chiral chromatograms of hydroxylactones and halo-hydroxylactones.
