# Supplementary material for: New Hydroxylactones and Chloro-Hydroxylactones Obtained by Biotransformation of Bicyclic Halolactones and Their Antibacterial Activity
Source: Molecules. 2024 Jun 13;29(12):2820. doi: 10.3390/molecules29122820 (PMC11206757; doi:10.3390/molecules29122820)
Supplement: Supplementary file 1 [file molecules-29-02820-s001.zip › Supplementary information.pdf]

Supplementary Materials

New hydroxylactones and chloro-hydroxylactones obtained by biotransformation of bicyclic halolactones and their antibacterial activity

Figure S1. Chromatogram of ester **2a** (mixture of isomers A (major) and B (minor))
Figure S2. Chromatogram of ester **2b** (mixture of isomers A (minor) and B (major))
Figure S3. Chromatogram of acid **3a** (mixture of isomers A (major) and B (minor))
Figure S4. Chromatogram of acid **2b** (mixture of isomers A (minor) and B (major))
Figure S5. Chromatogram of chlorolactone **4a** (mixture of isomers A (major) and B (minor))
Figure S6. Chromatogram of bromolactone **5a** (mixture of isomers A (major) and B (minor))
Figure S7. Chromatogram of iodolactone **6a** (mixture of isomers A (major) and B (minor))
Figure S8. Chromatogram of chlorolactone **4b** (mixture of isomers A (minor) and B (major))
Figure S9. Chromatogram of bromolactone **5b** (mixture of isomers A (minor) and B (major))
Figure S10. Chromatogram of iodolactone **6b** (mixture of isomers A (minor) and B (major))
Figure S11. ^{1}H NMR (400 MHz, $CDCl_3$) spectrum of ester **2a** (mixture of isomers A and B)
Figure S12. COSY (100 MHz, $CDCl_3$) spectrum of ester **2a** (mixture of isomers A and B)
Figure S13. HMQC (100 MHz, $CDCl_3$) spectrum of ester **2a** (mixture of isomers A and B)
Figure S14. ^{13}C NMR (100 MHz, $CDCl_3$) spectrum of ester **2a** (mixture of isomers A and B)
Figure S15. HRMS spectrum of ester **2a**
Figure S16. ^{1}H NMR (400 MHz, $CDCl_3$) spectrum of ester **2b** (mixture of isomers A and B)
Figure S17. COSY (100 MHz, $CDCl_3$) spectrum of ester **2b** (mixture of isomers A and B)
Figure S18. HMQC (100 MHz, $CDCl_3$) spectrum of ester **2b** (mixture of isomers A and B)
Figure S19. ^{13}C NMR (100 MHz, $CDCl_3$) spectrum of ester **2b** (mixture of isomers A and B)
Figure S20. HRMS spectrum of ester **2b**
Figure S21. ^{1}H NMR (400 MHz, $CDCl_3$) spectrum of acid **3a** (mixture of isomers A and B)
Figure S22. COSY (100 MHz, $CDCl_3$) spectrum of acid **3a** (mixture of isomers A and B)
Figure S23. HMQC (100 MHz, $CDCl_3$) spectrum of acid **3a** (mixture of isomers A and B)
Figure S24. ^{13}C NMR (100 MHz, $CDCl_3$) spectrum of acid **3a** (mixture of isomers A and B)
Figure S25. HRMS spectrum of acid **3a**
Figure S26. ^{1}H NMR (400 MHz, $CDCl_3$) spectrum of acid **3b** (mixture of isomers A and B)
Figure S27. COSY (100 MHz, $CDCl_3$) spectrum of acid **3b** (mixture of isomers A and B)
Figure S28. HMQC (100 MHz, $CDCl_3$) spectrum of acid **3b** (mixture of isomers A and B)
Figure S29. ^{13}C NMR (100 MHz, $CDCl_3$) spectrum of acid **3b** (mixture of isomers A and B)
Figure S30. HRMS spectrum of acid **3b** (mixture of isomers A and B)
Figure S31. ^{1}H NMR (600 MHz, $CDCl_3$) spectrum of chlorolactone **4a-A**

Figure S32. COSY (151 MHz, $CDCl_3$) spectrum of chlorolactone **4a-A**
Figure S33. HMQC (151 MHz, $CDCl_3$) spectrum of chlorolactone **4a-A**
Figure S34. ^{13}C NMR (151 MHz, $CDCl_3$) spectrum of chlorolactone **4a-A**
Figure S35.NOESY NMR (151 MHz, $CDCl_3$) spectrum of chlorolactone **4a-A**
Figure S36. ^{1}H NMR (400 MHz, $CDCl_3$) spectrum of chlorolactone **4a-B**
Figure S37. COSY (100 MHz, $CDCl_3$) spectrum of chlorolactone **4a-B**
Figure S38. HMQC (100 MHz, $CDCl_3$) spectrum of chlorolactone **4a-B**
Figure S39. ^{13}C NMR (100 MHz, $CDCl_3$) spectrum of chlorolactone **4a-B**
Figure S40.NOESY NMR (151 MHz, $CDCl_3$) spectrum of chlorolactone **4a-B**
Figure S41. HRMS spectrum chlorolactone **4a**
Figure S42. ^{1}H NMR (600 MHz, $CDCl_3$) spectrum of bromolactone **5a-A**
Figure S43. COSY (151 MHz, $CDCl_3$) spectrum of bromolactone **5a-A**
Figure S44. HMQC (151 MHz, $CDCl_3$) spectrum of bromolactone **5a-A**
Figure S45. ^{13}C NMR (151 MHz, $CDCl_3$) spectrum bromolactone **5a-A**
Figure S46.NOESY NMR (151 MHz, $CDCl_3$) spectrum bromolactone **5a-A**
Figure S47. ^{1}H NMR (600 MHz, $CDCl_3$) spectrum of bromolactone **5a-B**
Figure S48. COSY (151 MHz, $CDCl_3$) spectrum of bromolactone **5a-B**
Figure S49. HMQC (151 MHz, $CDCl_3$) spectrum of bromolactone **5a-B**
Figure S50. ^{13}C NMR (151 MHz, $CDCl_3$) spectrum bromolactone **5a-B**
Figure S51.NOESY NMR (151 MHz, $CDCl_3$) spectrum bromolactone **5a-B**
Figure S52. HRMS spectrum bromolactone **5a**
Figure S53. ^{1}H NMR (400 MHz, $CDCl_3$) spectrum of iodolactone **6a-A**
Figure S54. COSY (100 MHz, $CDCl_3$) spectrum of iodolactone **6a-A**
Figure S55. HMQC (100 MHz, $CDCl_3$) spectrum of iodolactone **6a-A**
Figure S56. ^{13}C NMR (100 MHz, $CDCl_3$) spectrum of iodolactone **6a-A**
Figure S57.NOESY NMR (151 MHz, $CDCl_3$) spectrum iodolactone **6a-A**
Figure S58. ^{1}H NMR (400 MHz, $CDCl_3$) spectrum of iodolactone **6a-B+A**
Figure S59. COSY (100 MHz, $CDCl_3$) spectrum of iodolactone **6a-B+A**
Figure S60. HMQC (100 MHz, $CDCl_3$) spectrum of iodolactone **6a-B+A**
Figure S61. ^{13}C NMR (100 MHz, $CDCl_3$) spectrum of iodolactone **6a-B+A**
Figure S62. HRMS spectrum of iodolactone **6a**
Figure S63. ^{1}H NMR (400 MHz, $CDCl_3$) spectrum of chlorolactone **4b-A**
Figure S64. COSY (100 MHz, $CDCl_3$) spectrum of chlorolactone **4b-A**
Figure S65. HMQC (100 MHz, $CDCl_3$) spectrum of chlorolactone **4b-A**
Figure S66. ^{13}C NMR (100 MHz, $CDCl_3$) spectrum of chlorolactone **4b-A**
Figure S67.NOESY NMR (151 MHz, $CDCl_3$) spectrum of chlorolactone **4b-A**

Figure S68. ^{1}H NMR (400 MHz, $CDCl_3$) spectrum of chlorolactone **4b-B**
Figure S69. COSY (100 MHz, $CDCl_3$) spectrum of chlorolactone **4b-B**
Figure S70. HMQC (100 MHz, $CDCl_3$) spectrum of chlorolactone **4b-B**
Figure S71. ^{13}C NMR (100 MHz, $CDCl_3$) spectrum of chlorolactone **4b-B**
Figure S72.NOESY NMR (151 MHz, $CDCl_3$) spectrum of chlorolactone **4b-B**
Figure S73. HRMS spectrum chlorolactone **4b**
Figure S74. ^{1}H NMR (600 MHz, $CDCl_3$) spectrum of bromolactone **5b-A**
Figure S75. COSY (151 MHz, $CDCl_3$) spectrum of bromolactone **5b-A**
Figure S76. HMQC (151 MHz, $CDCl_3$) spectrum of bromolactone **5b-A**
Figure S77. ^{13}C NMR (151 MHz, $CDCl_3$) spectrum bromolactone **5b-A**
Figure S78.NOESY NMR (151 MHz, $CDCl_3$) spectrum bromolactone **5b-A**
Figure S79. ^{1}H NMR (600 MHz, $CDCl_3$) spectrum of bromolactone **5b-B**
Figure S80. COSY (151 MHz, $CDCl_3$) spectrum of bromolactone **5b-B**
Figure S81. HMQC (151 MHz, $CDCl_3$) spectrum of bromolactone **5b-B**
Figure S82. ^{13}C NMR (151 MHz, $CDCl_3$) spectrum bromolactone **5b-B**
Figure S83.NOESY NMR (151 MHz, $CDCl_3$) spectrum bromolactone **5b-B**
Figure S84. HRMS spectrum bromolactone **5b**
Figure S85. ^{1}H NMR (400 MHz, $CDCl_3$) spectrum of iodolactone **6b-A**
Figure S86. COSY (100 MHz, $CDCl_3$) spectrum of iodolactone **6b-A**
Figure S87. HMQC (100 MHz, $CDCl_3$) spectrum of iodolactone **6b-A**
Figure S88. ^{13}C NMR (100 MHz, $CDCl_3$) spectrum of iodolactone **6b-A**
Figure S89.NOESY NMR (151 MHz, $CDCl_3$) spectrum iodolactone **6b-A**
Figure S90. ^{1}H NMR (400 MHz, $CDCl_3$) spectrum of iodolactone **6b-B**
Figure S91. COSY (100 MHz, $CDCl_3$) spectrum of iodolactone **6b-B**
Figure S92. HMQC (100 MHz, $CDCl_3$) spectrum of iodolactone **6b-B**
Figure S93. ^{13}C NMR (100 MHz, $CDCl_3$) spectrum of iodolactone **6b-B**
Figure S94.NOESY NMR (151 MHz, $CDCl_3$) spectrum iodolactone **6b-B**
Figure S95. HRMS spectrum of iodolactone **6b**
Figure S96. ^{1}H NMR (400 MHz, $CDCl_3$) spectrum of hydroxylactone **7a**
Figure S97. COSY (100 MHz, $CDCl_3$) spectrum of hydroxylactone **7a**
Figure S98. HMQC (100 MHz, $CDCl_3$) spectrum of hydroxylactone **7a**
Figure S99. ^{13}C NMR (100 MHz, $CDCl_3$) spectrum of hydroxylactone **7a**
Figure S100. HRMS spectrum of hydroxylactone **7a**
Figure S101. ^{1}H NMR (400 MHz, $CDCl_3$) spectrum of hydroxylactone **8a**
Figure S102. COSY (100 MHz, $CDCl_3$) spectrum of hydroxylactone **8a**
Figure S103. HMQC (100 MHz, $CDCl_3$) spectrum of hydroxylactone **8a**

Figure S104. ^{13}C NMR (100 MHz, $CDCl_3$) spectrum of hydroxylactone **8a**
Figure S105. HRMS spectrum of bromo-hydroxylactone **8a**
Figure S106. ^{1}H NMR (400 MHz, $CDCl_3$) spectrum of chloro-hydroxylactone **9a**
Figure S107. COSY (100 MHz, $CDCl_3$) spectrum of chloro-hydroxylactone **9a**
Figure S108. HMQC (100 MHz, $CDCl_3$) spectrum of chloro-hydroxylactone **9a**
Figure S109. ^{13}C NMR (100 MHz, $CDCl_3$) spectrum of chloro-hydroxylactone **9a**
Figure S110. HRMS spectrum of chloro-hydroxylactone **9a**
Figure S111. ^{1}H NMR (400 MHz, $CDCl_3$) spectrum of bromo-hydroxylactone **10a**
Figure S112. COSY (100 MHz, $CDCl_3$) spectrum of bromo-hydroxylactone **10a**
Figure S113. HMQC (100 MHz, $CDCl_3$) spectrum of bromo-hydroxylactone **10a**
Figure S114. ^{13}C NMR (100 MHz, $CDCl_3$) spectrum of bromo-hydroxylactone **10a**
Figure S115. HRMS spectrum of bromo -hydroxylactone **10a**
Figure S116. ^{1}H NMR (400 MHz, $CDCl_3$) spectrum of iodo-hydroxylactone **11a**
Figure S117. COSY (100 MHz, $CDCl_3$) spectrum of iodo-hydroxylactone **11a**
Figure S118. HMQC (100 MHz, $CDCl_3$) spectrum of iodo-hydroxylactone **11a**
Figure S119. ^{13}C NMR (100 MHz, $CDCl_3$) spectrum of iodo-hydroxylactone **11a**
Figure S120. HRMS spectrum of iodo-hydroxylactone **11a**
Figure S121. Chiral chromatogram of hydroxylactone **7a** obtained from bromolactone **5a**
Figure S122. Chiral chromatogram of hydroxylactone **7a** obtained from iodolactone **6a**
Figure S123. Chiral chromatogram of hydroxylactone **8a** obtained from iodolactone **6a**
Figure S124. Chiral chromatogram of chloro-hydroxylactone **9a**
Figure S125. Chiral chromatogram of bromo-hydroxylactone **10a**
Figure S126. Chiral chromatogram of iodo-hydroxylactone **11a**

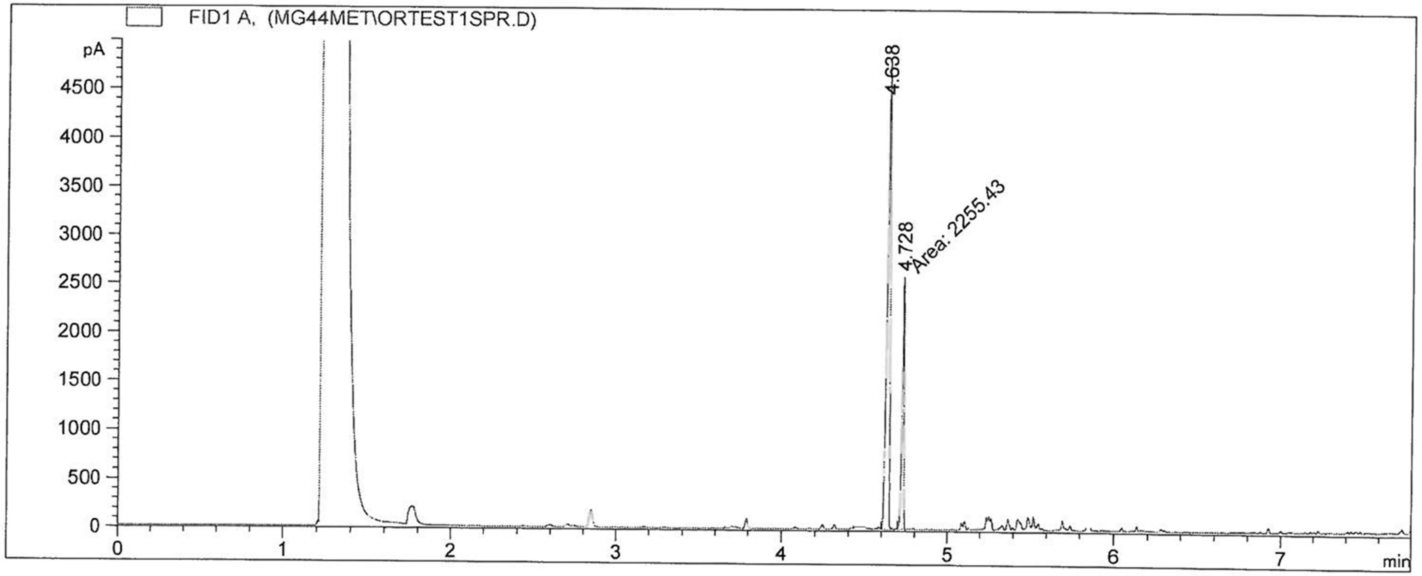

Peak #	RetTime [min]	Type	Width [min]	Area [pA*s]	Height [pA]	Area %
1	4.638	VV S	0.0190	5859.38916	4808.77393	72.20602
2	4.728	MM	0.0145	2255.43213	2587.50635	27.79398

Figure S1. Chromatogram of ester **2a** (mixture of isomers A (major) and B (minor))

GC program: injector 150 ºC, detector (FID) 300 ºC, 100 ºC/1 min, ramp 25 ºC/min to 220 ºC, ramp 40 ºC/min to 300 ºC.

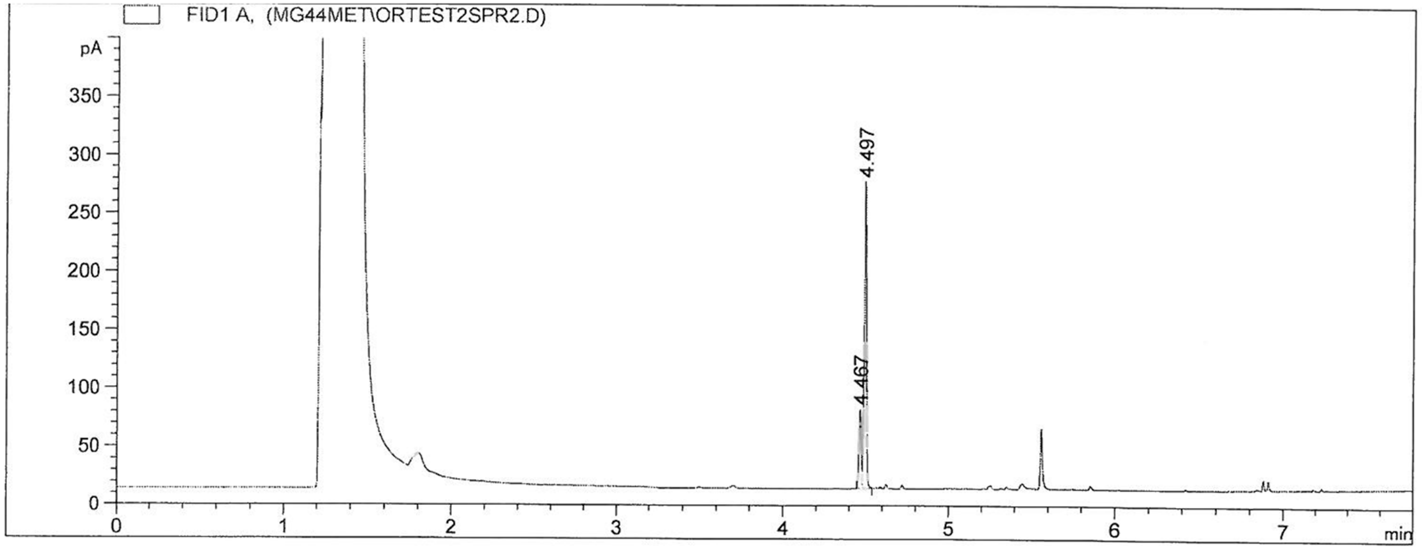

Peak #	RetTime [min]	Type	Width [min]	Area [pA*s]	Height [pA]	Area %
1	4.467	BV	0.0151	63.37798	66.60393	20.18971
2	4.497	VB	0.0152	250.53424	261.84799	79.81029

Figure S2. Chromatogram of ester **2b** (mixture of isomers A (minor) and B (major))

GC program: injector 150 ºC, detector (FID) 300 ºC, 100 ºC/1 min, ramp 25 ºC/min to 220 ºC, ramp 40 ºC/min to 300 ºC.

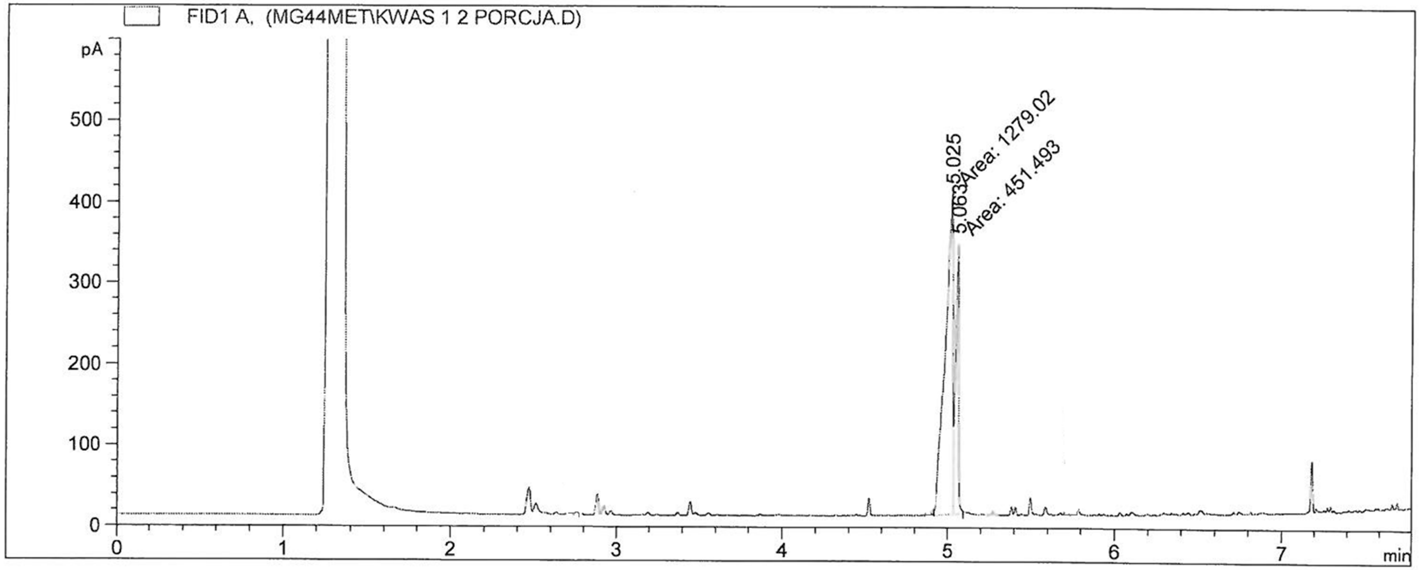

Peak #	RetTime [min]	Type	Width [min]	Area [pA*s]	Height [pA]	Area %
1	5.025	MM	0.0532	1279.01746	400.55502	73.90984
2	5.063	MM	0.0222	451.49301	339.21732	26.09016

Figure S3. Chromatogram of acid **3a** (mixture of isomers A (major) and B (minor))

GC program: injector 150 ºC, detector (FID) 300 ºC, 100 ºC/1 min, ramp 25 ºC/min to 220 ºC, ramp 40 ºC/min to 300 ºC.

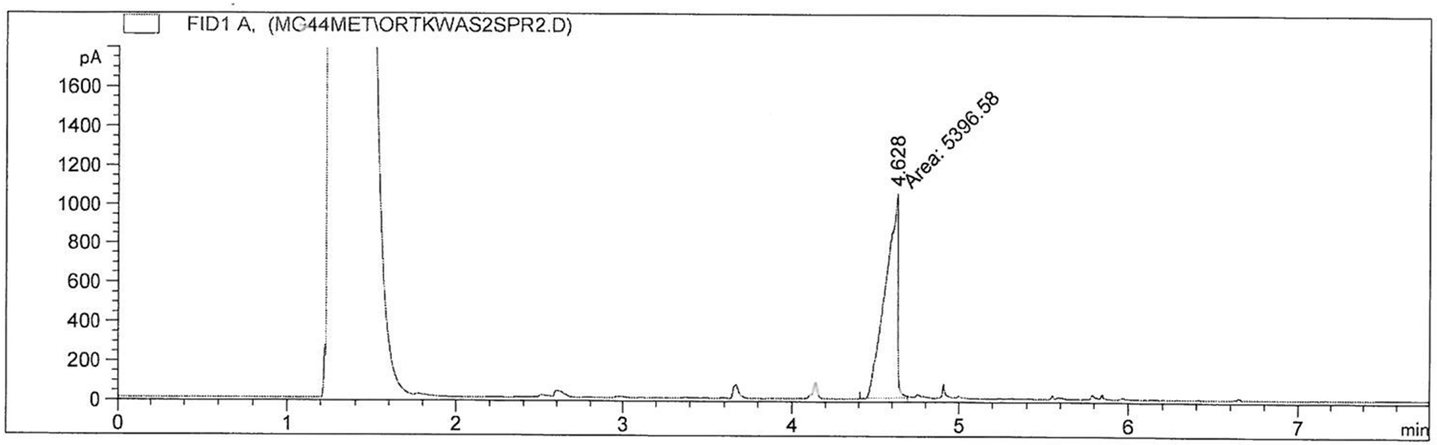

Peak #	RetTime [min]	Type	Width [min]	Area [pA*s]	Height [pA]	Area %
1	4.628	MM	0.0854	5396.57568	1053.76794	1.000e2

Figure S4. Chromatogram of acid **3b** (mixture of isomers A (minor) and B (major))

GC program: injector 150 ºC, detector (FID) 300 ºC, 100 ºC/1 min, ramp 25 ºC/min to 220 ºC, ramp 40 ºC/min to 300 ºC.

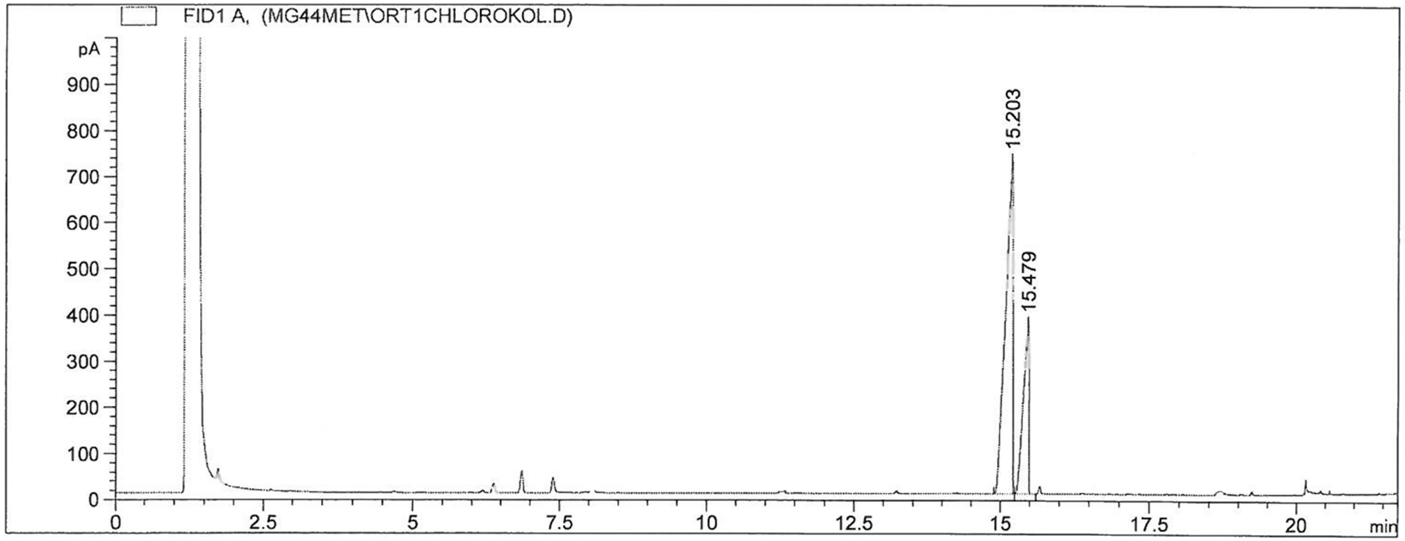

Peak #	RetTime [min]	Type	Width [min]	Area [pA*s]	Height [pA]	Area %
1	15.203	BV	0.1020	6169.53418	734.11865	71.87372
2	15.479	VV	0.0788	2414.31860	380.44421	28.12628

Figure S5. Chromatogram of chlorolactone **4a** (mixture of isomers A (major) and B (minor))

GC program: injector 200 ºC, detector (FID) 300 ºC, 120 ºC/5 min, ramp 5 ºC/min to 200 ºC, ramp 40 ºC/min to 300 ºC.

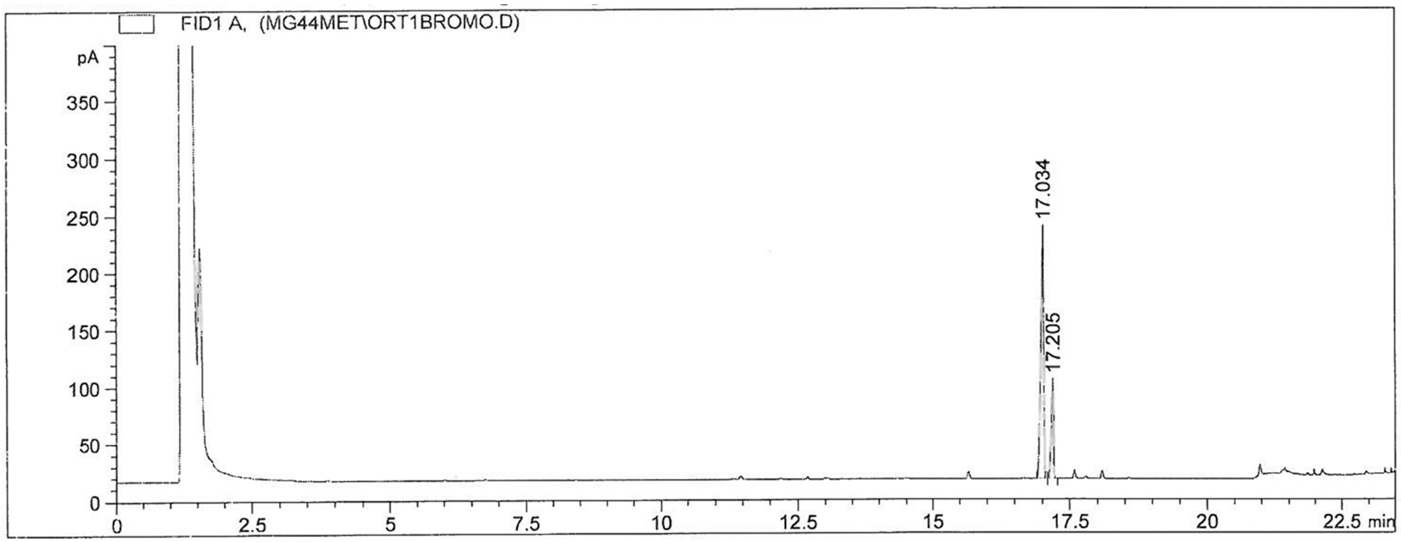

Peak #	RetTime [min]	Type	Width [min]	Area [pA*s]	Height [pA]	Area %
1	17.034	BV	0.0481	826.06482	222.33069	76.09866
2	17.205	VB	0.0417	259.45337	87.74355	23.90134

Figure S6. Chromatogram of bromolactone **5a** (mixture of isomers A (major) and B (minor))

GC program: injector 200 ºC, detector (FID) 300 ºC, 120 ºC/5 min, ramp 5 ºC/min to 200 ºC, ramp 40 ºC/min to 300 ºC.

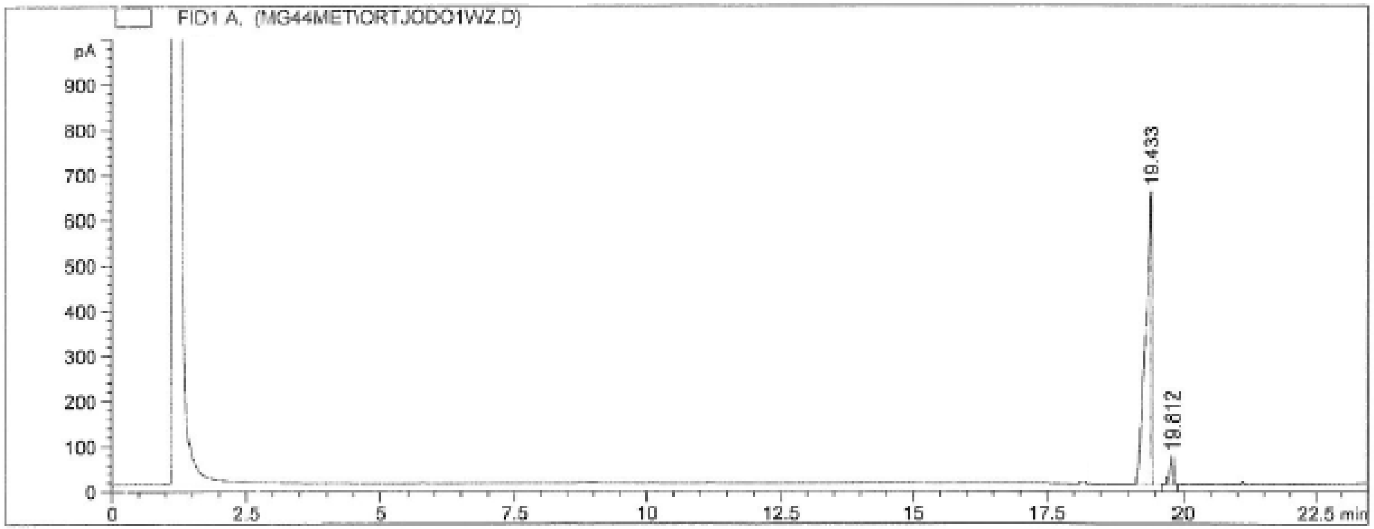

Peak #	RetTime [min]	Type	Width [min]	Area [pA*s]	Height [pA]	Area %
1	19.433	BB	0.0949	4851.34814	645.53229	96.55914
2	19.812	BB	0.0405	172.87650	61.97029	3.44086

Figure S7. Chromatogram of iodolactone **6a** (mixture of isomers A (major) and B (minor))

GC program: injector 200 ºC, detector (FID) 300 ºC, 120 ºC/5 min, ramp 5 ºC/min to 200 ºC, ramp 40 ºC/min to 300 ºC.

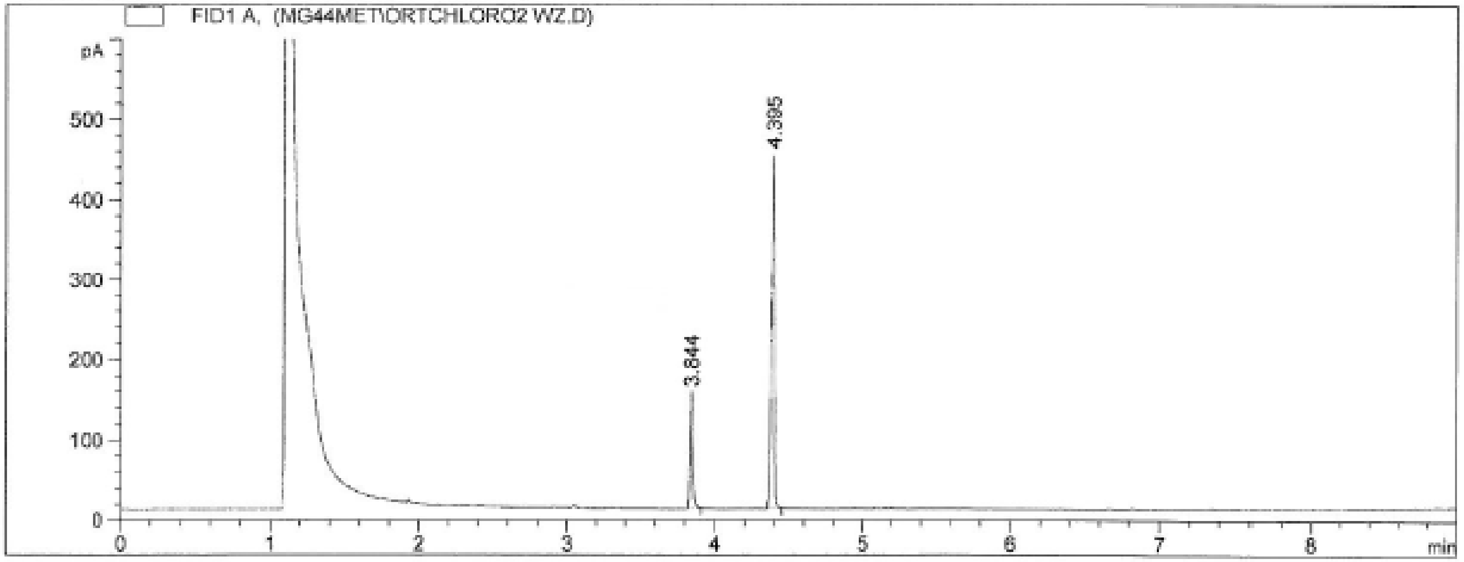

Peak #	RetTime [min]	Type	Width [min]	Area [pA*s]	Height [pA]	Area %
1	3.844	BB	0.0181	174.86308	144.90402	21.54312
2	4.395	BB	0.0232	636.82550	442.38522	78.45688

Figure S8. Chromatogram of chlorolactone **4b** (mixture of isomers A (minor) and B (major))

GC program: injector 250 ºC, detector (FID) 300 ºC, 160 ºC/1 min, ramp 15 ºC/min to 230 ºC, ramp 30 ºC/min to 300 ºC.

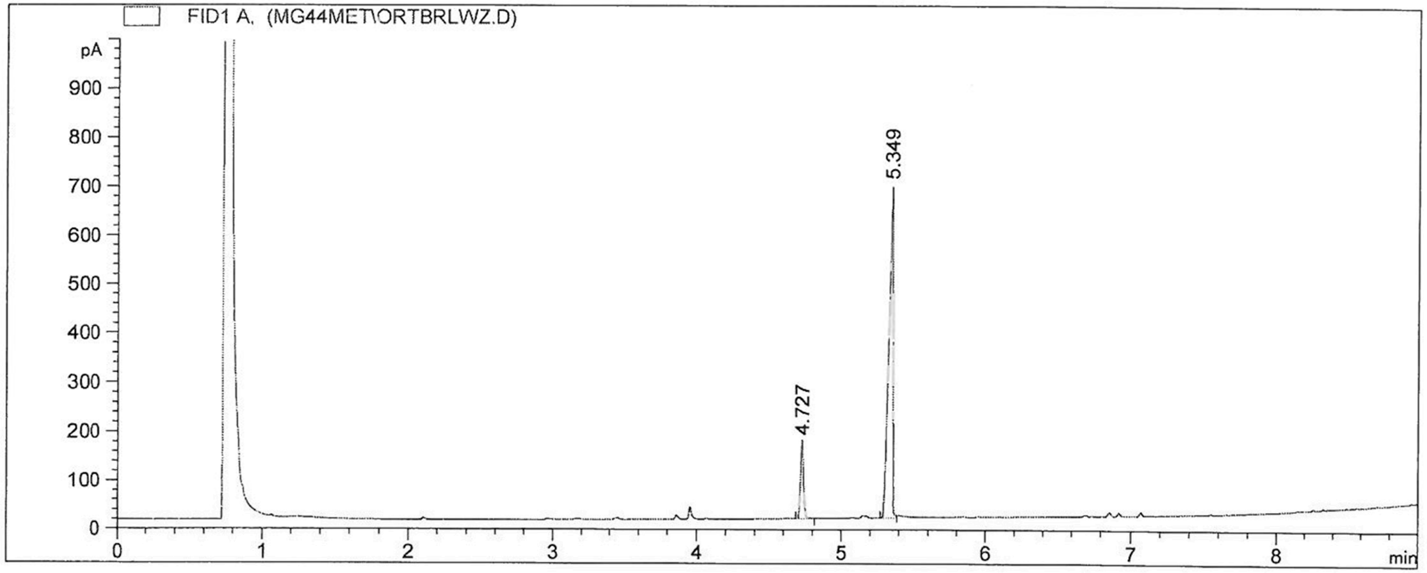

Peak #	RetTime [min]	Type	Width [min]	Area [pA*s]	Height [pA]	Area %
1	4.727	VV	0.0223	229.77773	160.76804	14.36784
2	5.349	VV	0.0307	1369.47290	680.99969	85.63216

Figure S9. Chromatogram of bromolactone **5b** (mixture of isomers A (minor) and B (major))

GC program: injector 250 ºC, detector (FID) 300 ºC, 160 ºC/1 min, ramp 15 ºC/min to 230 ºC, ramp 30 ºC/min to 300 ºC.

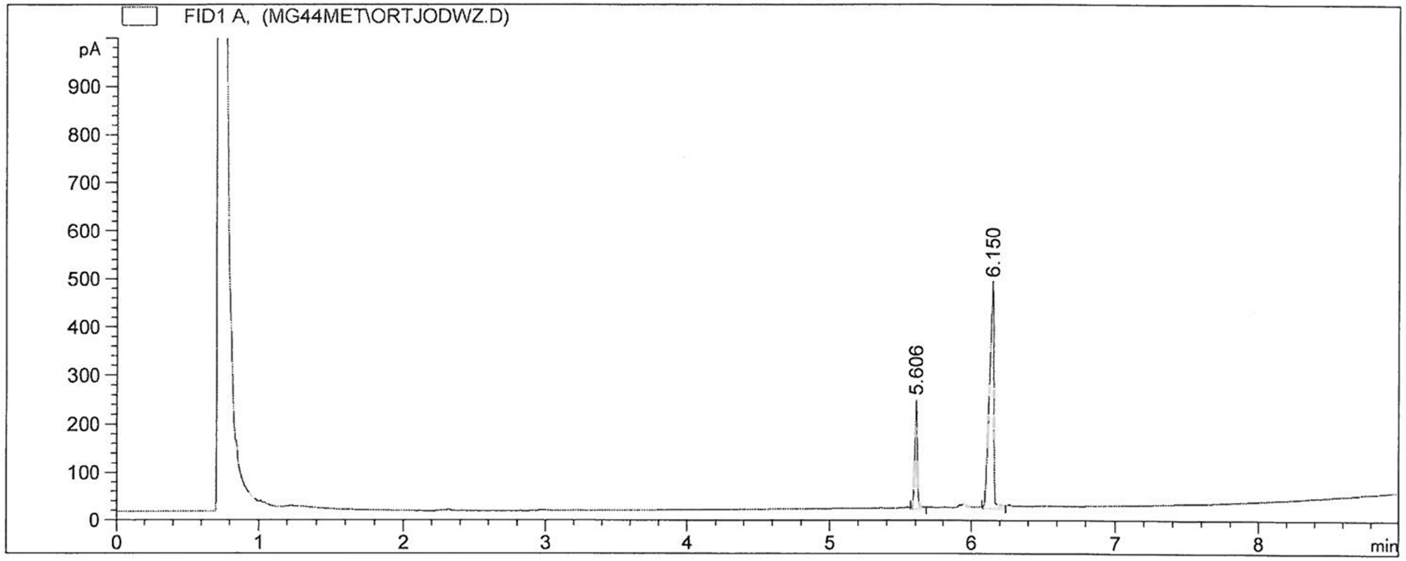

Peak #	RetTime [min]	Type	Width [min]	Area [pA*s]	Height [pA]	Area %
1	5.606	VV	0.0207	305.67554	224.27513	22.59300
2	6.150	VV	0.0325	1047.29004	468.84921	77.40700

Figure S10. Chromatogram of iodolactone **6b** (mixture of isomers A (minor) and B (major))

GC program: injector 250 ºC, detector (FID) 300 ºC, 160 ºC/1 min, ramp 15 ºC/min to 230 ºC, ramp 30 ºC/min to 300 ºC.

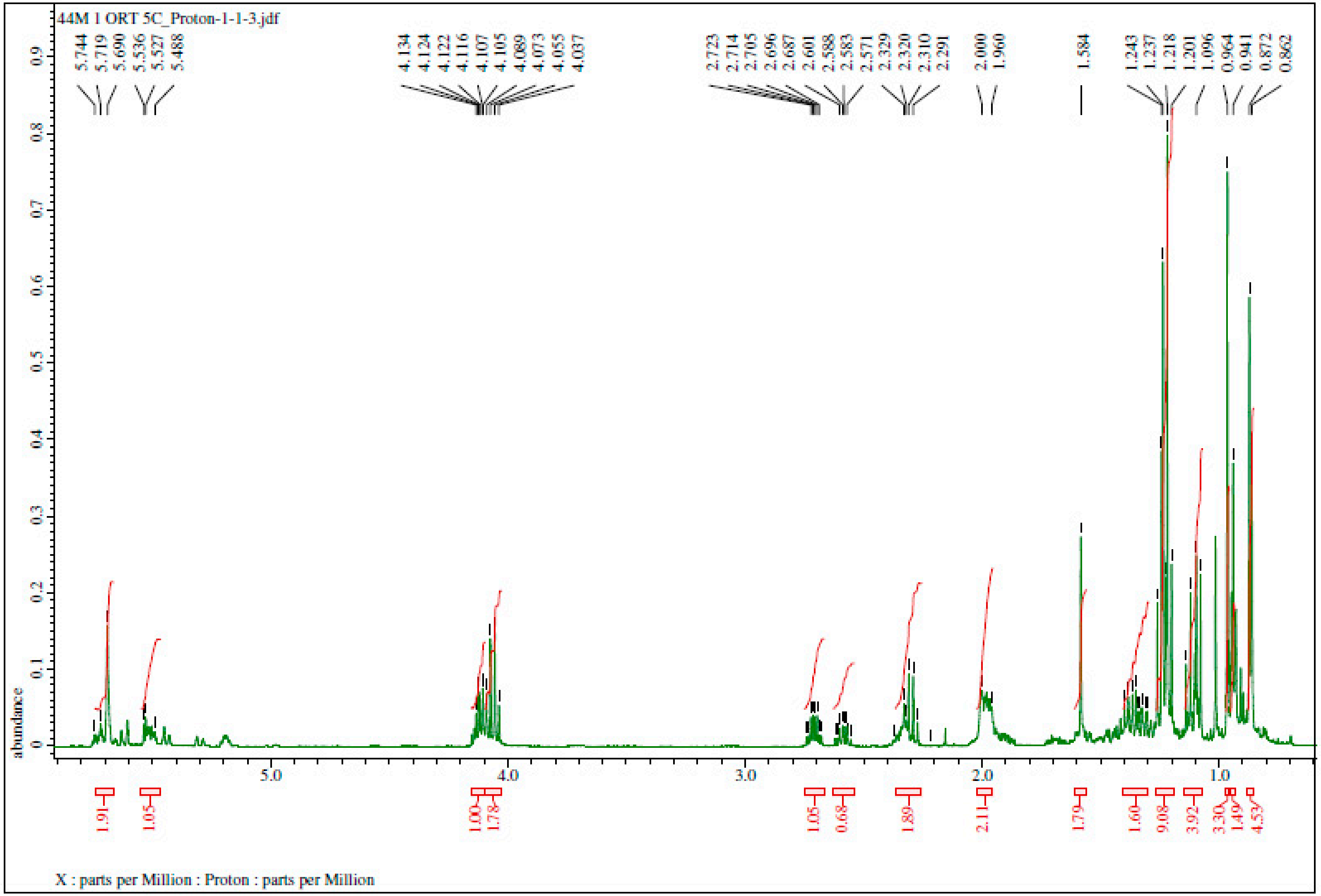

Figure S11. ^{1}H NMR (400 MHz, $CDCl_3$) spectrum of ester **2a** (mixture of isomers A and B)

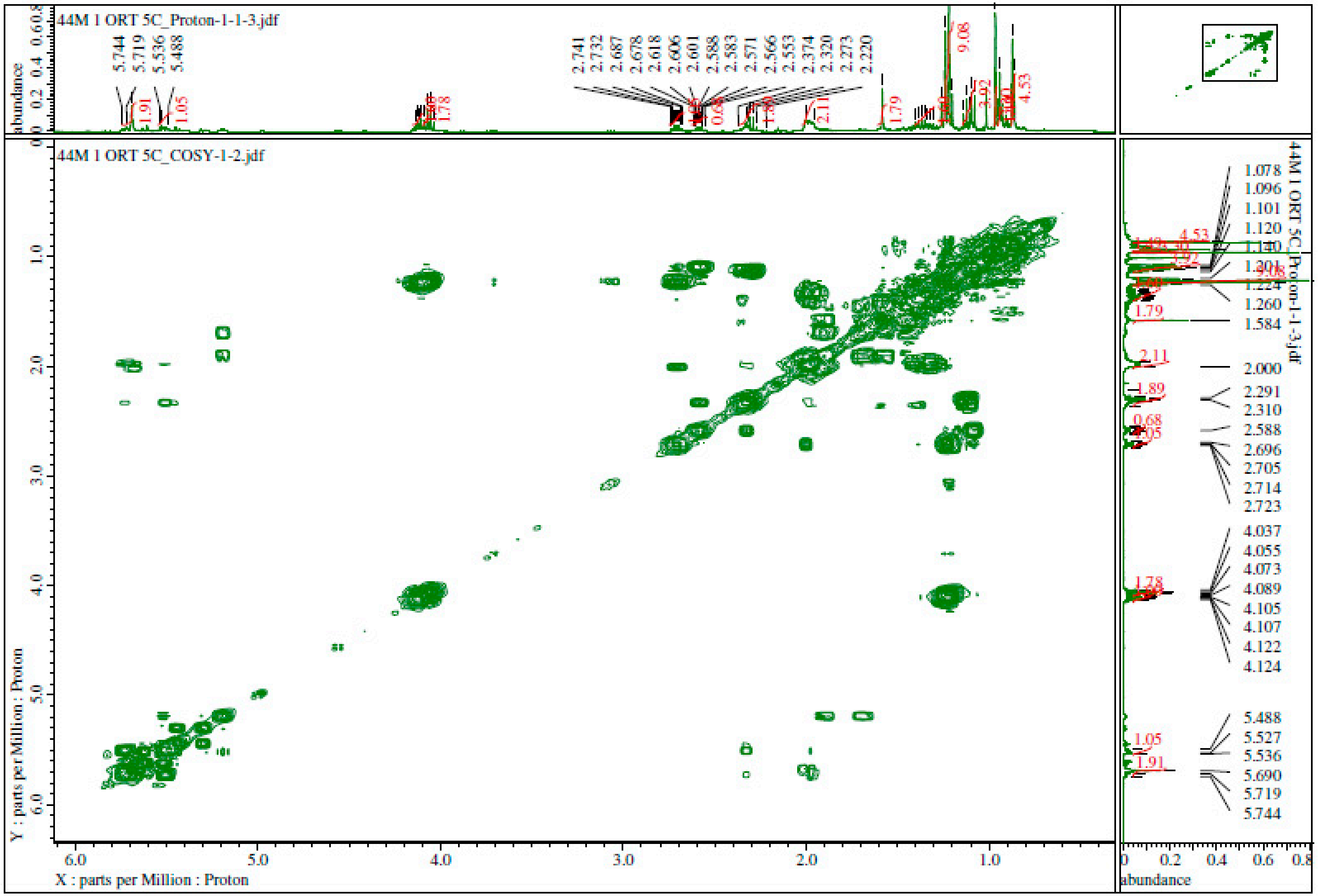

Figure S12. COSY (100 MHz, $CDCl_3$) spectrum of ester **2a** (mixture of isomers A and B)

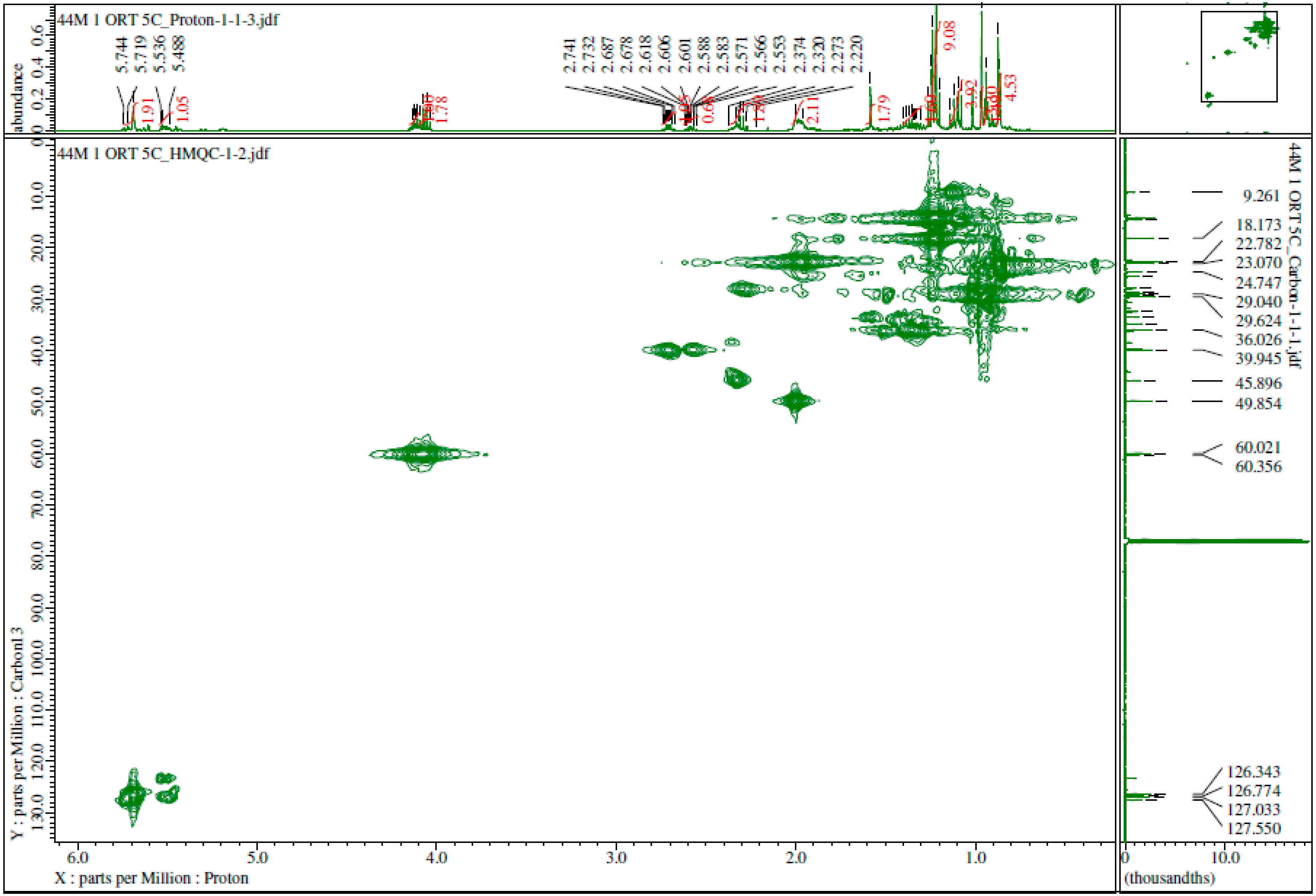

Figure S13. HMQC (100 MHz, $CDCl_3$) spectrum of ester **2a** (mixture of isomers A and B)

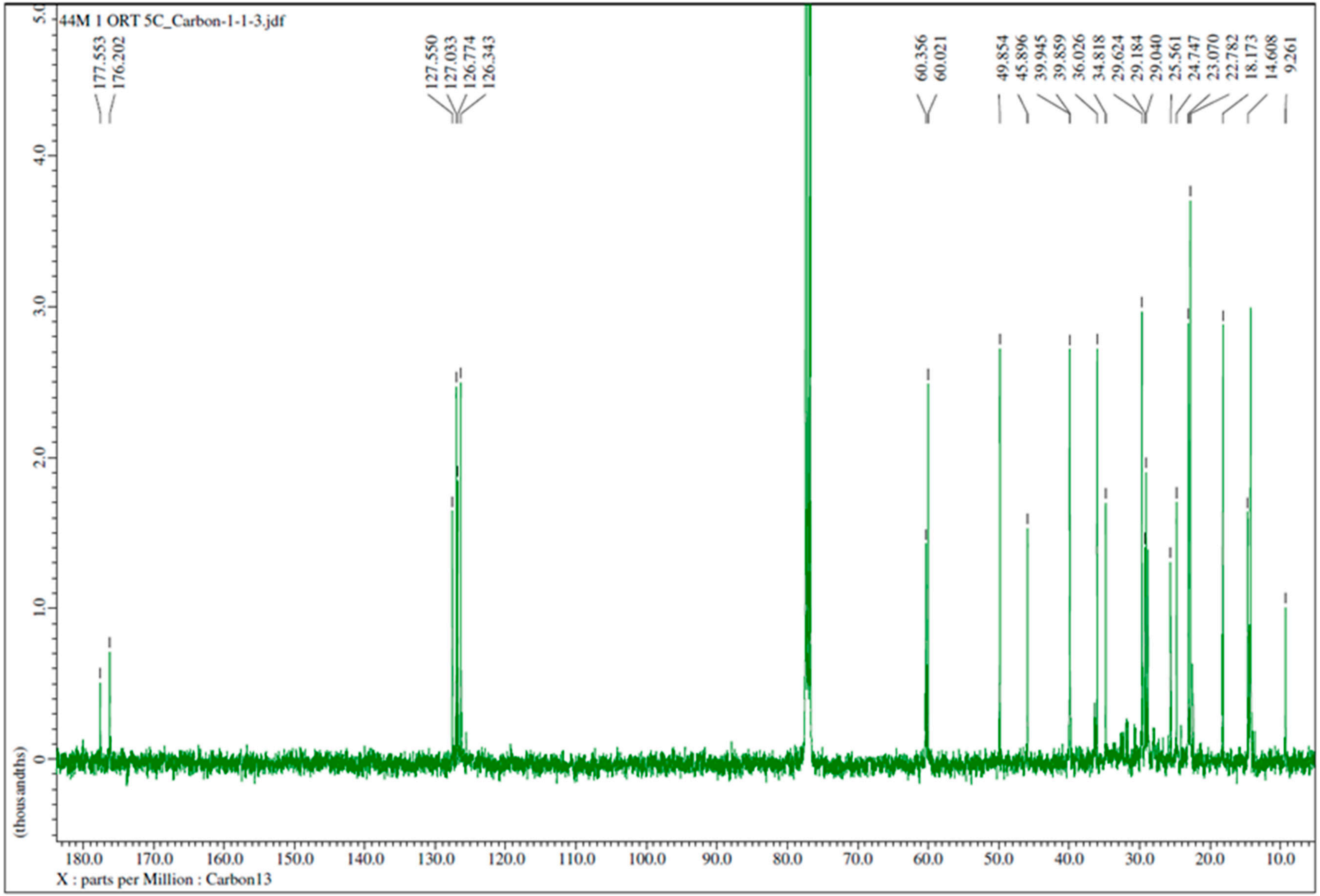

Figure S14. ^{13}C NMR (100 MHz, $CDCl_3$) spectrum of ester **2a** (mixture of isomers A and B)

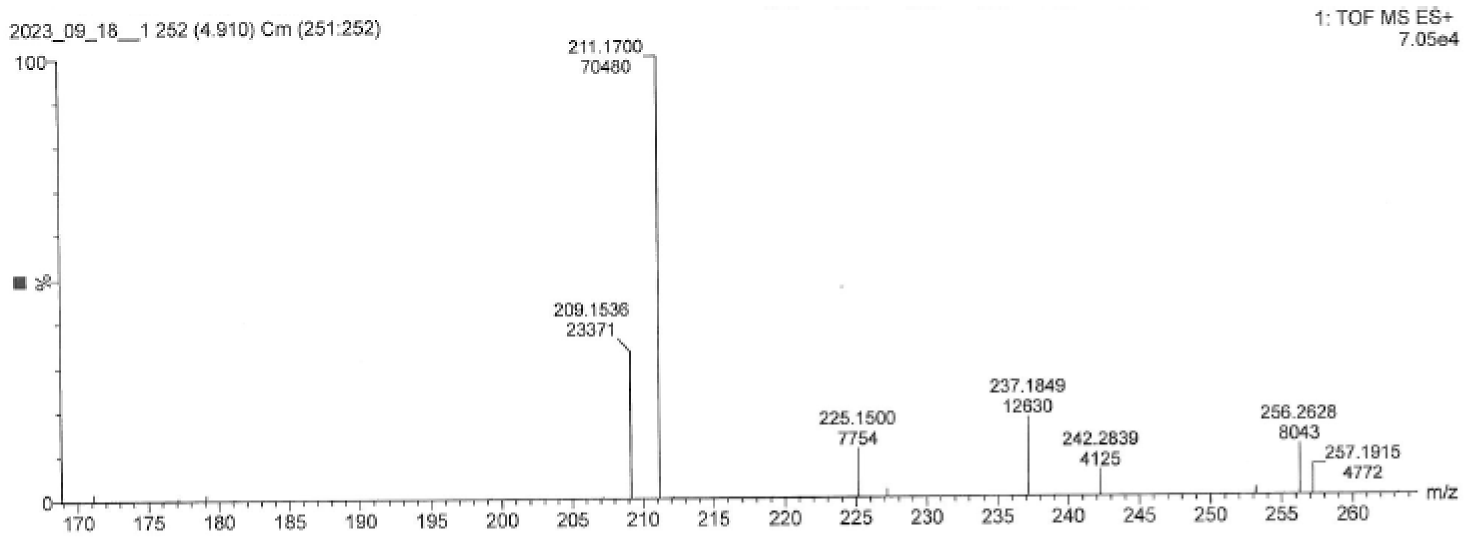

Figure S15. HRMS spectrum of ester **2a**

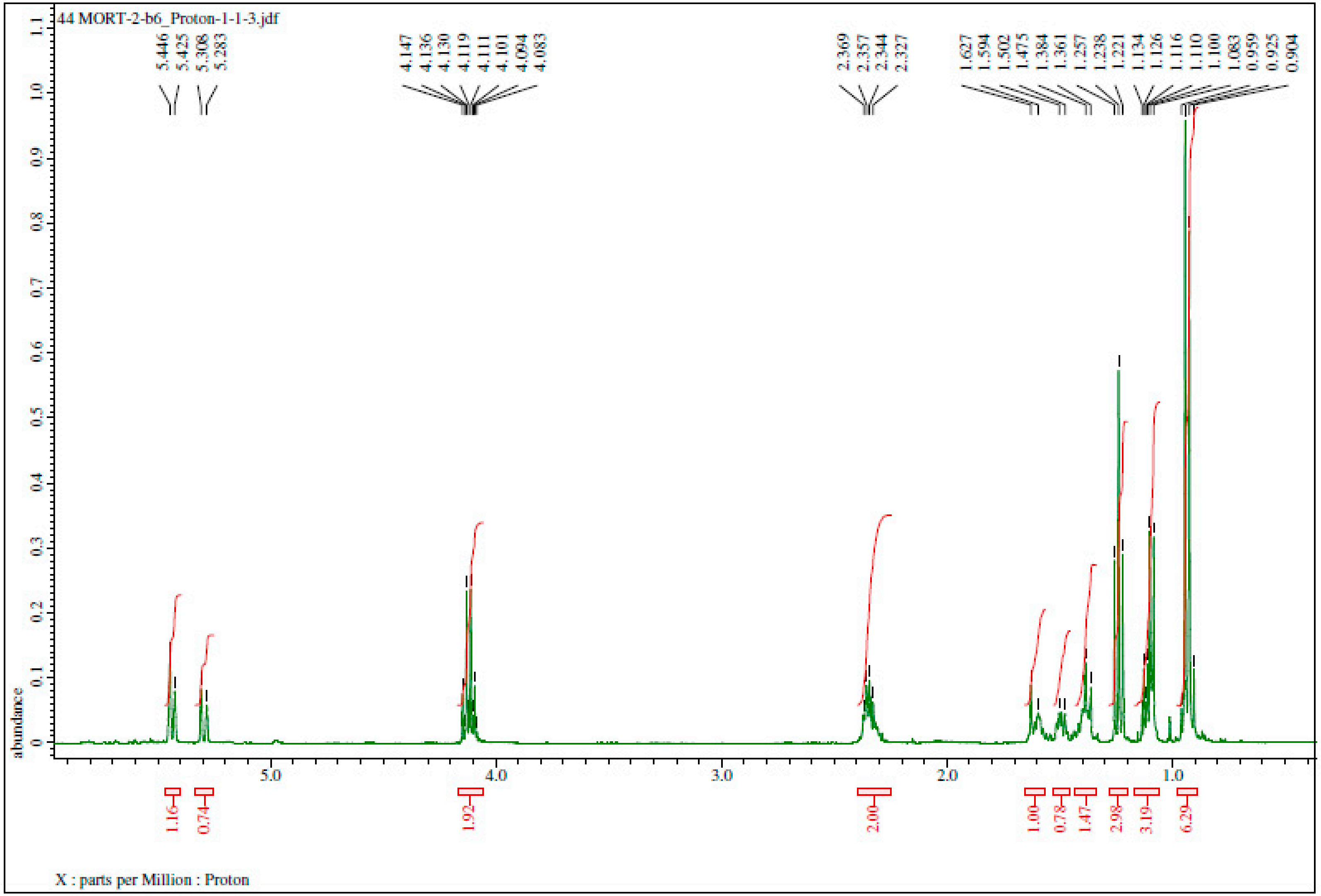

Figure S16. ^{1}H NMR (400 MHz, $CDCl_3$) spectrum of ester **2b** (mixture of isomers A and B)

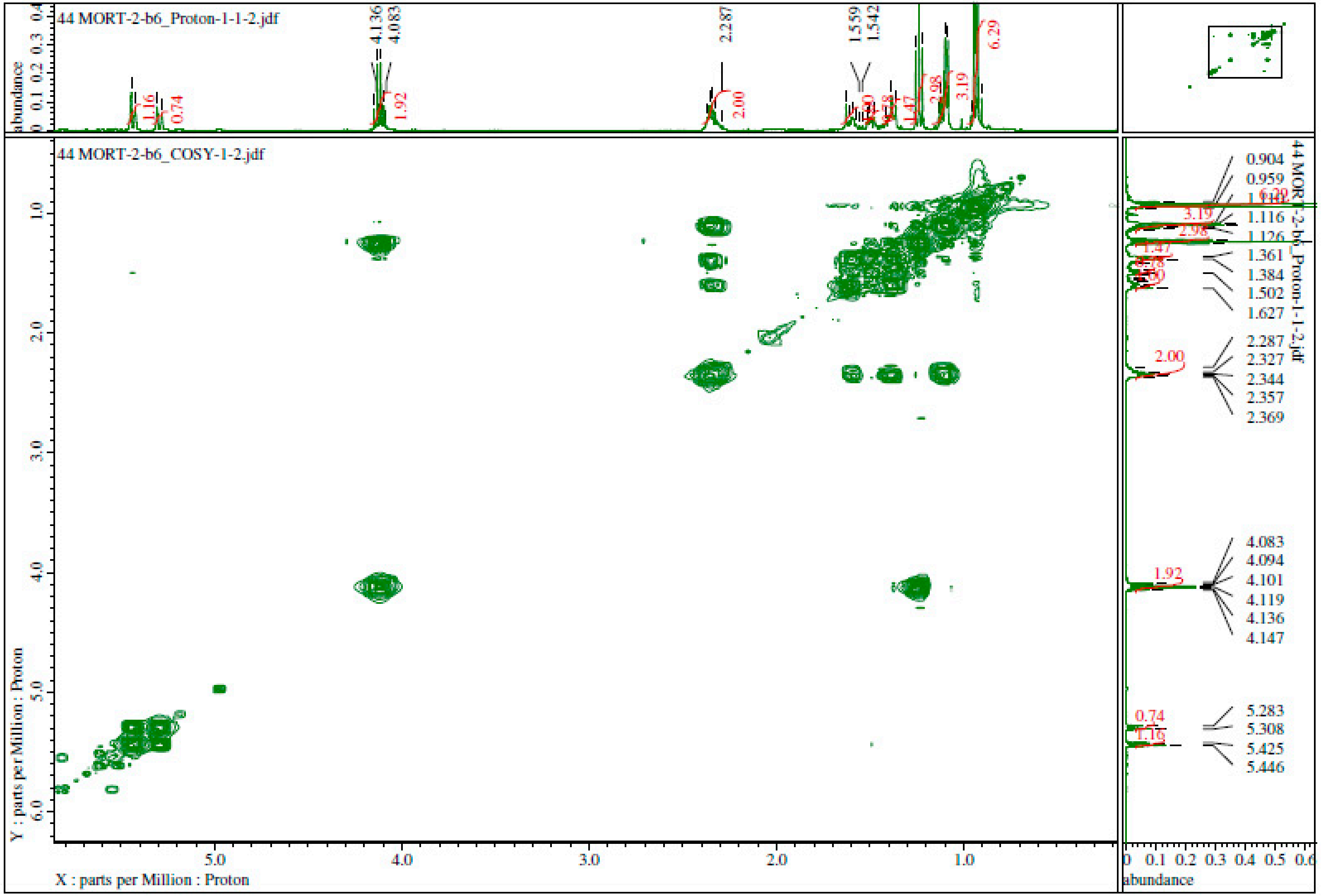

Figure S17. COSY (100 MHz, $CDCl_3$) spectrum of ester **2b** (mixture of isomers A and B)

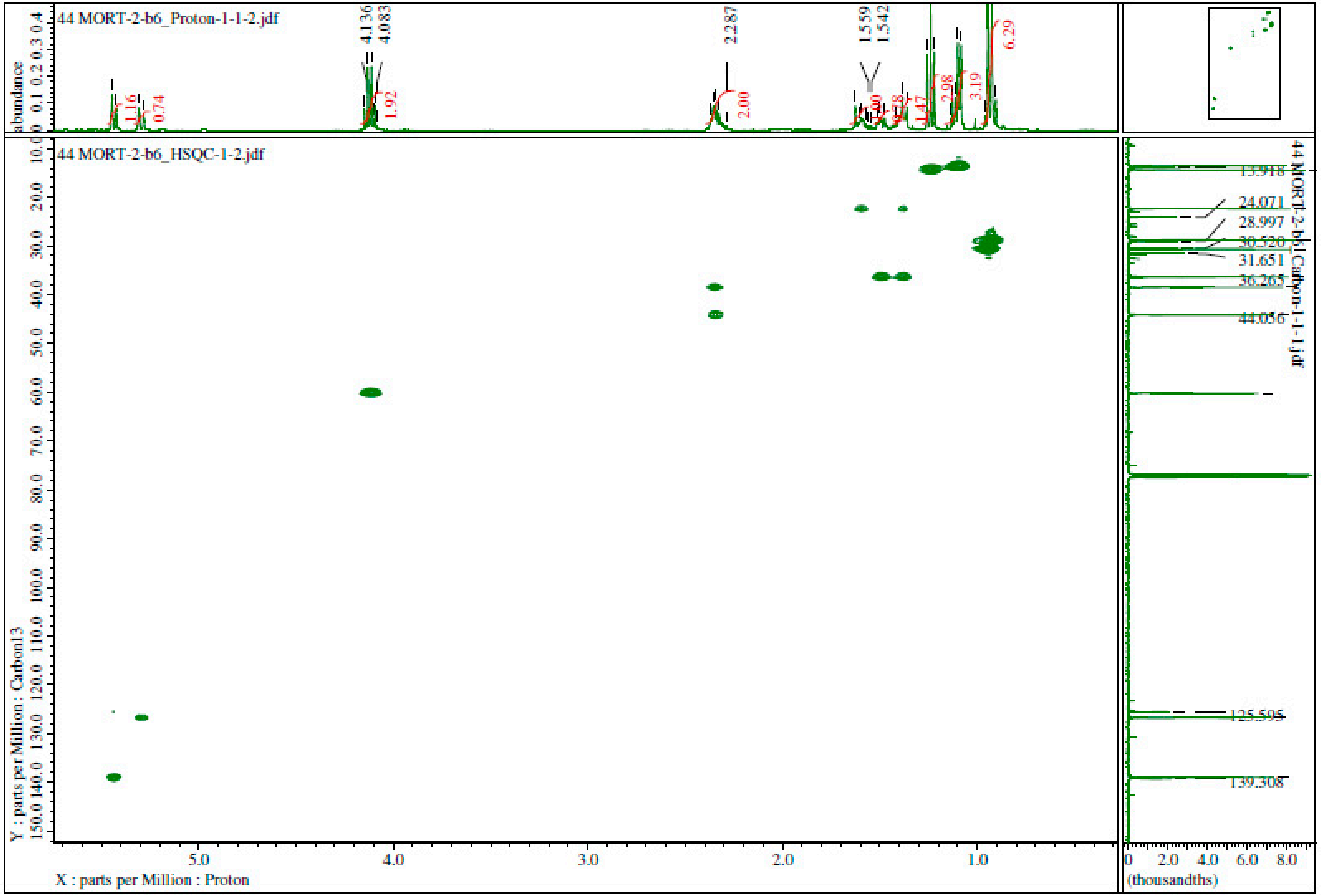

Figure S18. HMQC (100 MHz, $CDCl_3$) spectrum of ester **2b** (mixture of isomers A and B)

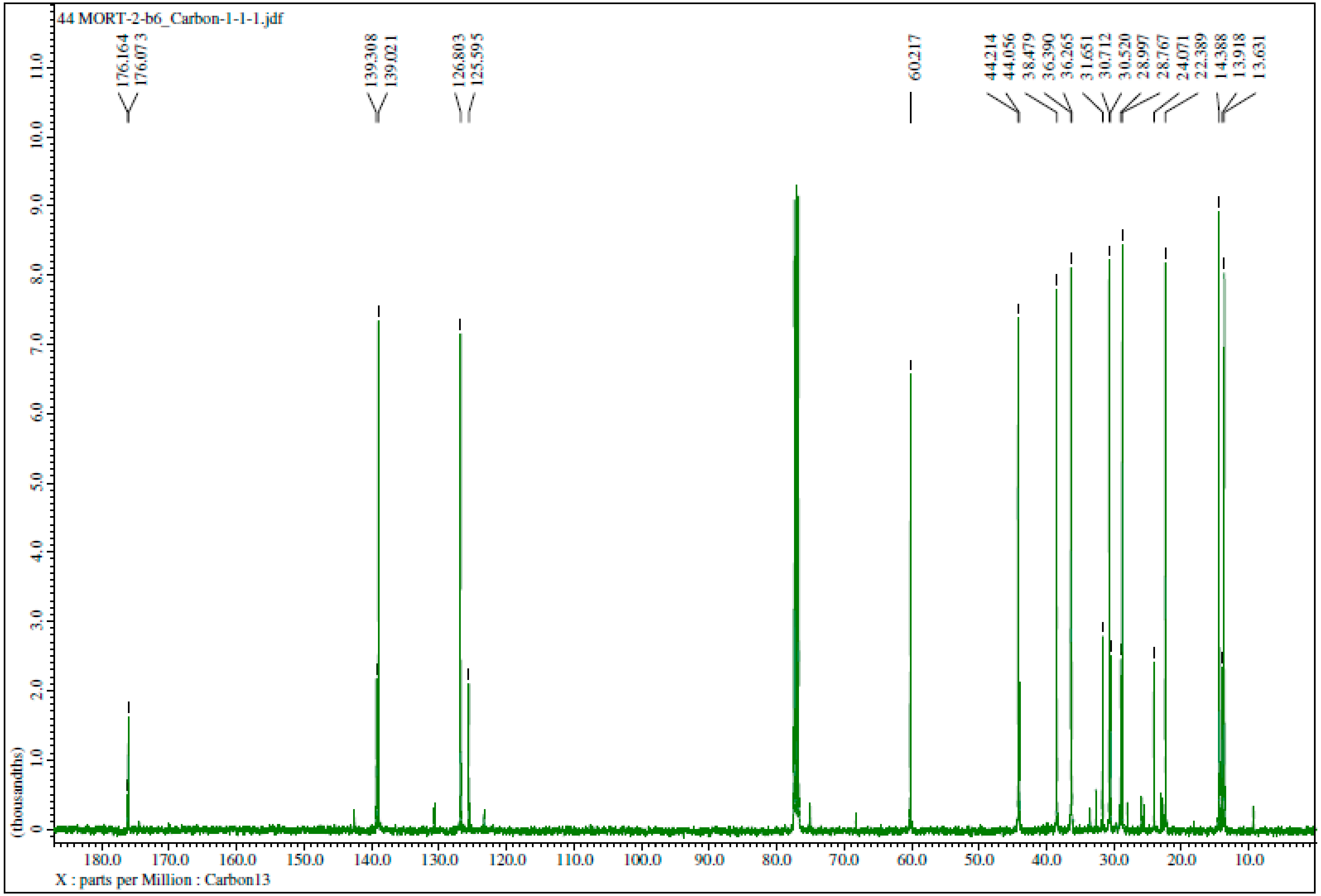

Figure S19. ^{13}C NMR (100 MHz, $CDCl_3$) spectrum of ester **2b** (mixture of isomers A and B)

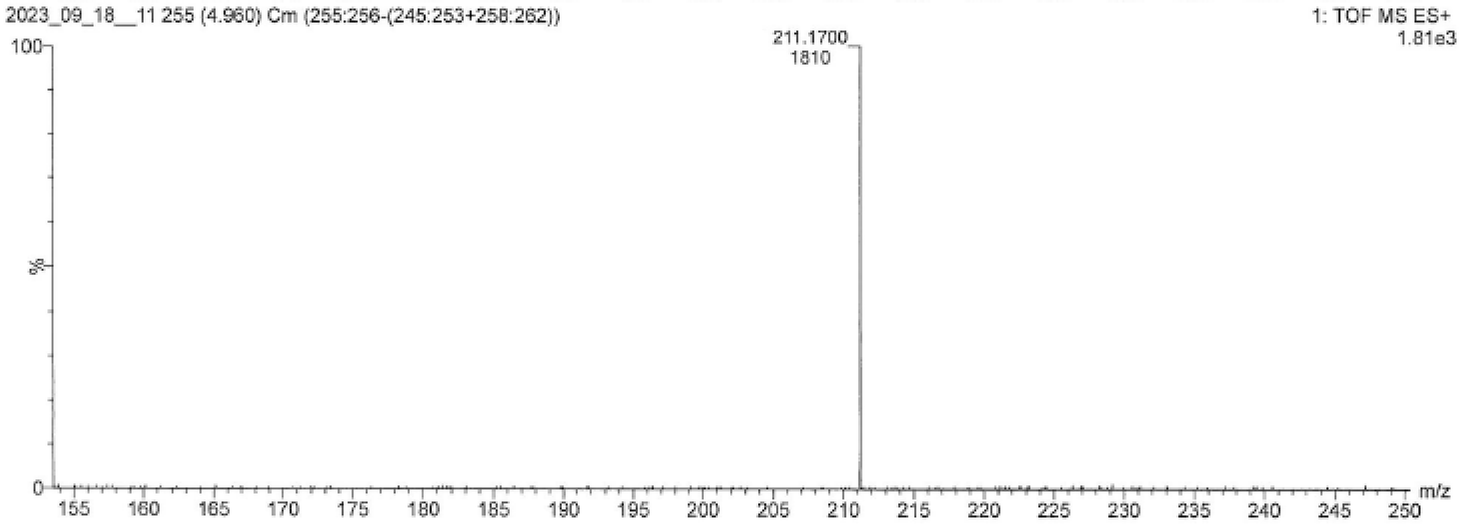

Figure S20. HRMS spectrum of ester **2b**

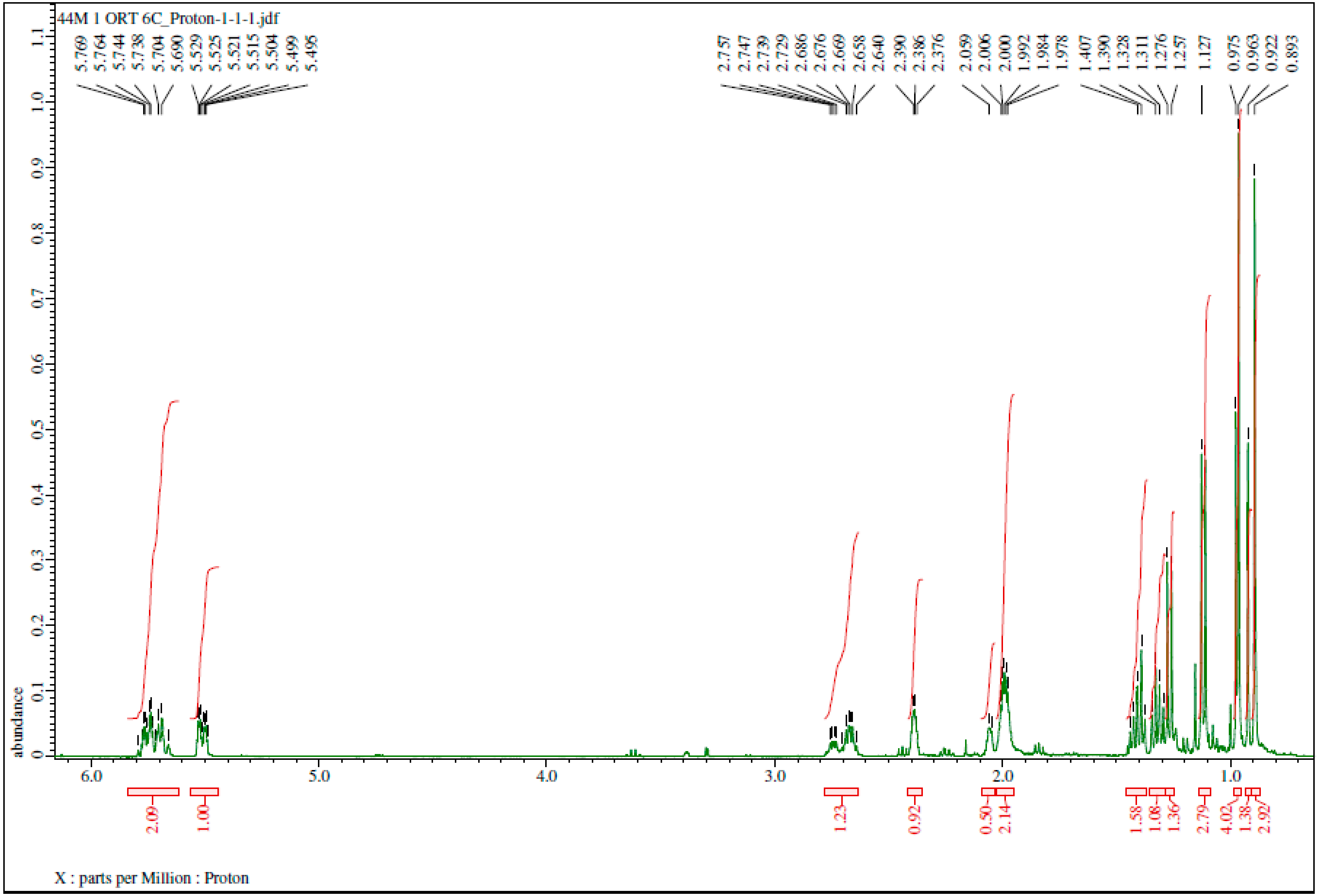

Figure S21. ^{1}H NMR (400 MHz, $CDCl_3$) spectrum of acid **3a** (mixture of isomers A and B)

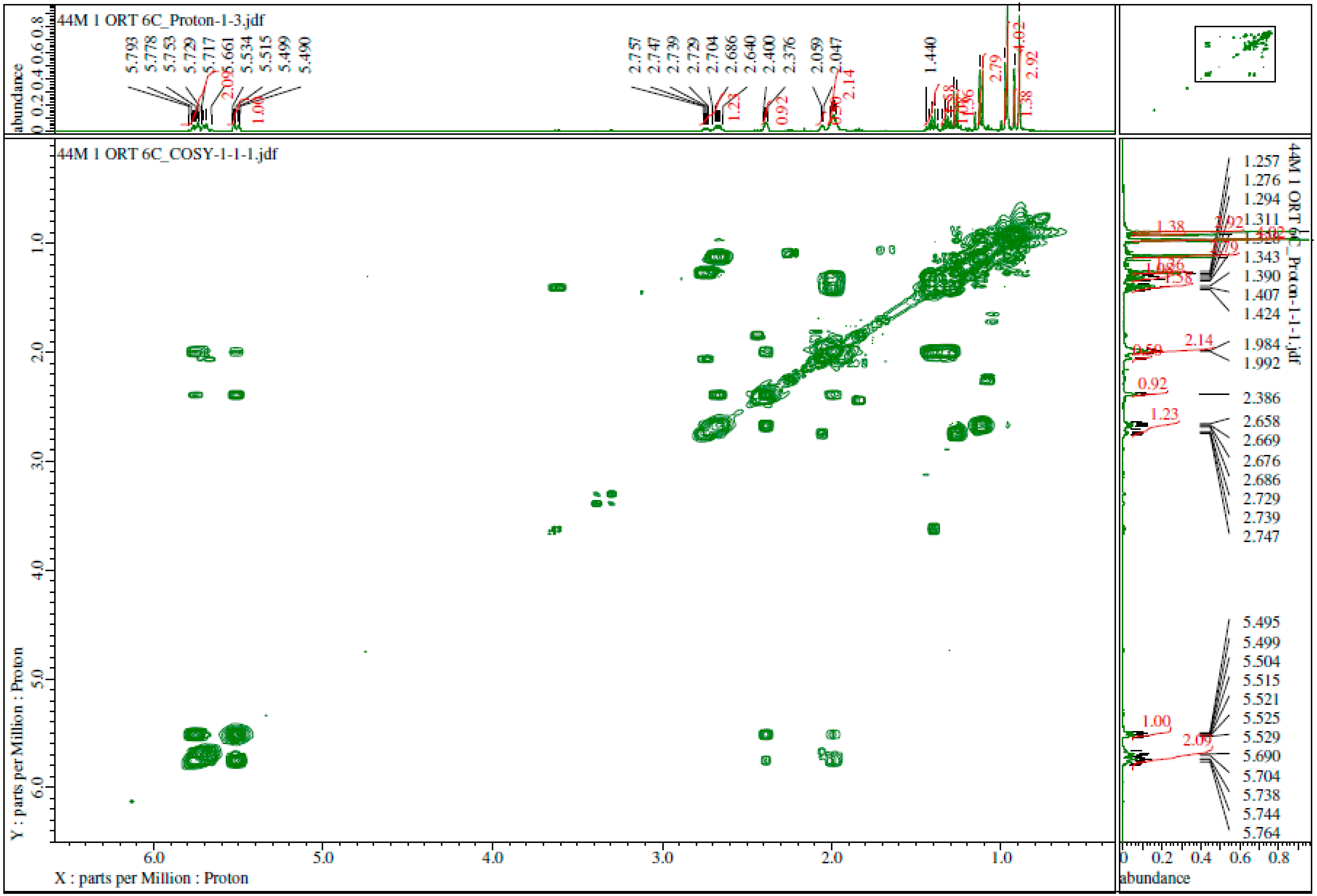

Figure S22. COSY (100 MHz, $CDCl_3$) spectrum of acid **3a** (mixture of isomers A and B)

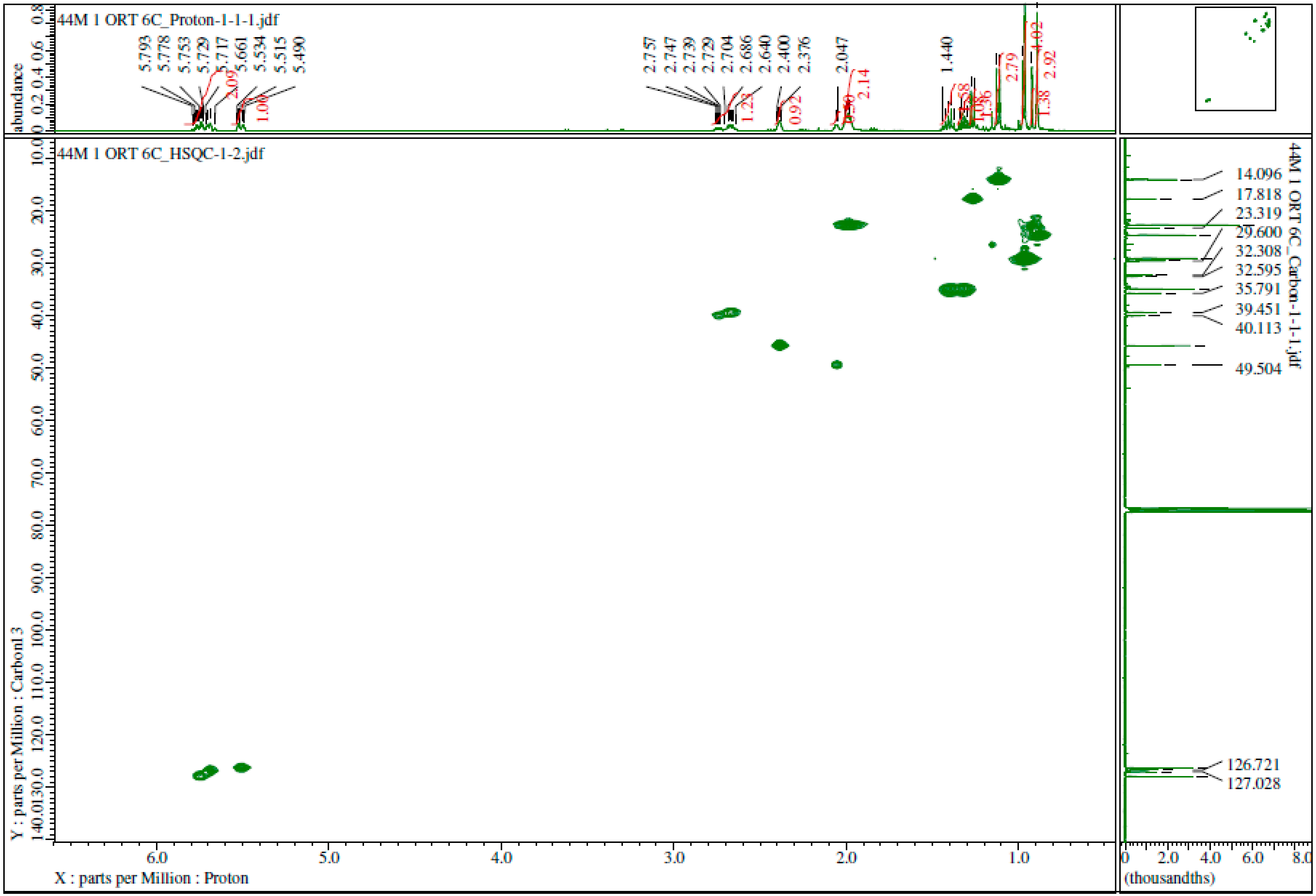

Figure S23. HMQC (100 MHz, $CDCl_3$) spectrum of acid **3a** (mixture of isomers A and B)

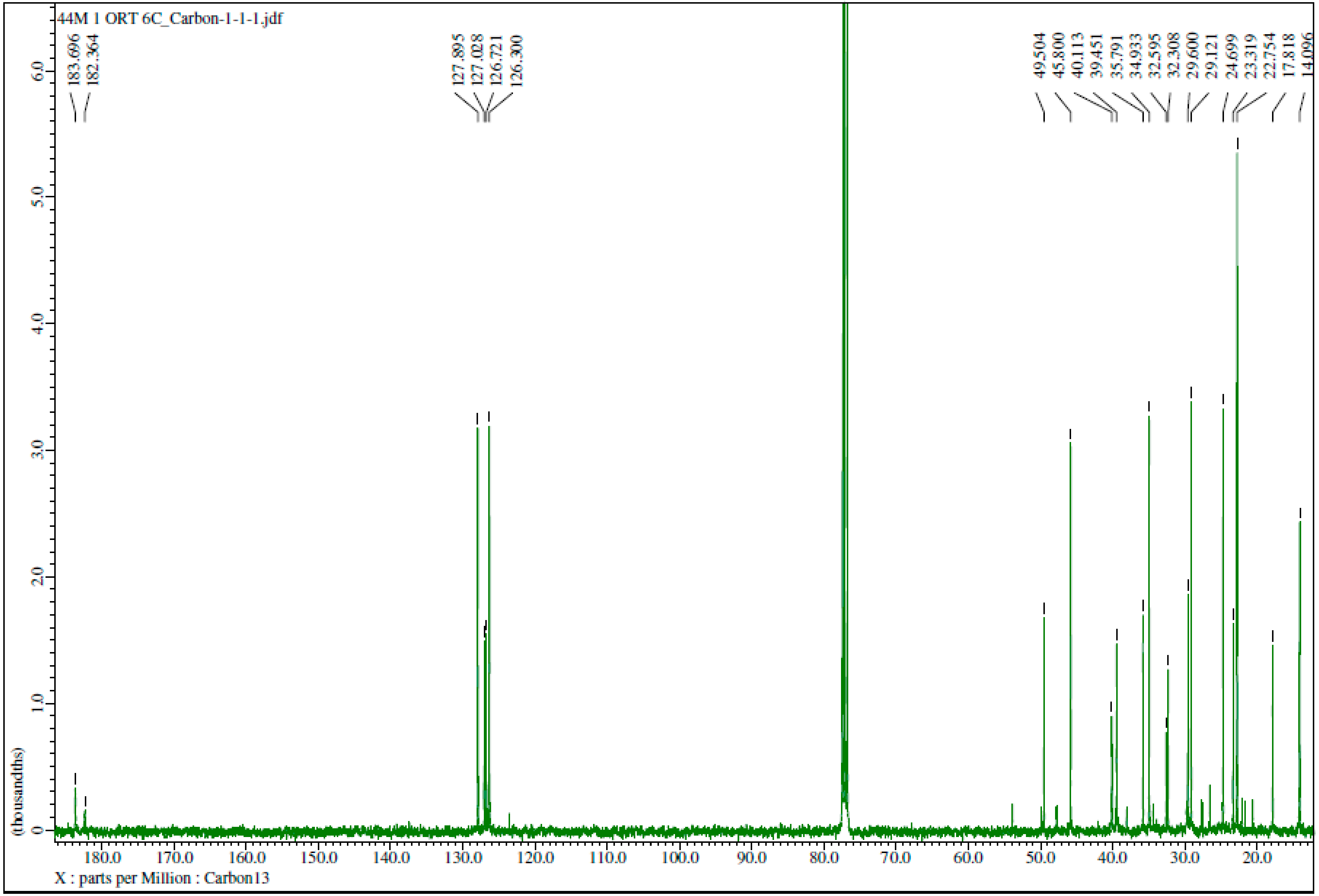

Figure S24. ^{13}C NMR (100 MHz, $CDCl_3$) spectrum of acid **3a** (mixture of isomers A and B)

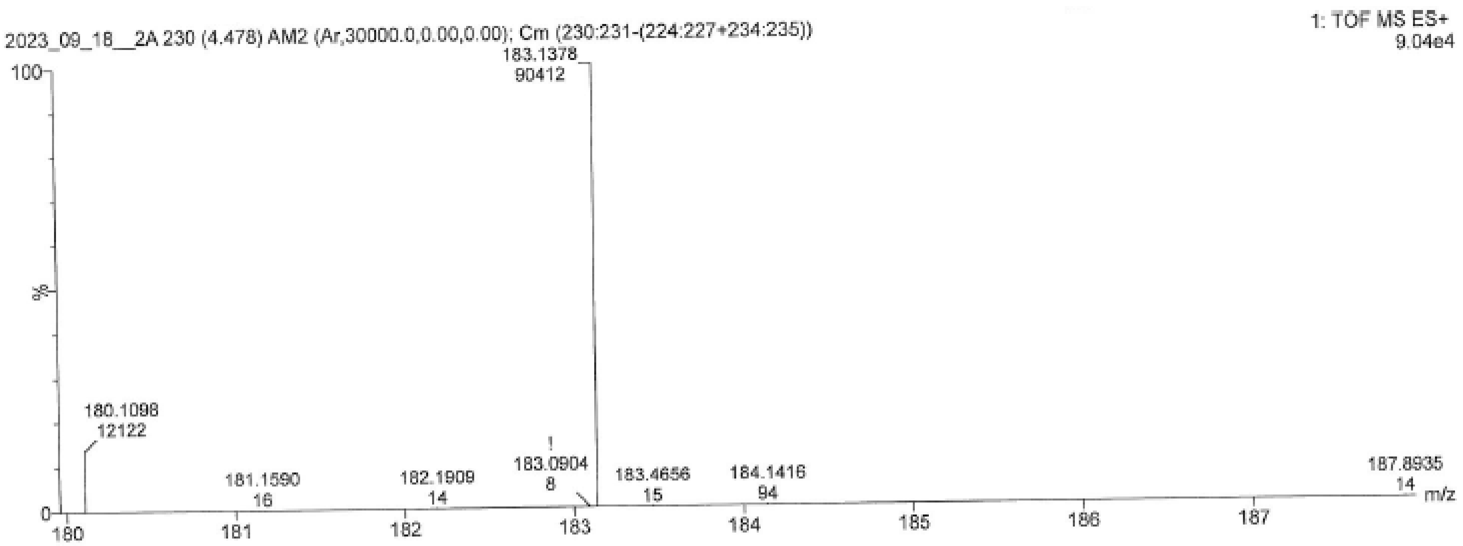

Figure S25. HRMS spectrum of acid **3a**

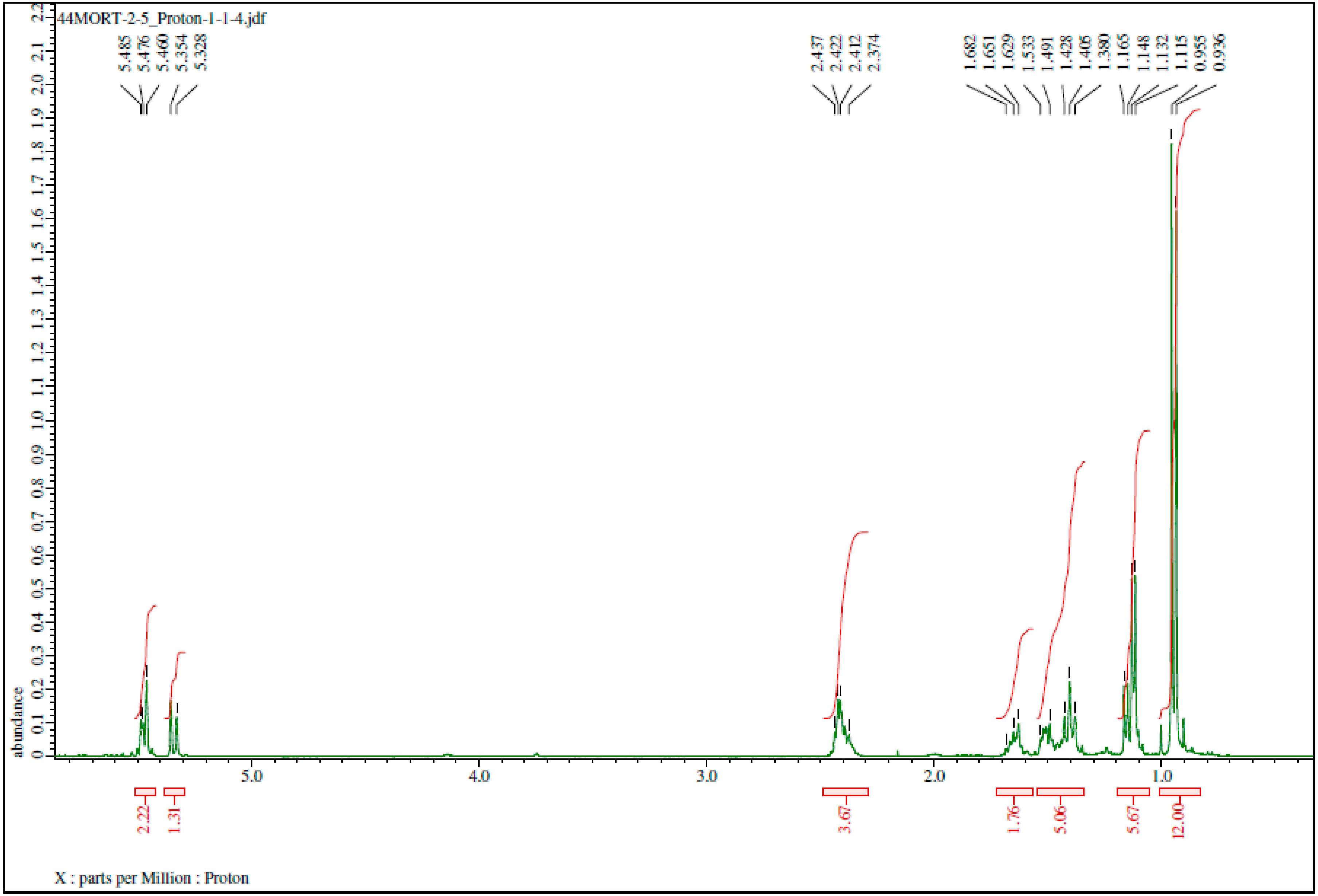

Figure S26. ^{1}H NMR (400 MHz, $CDCl_3$) spectrum of acid **3b** (mixture of isomers A and B)

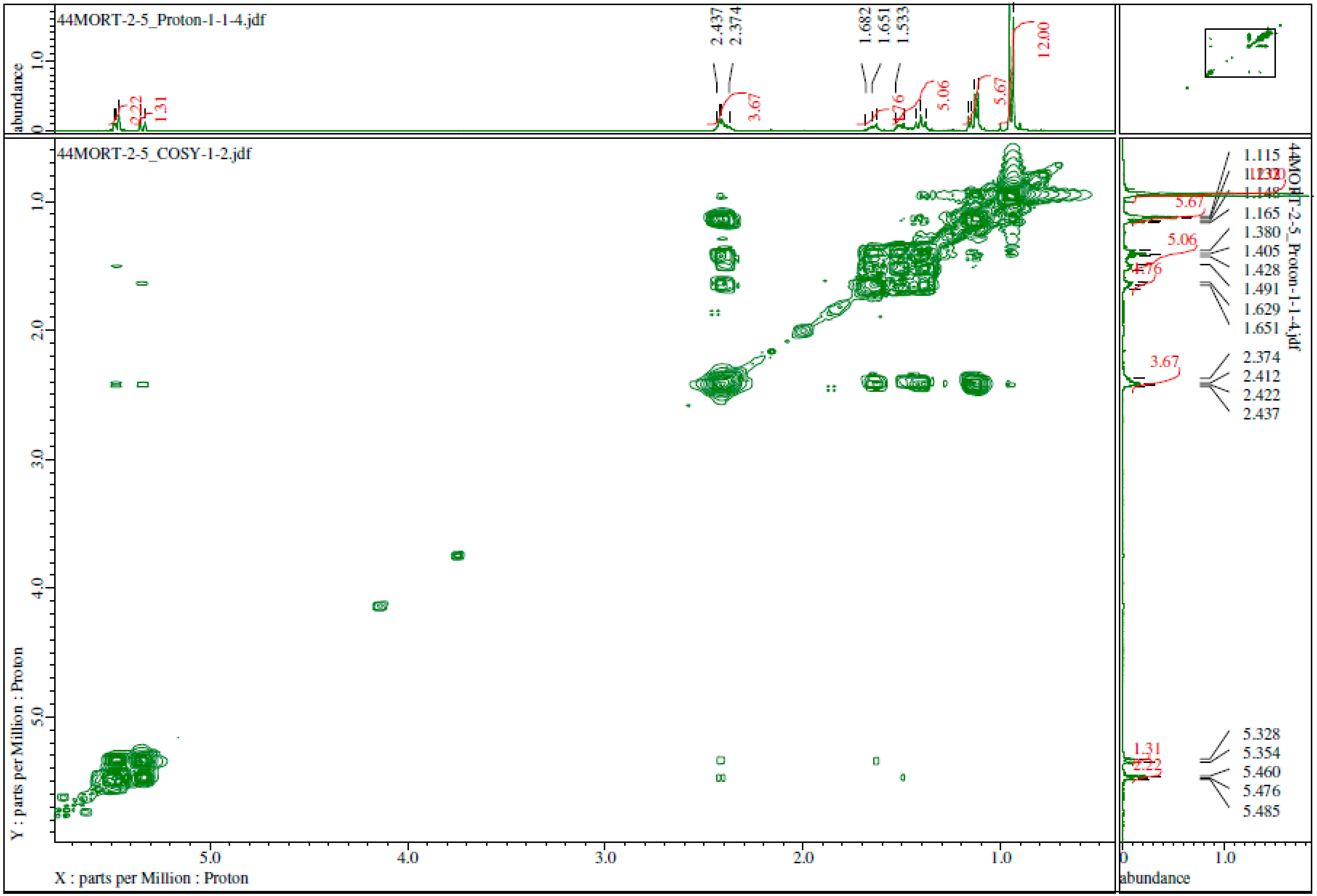

Figure S27. COSY (100 MHz, $CDCl_3$) spectrum of acid **3b** (mixture of isomers A and B)

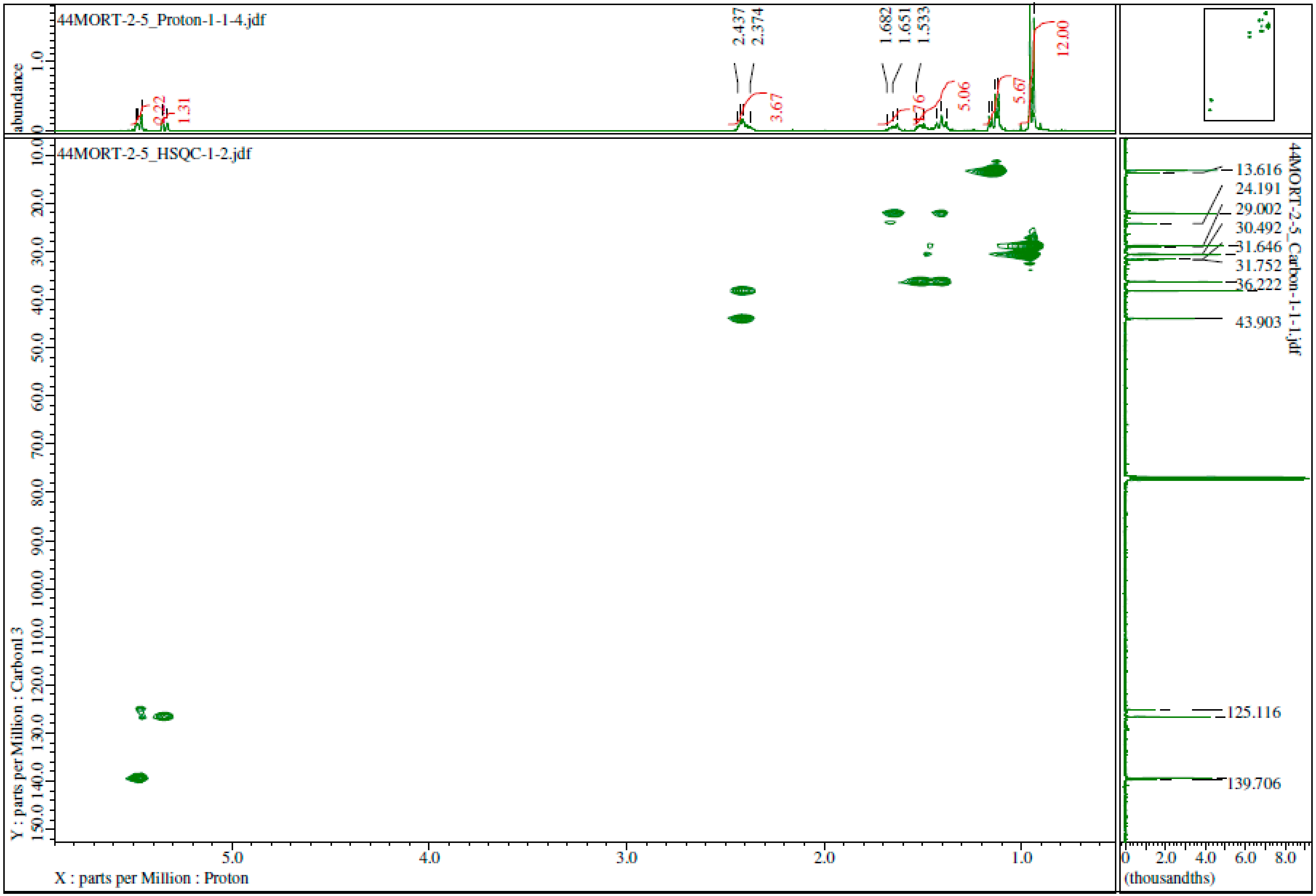

Figure S28. HMQC (100 MHz, $CDCl_3$) spectrum of acid **3b** (mixture of isomers A and B)

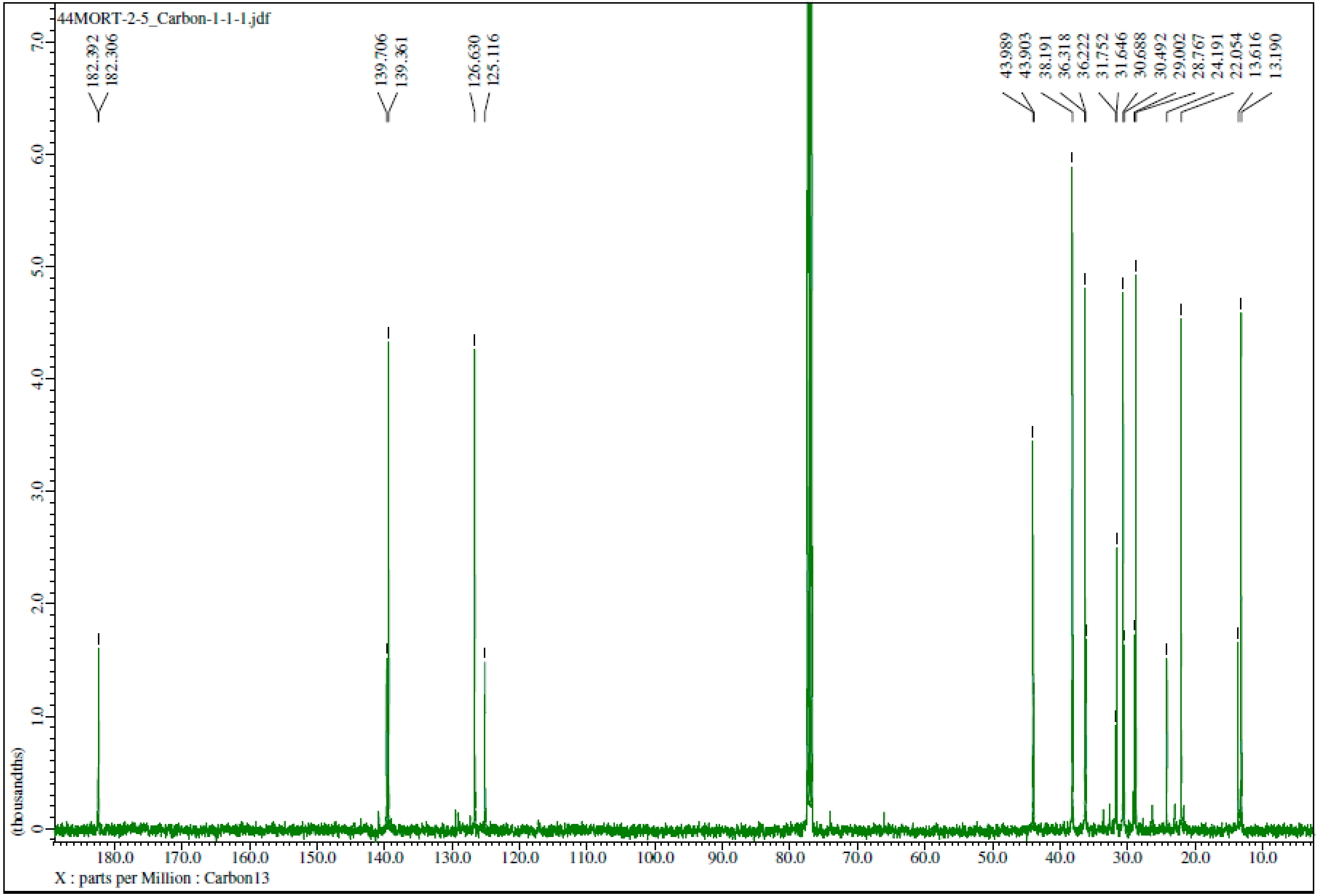

Figure S29. ^{13}C NMR (100 MHz, $CDCl_3$) spectrum of acid **3b** (mixture of isomers A and B)

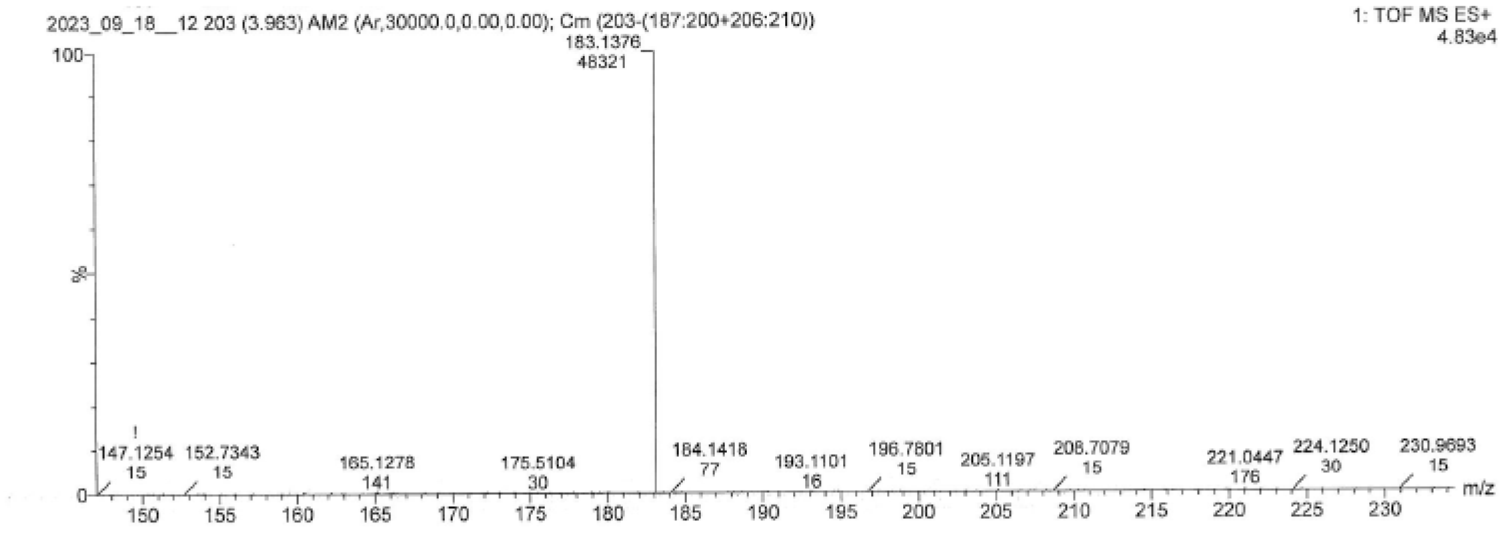

Figure S30. HRMS spectrum of acid **3b** (mixture of isomers A and B)

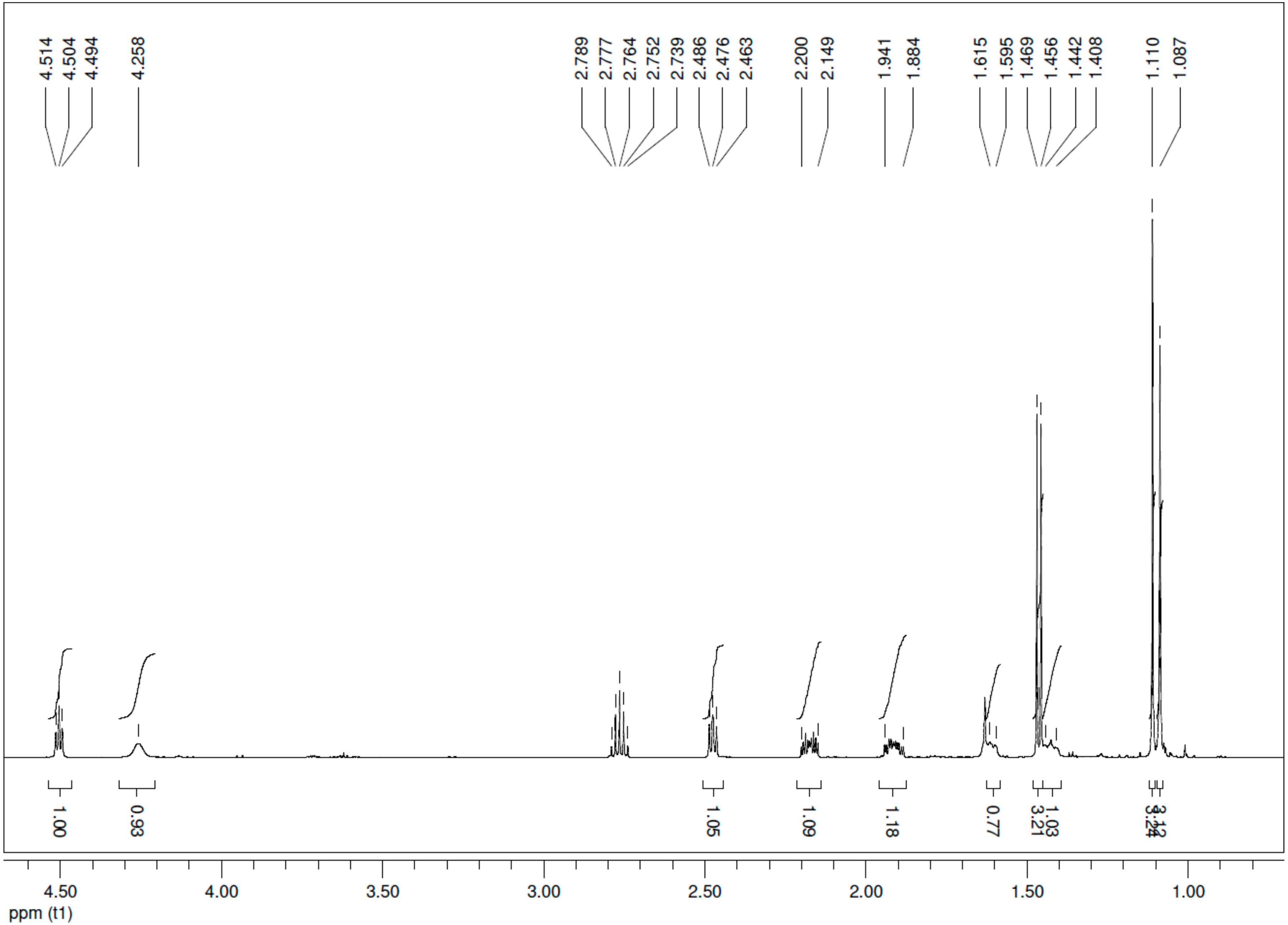

Figure S31. ^{1}H NMR (600 MHz, $CDCl_3$) spectrum of chlorolactone **4a-A**

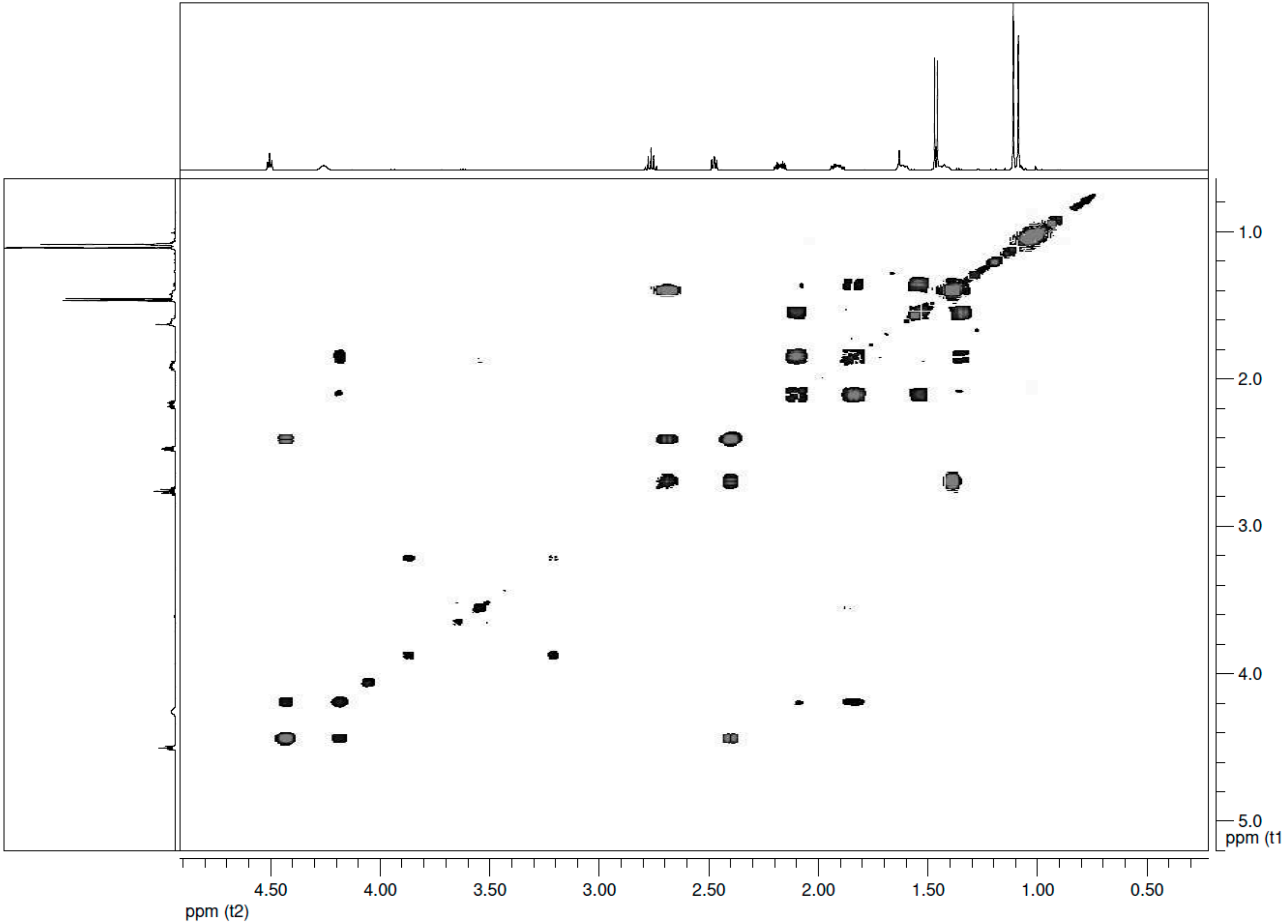

Figure S32. COSY (151 MHz, $CDCl_3$) spectrum of chlorolactone **4a-A**

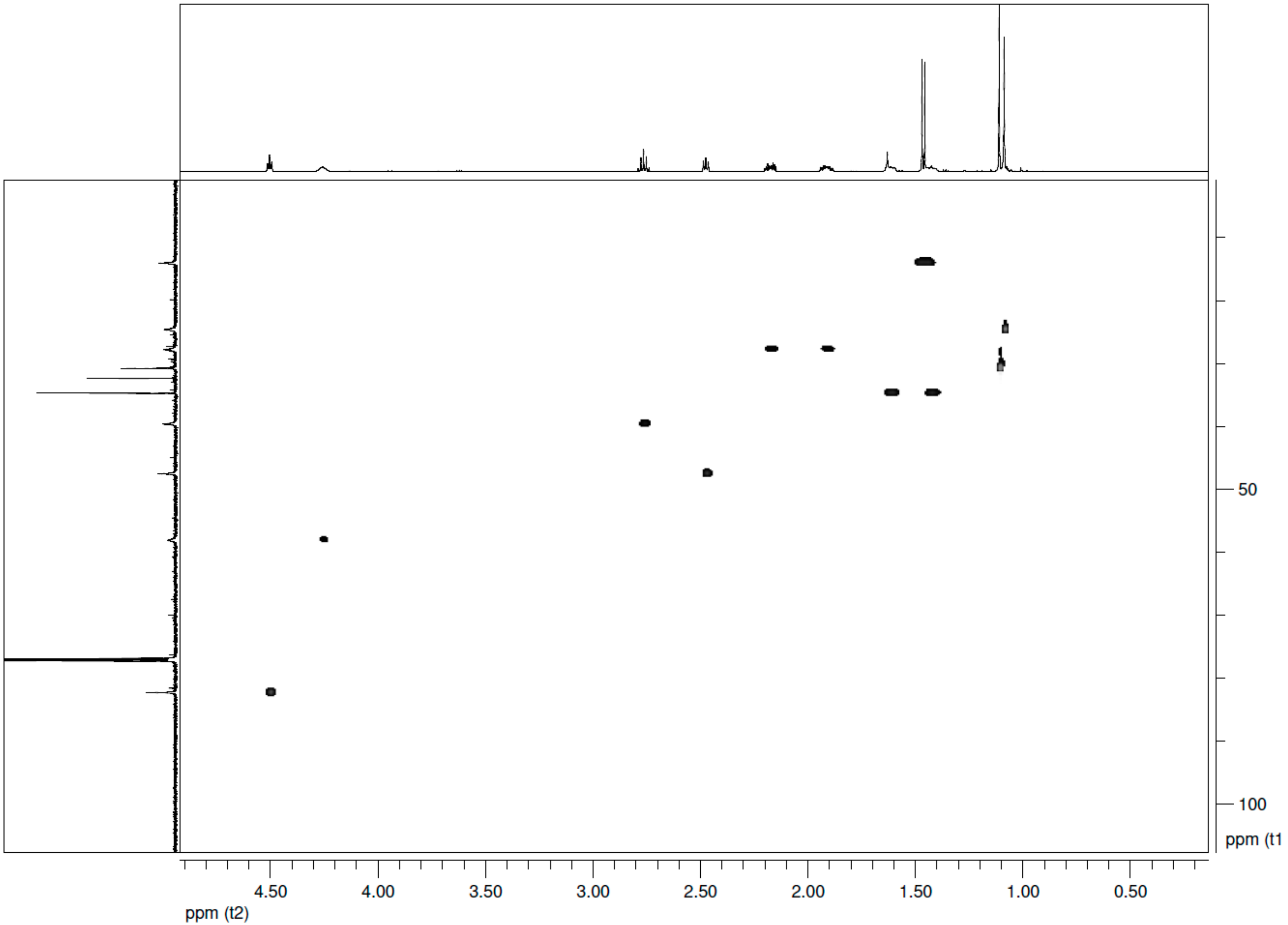

Figure S33. HMQC (151 MHz, $CDCl_3$) spectrum of chlorolactone **4a-A**

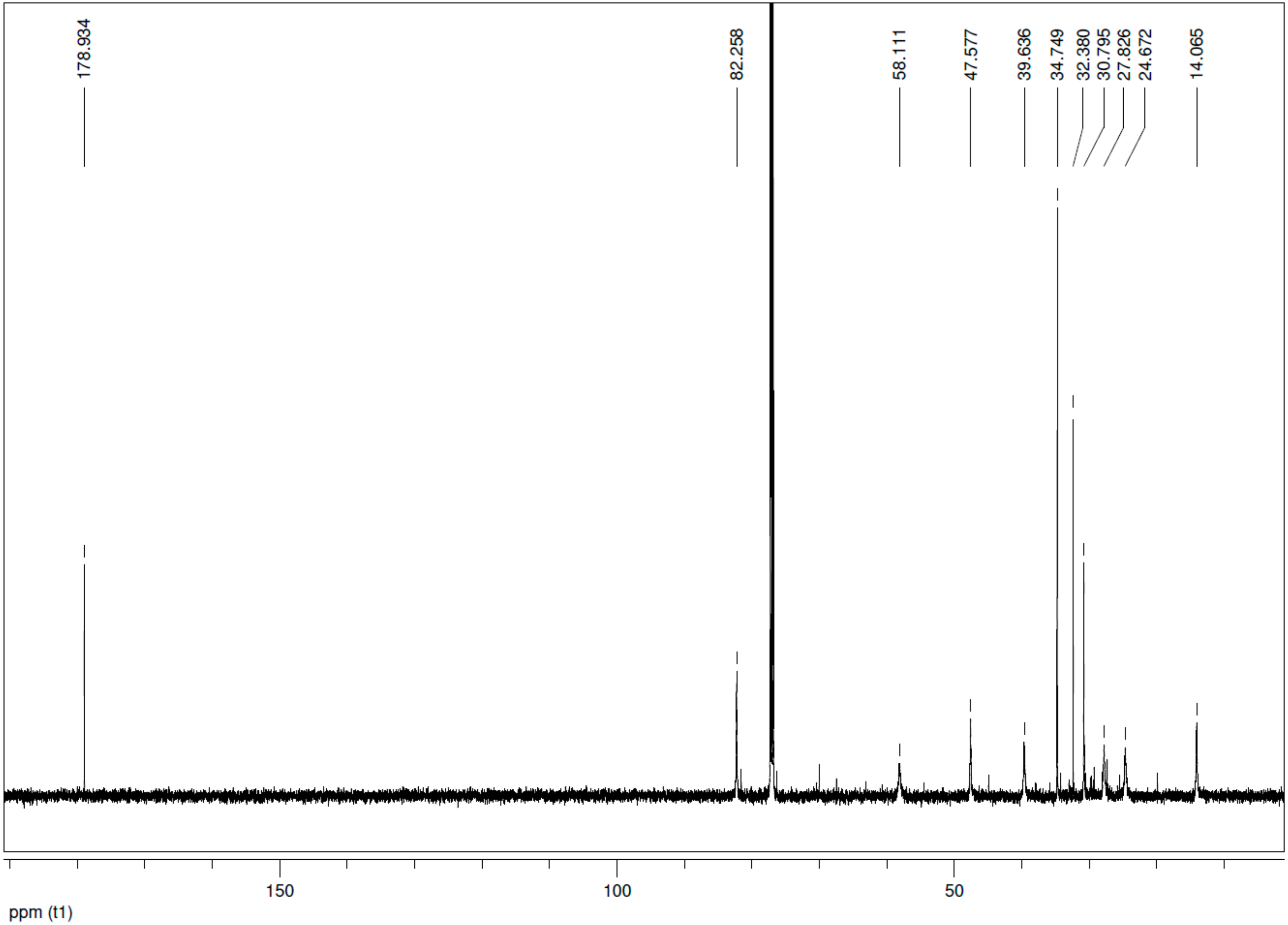

Figure S34. ^{13}C NMR (151 MHz, $CDCl_3$) spectrum of chlorolactone **4a-A**

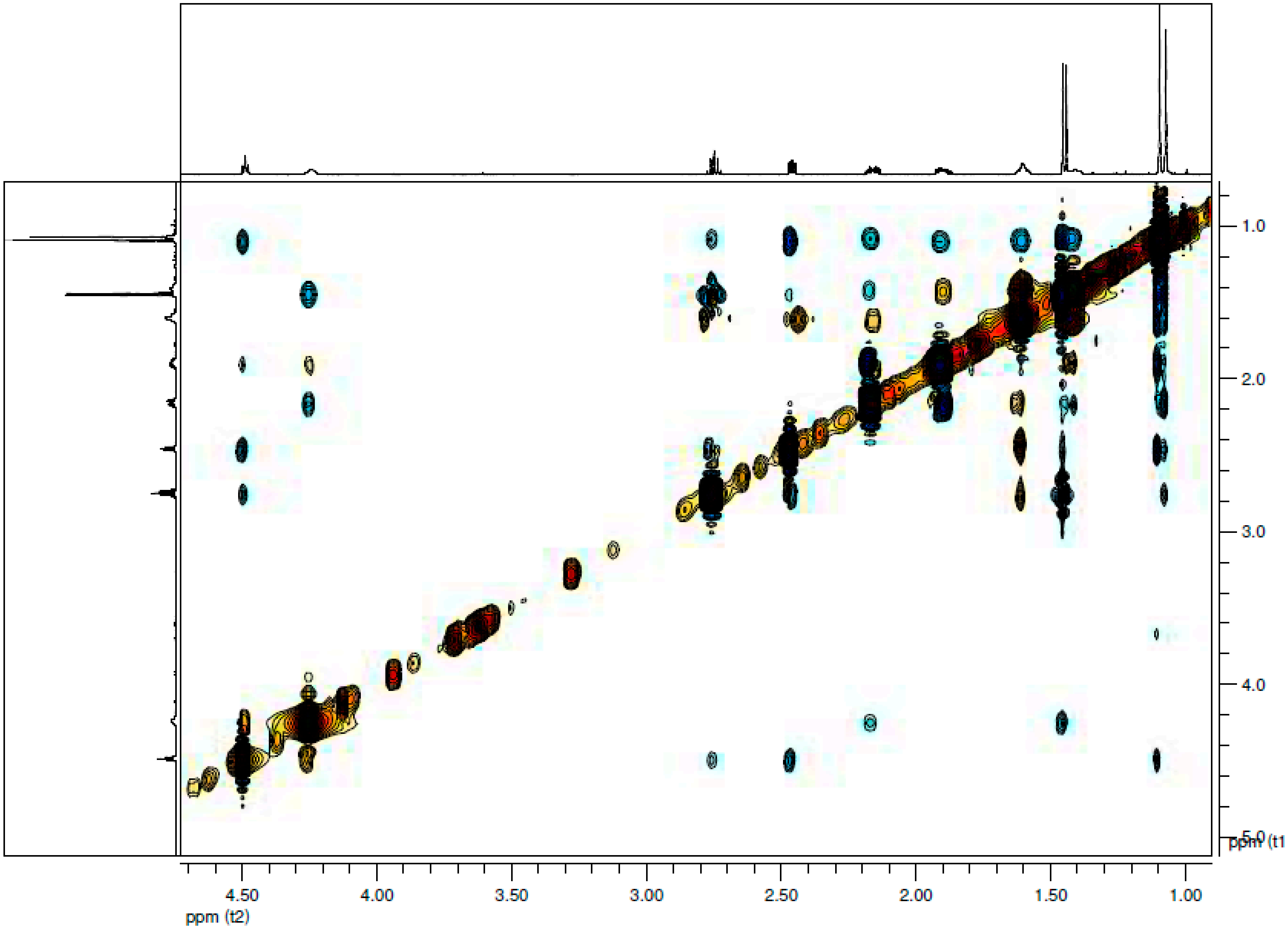

Figure S35.NOESY NMR (151 MHz, $CDCl_3$) spectrum of chlorolactone **4a-A**

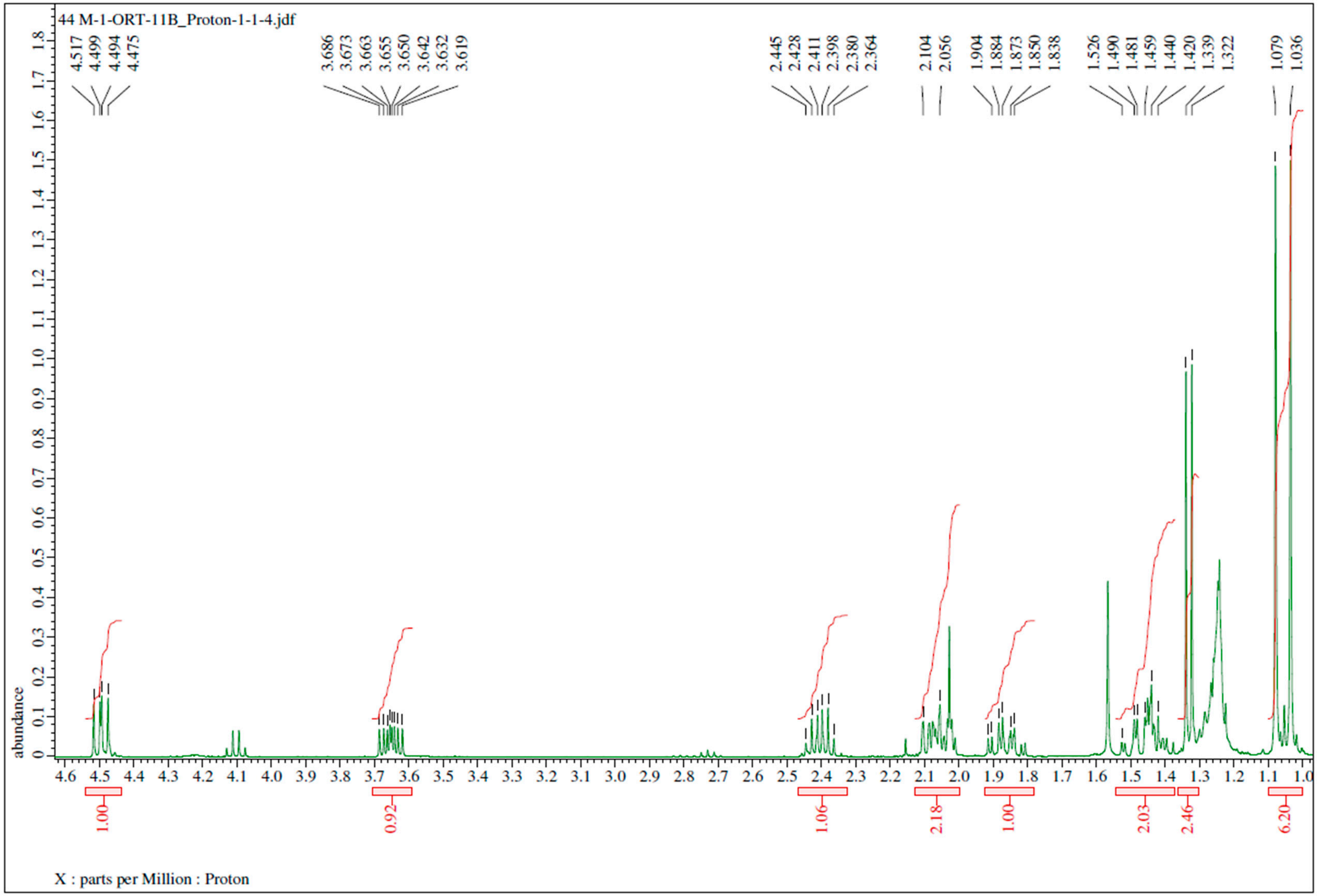

Figure S36. ^{1}H NMR (400 MHz, $CDCl_3$) spectrum of chlorolactone **4a-B**

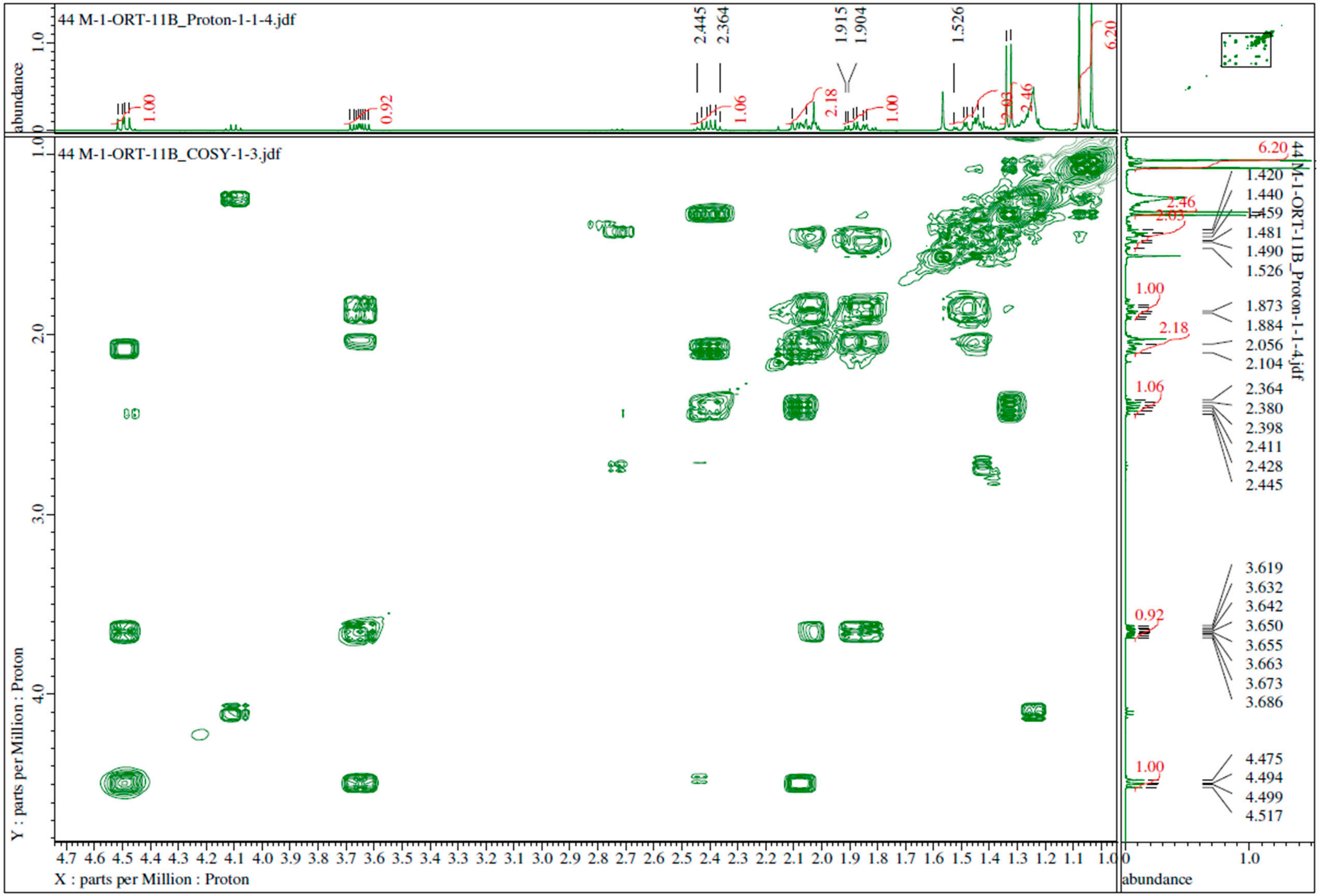

Figure S37. COSY (100 MHz, $CDCl_3$) spectrum of chlorolactone **4a-B**

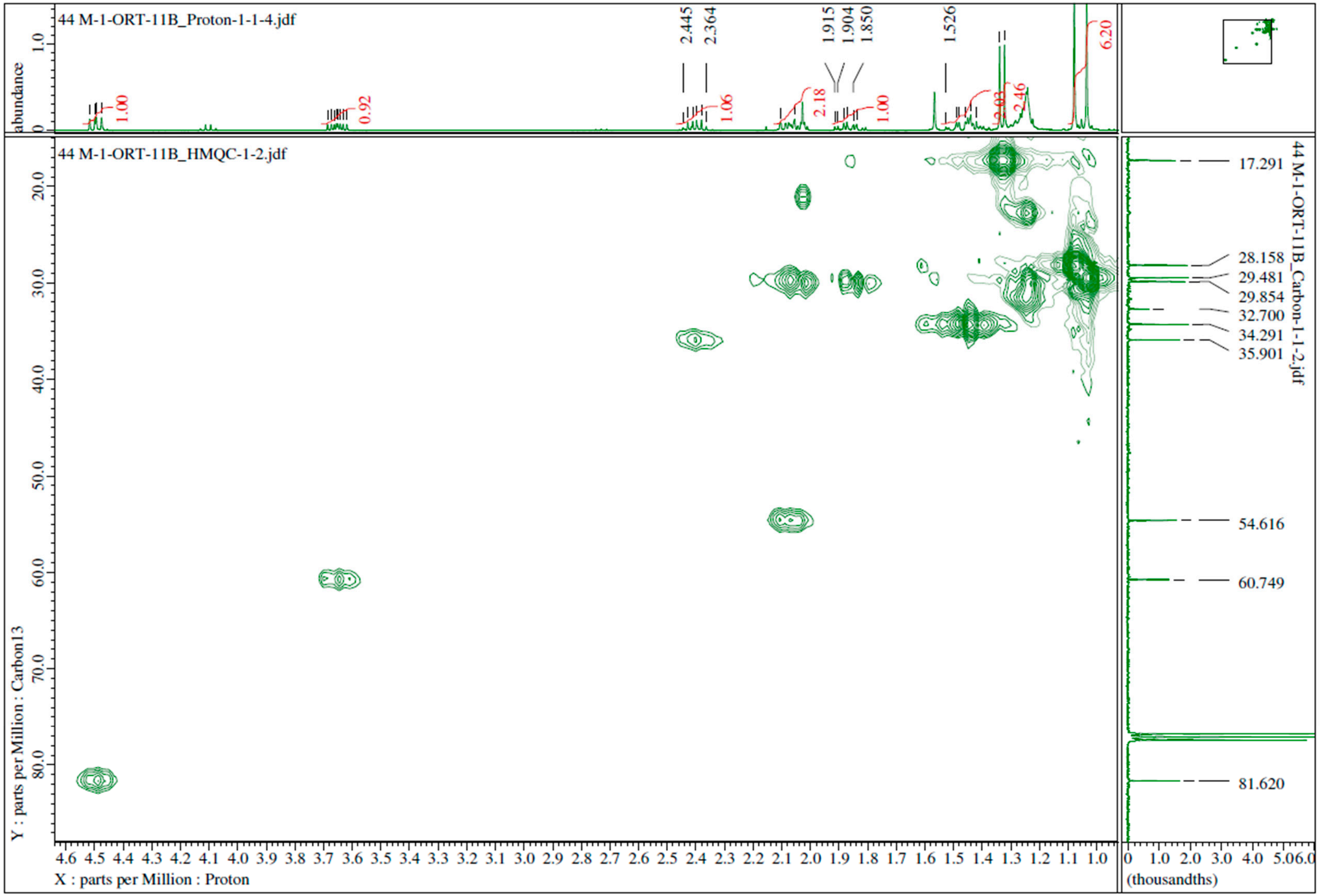

Figure S38. HMQC (100 MHz, $CDCl_3$) spectrum of chlorolactone **4a-B**

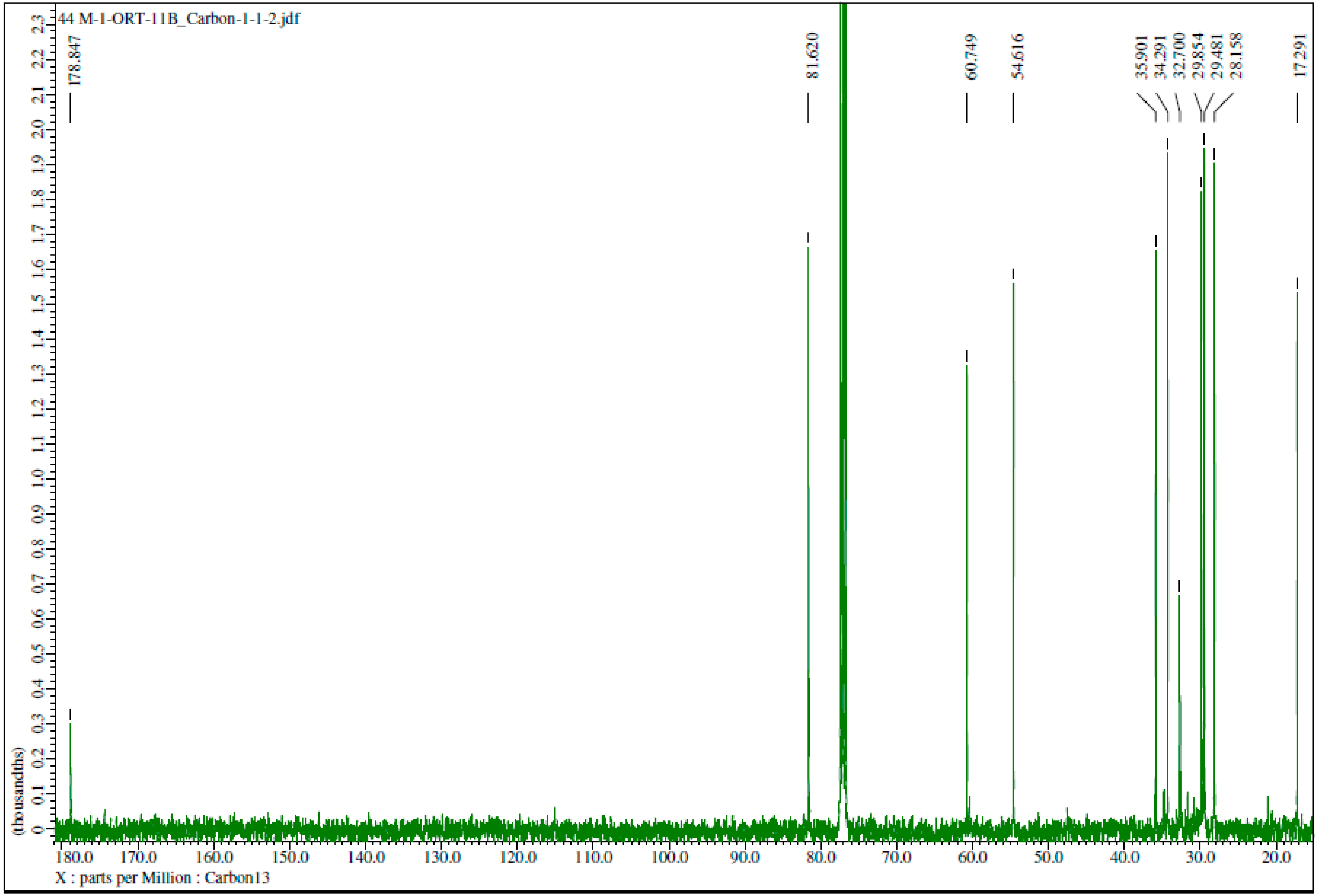

Figure S39. ^{13}C NMR (100 MHz, $CDCl_3$) spectrum of chlorolactone **4a-B**

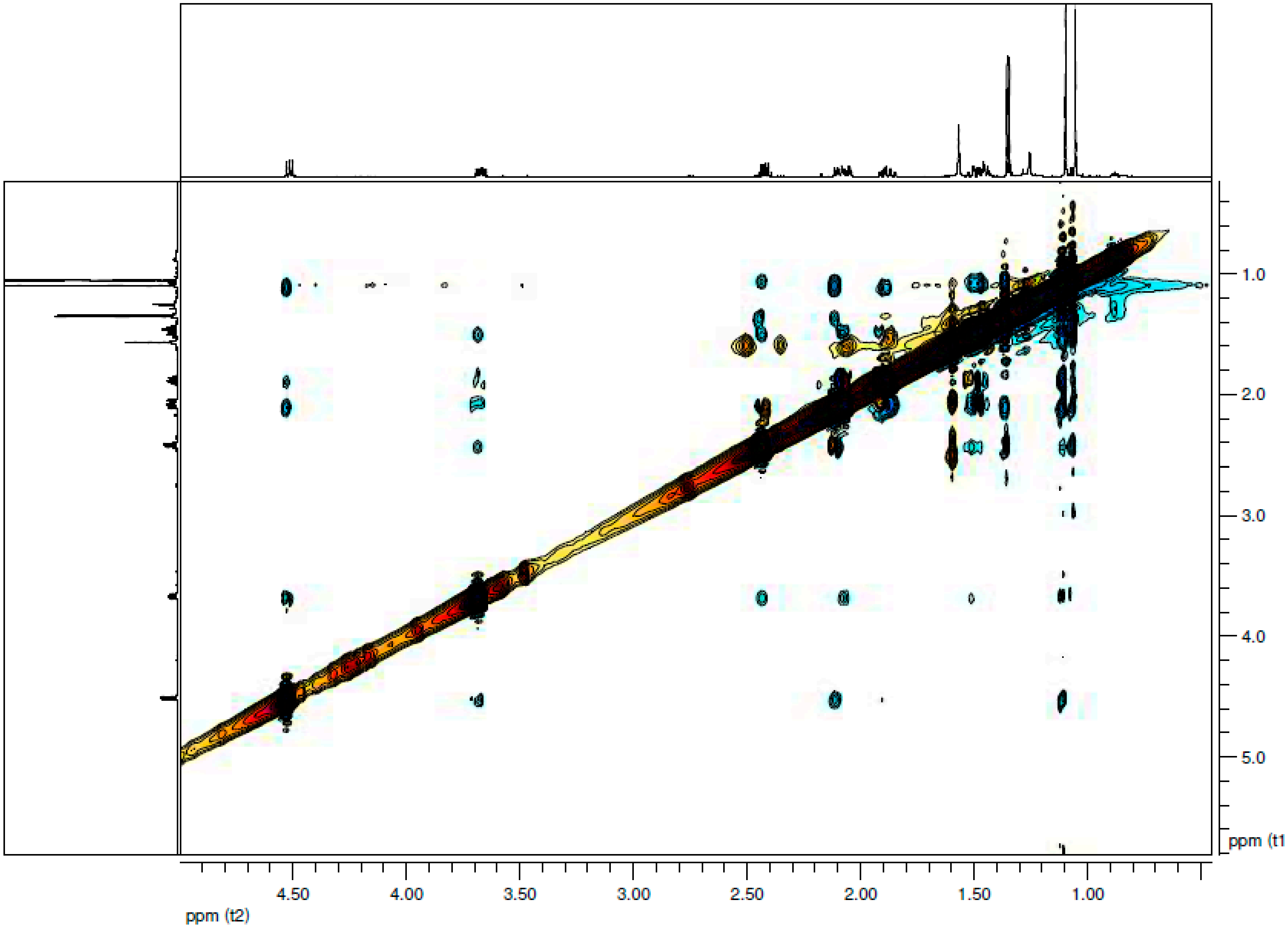

Figure S40.NOESY NMR (151 MHz, $CDCl_3$) spectrum of chlorolactone **4a-B**

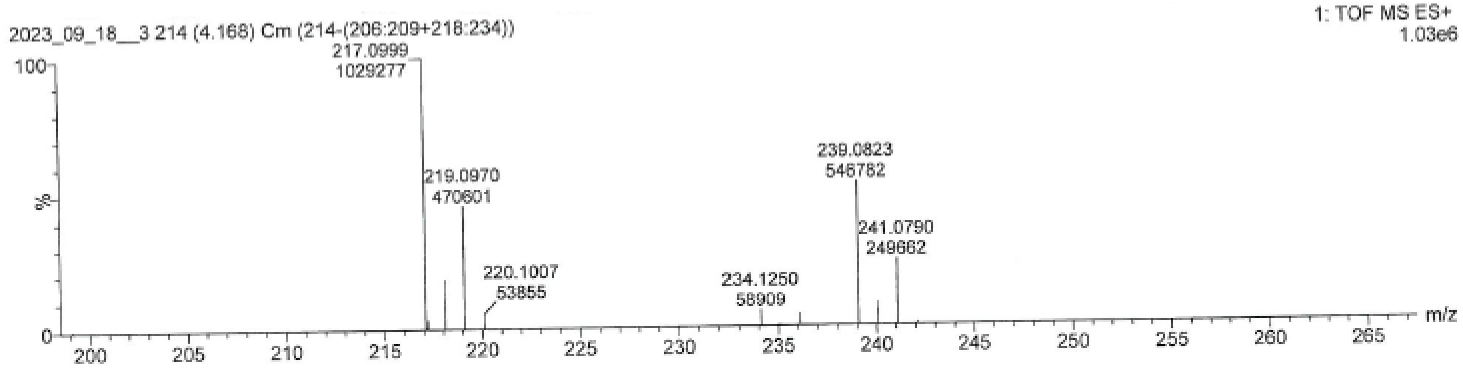

Figure S41. HRMS spectrum chlorolactone **4a**

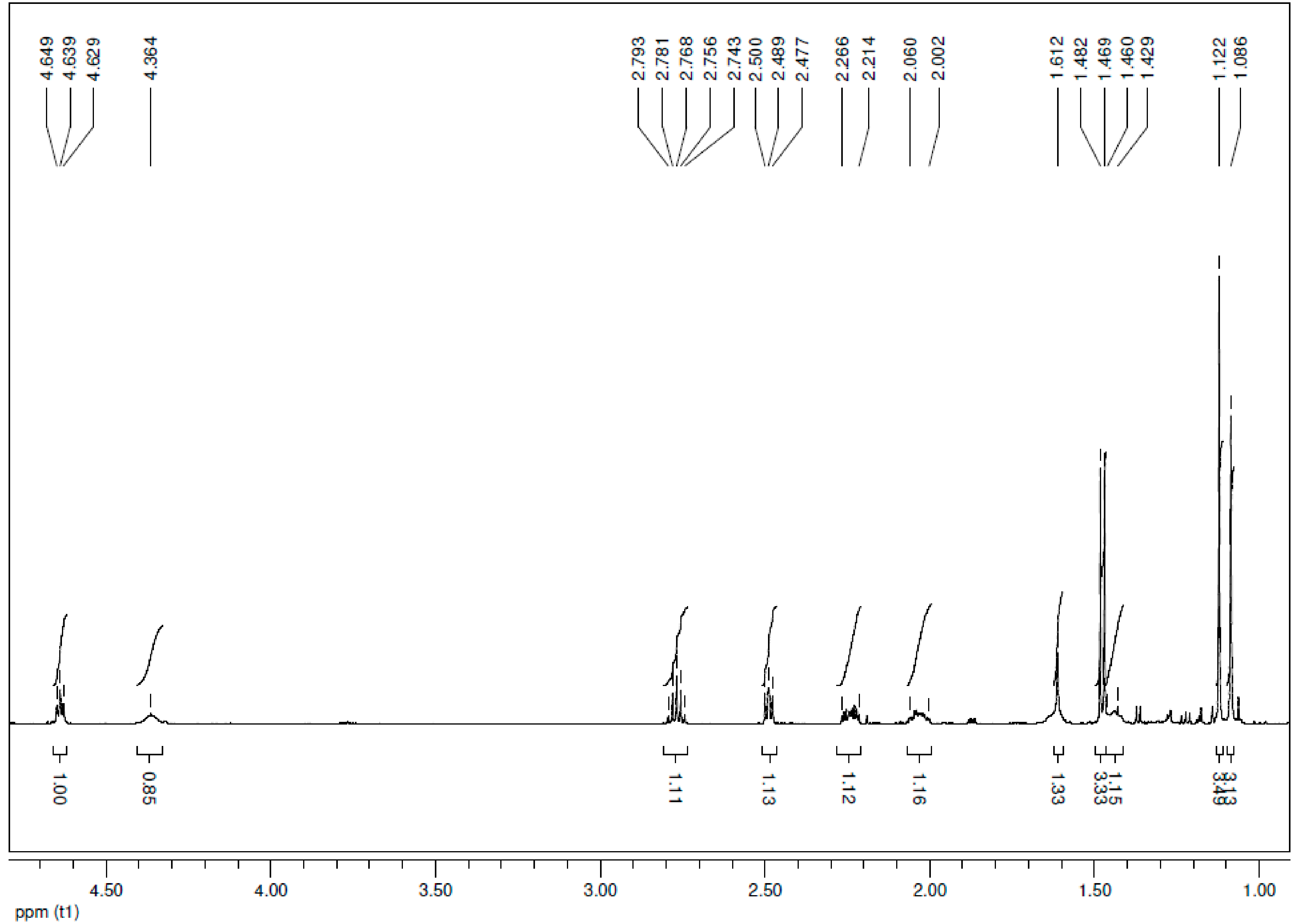

Figure S42. ^{1}H NMR (600 MHz, $CDCl_3$) spectrum of bromolactone **5a-A**

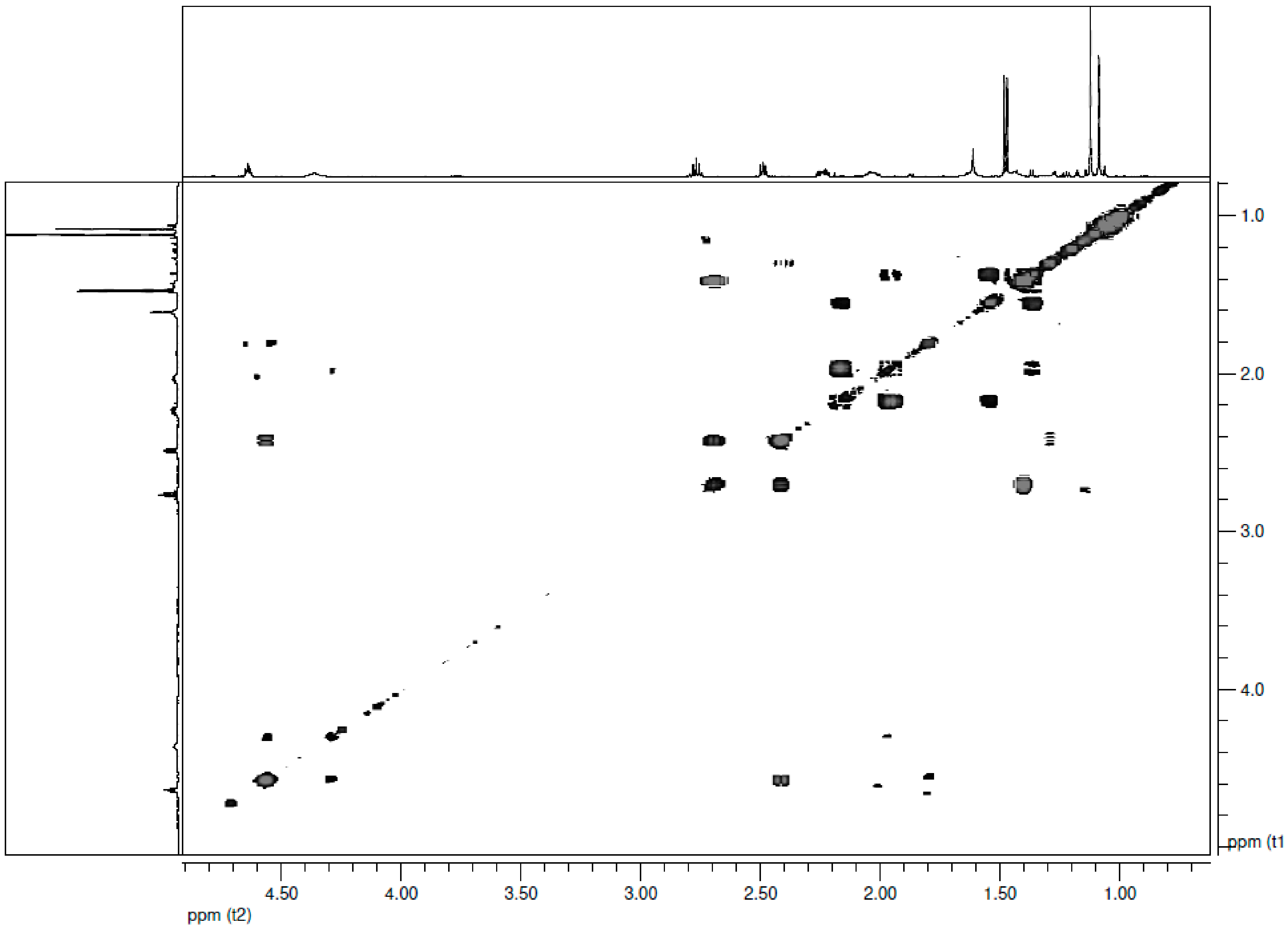

Figure S43. COSY (151 MHz, $CDCl_3$) spectrum of bromolactone **5a-A**

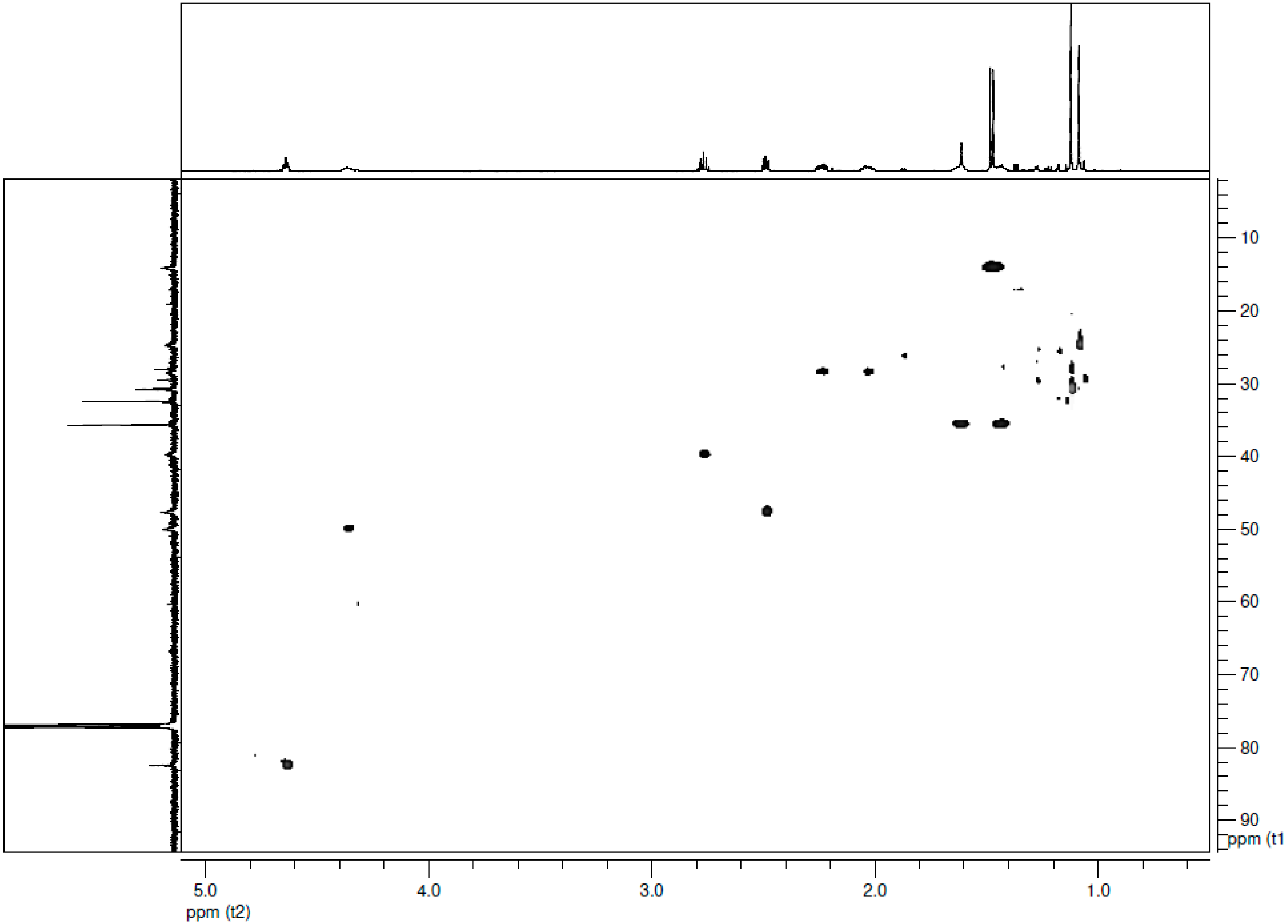

Figure S44. HMQC (151 MHz, $CDCl_3$) spectrum of bromolactone **5a-A**

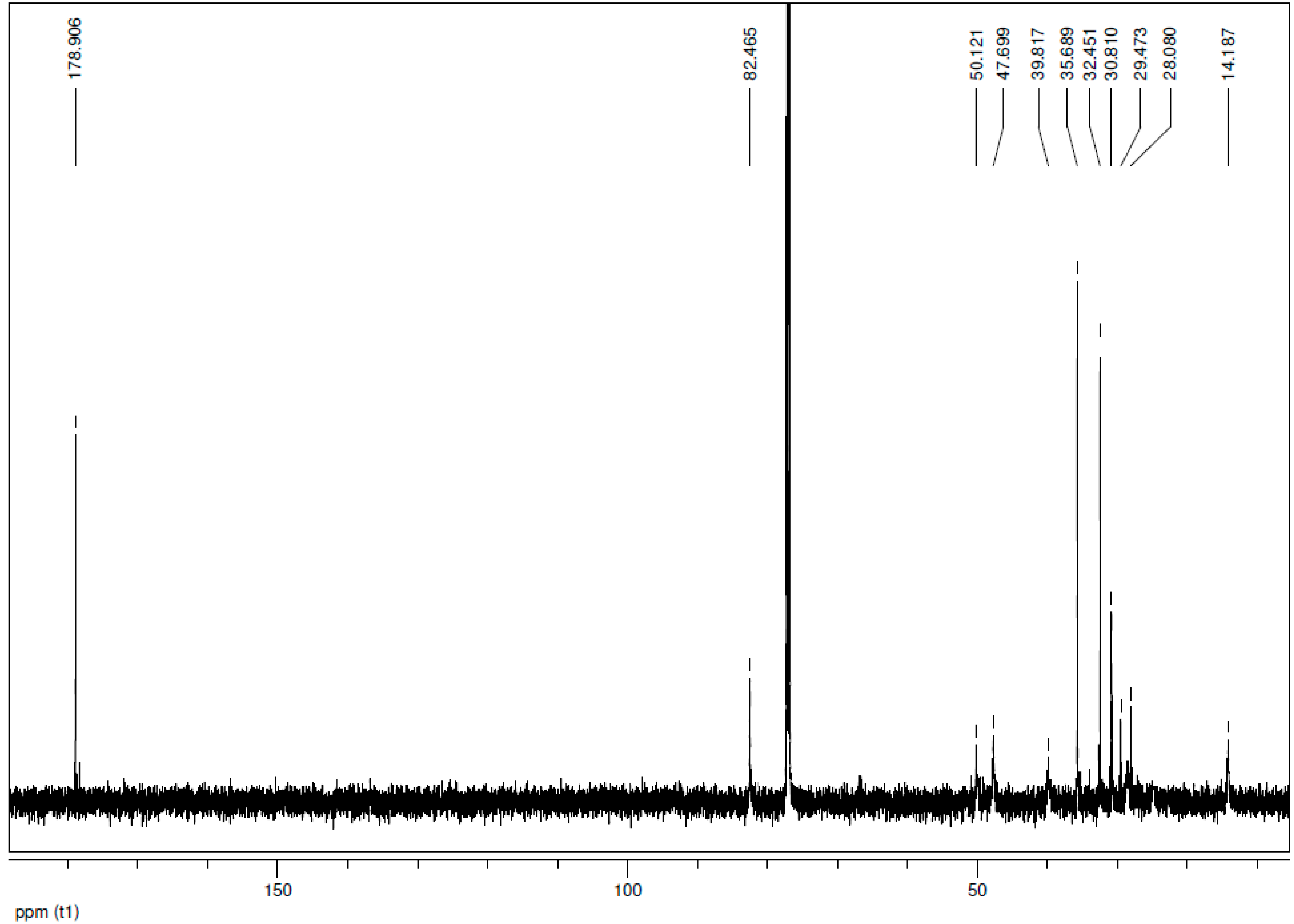

Figure S45. ^{13}C NMR (151 MHz, $CDCl_3$) spectrum bromolactone **5a-A**

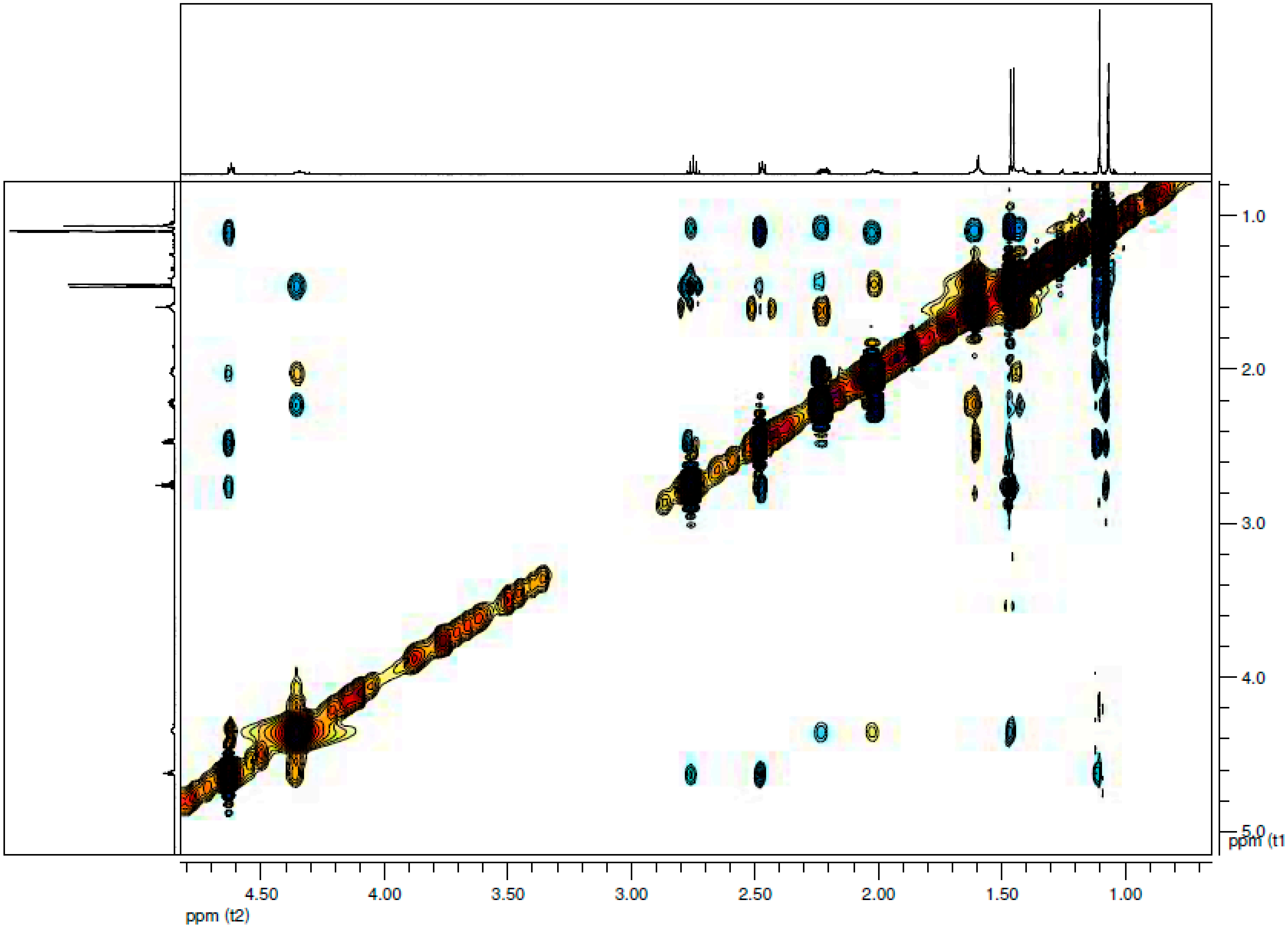

Figure S46.NOESY NMR (151 MHz, $CDCl_3$) spectrum bromolactone **5a-A**

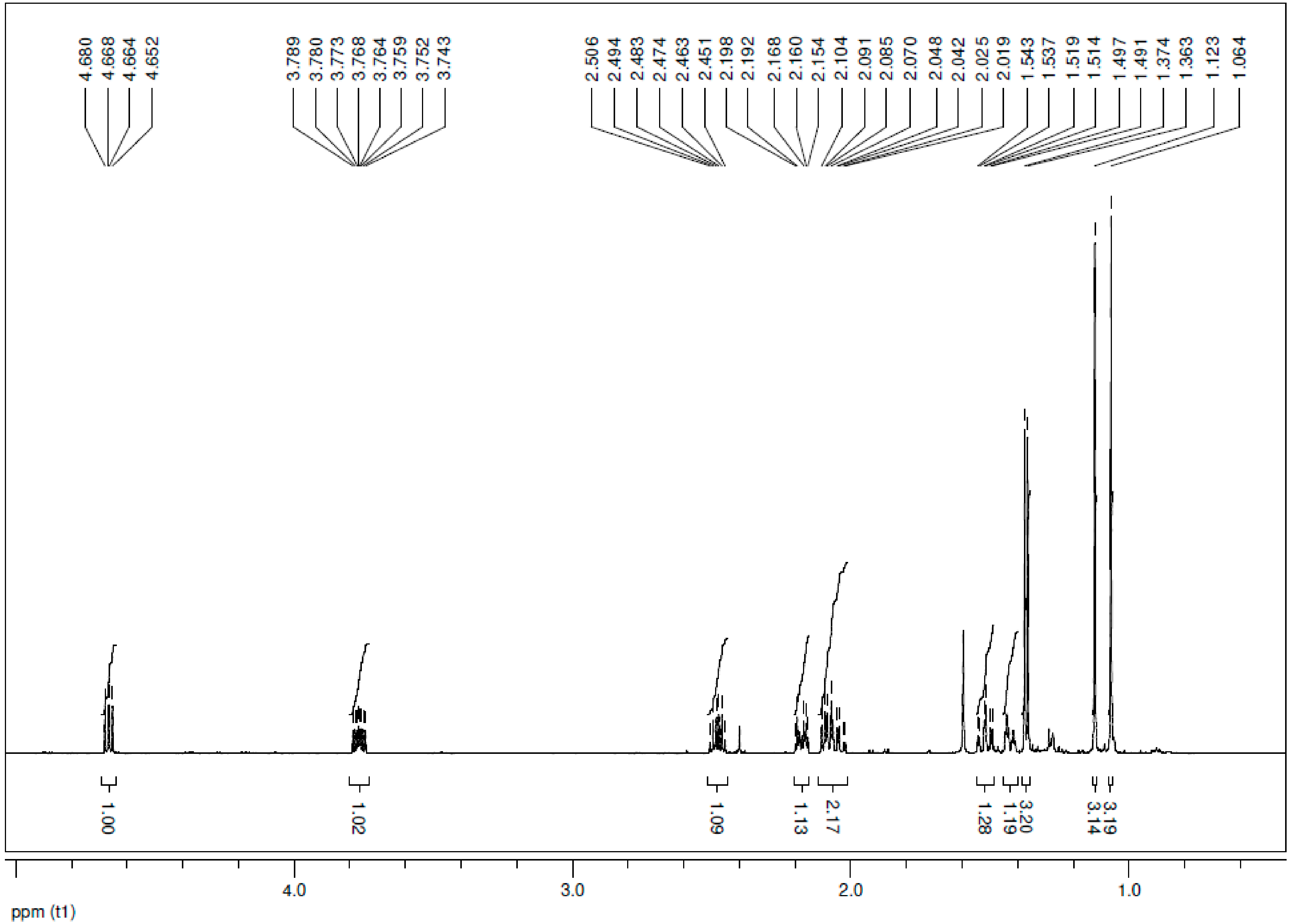

Figure S47. ^{1}H NMR (600 MHz, $CDCl_3$) spectrum of bromolactone **5a-B**

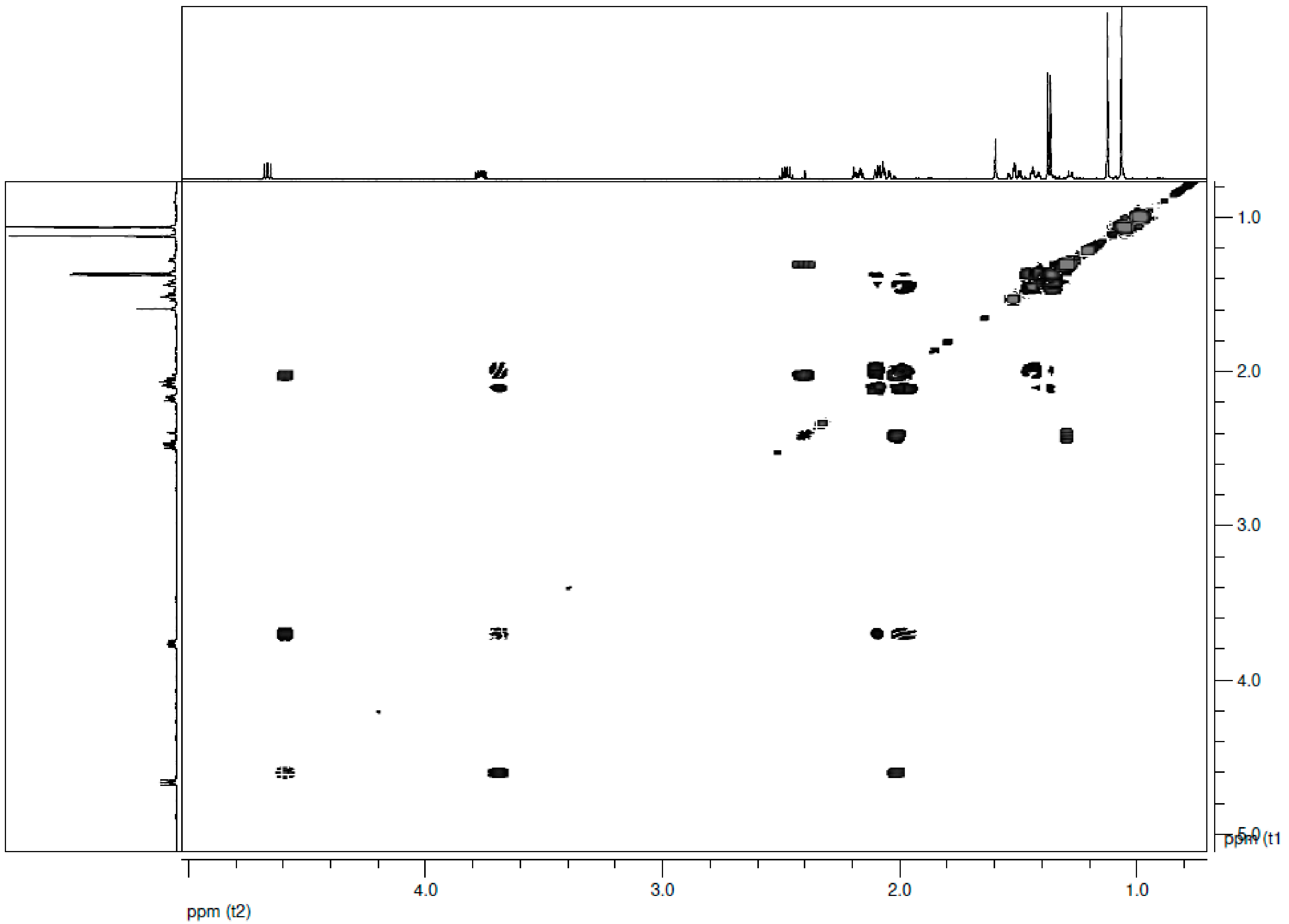

Figure S48. COSY (151 MHz, $CDCl_3$) spectrum of bromolactone **5a-B**

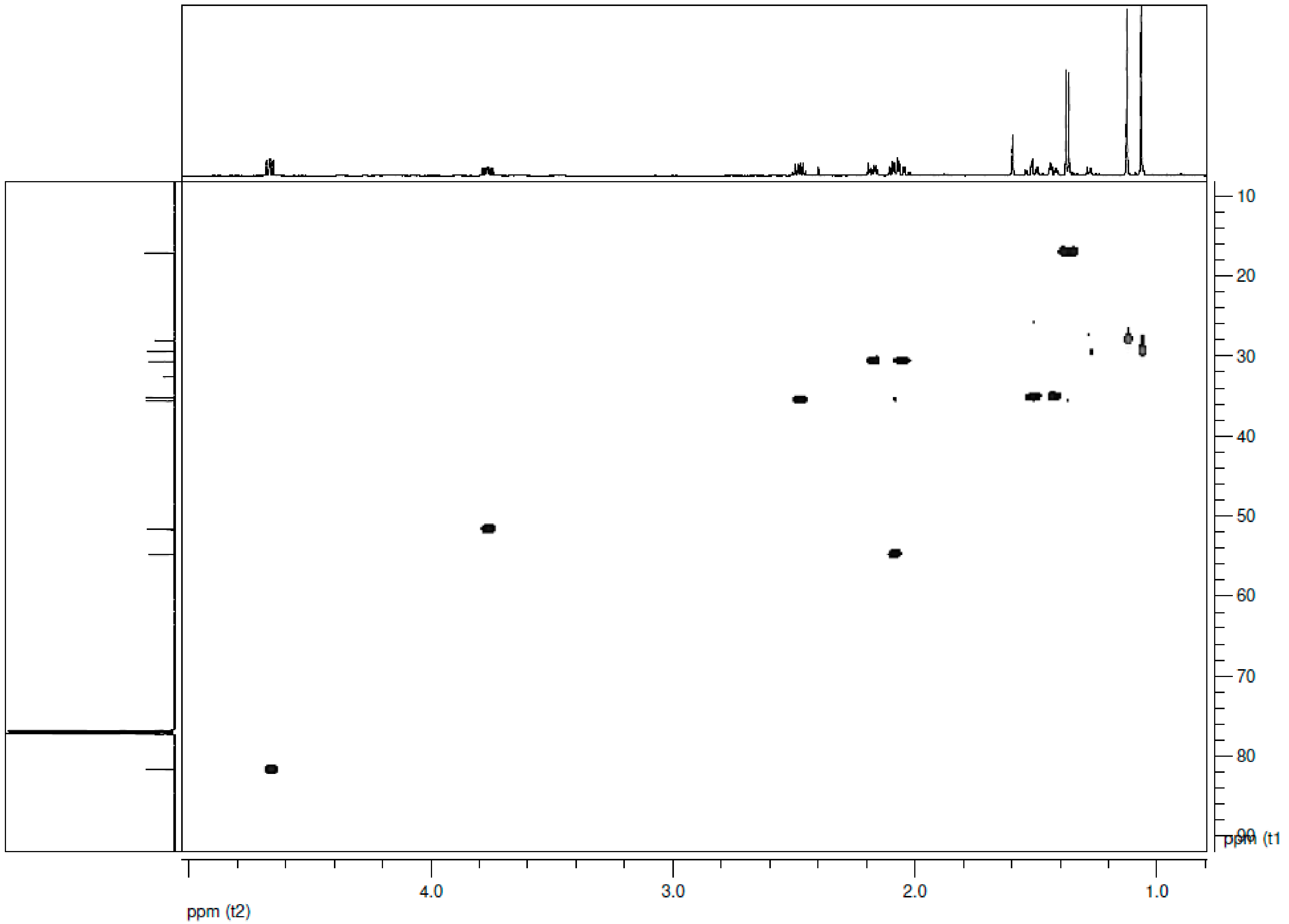

Figure S49. HMQC (151 MHz, $CDCl_3$) spectrum of bromolactone **5a-B**

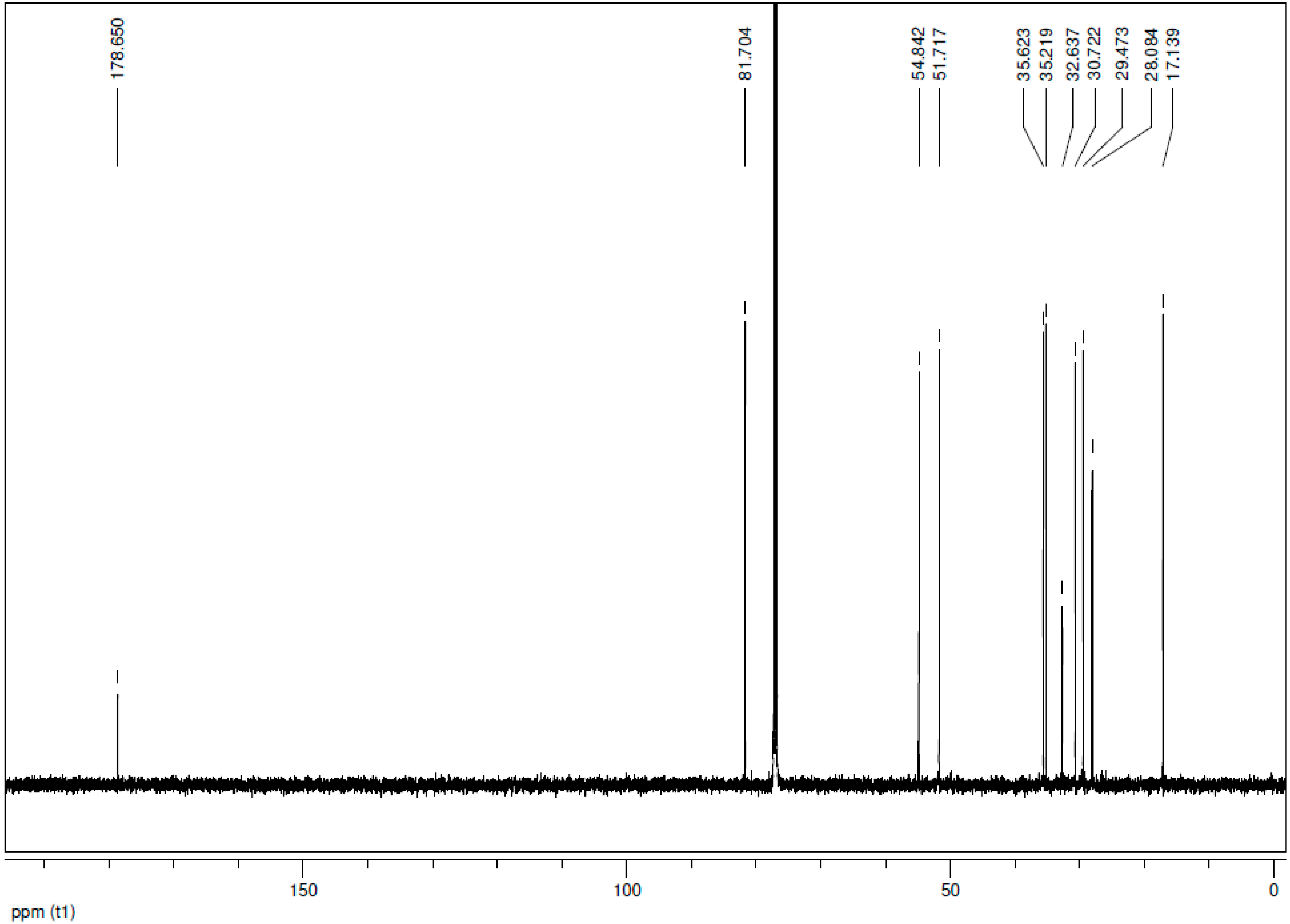

Figure S50. ^{13}C NMR (151 MHz, $CDCl_3$) spectrum bromolactone **5a-B**

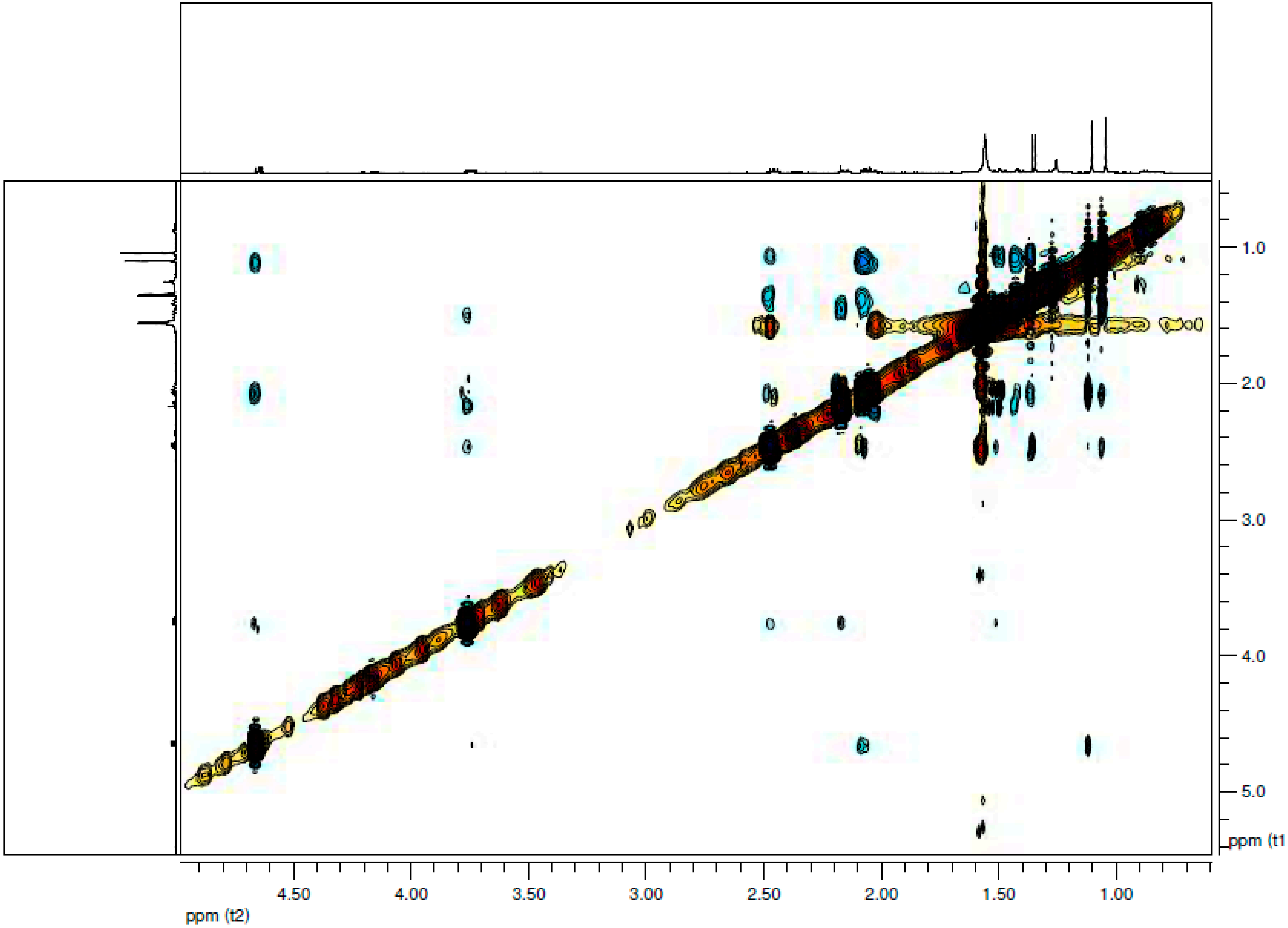

Figure S51.NOESY NMR (151 MHz, $CDCl_3$) spectrum bromolactone **5a-B**

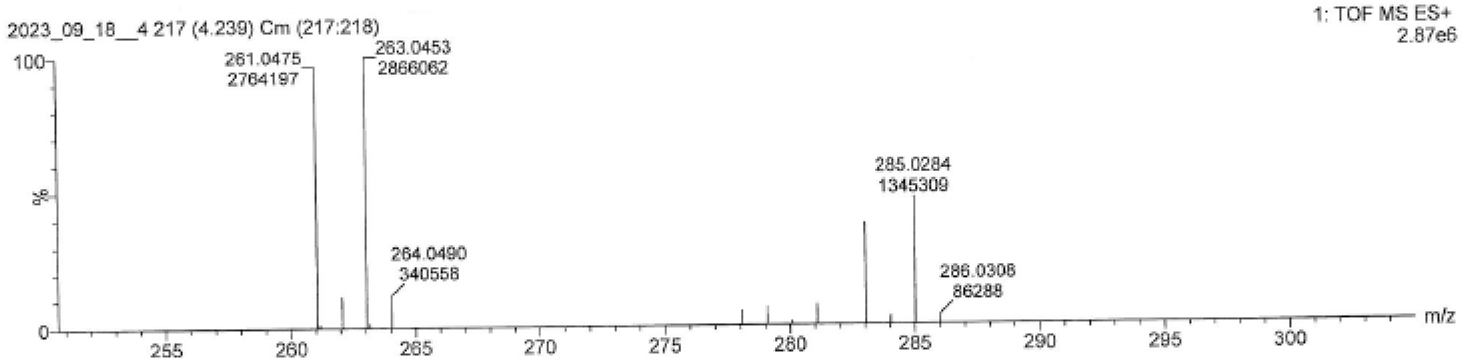

Figure S52. HRMS spectrum bromolactone **5a**

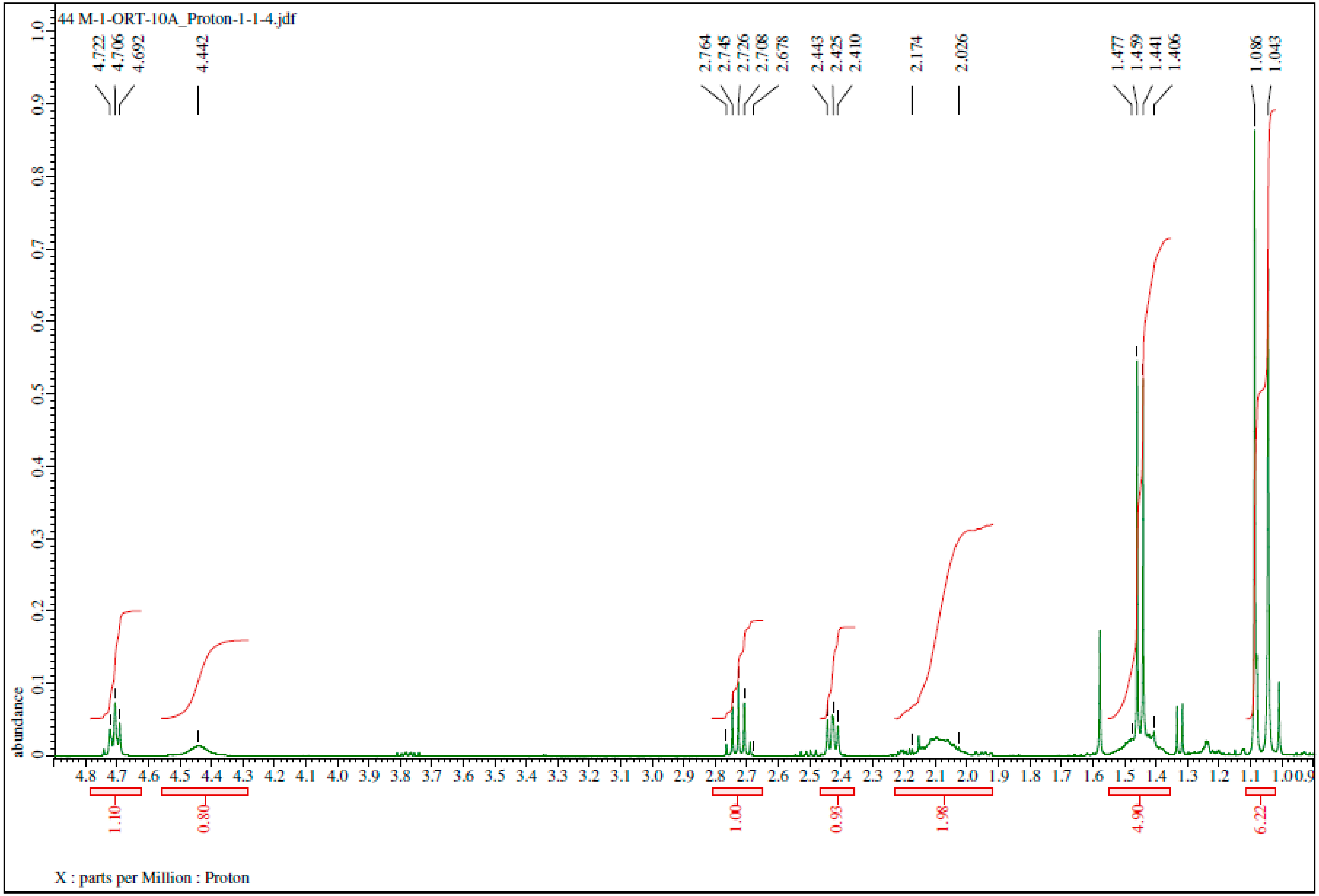

Figure S53. ^{1}H NMR (400 MHz, $CDCl_3$) spectrum of iodolactone **6a-A**

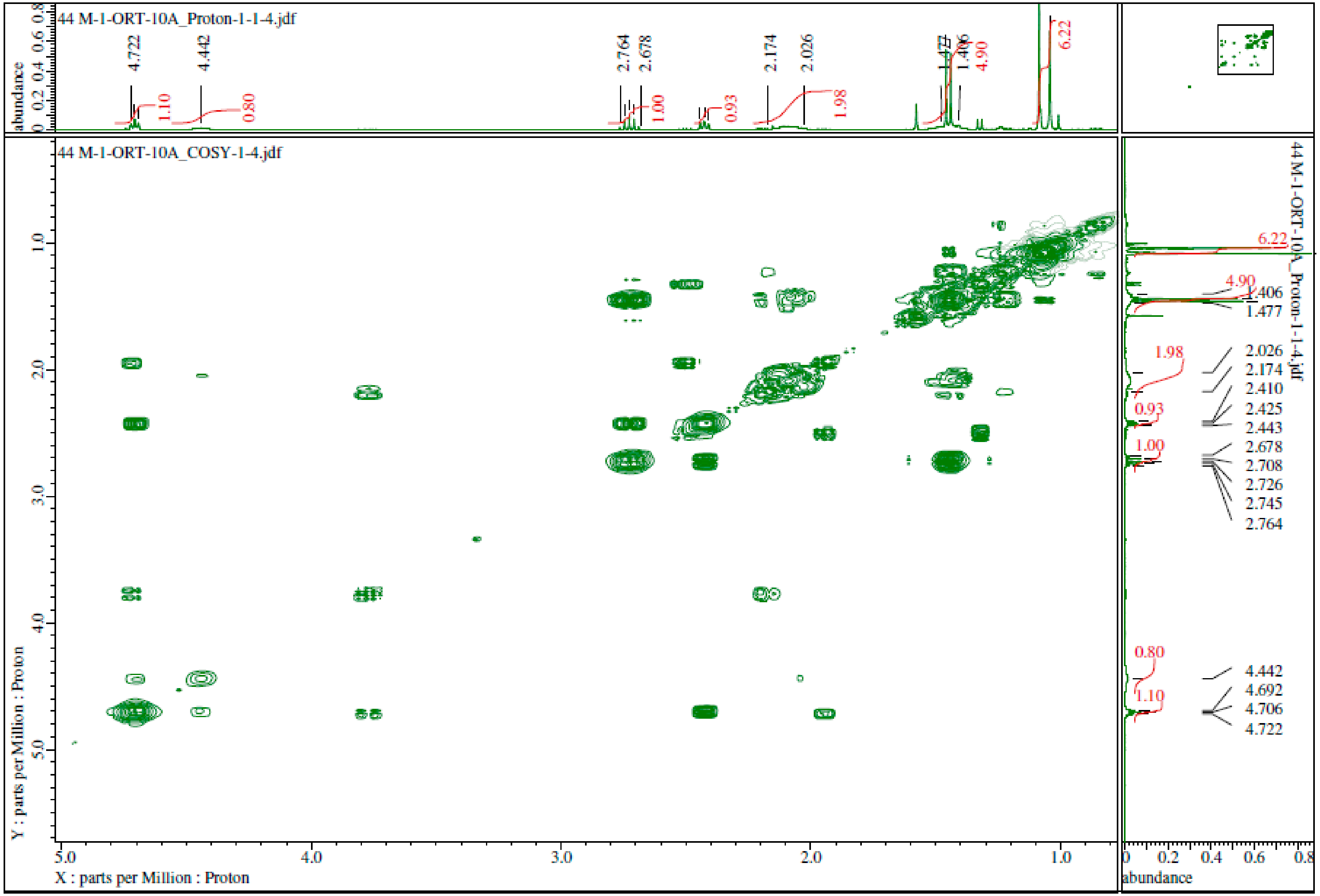

Figure S54. COSY (100 MHz, $CDCl_3$) spectrum of iodolactone **6a-A**

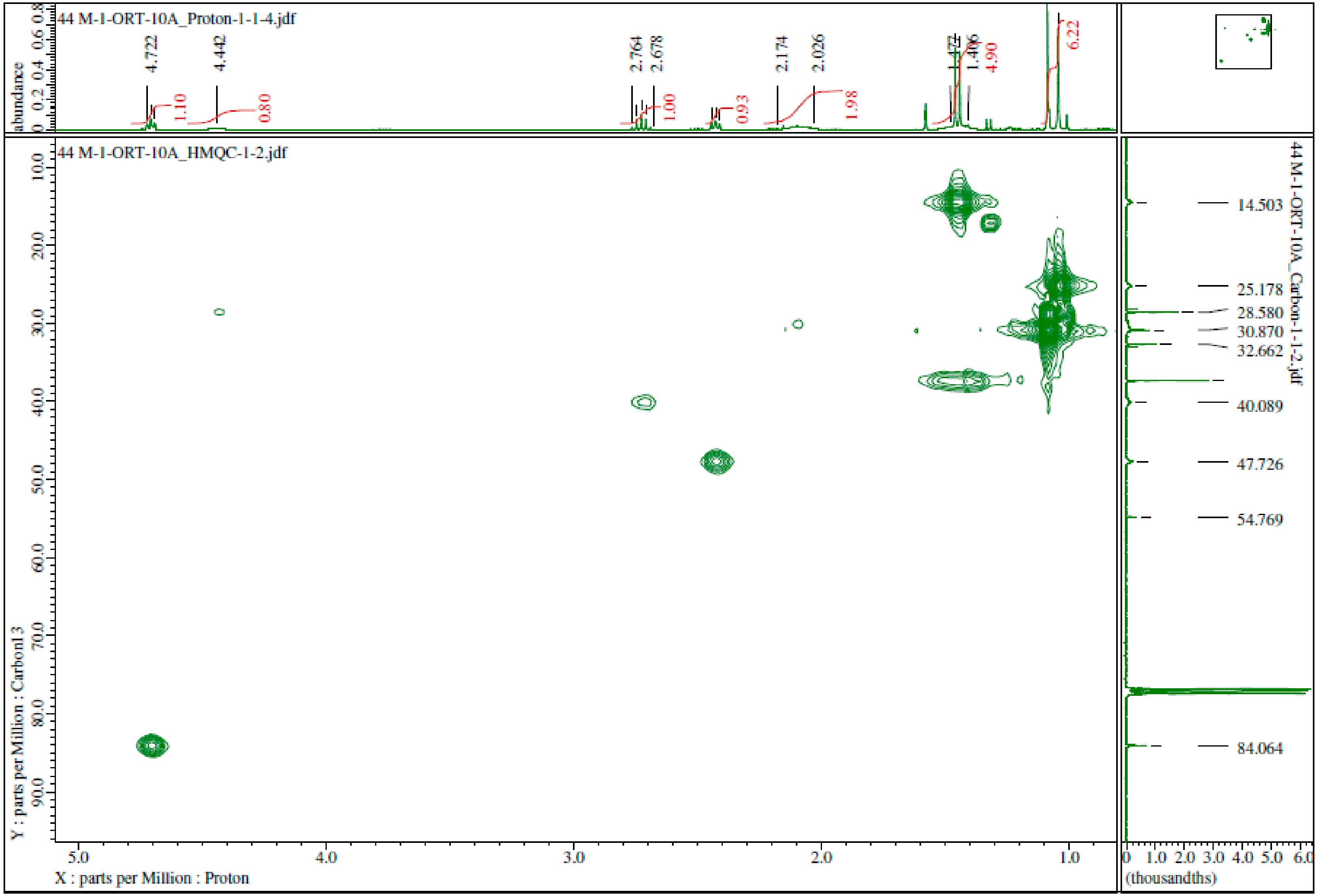

Figure S55. HMQC (100 MHz, $CDCl_3$) spectrum of iodolactone **6a-A**

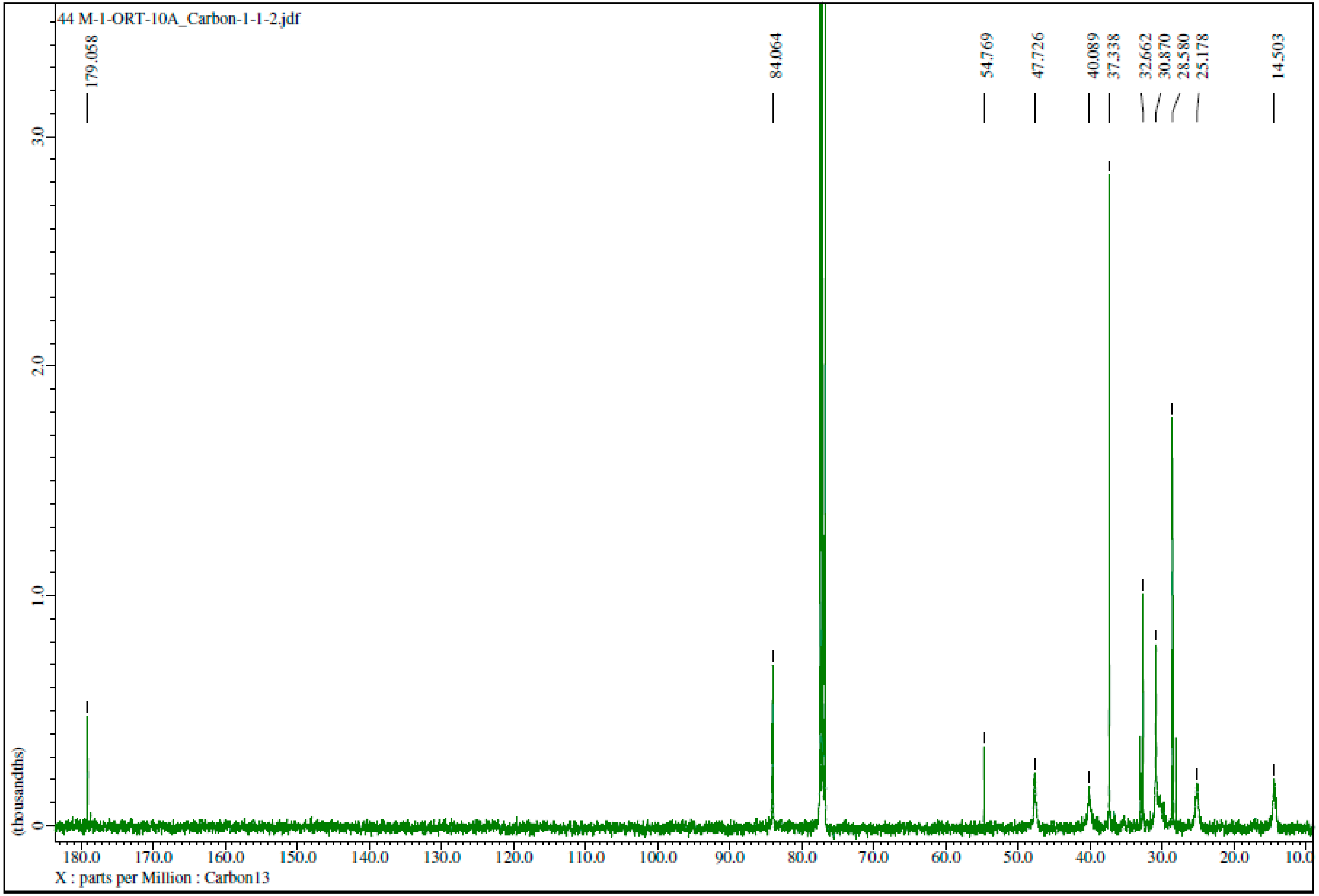

Figure S56. ^{13}C NMR (100 MHz, $CDCl_3$) spectrum of iodolactone **6a-A**

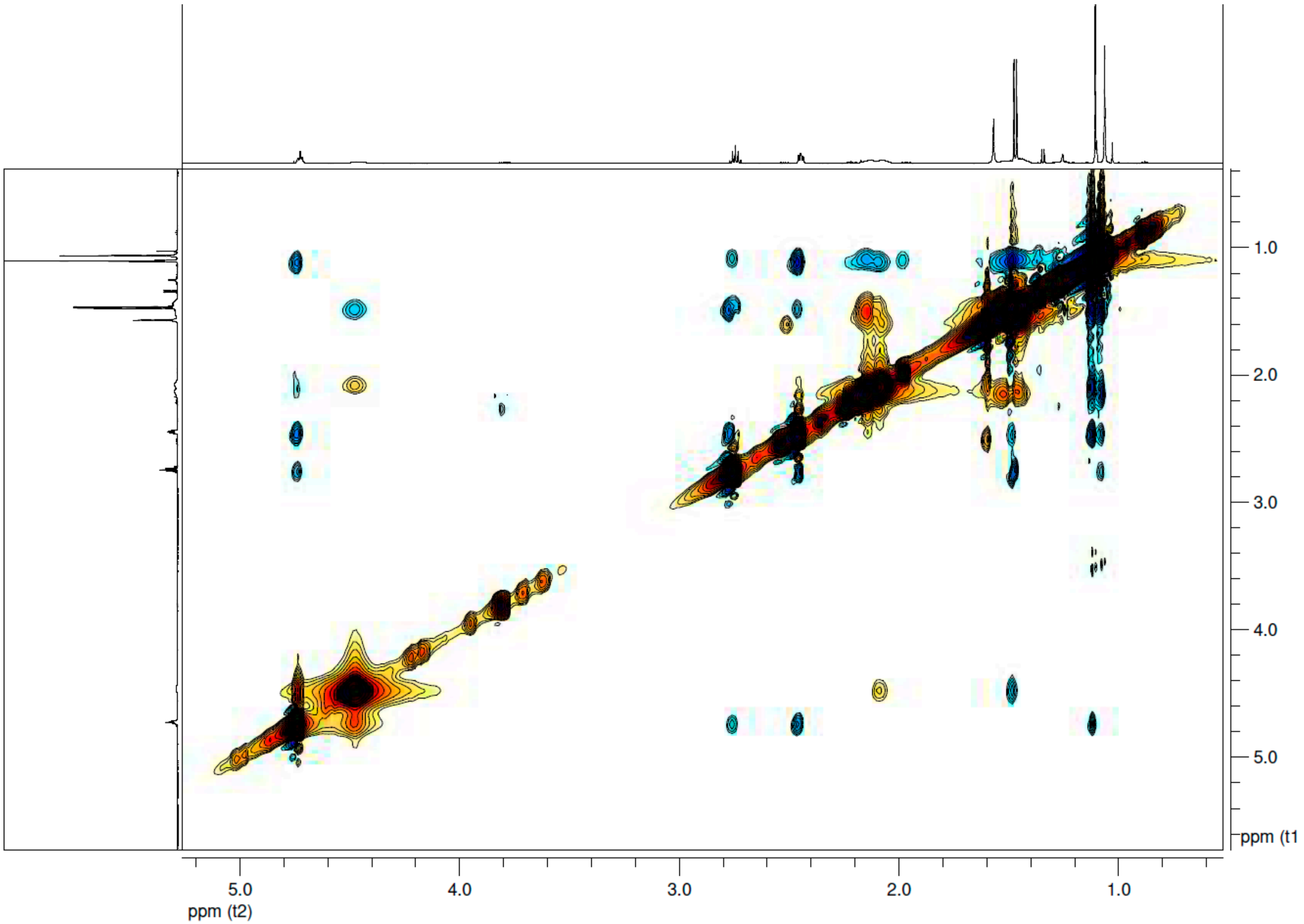

1.0
2.0
3.0
4.0
5.0
ppm (t1
5.0
4.0
3.0
2.0
1.0
ppm (t2)

Figure S57.NOESY NMR (151 MHz, $CDCl_3$) spectrum iodolactone **6a-A**

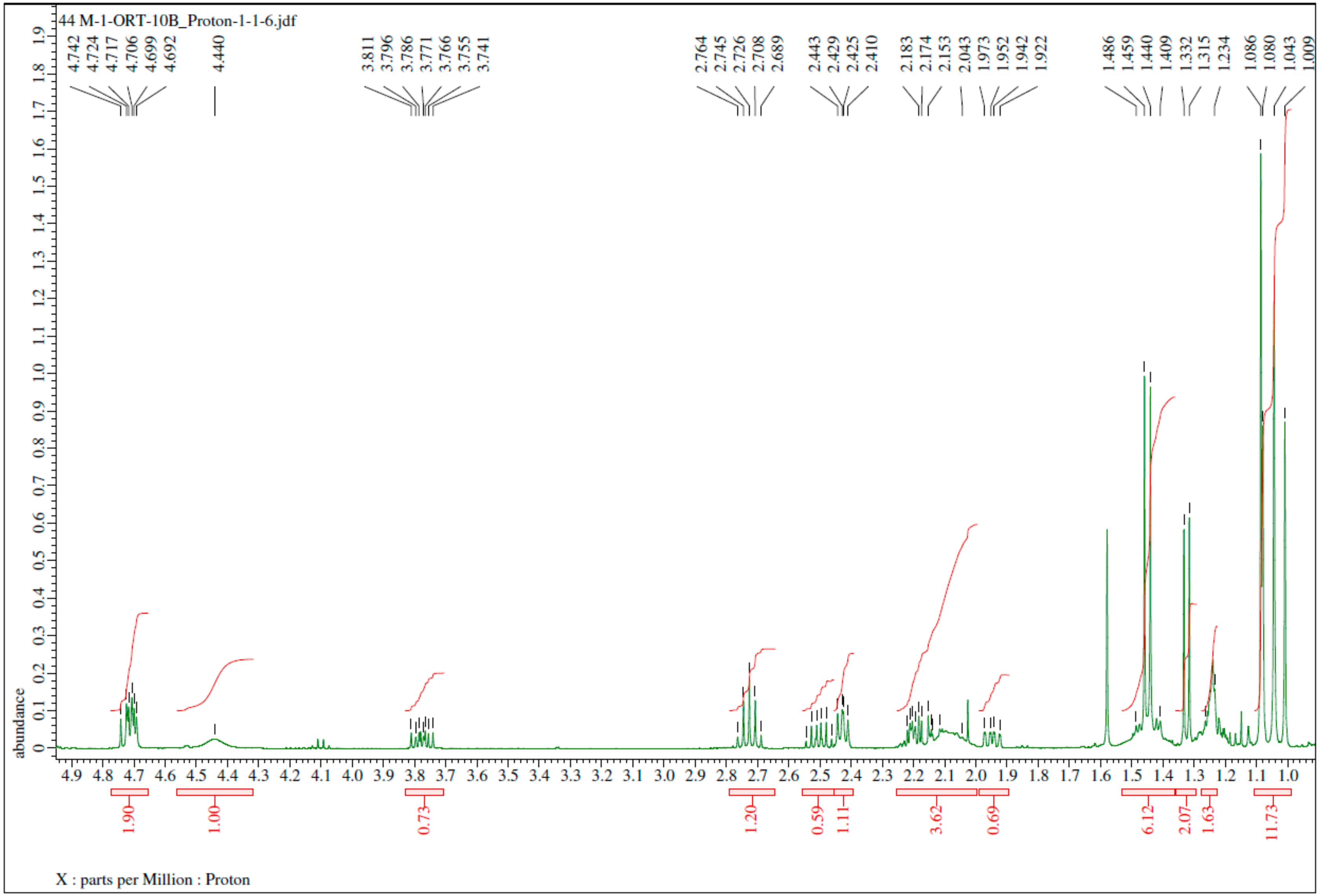

Figure S58. ^{1}H NMR (400 MHz, $CDCl_3$) spectrum of iodolactone **6a-B+A**

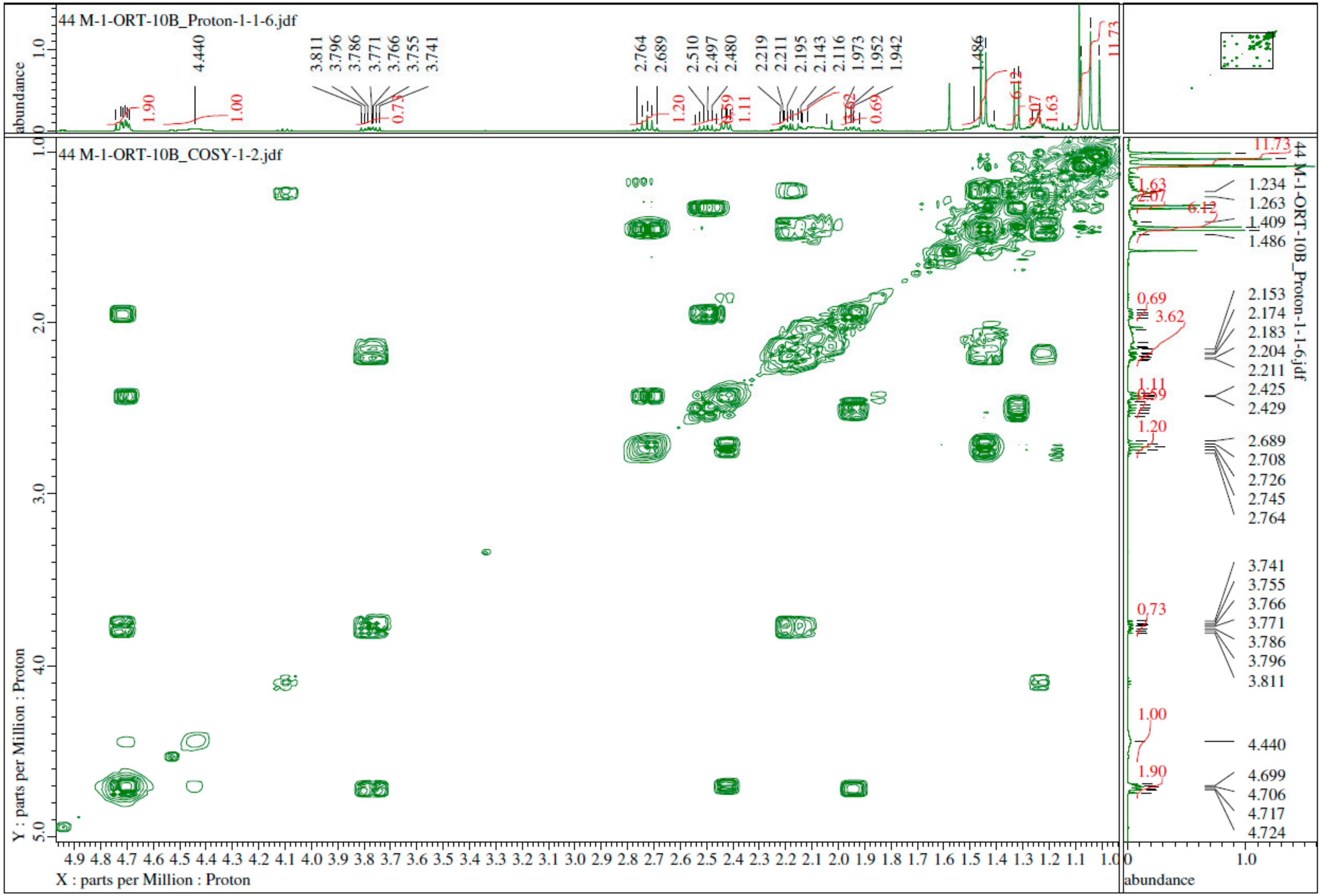

Figure S59. COSY (100 MHz, $CDCl_3$) spectrum of iodolactone **6a-B+A**

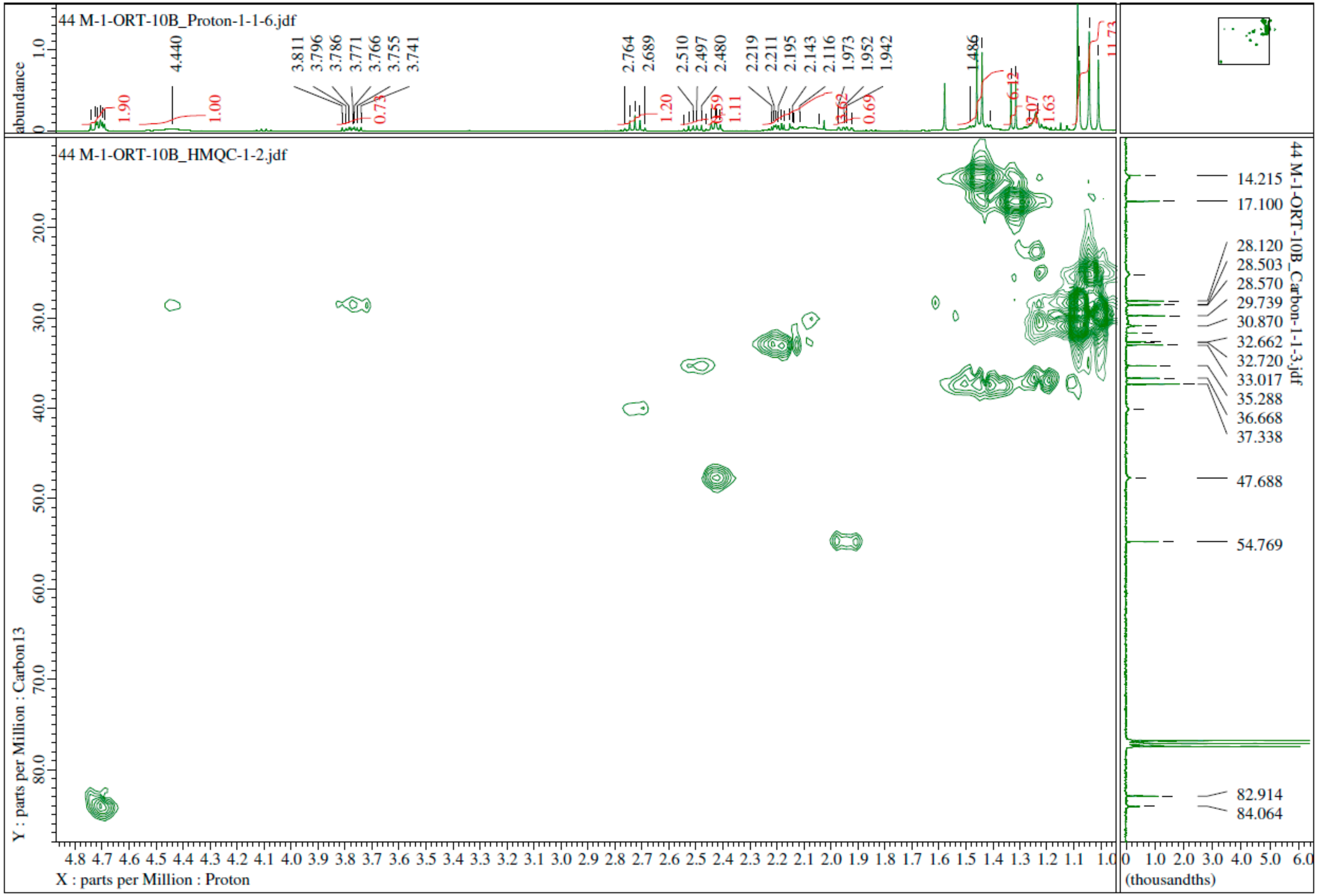

Figure S60. HMQC (100 MHz, $CDCl_3$) spectrum of iodolactone **6a-B+A**

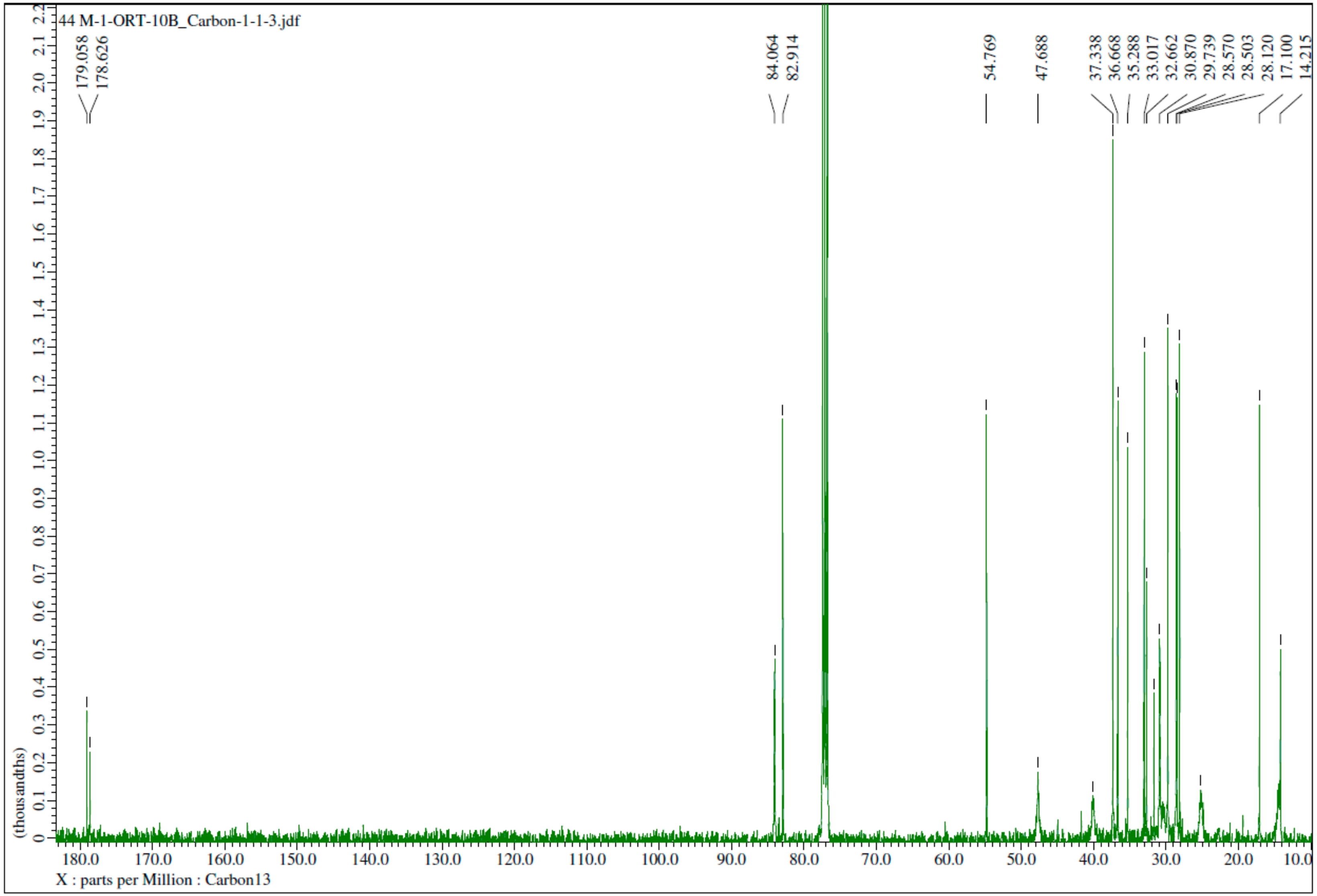

Figure S61. ^{13}C NMR (100 MHz, $CDCl_3$) spectrum of iodolactone **6a-B+A**

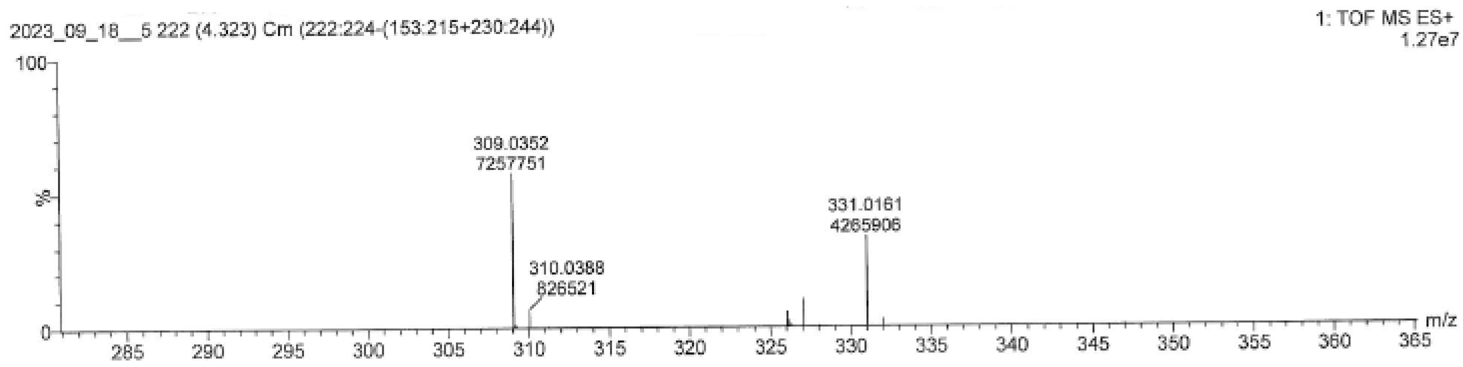

Figure S62. HRMS spectrum of iodolactone **6a**

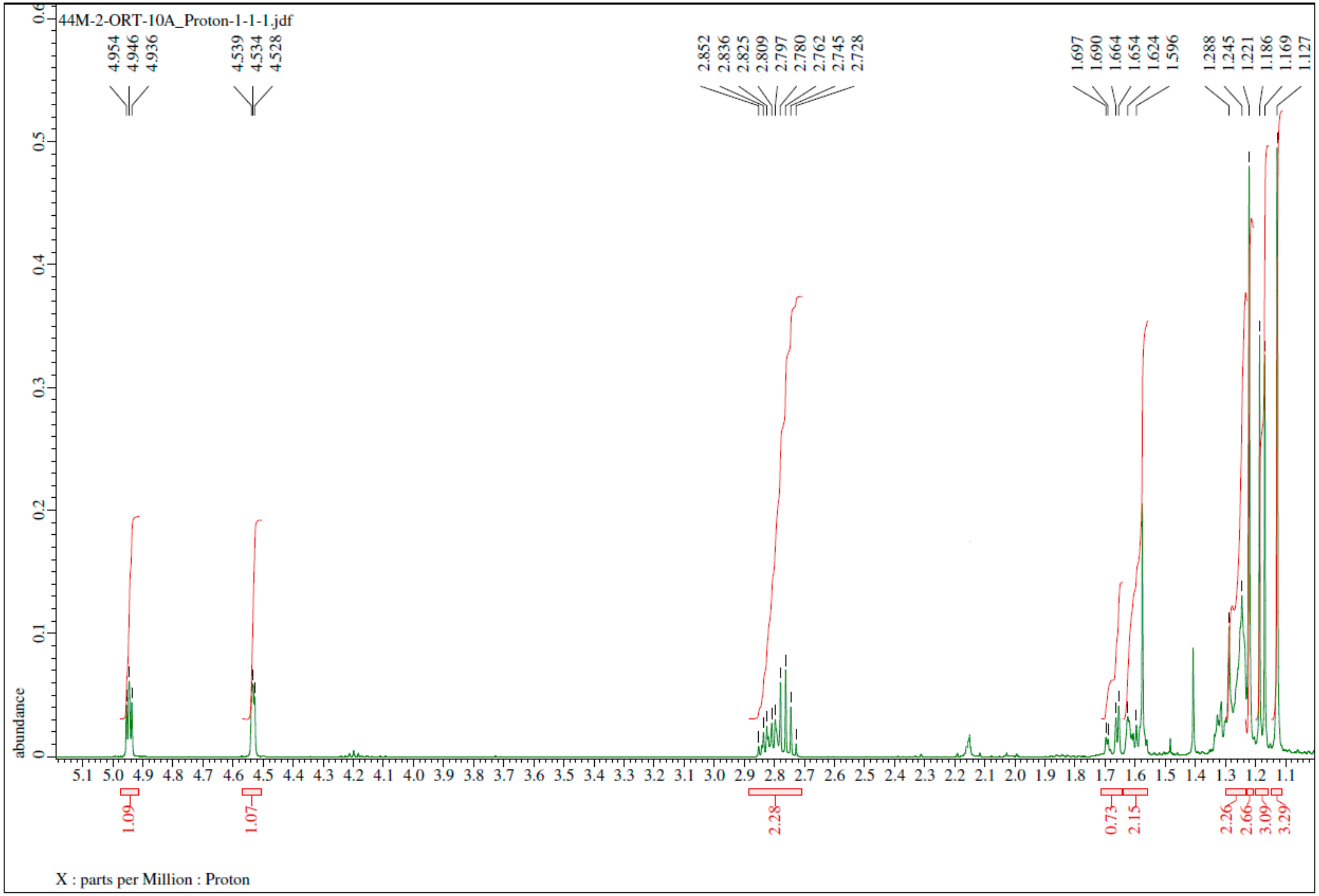

Figure S63. ^{1}H NMR (400 MHz, $CDCl_3$) spectrum of chlorolactone **4b-A**

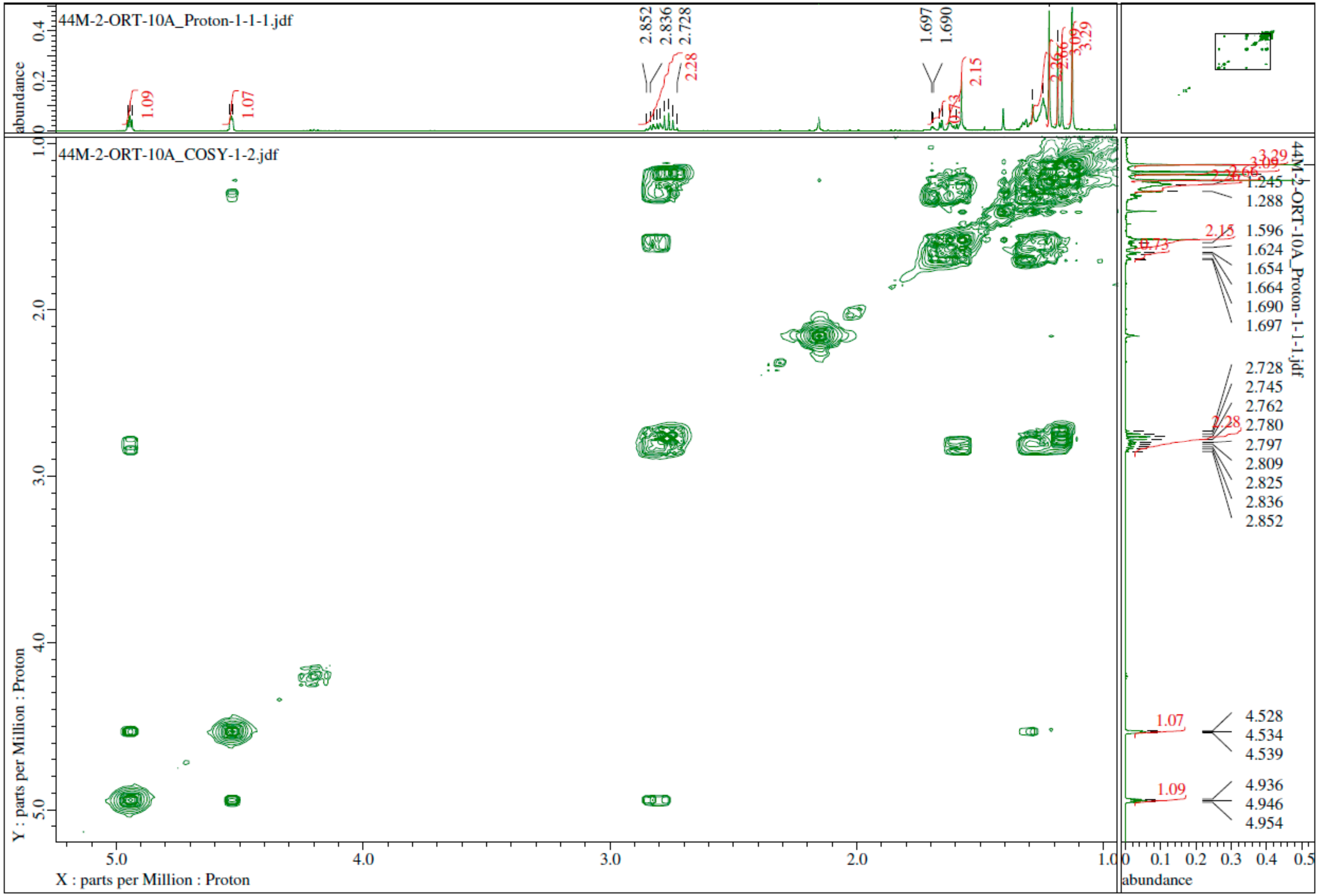

Figure S64. COSY (100 MHz, $CDCl_3$) spectrum of chlorolactone **4b-A**

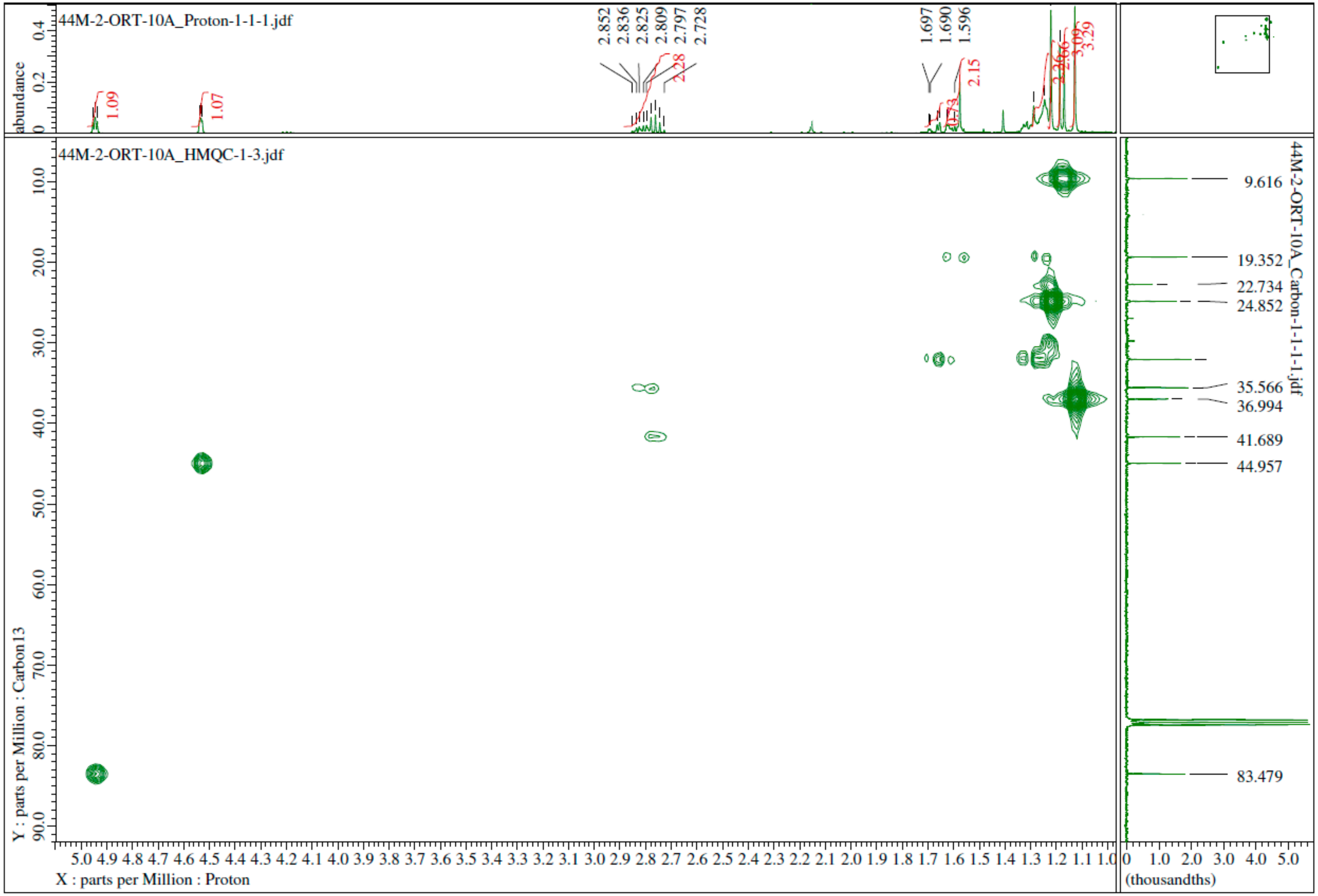

Figure S65. HMQC (100 MHz, $CDCl_3$) spectrum of chlorolactone **4b-A**

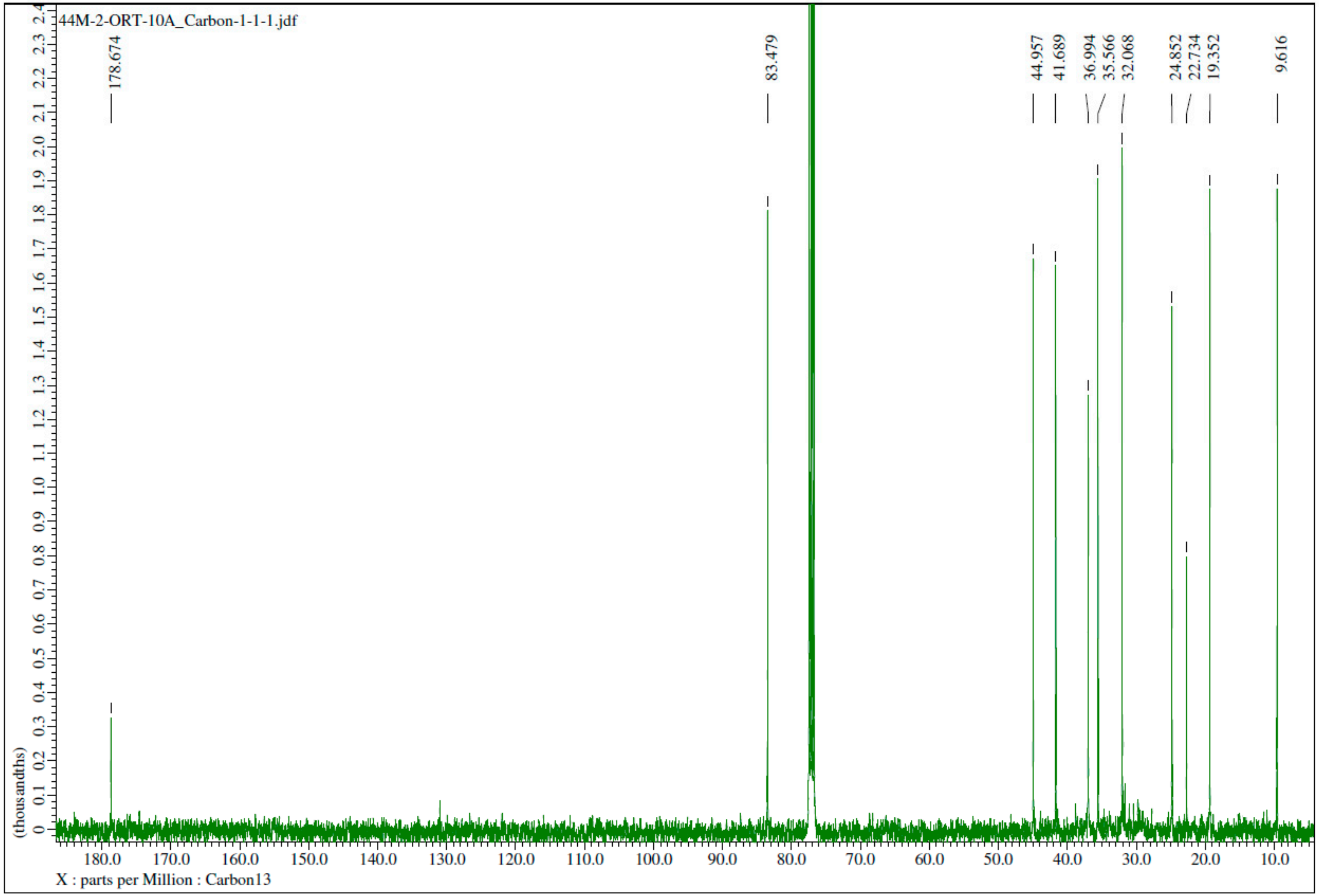

Figure S66. ^{13}C NMR (100 MHz, $CDCl_3$) spectrum of chlorolactone **4b-A**

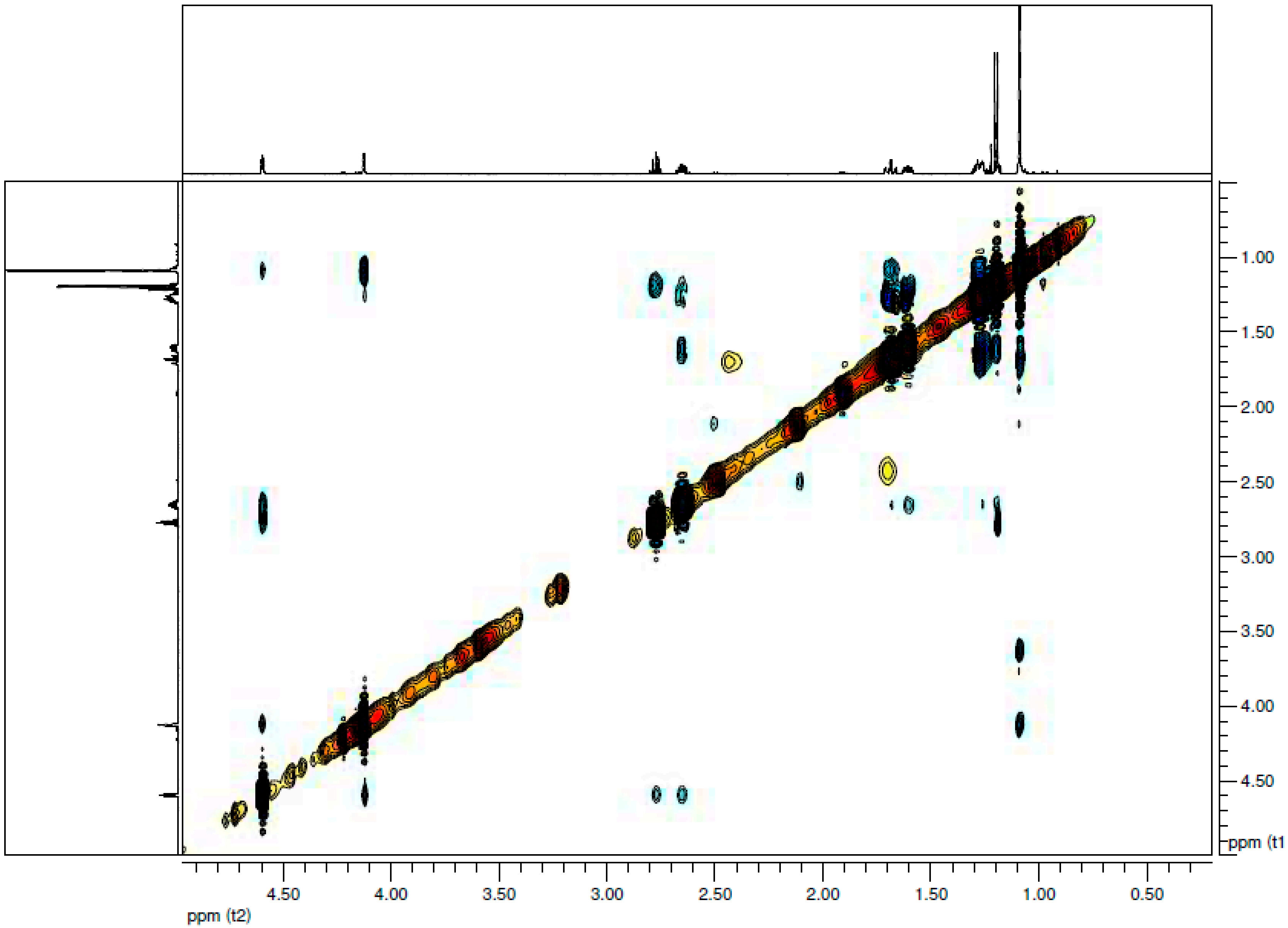

Figure S67.NOESY NMR (151 MHz, $CDCl_3$) spectrum of chlorolactone **4b-A**

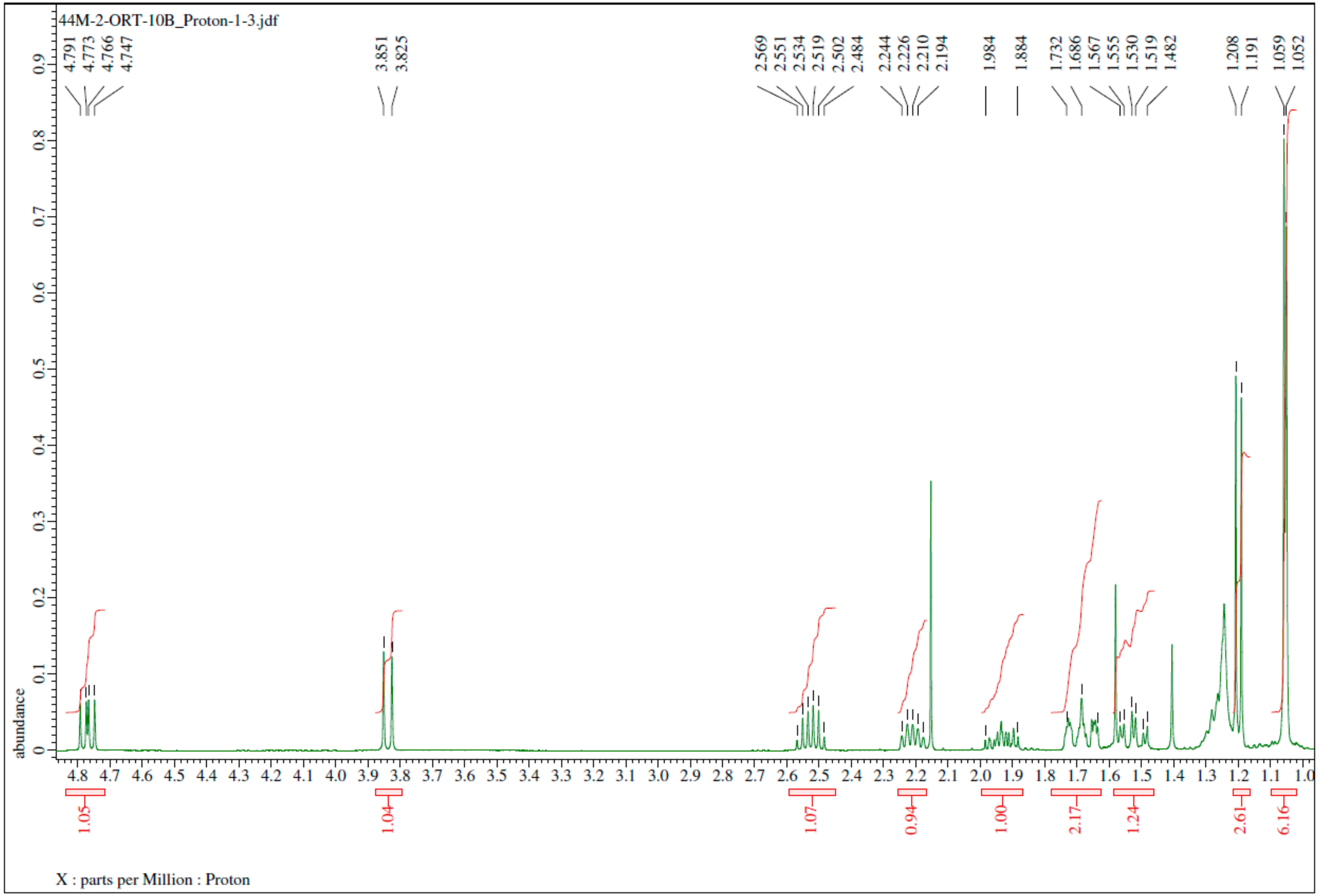

Figure S68. ^{1}H NMR (400 MHz, $CDCl_3$) spectrum of chlorolactone **4b-B**

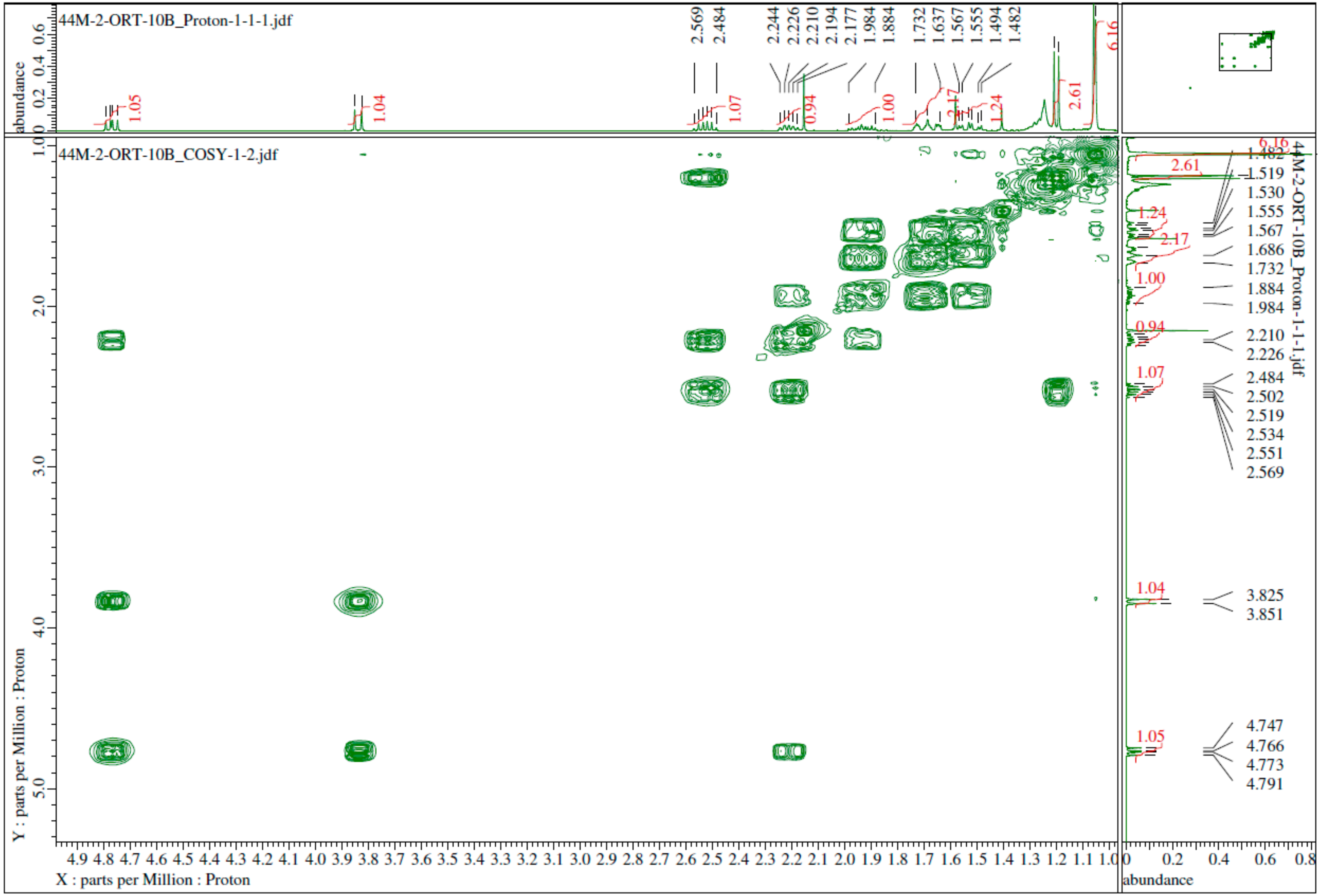

Figure S69. COSY (100 MHz, $CDCl_3$) spectrum of chlorolactone **4b-B**

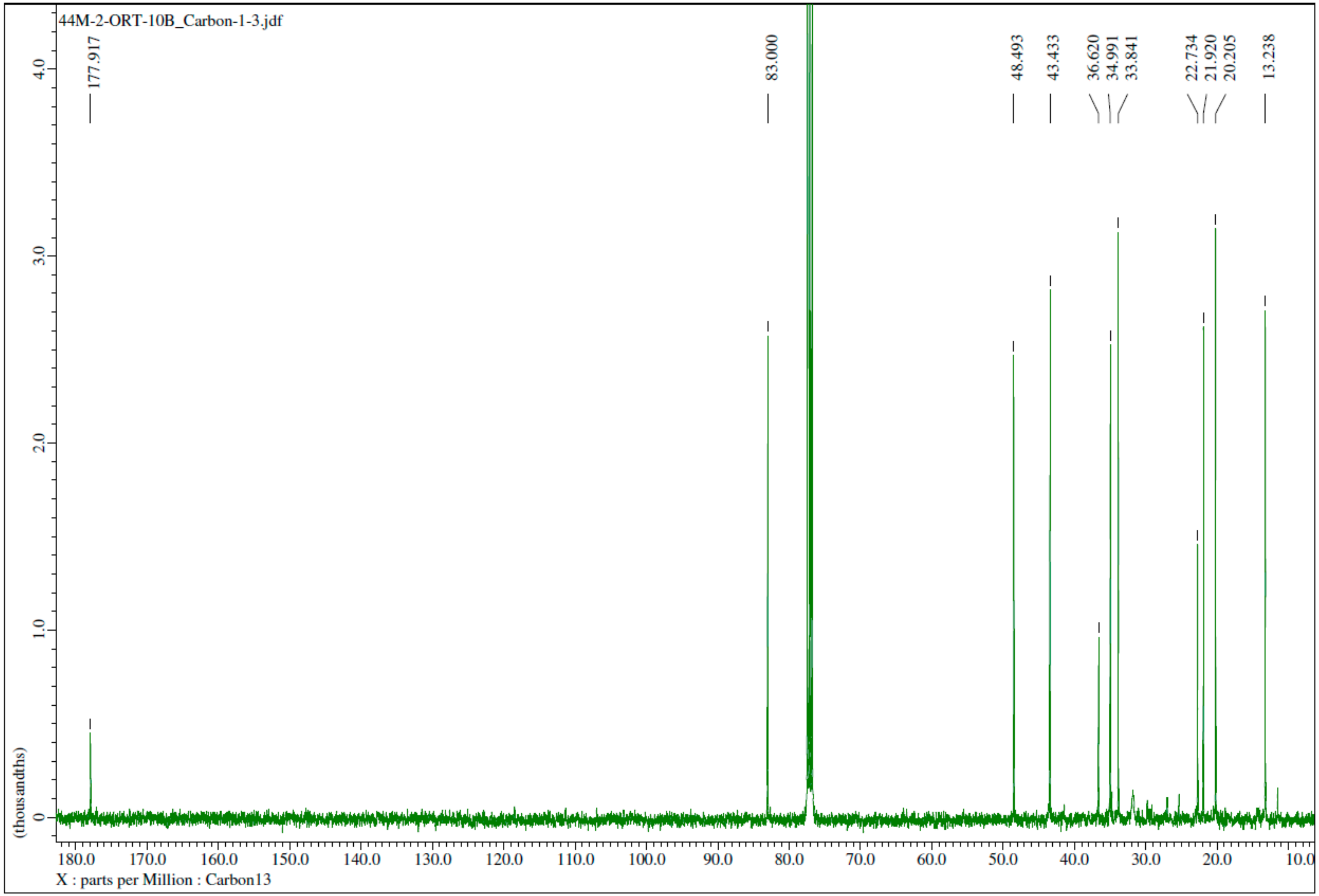

Figure S70. HMQC (100 MHz, $CDCl_3$) spectrum of chlorolactone **4b-B**

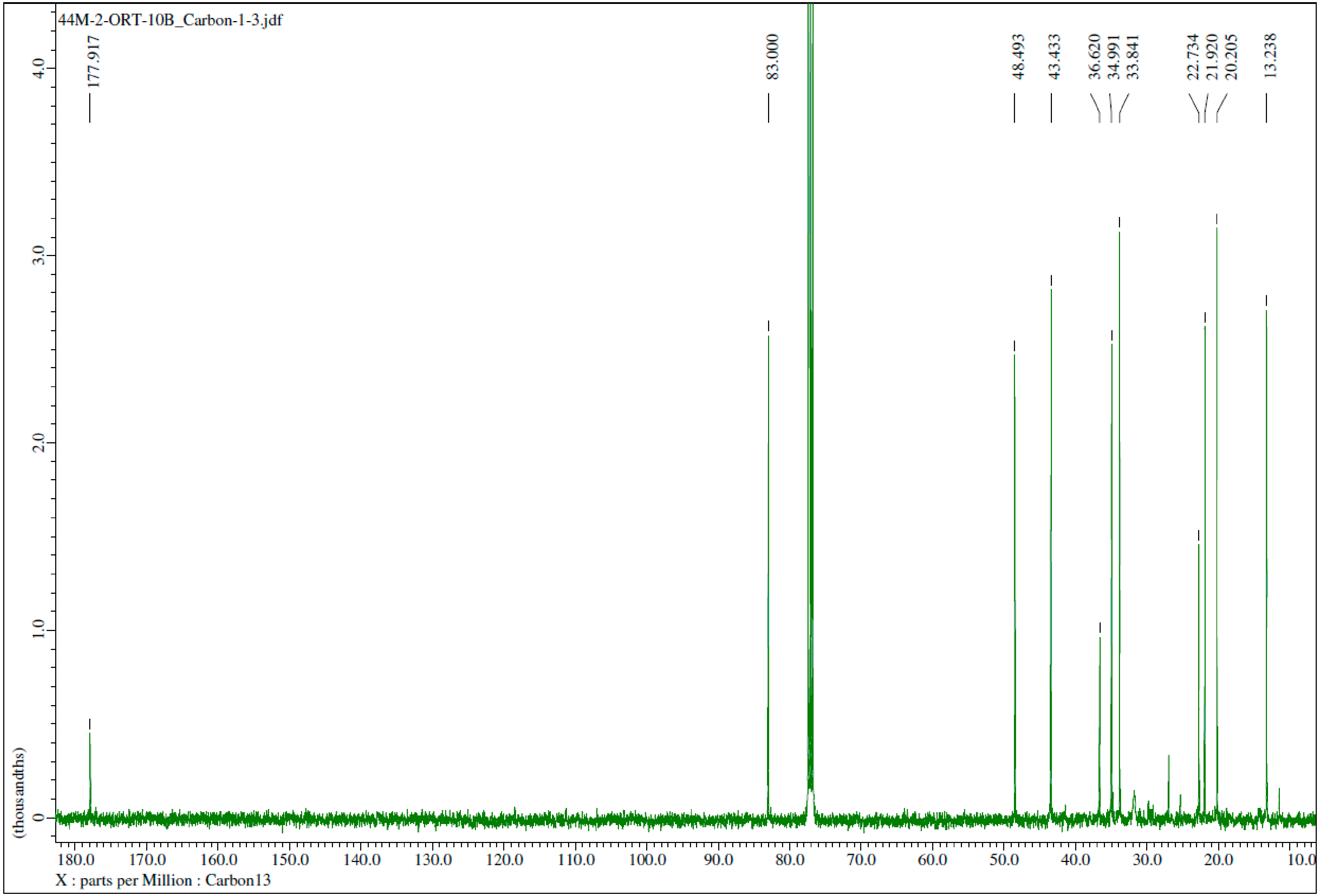

Figure S71. ^{13}C NMR (100 MHz, $CDCl_3$) spectrum of chlorolactone **4b-B**

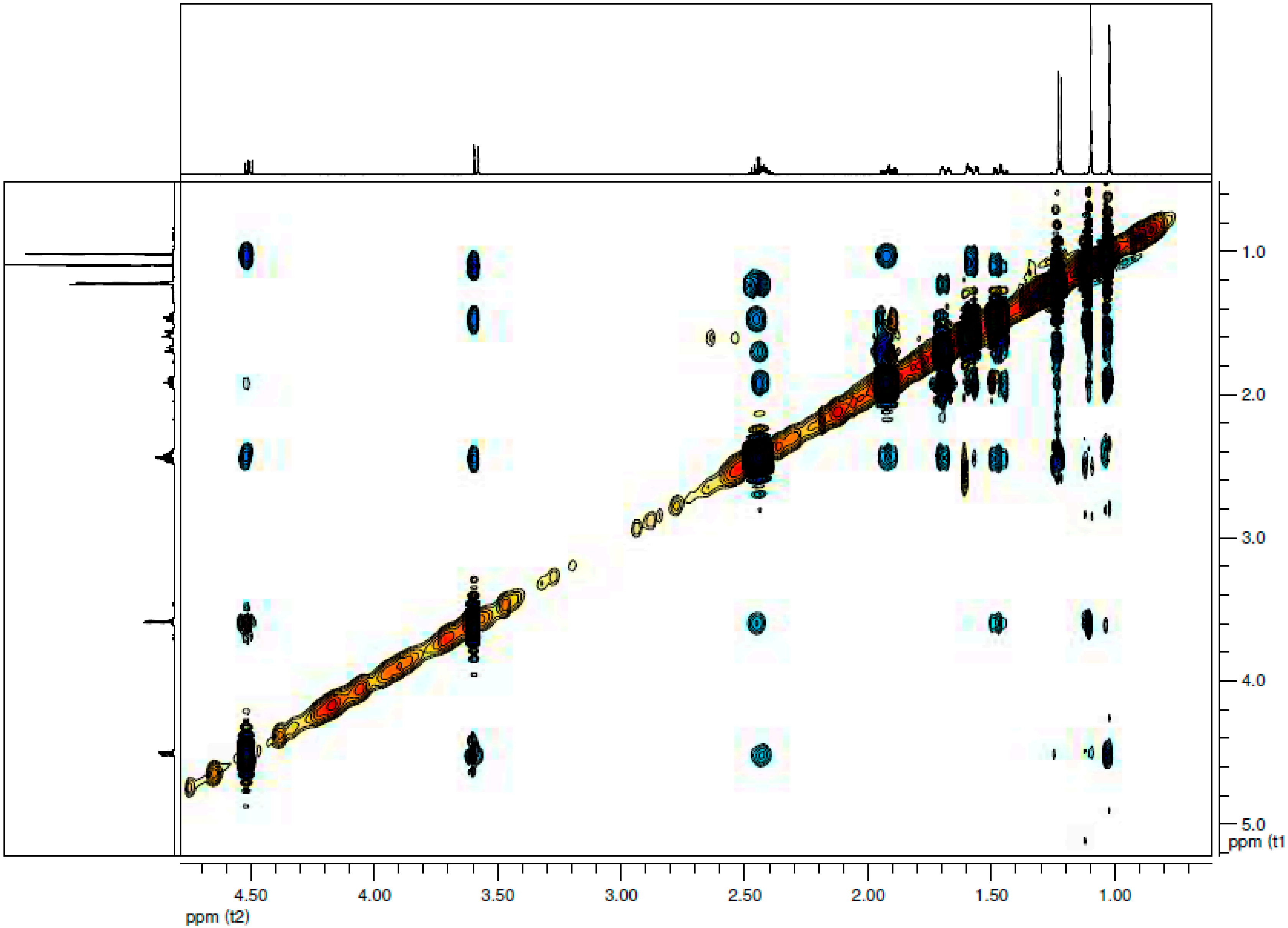

Figure S72.NOESY NMR (151 MHz, $CDCl_3$) spectrum of chlorolactone **4b-B**

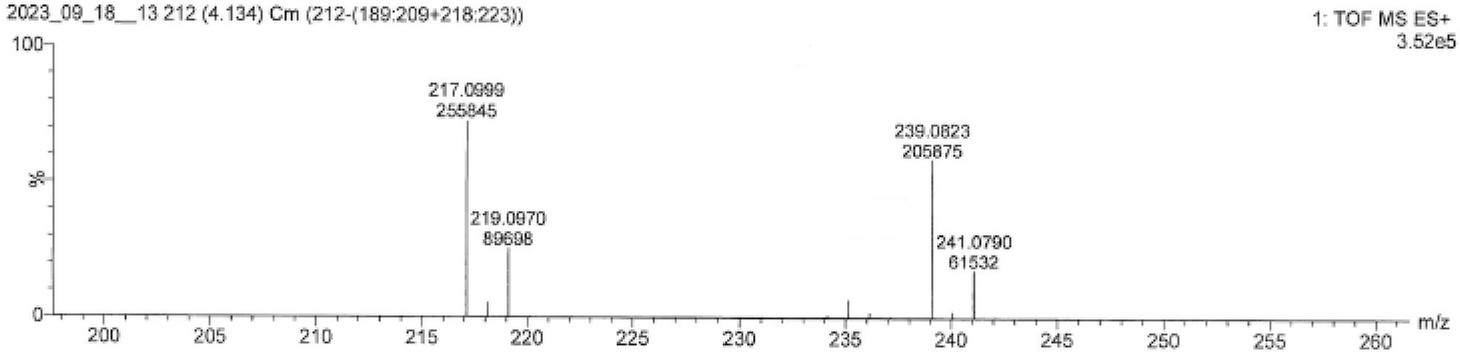

Figure S73. HRMS spectrum chlorolactone **4b**

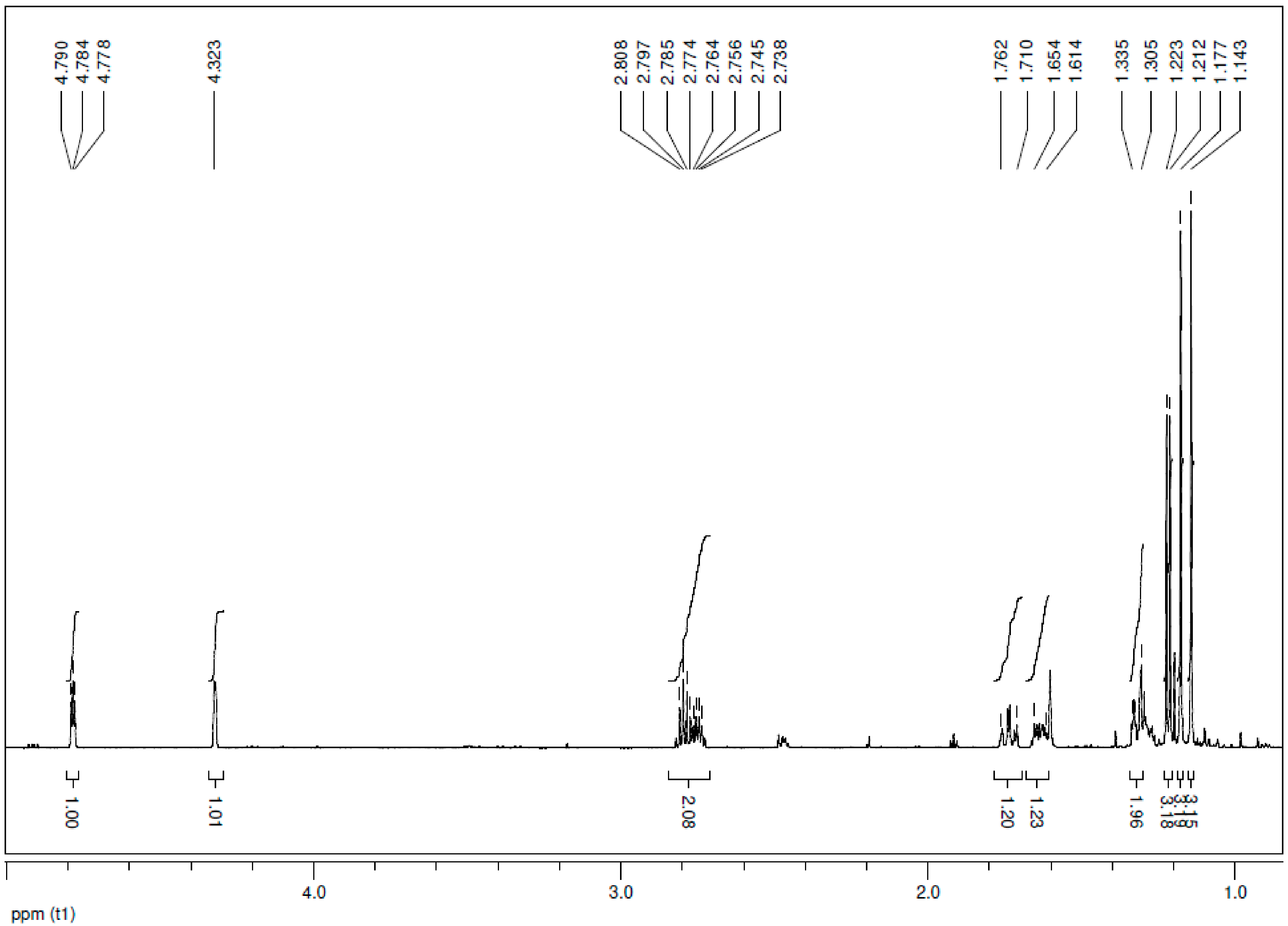

Figure S74. ^{1}H NMR (600 MHz, $CDCl_3$) spectrum of bromolactone **5b-A**

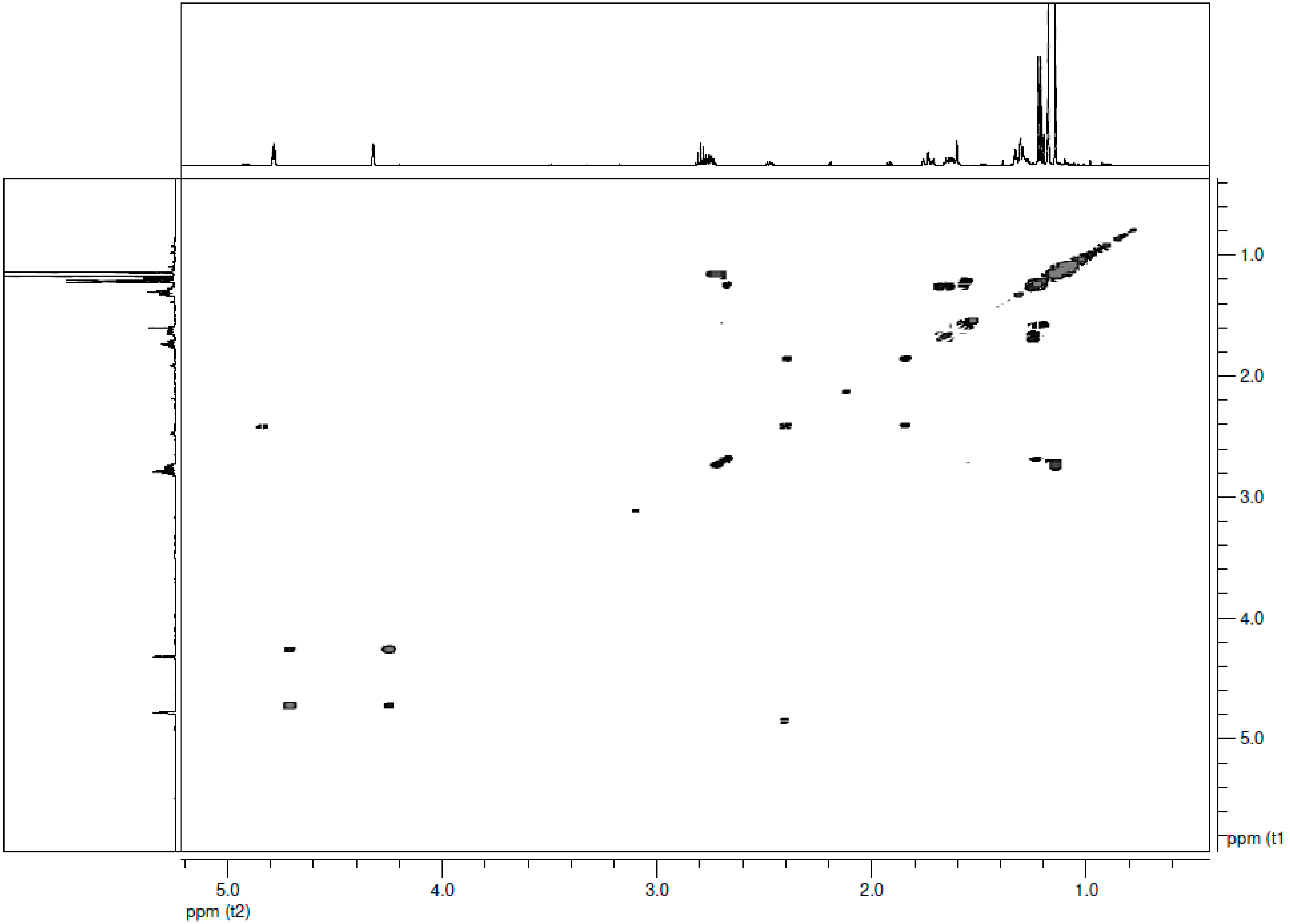

Figure S75. COSY (150 MHz, $CDCl_3$) spectrum of bromolactone **5b-A**

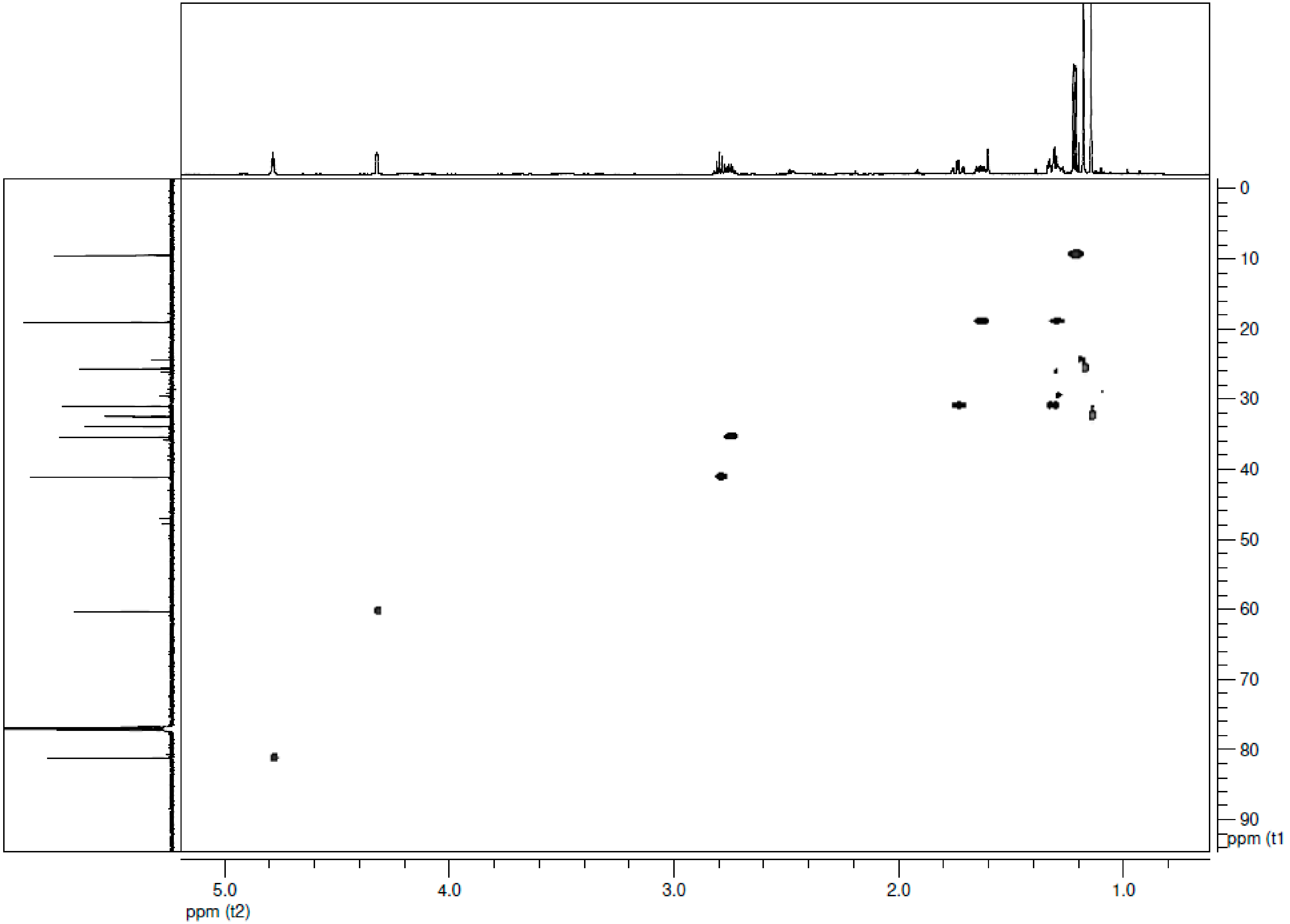

Figure S76. HMQC (150 MHz, $CDCl_3$) spectrum of bromolactone **5b-A**

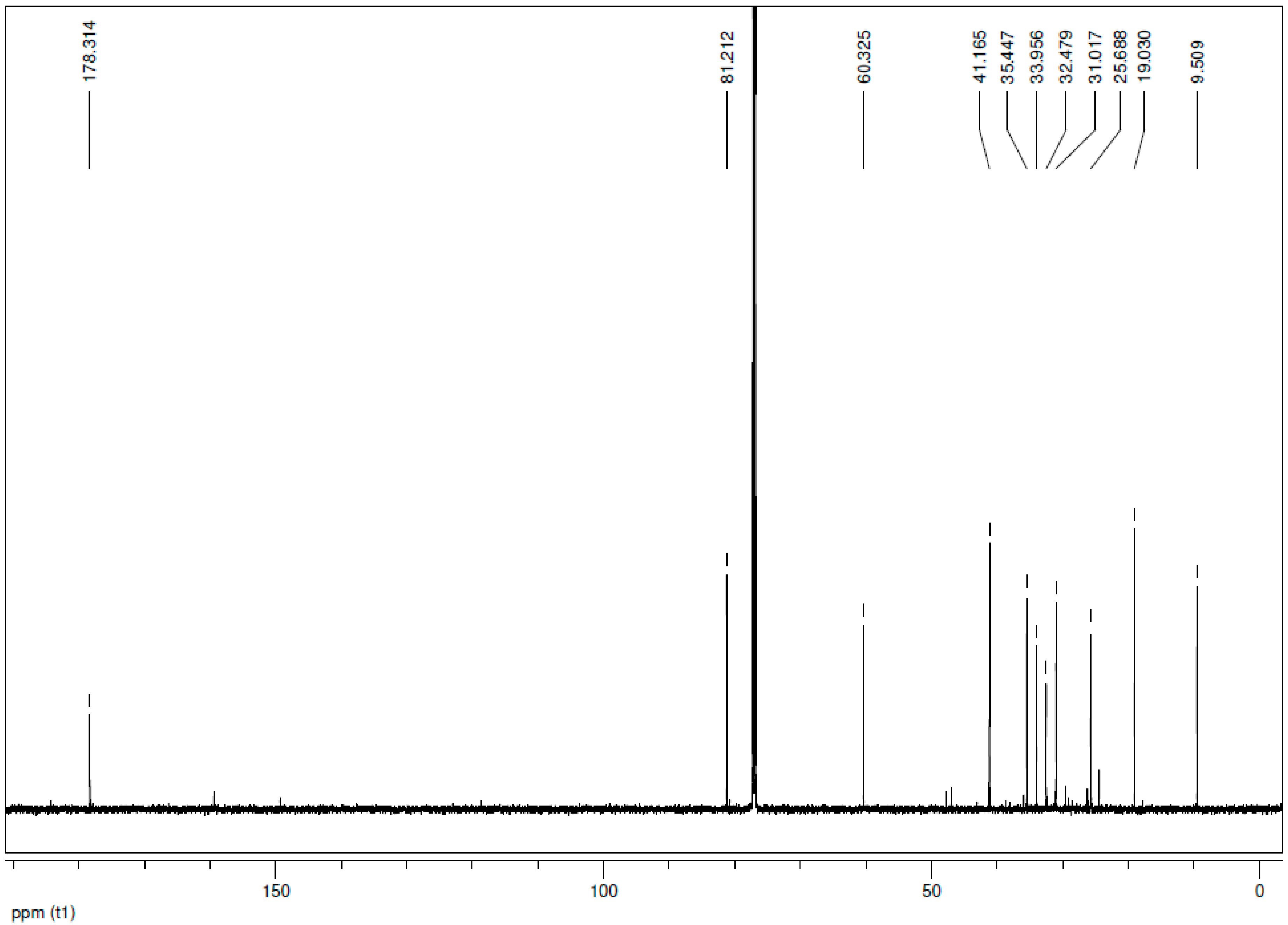

Figure S77. ^{13}C NMR (150 MHz, $CDCl_3$) spectrum bromolactone **5b-A**

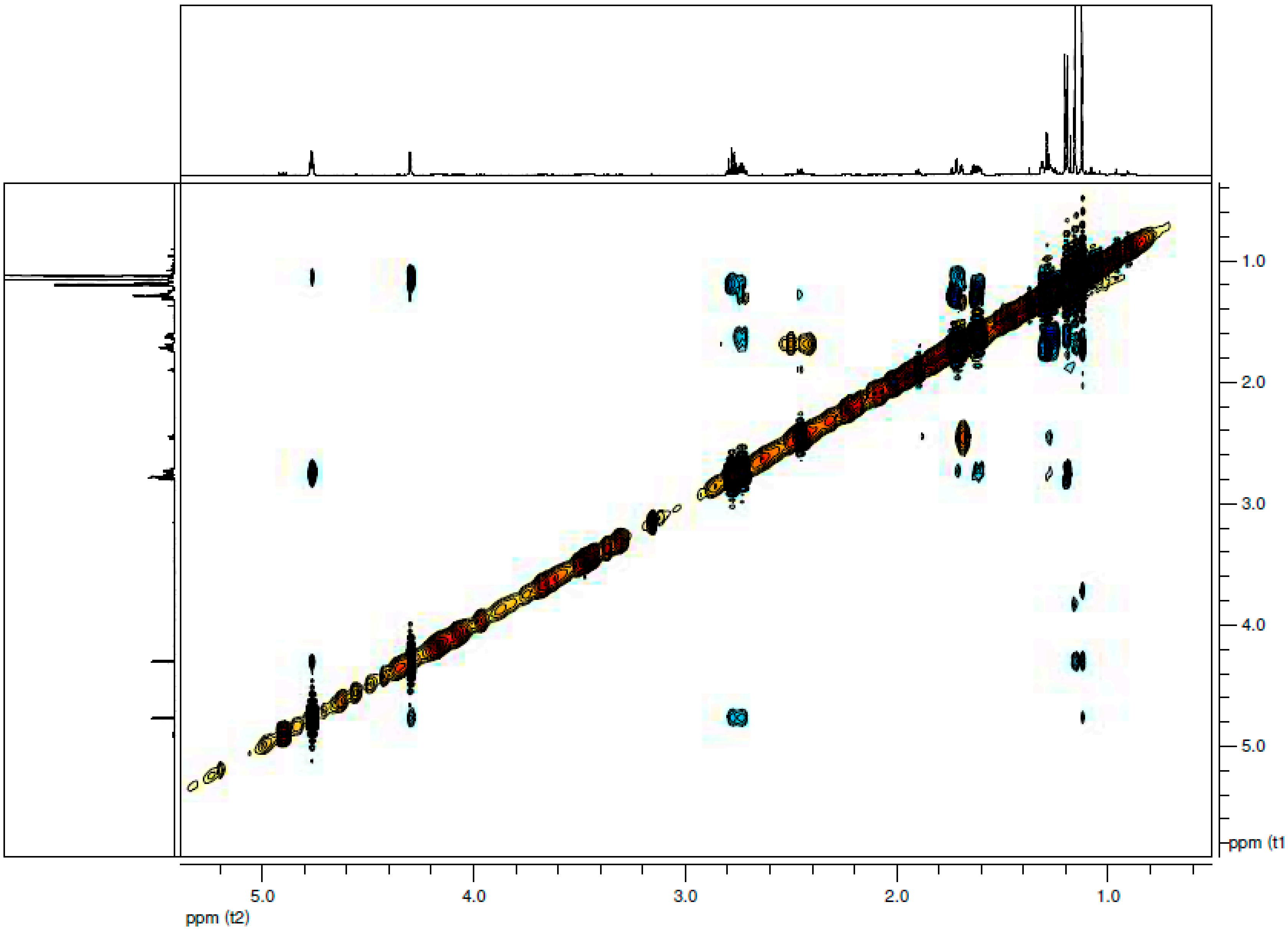

Figure S78.NOESY NMR (151 MHz, $CDCl_3$) spectrum bromolactone **5b-A**

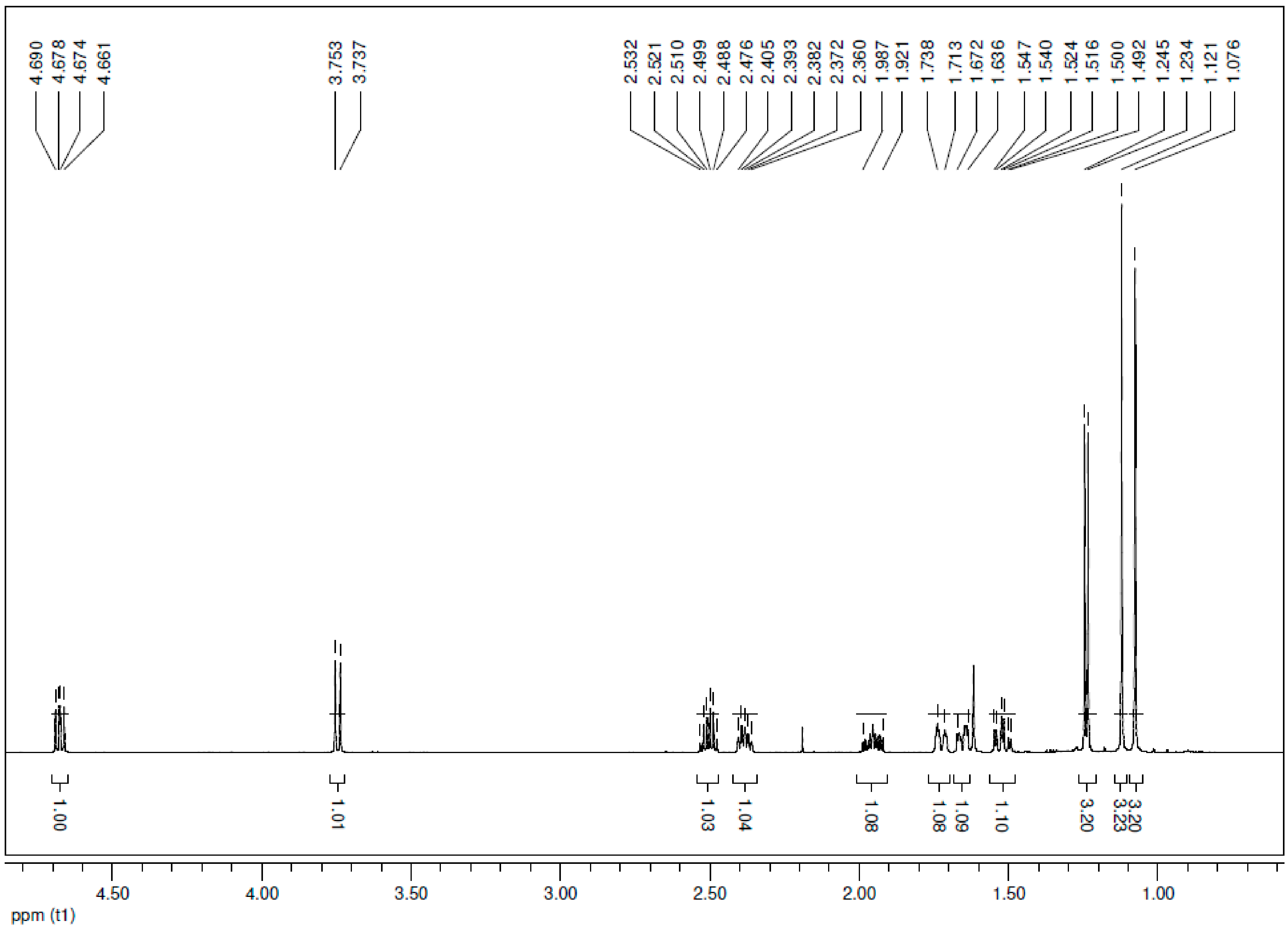

Figure S79. ^{1}H NMR (600 MHz, $CDCl_3$) spectrum of bromolactone **5b-B**

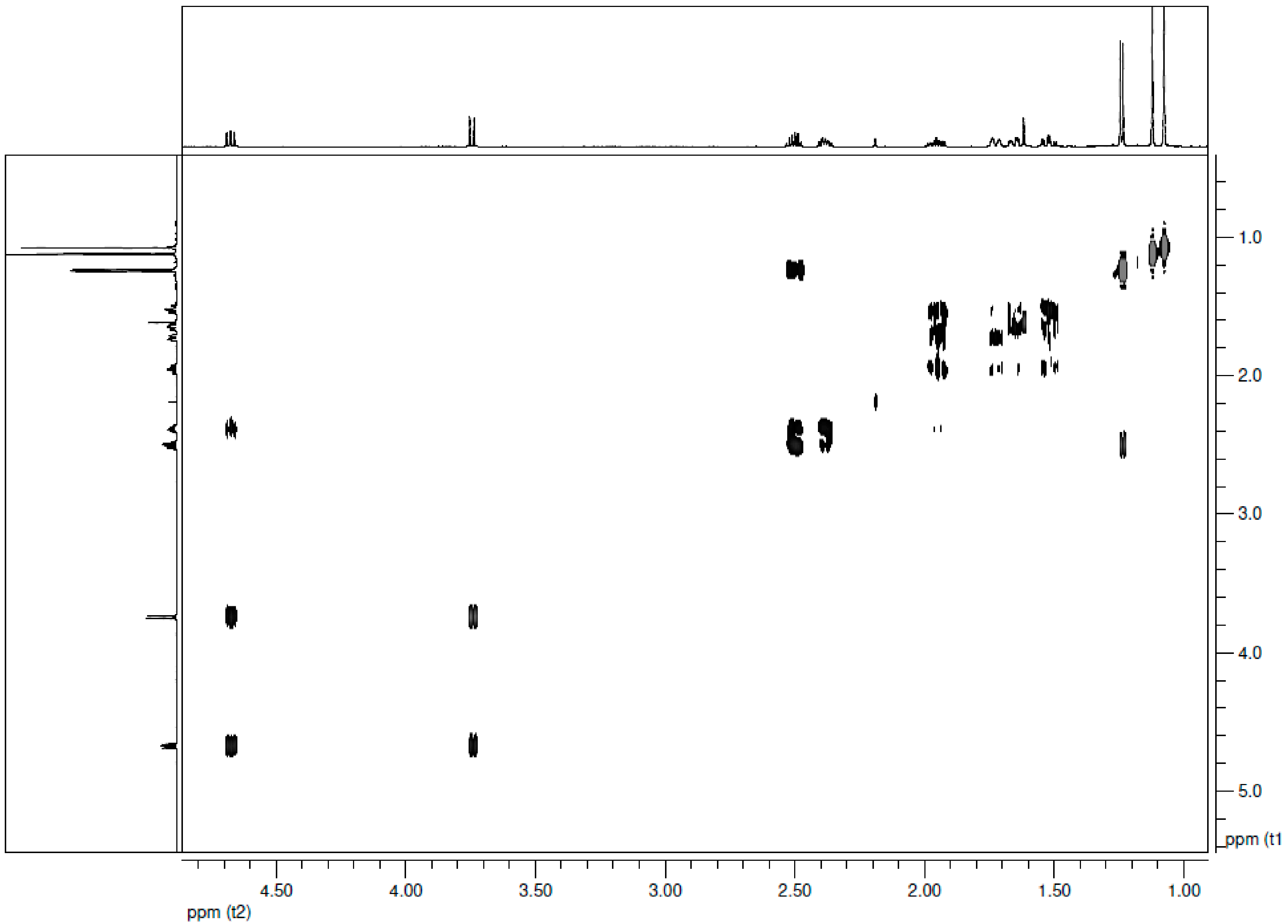

Figure S80. COSY (150 MHz, $CDCl_3$) spectrum of bromolactone **5b-B**

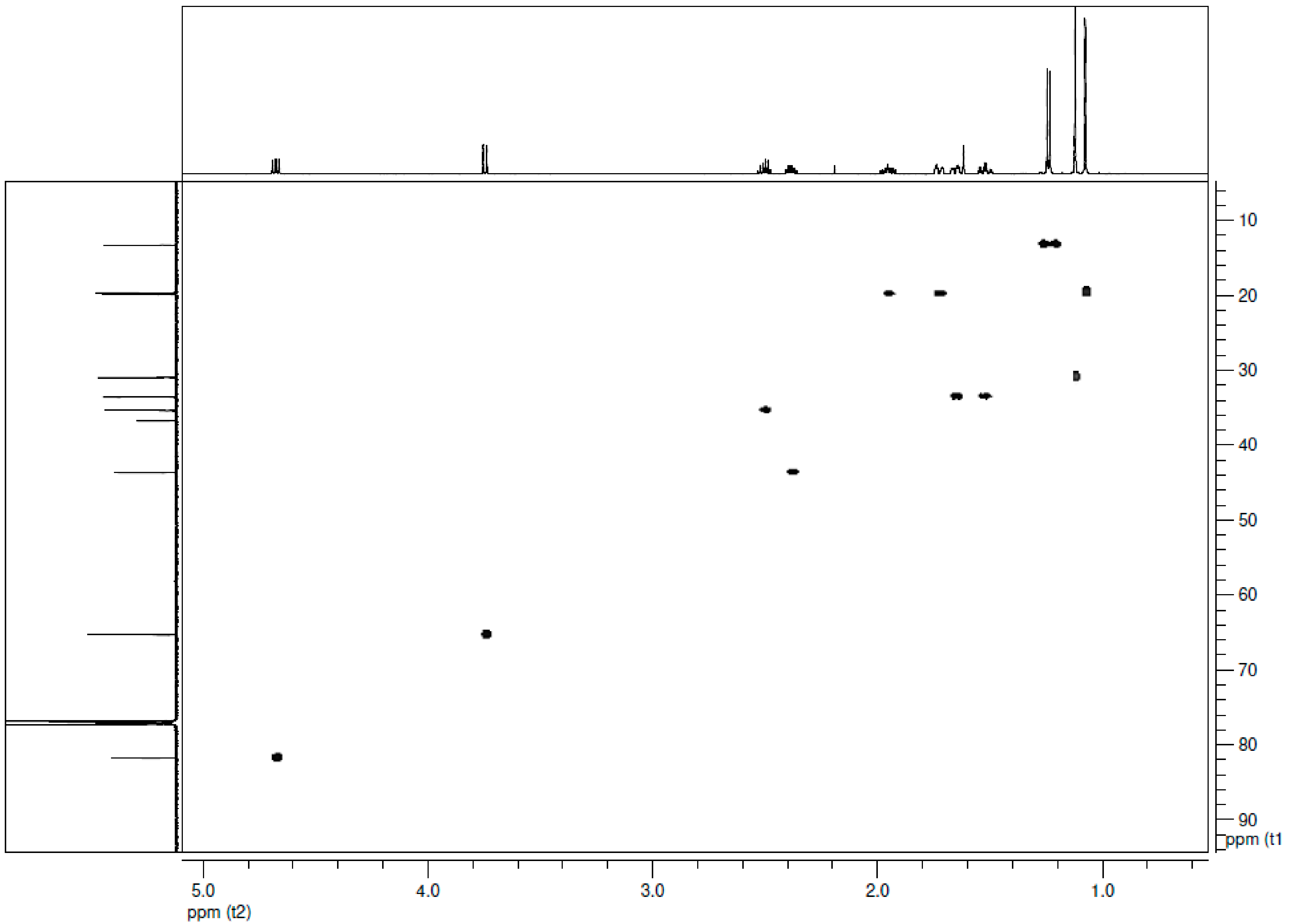

Figure S81. HMQC (150 MHz, $CDCl_3$) spectrum of bromolactone **5b-B**

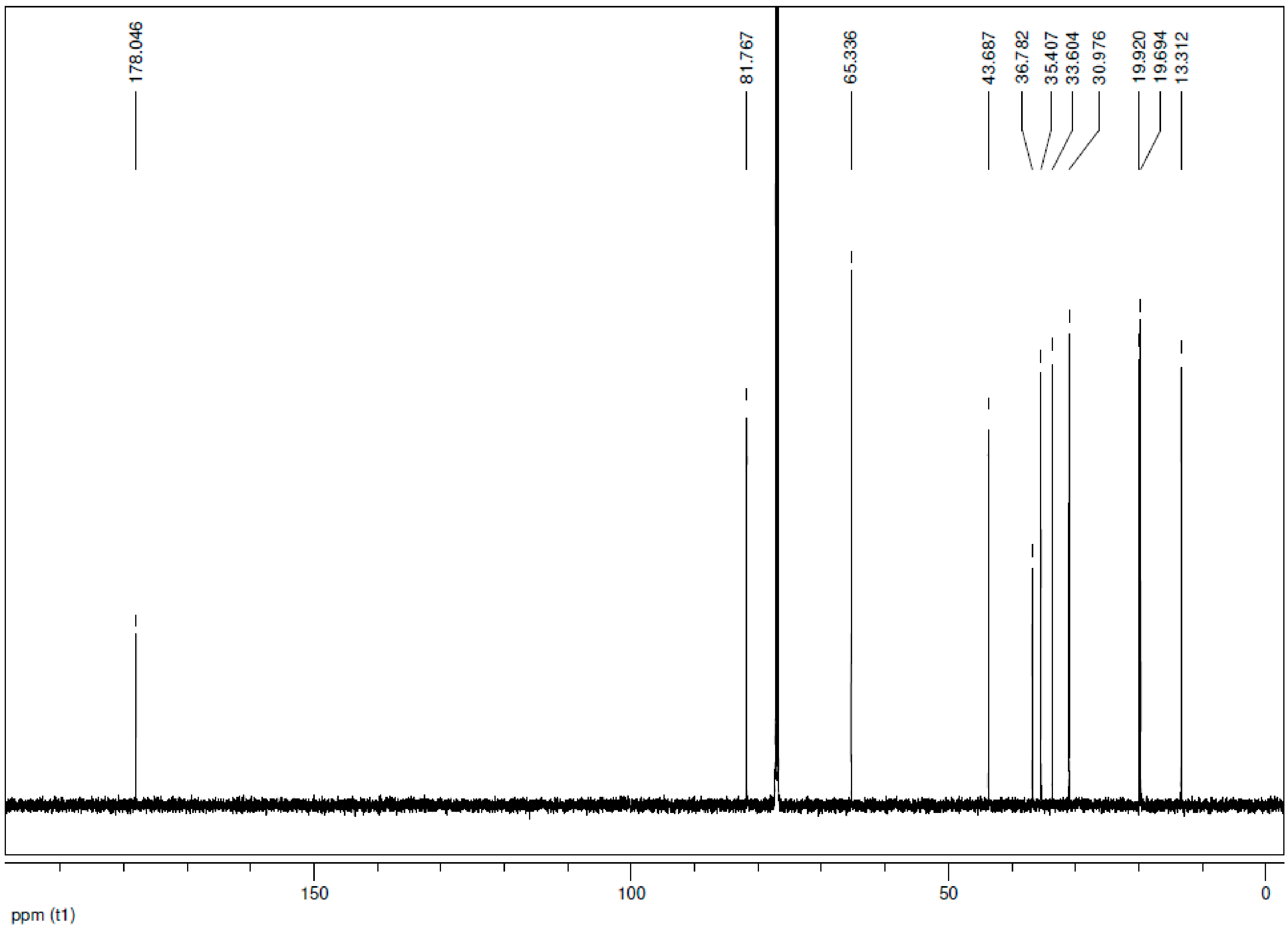

Figure S82. ^{13}C NMR (150 MHz, $CDCl_3$) spectrum bromolactone **5b-B**

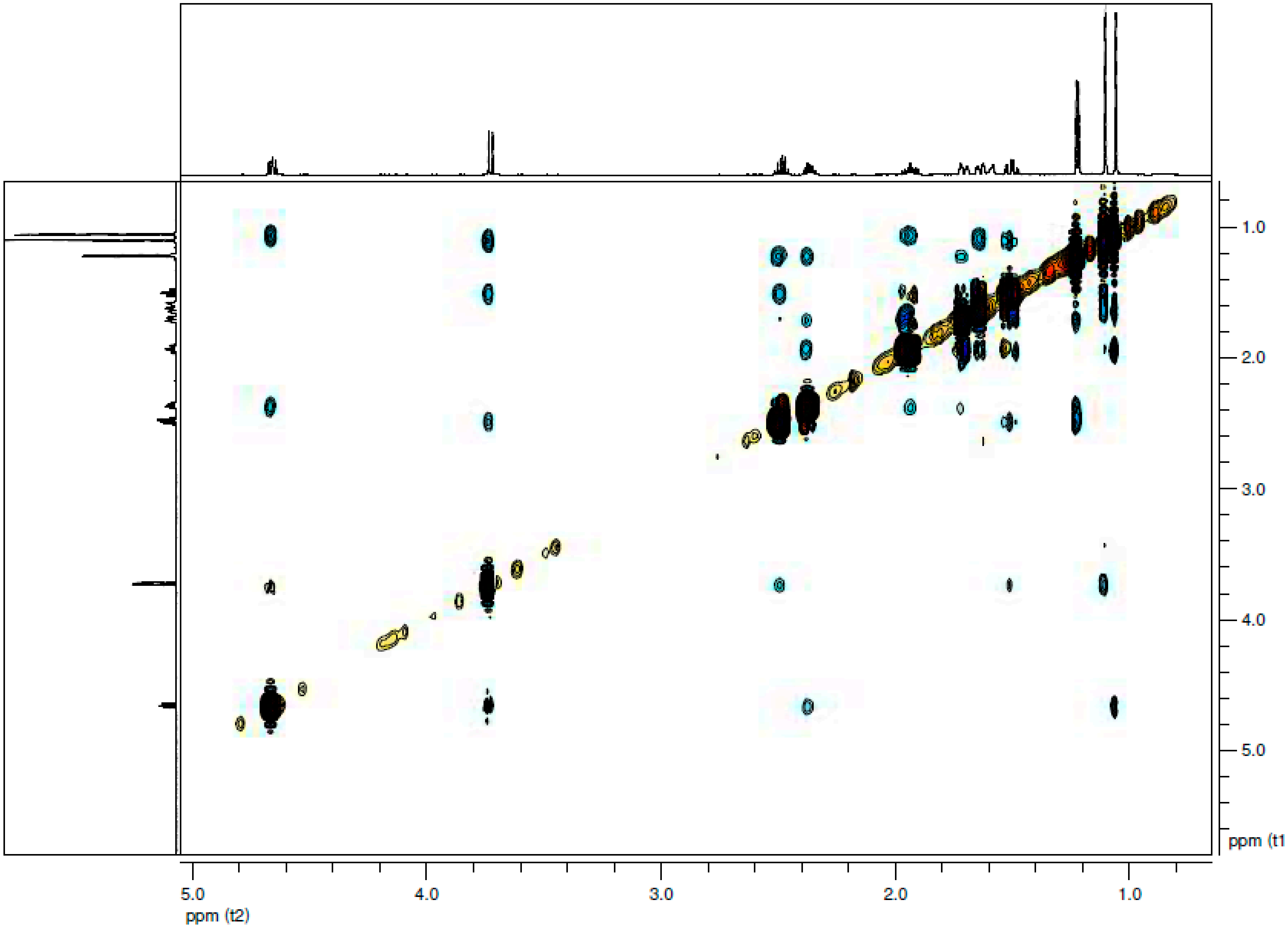

Figure S83.NOESY NMR (151 MHz, $CDCl_3$) spectrum bromolactone **5b-B**

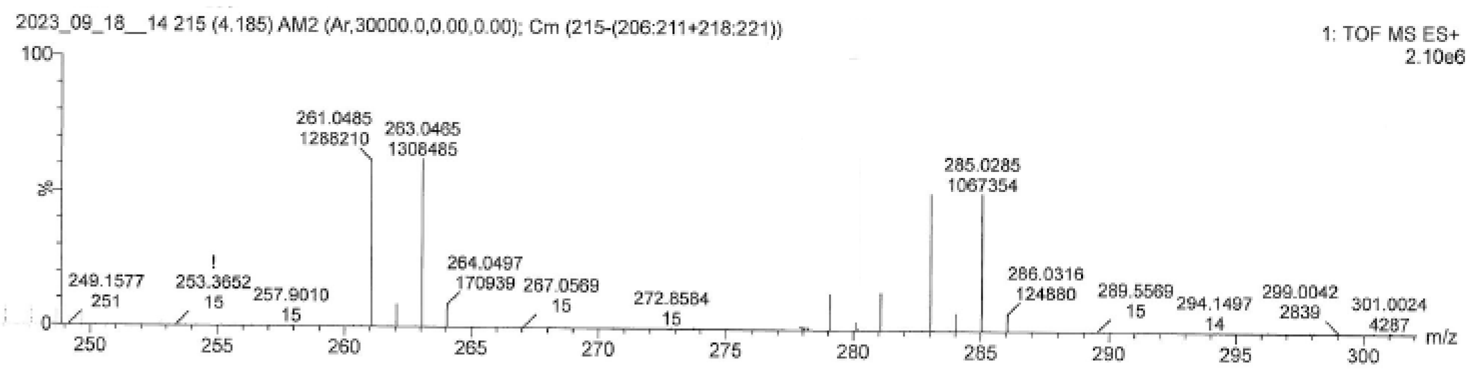

Figure S84. HRMS spectrum bromolactone **5b**

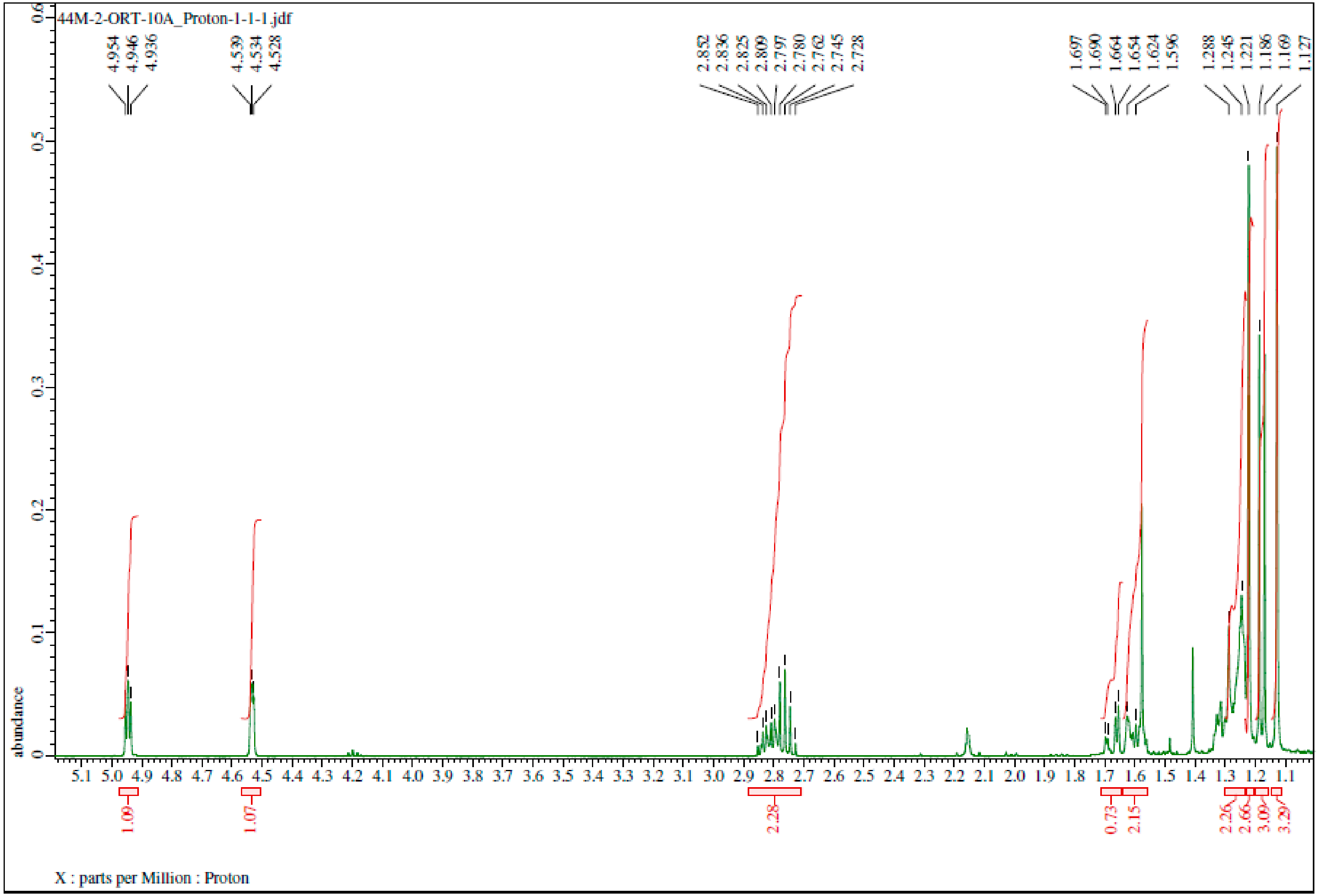

Figure S85. ^{1}H NMR (400 MHz, $CDCl_3$) spectrum of iodolactone **6b-A**

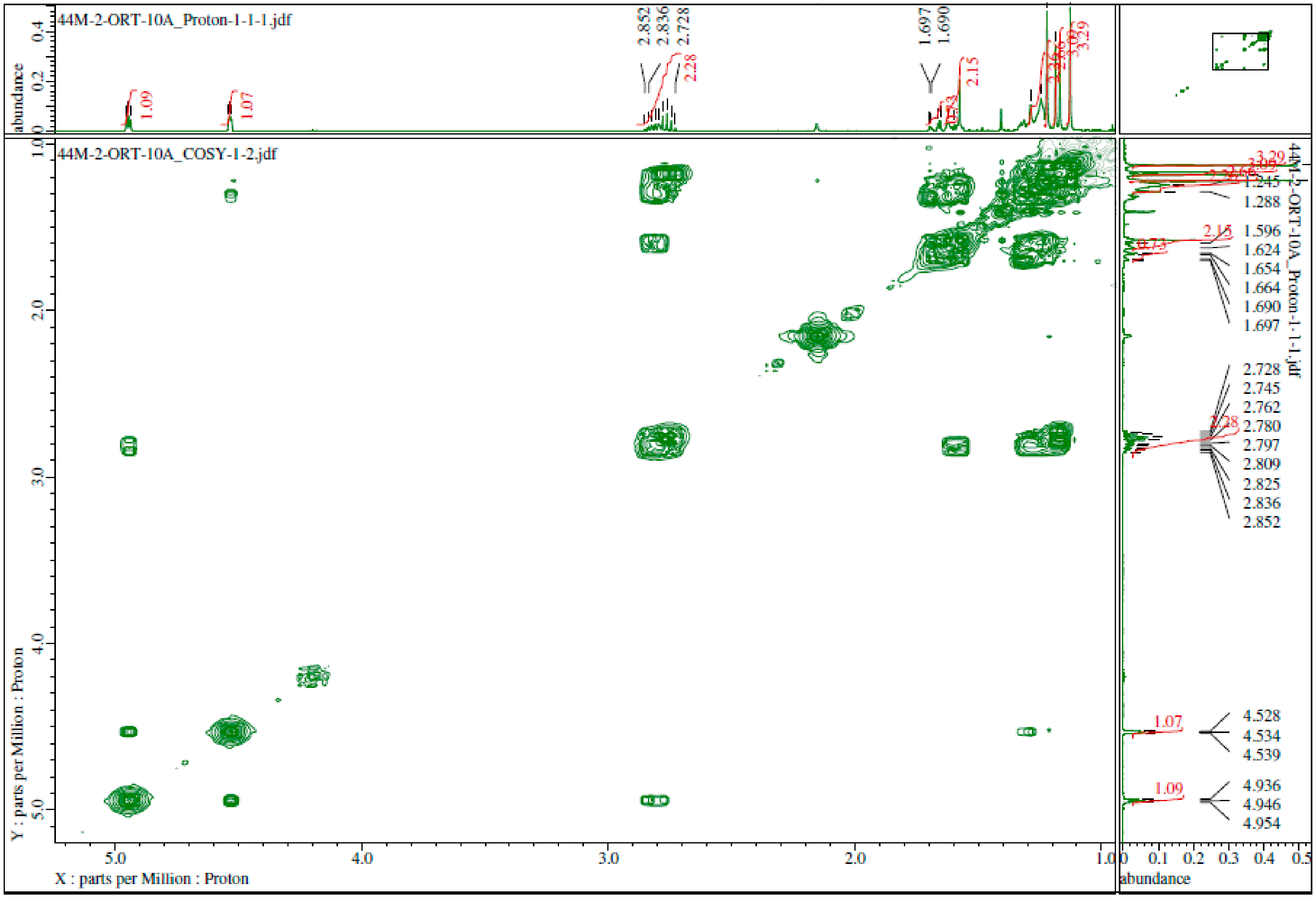

Figure S86. COSY (100 MHz, $CDCl_3$) spectrum of iodolactone **6b-A**

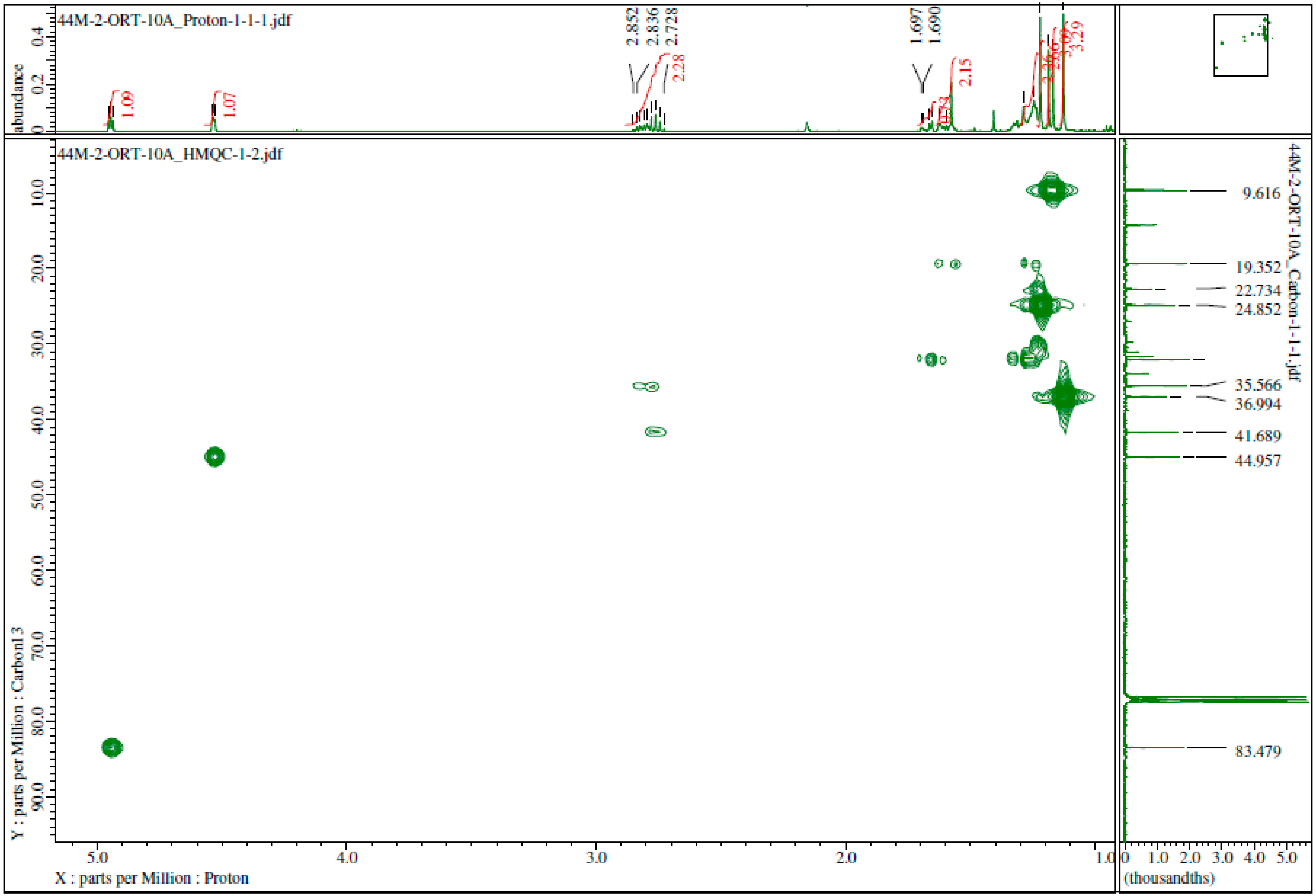

Figure S87. HMQC (100 MHz, $CDCl_3$) spectrum of iodolactone **6b-A**

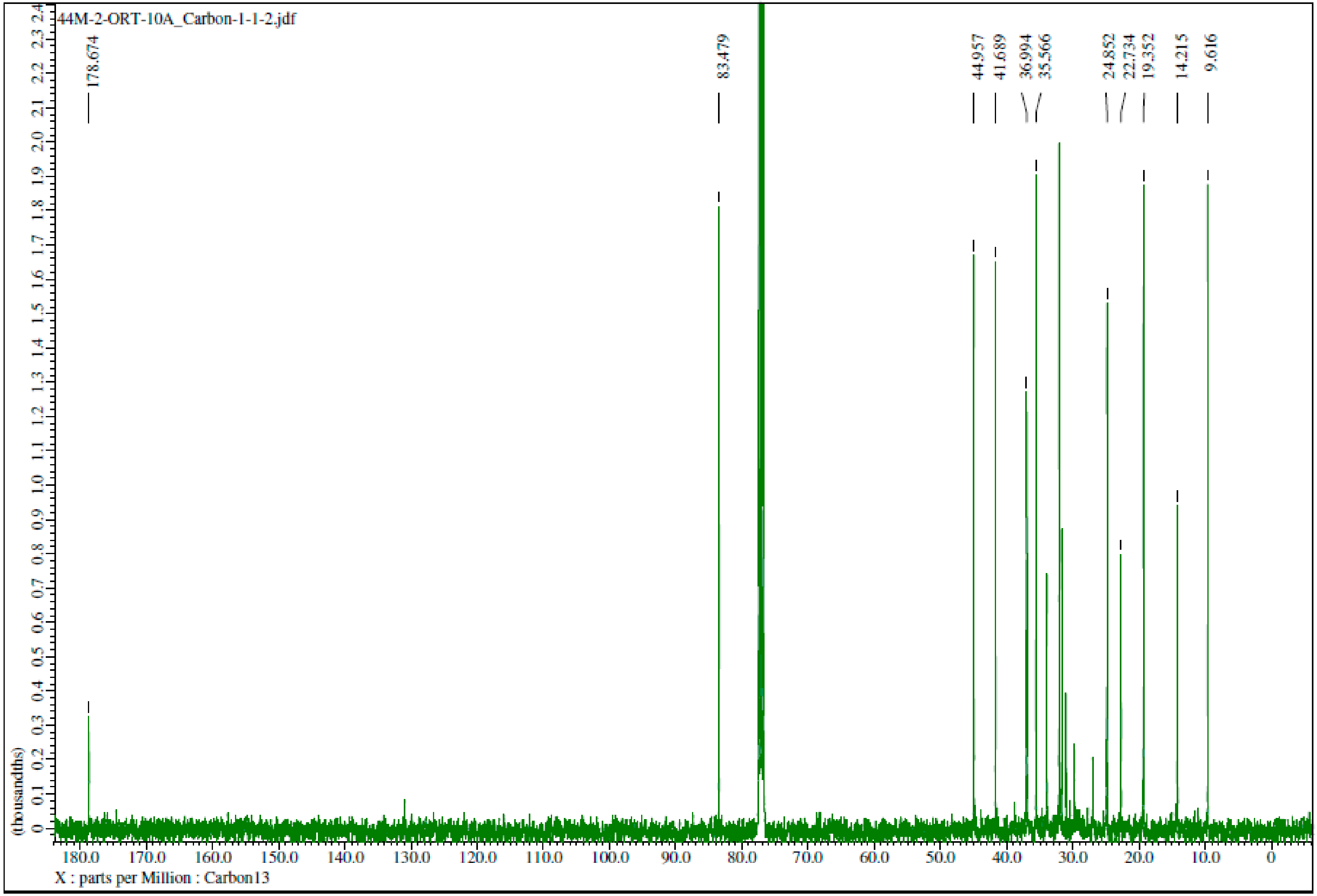

Figure S88. ^{13}C NMR (100 MHz, $CDCl_3$) spectrum of iodolactone **6b-A**

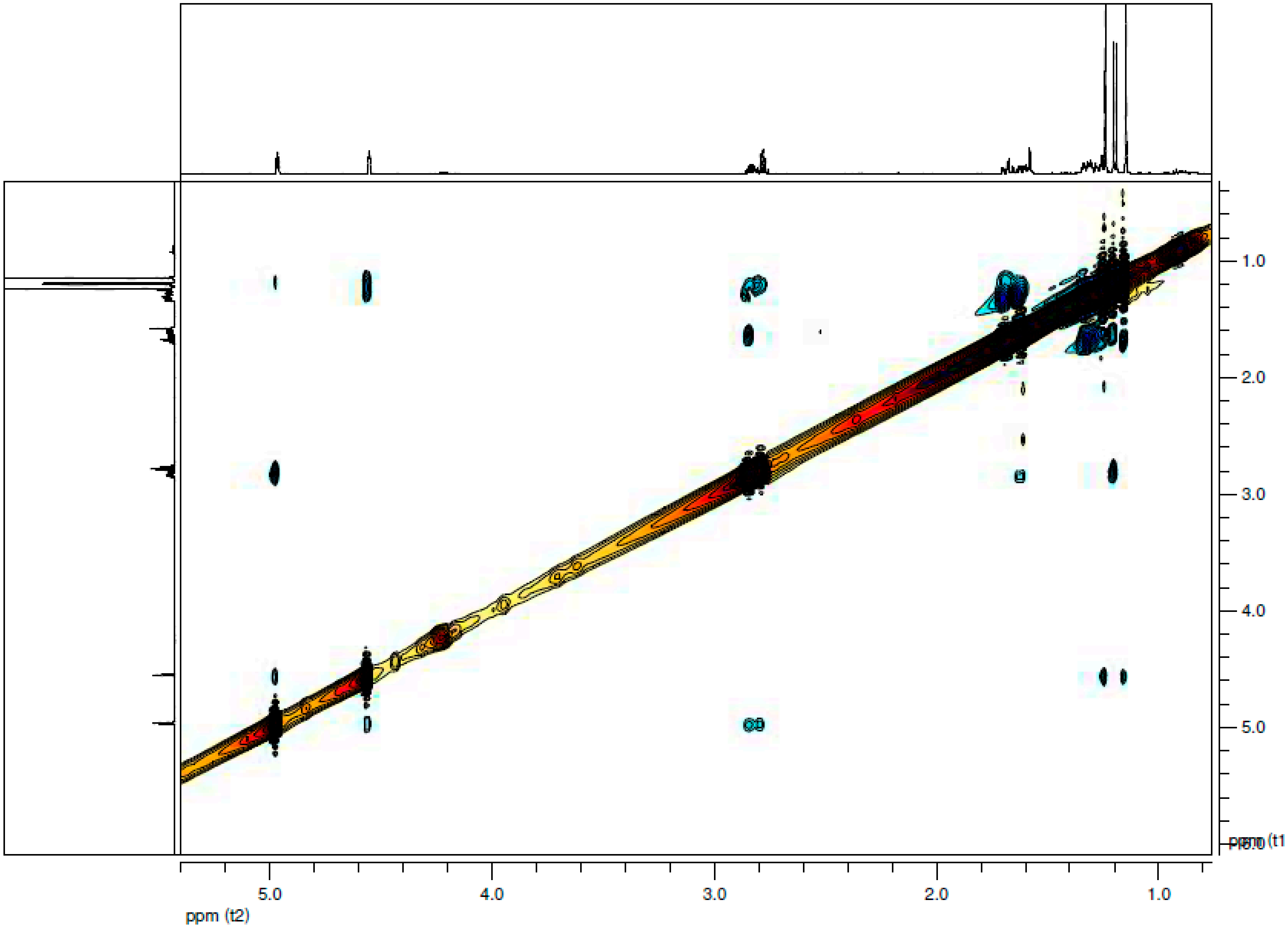

Figure S89.NOESY NMR (151 MHz, $CDCl_3$) spectrum iodolactone **6b-A**

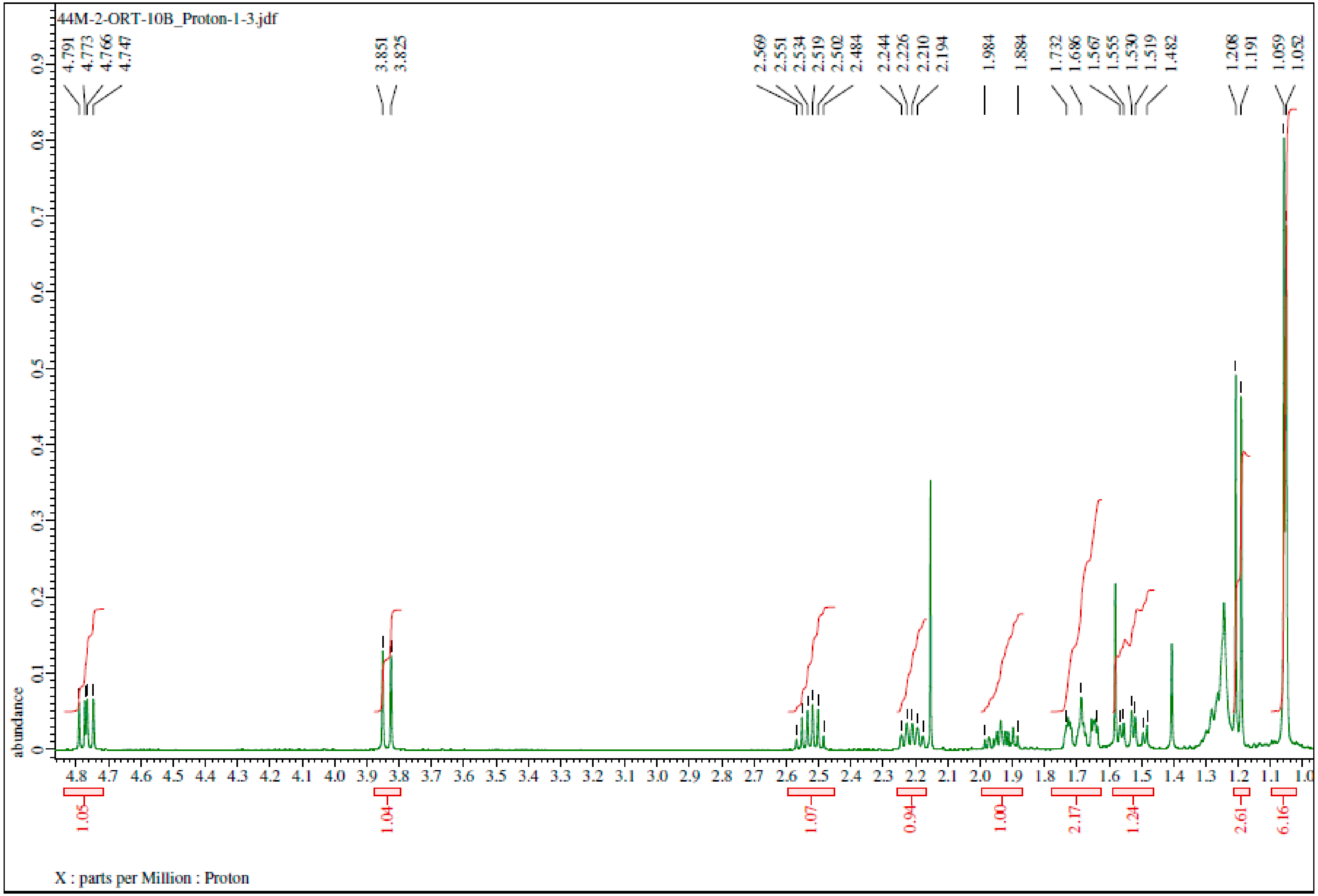

Figure S90. ^{1}H NMR (400 MHz, $CDCl_3$) spectrum of iodolactone **6b-B**

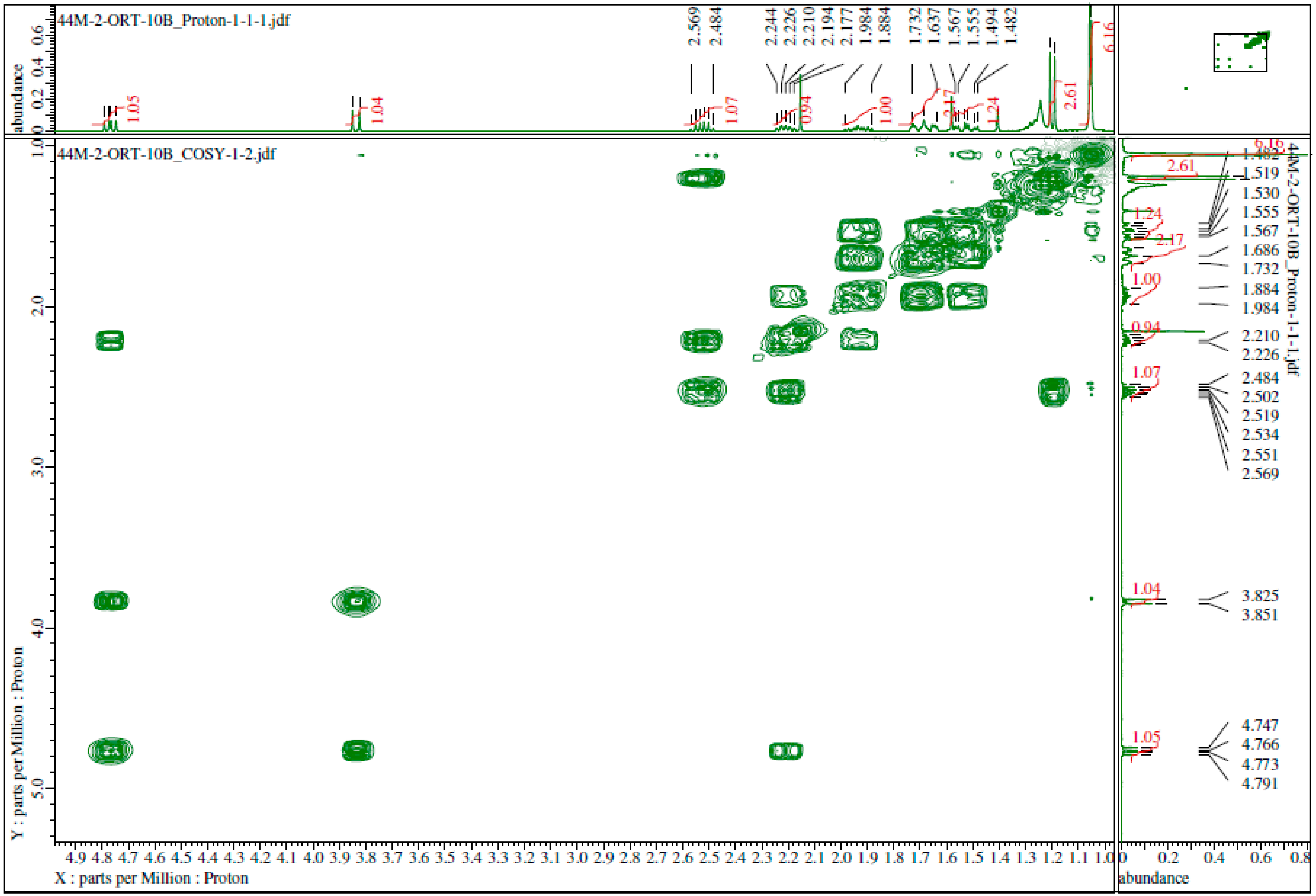

Figure S91. COSY (100 MHz, CDCl3) spectrum of iodolactone **6b-B**

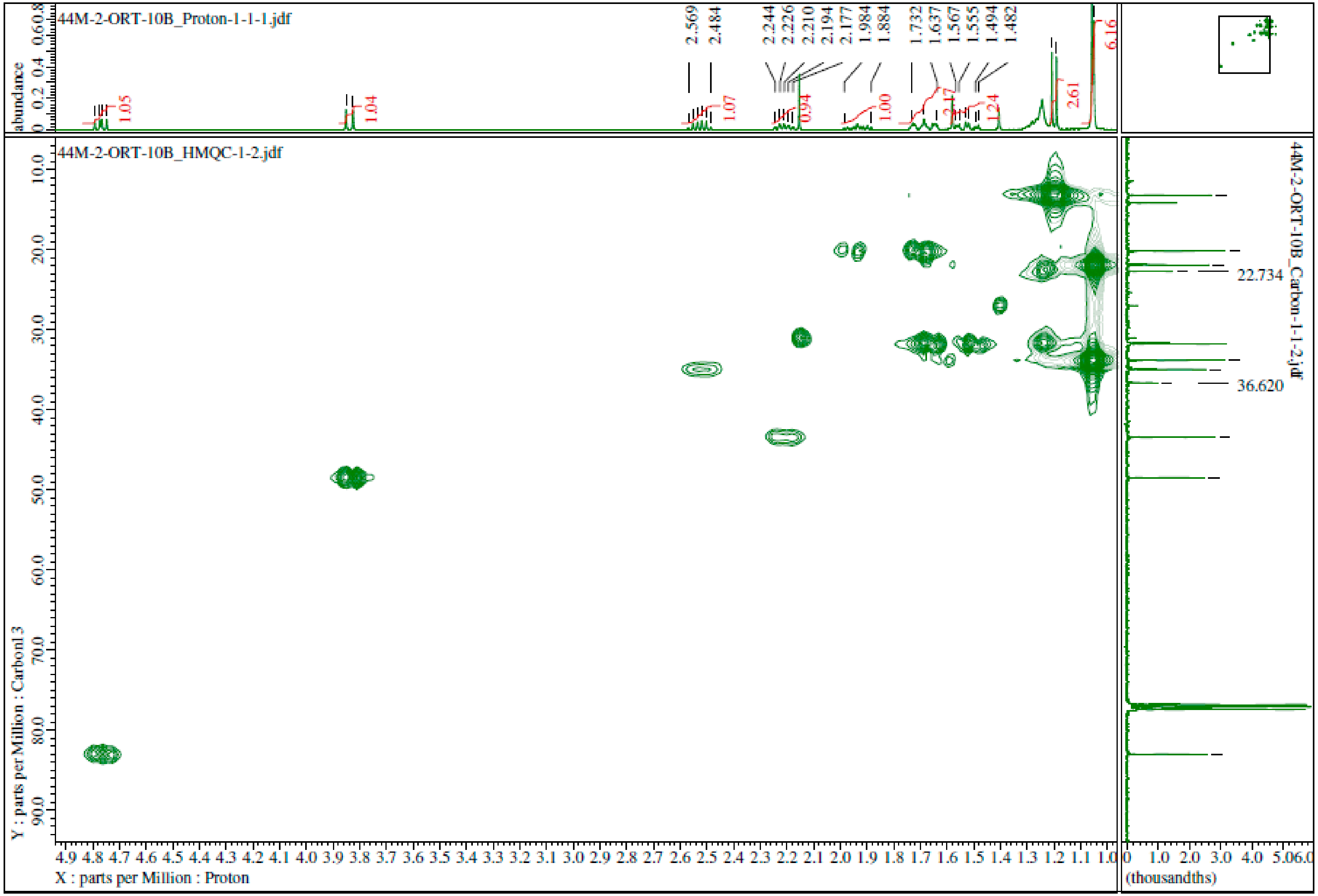

Figure S92. HMQC (100 MHz, $CDCl_3$) spectrum of iodolactone **6b-B**

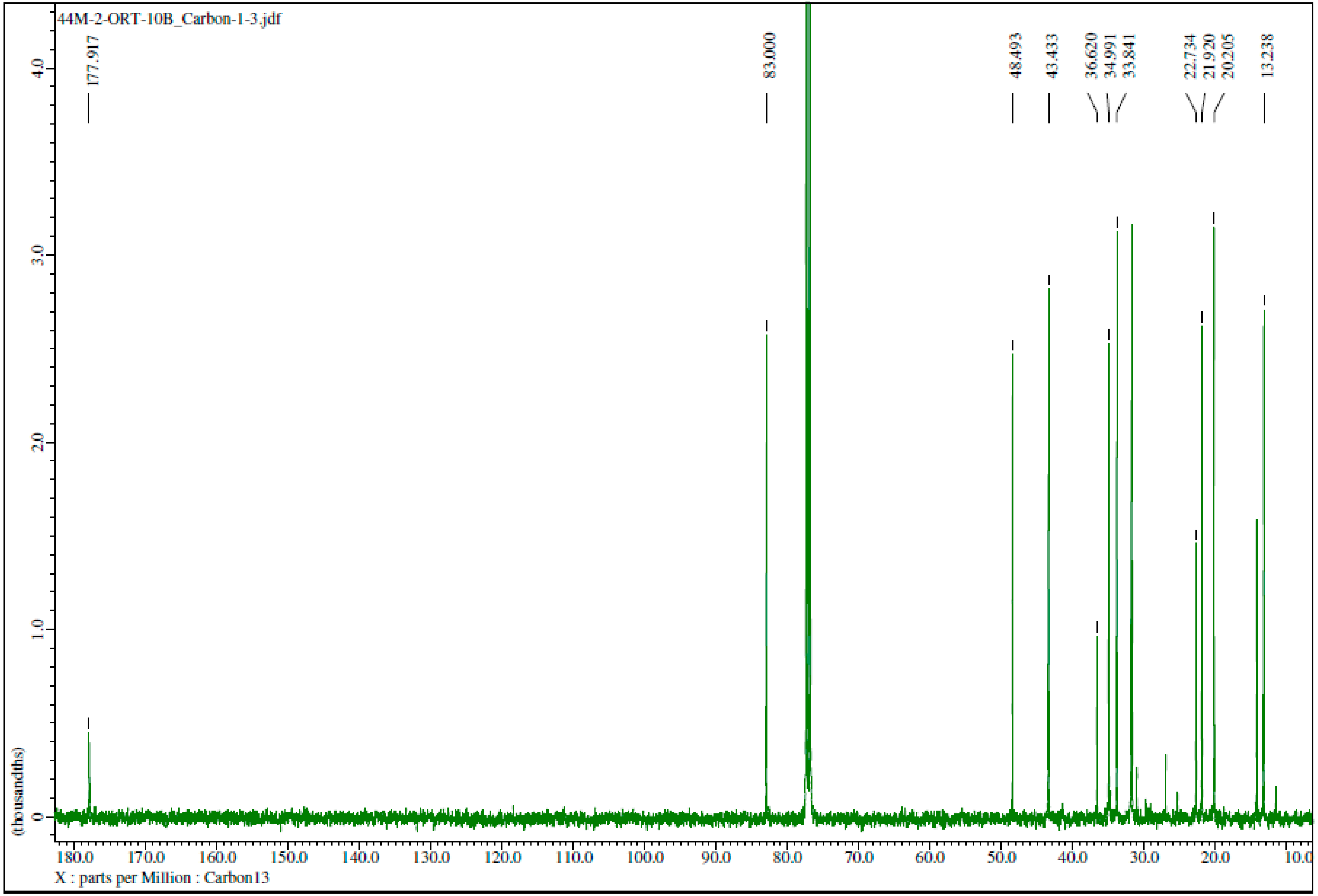

Figure S93. ^{13}C NMR (100 MHz, $CDCl_3$) spectrum of iodolactone **6b-B**

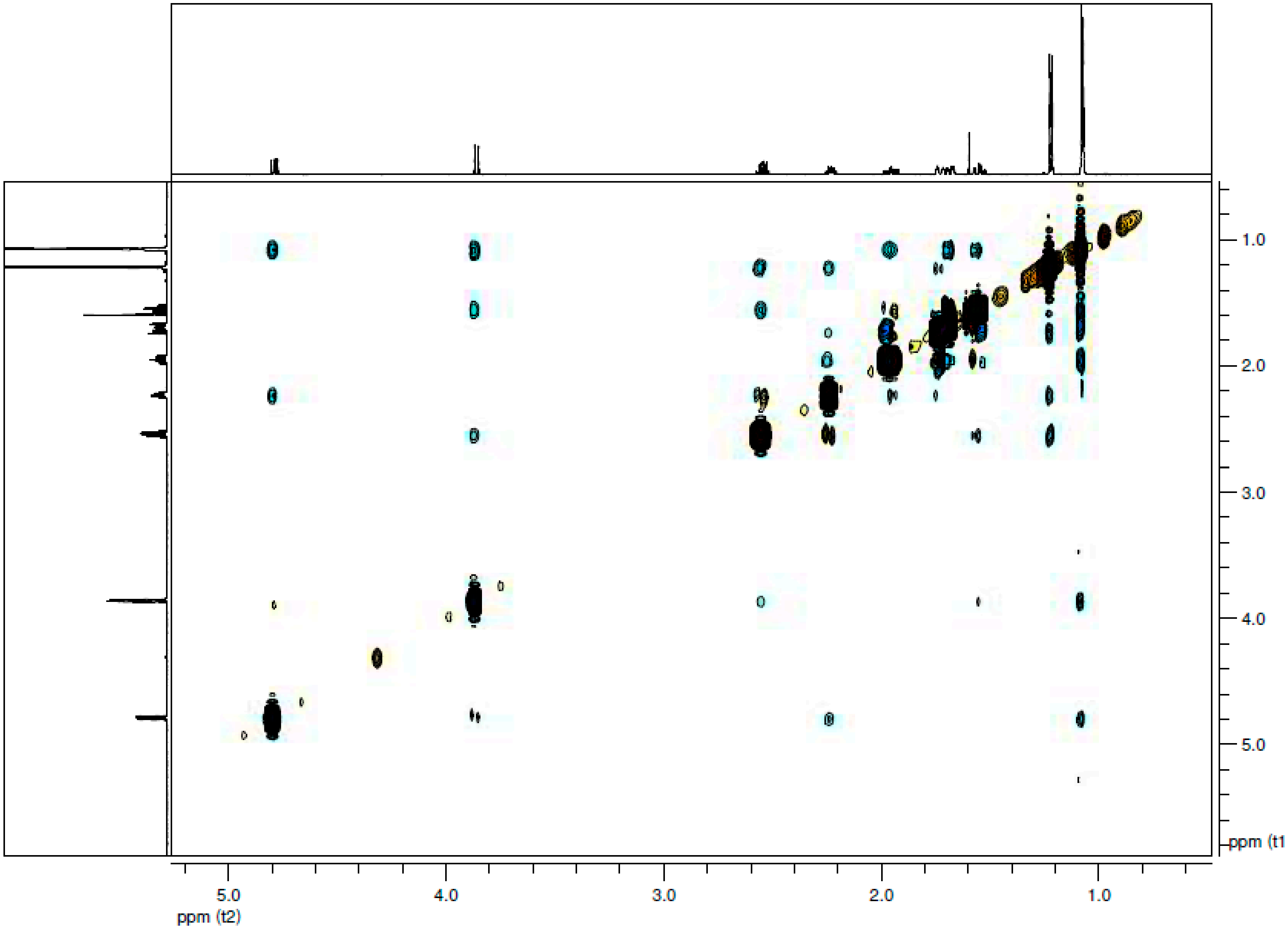

Figure S94.NOESY NMR (151 MHz, CDCl$_3$) spectrum iodolactone **6b-B**

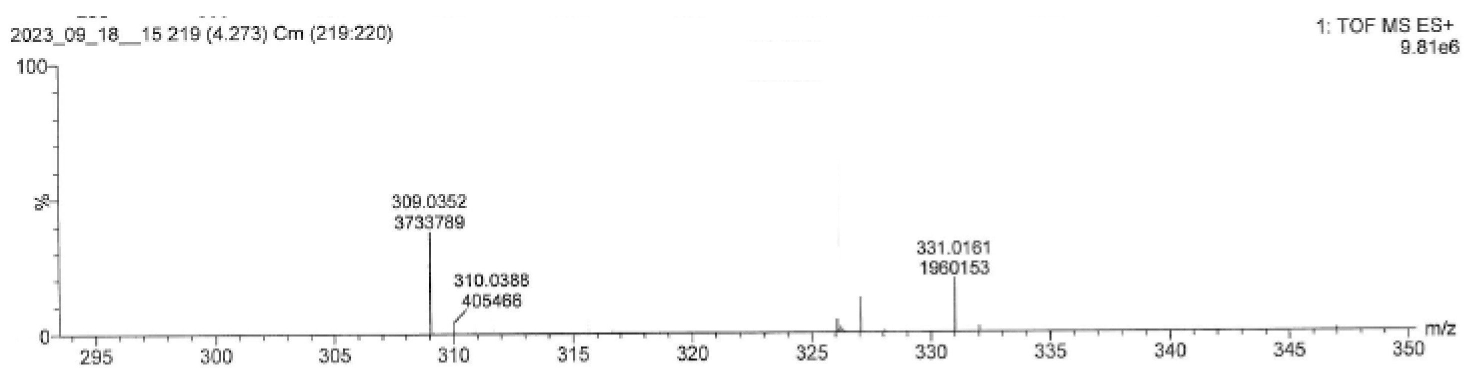

Figure S95. HRMS spectrum of iodolactone **6b**

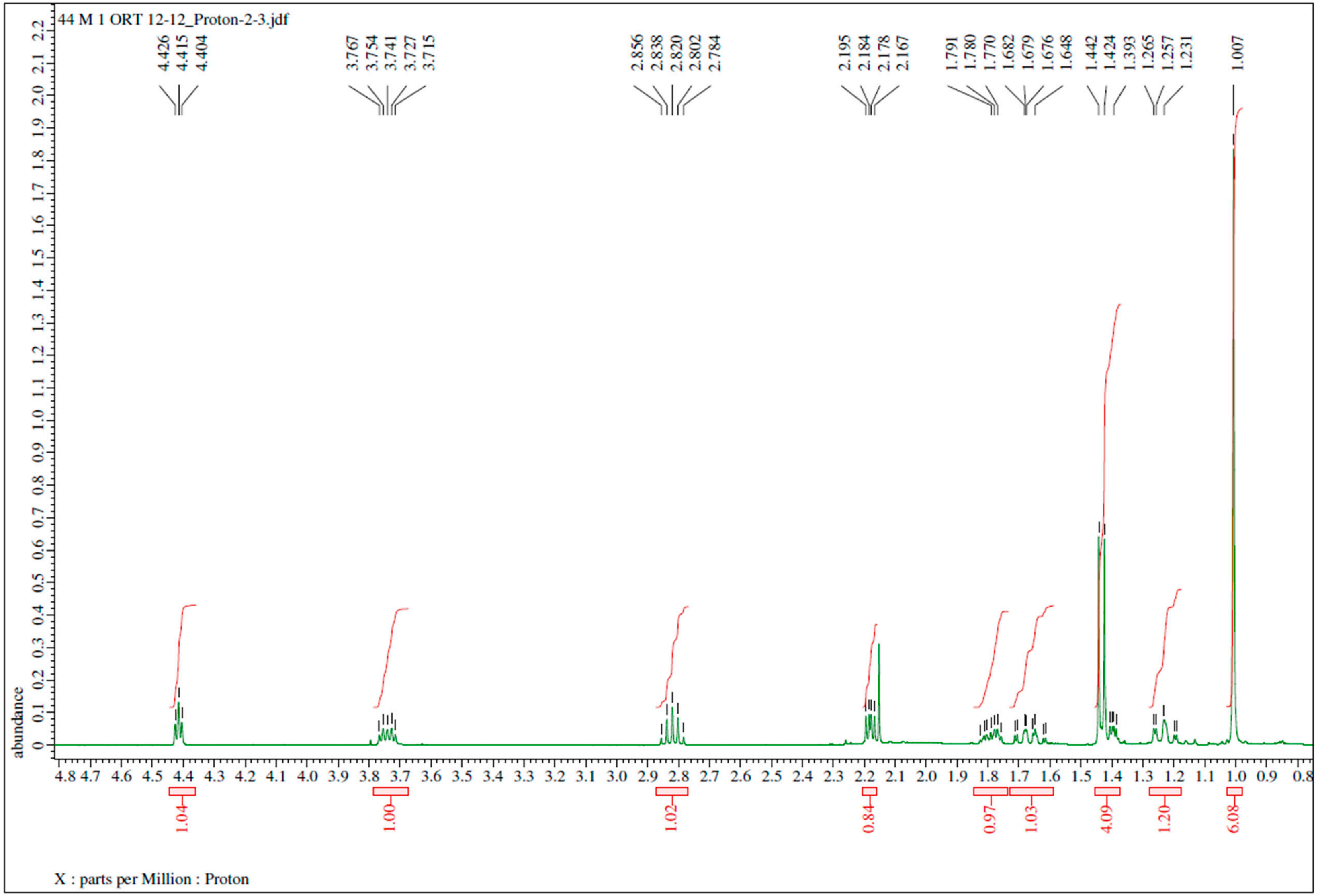

Figure S96. ^{1}H NMR (400 MHz, $CDCl_3$) spectrum of hydroxylactone **7a**

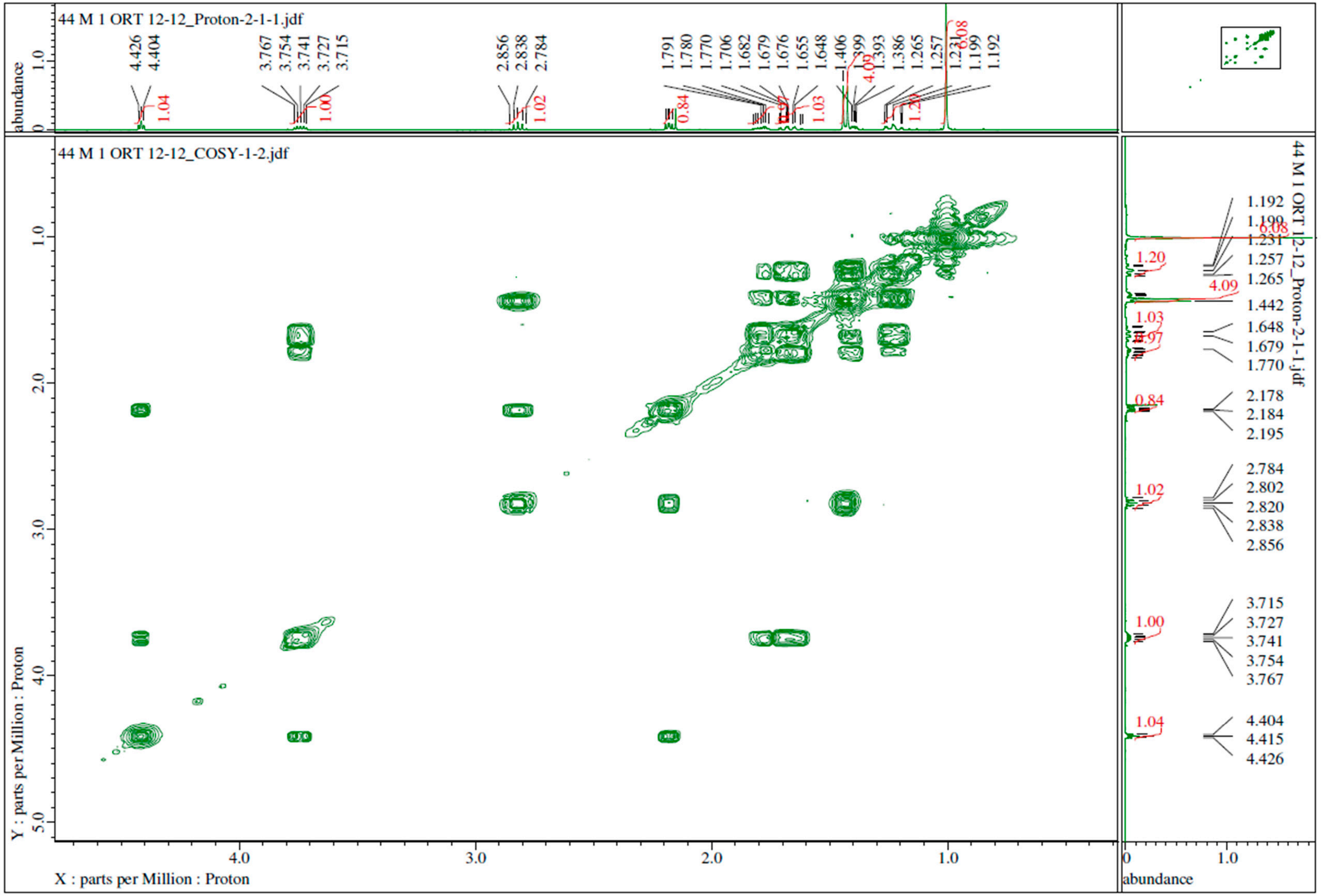

Figure S97. COSY (100 MHz, CDCl3) spectrum of hydroxylactone **7a**

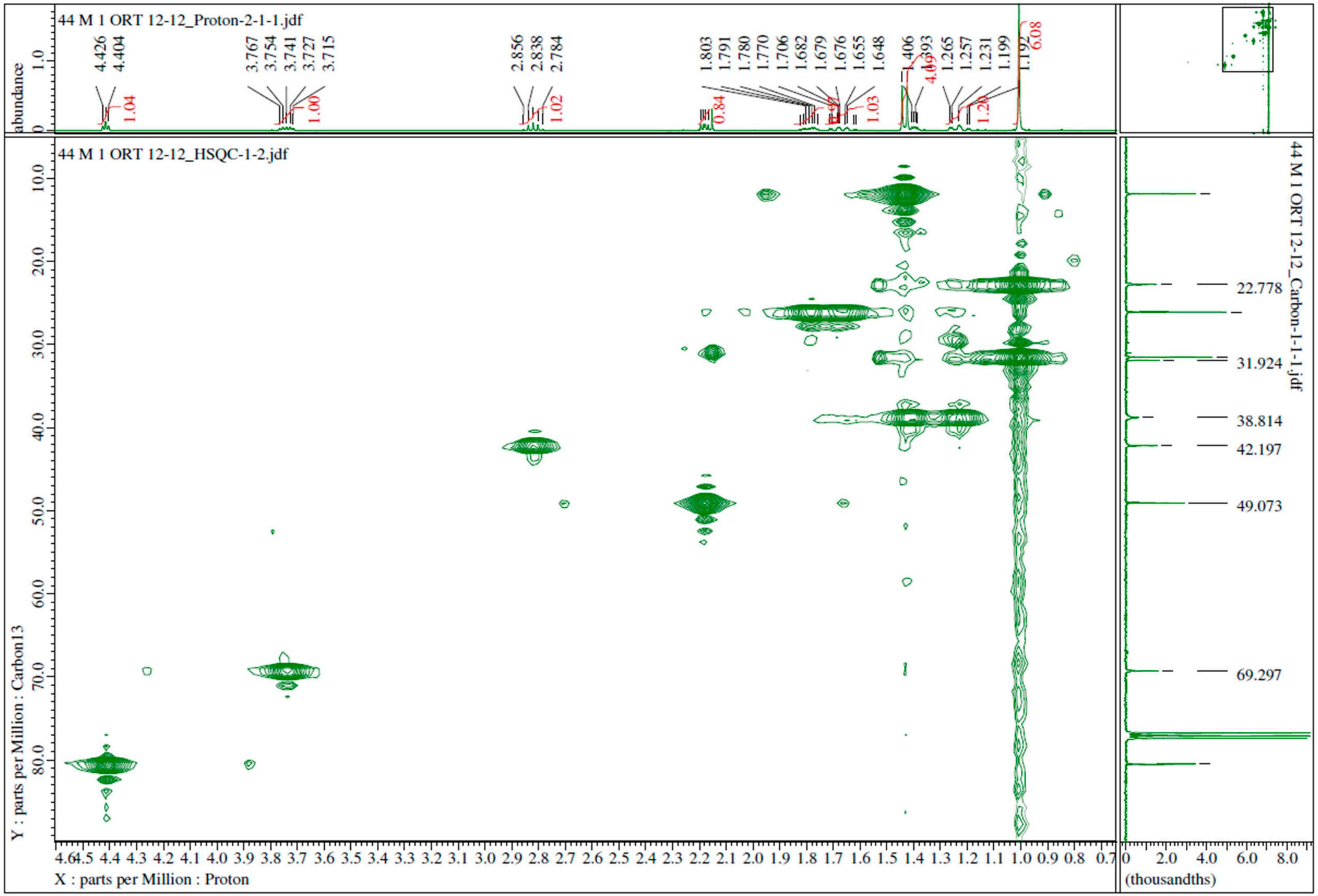

Figure S98. HMQC (100 MHz, $CDCl_3$) spectrum of hydroxylactone **7a**

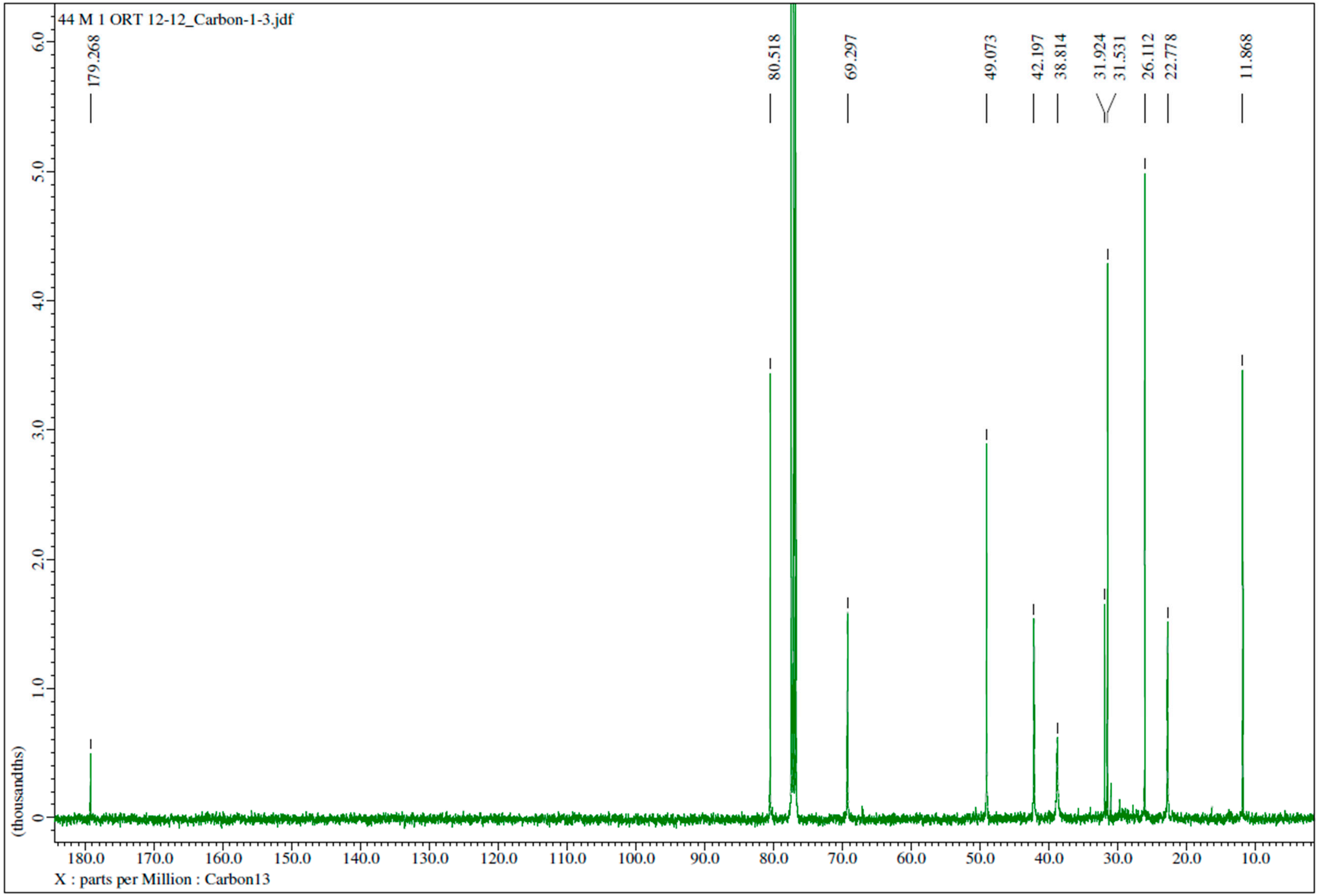

Figure S99. ^{13}C NMR (100 MHz, $CDCl_3$) spectrum of hydroxylactone **7a**

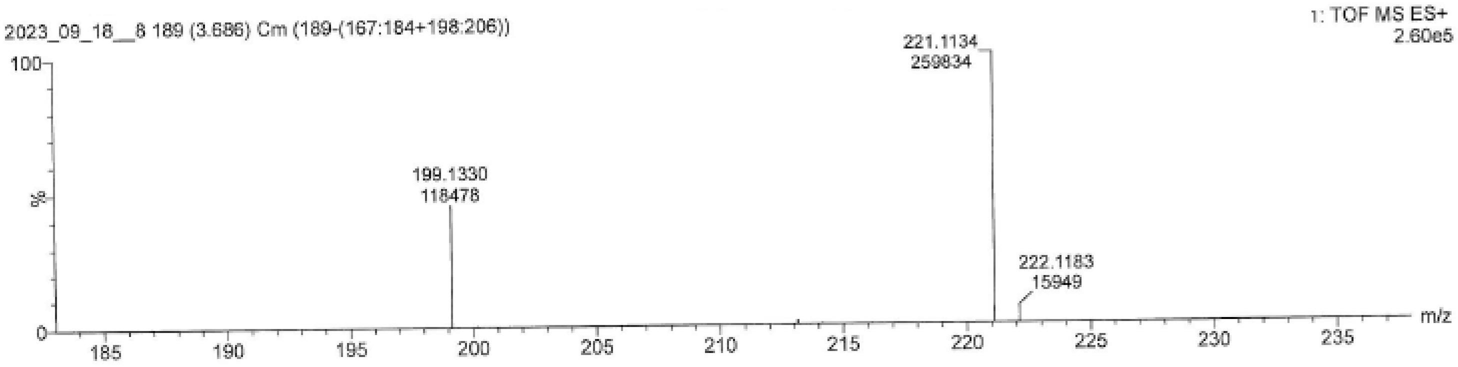

Figure S100. HRMS spectrum of hydroxylactone **7a**

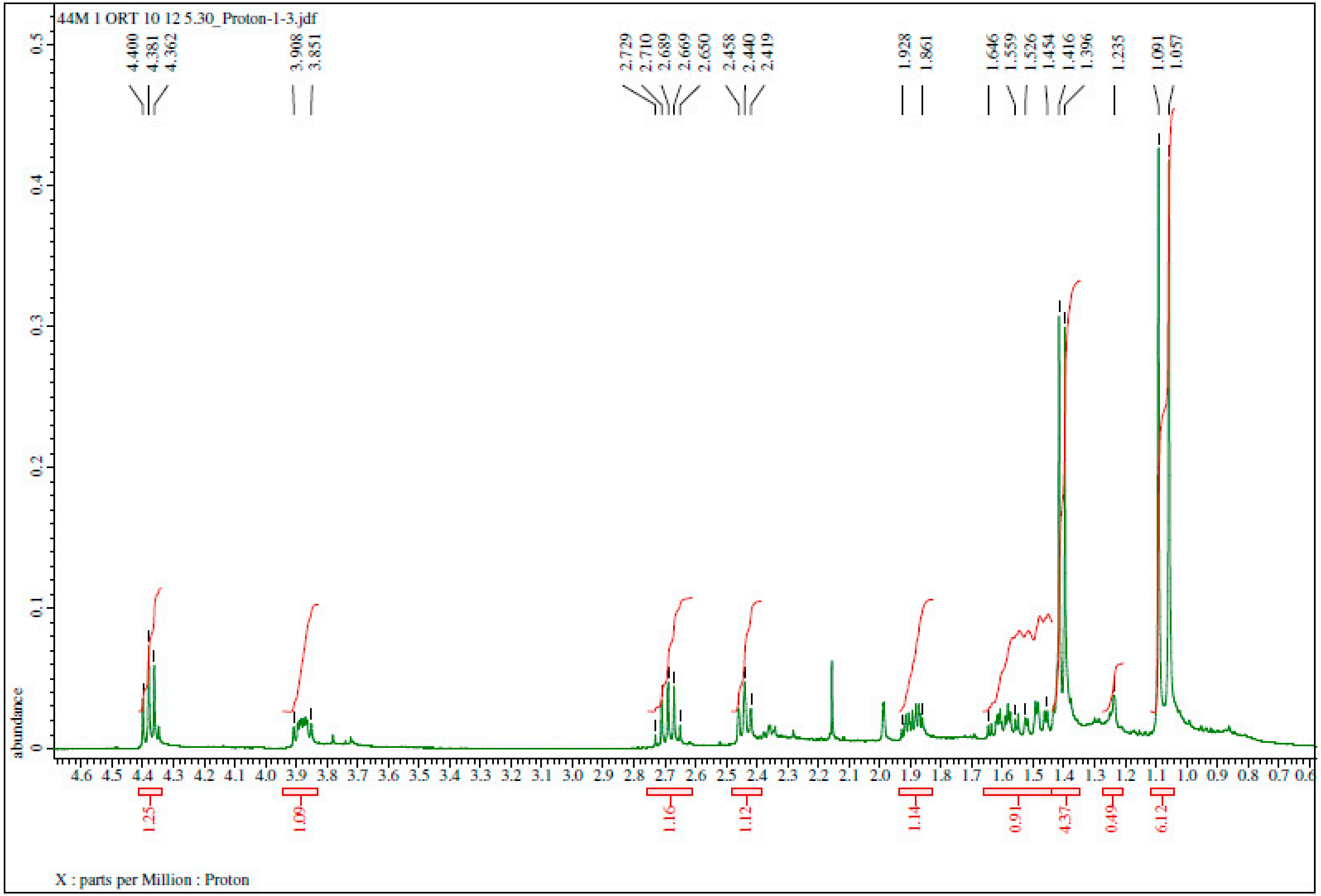

Figure S101. ^{1}H NMR (400 MHz, $CDCl_3$) spectrum of hydroxylactone **8a**

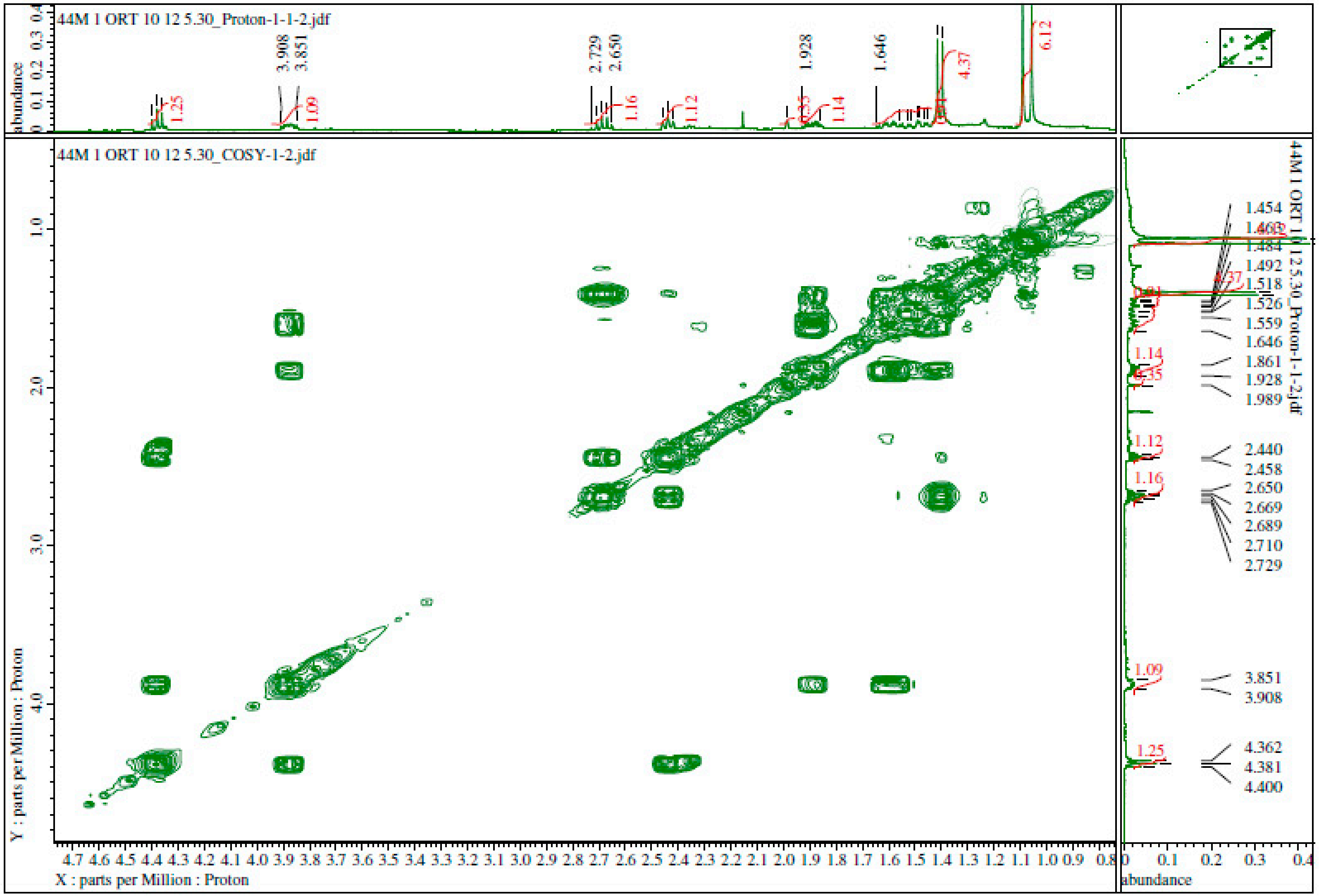

Figure S102. COSY (100 MHz, $CDCl_3$) spectrum of hydroxylactone **8a**

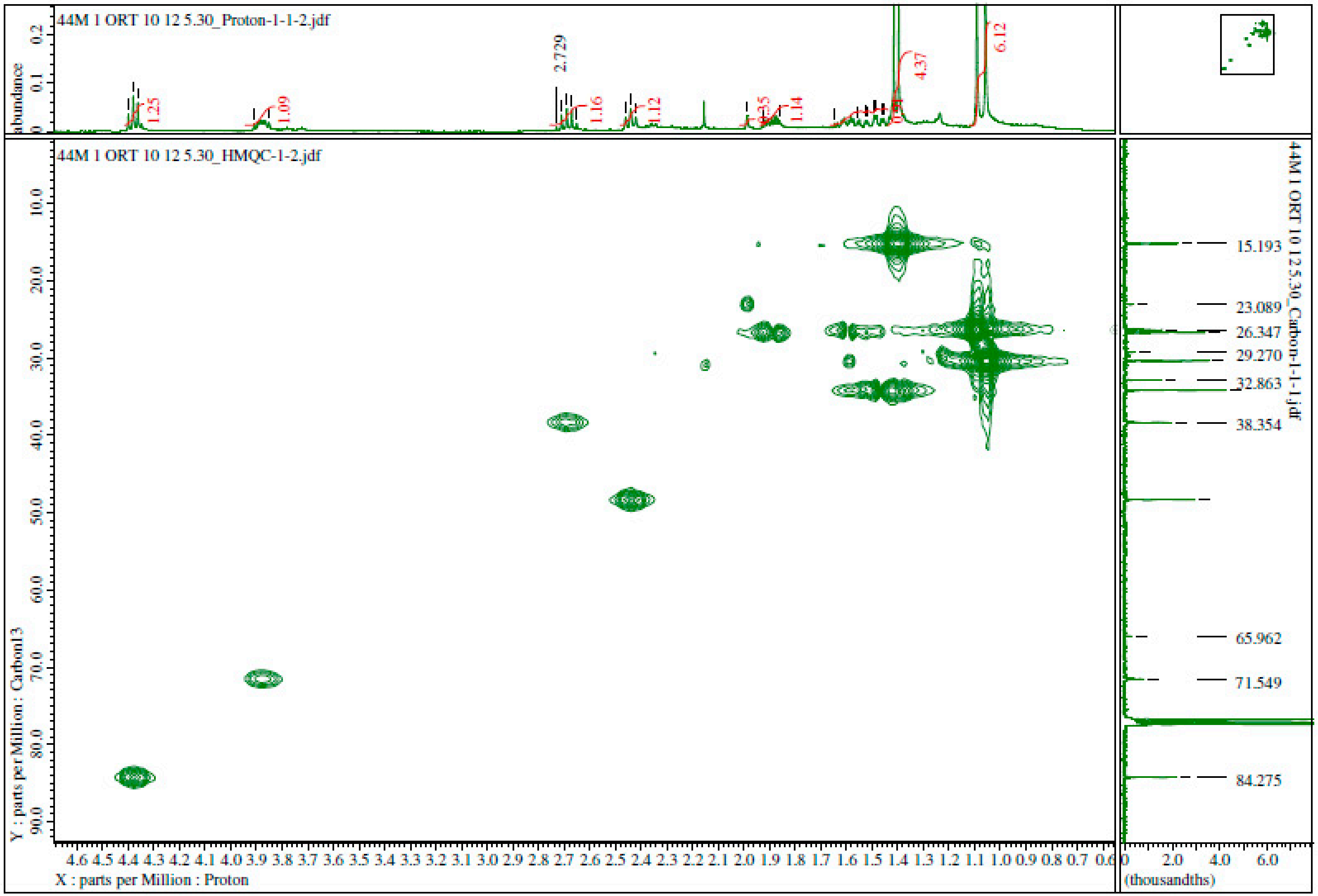

Figure S103. HMQC (100 MHz, $CDCl_3$) spectrum of hydroxylactone **8a**

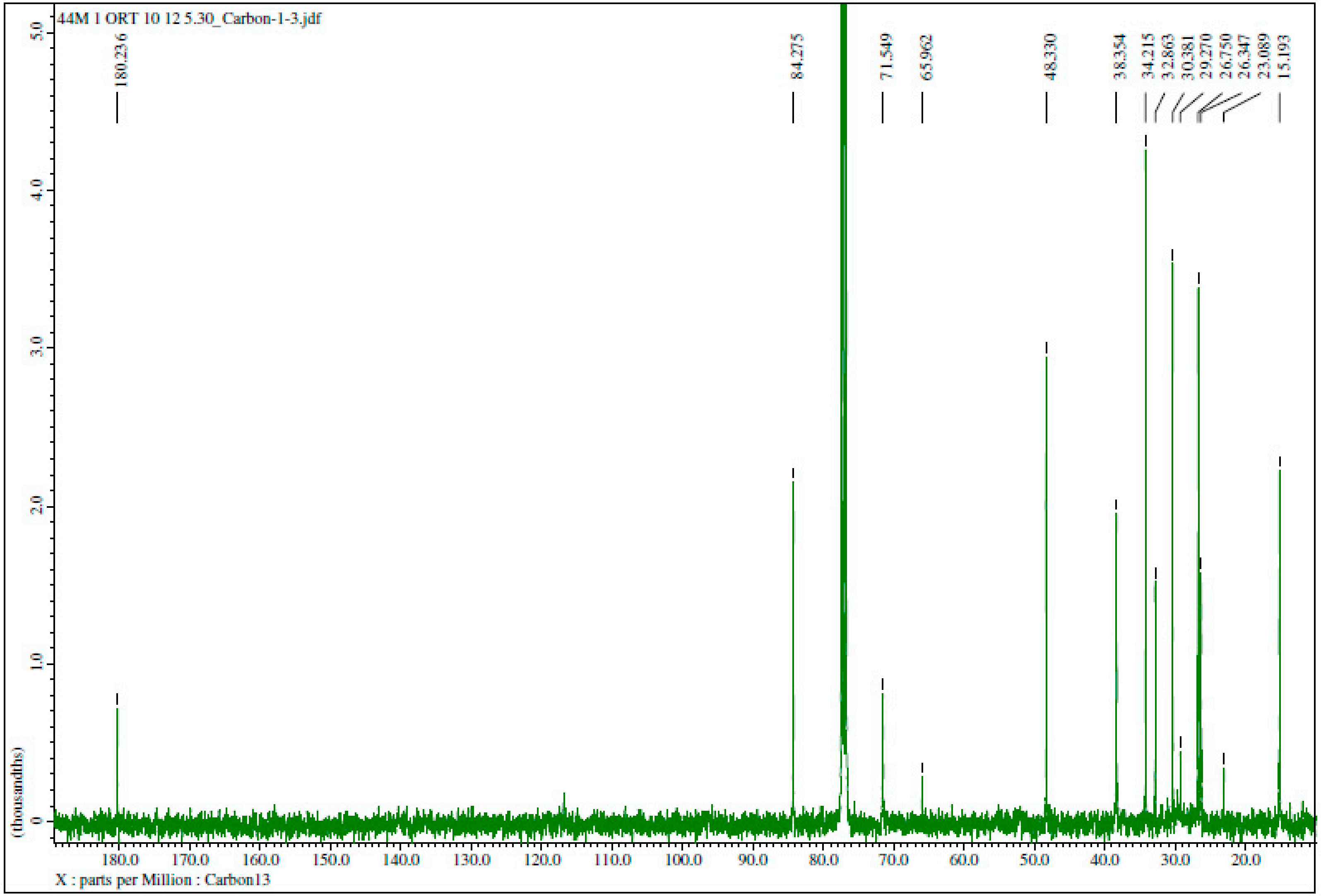

Figure S104. ^{13}C NMR (100 MHz, $CDCl_3$) spectrum of hydroxylactone **8a**

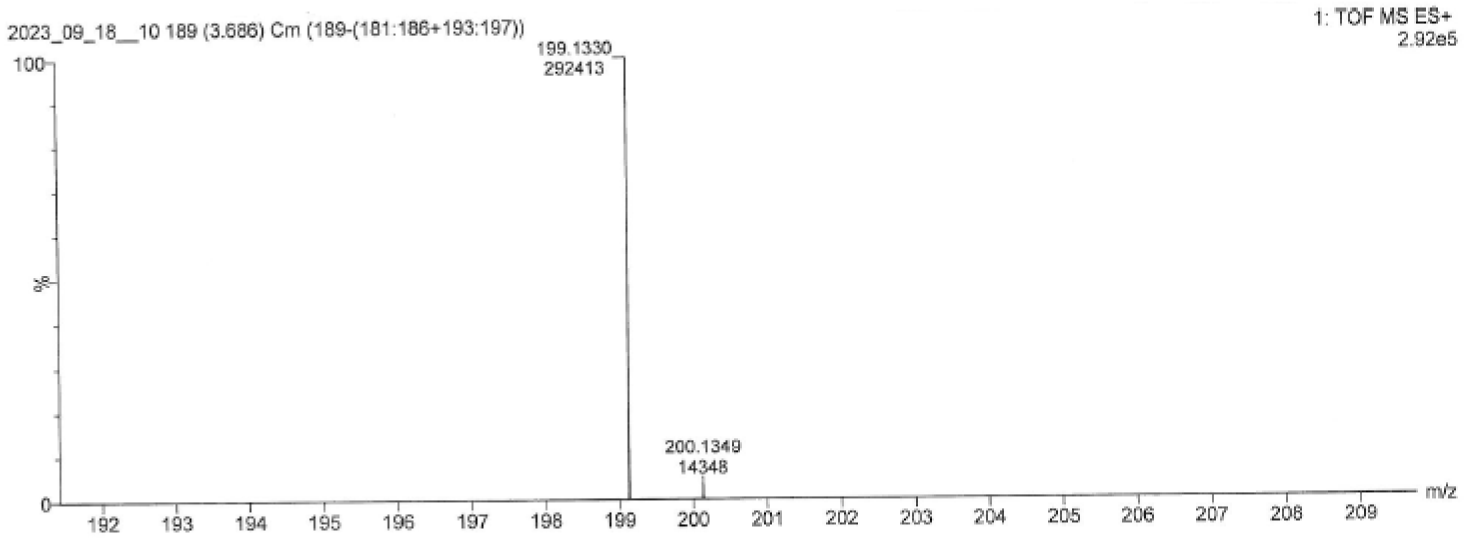

Figure S105. HRMS spectrum of bromo-hydroxylactone **8a**

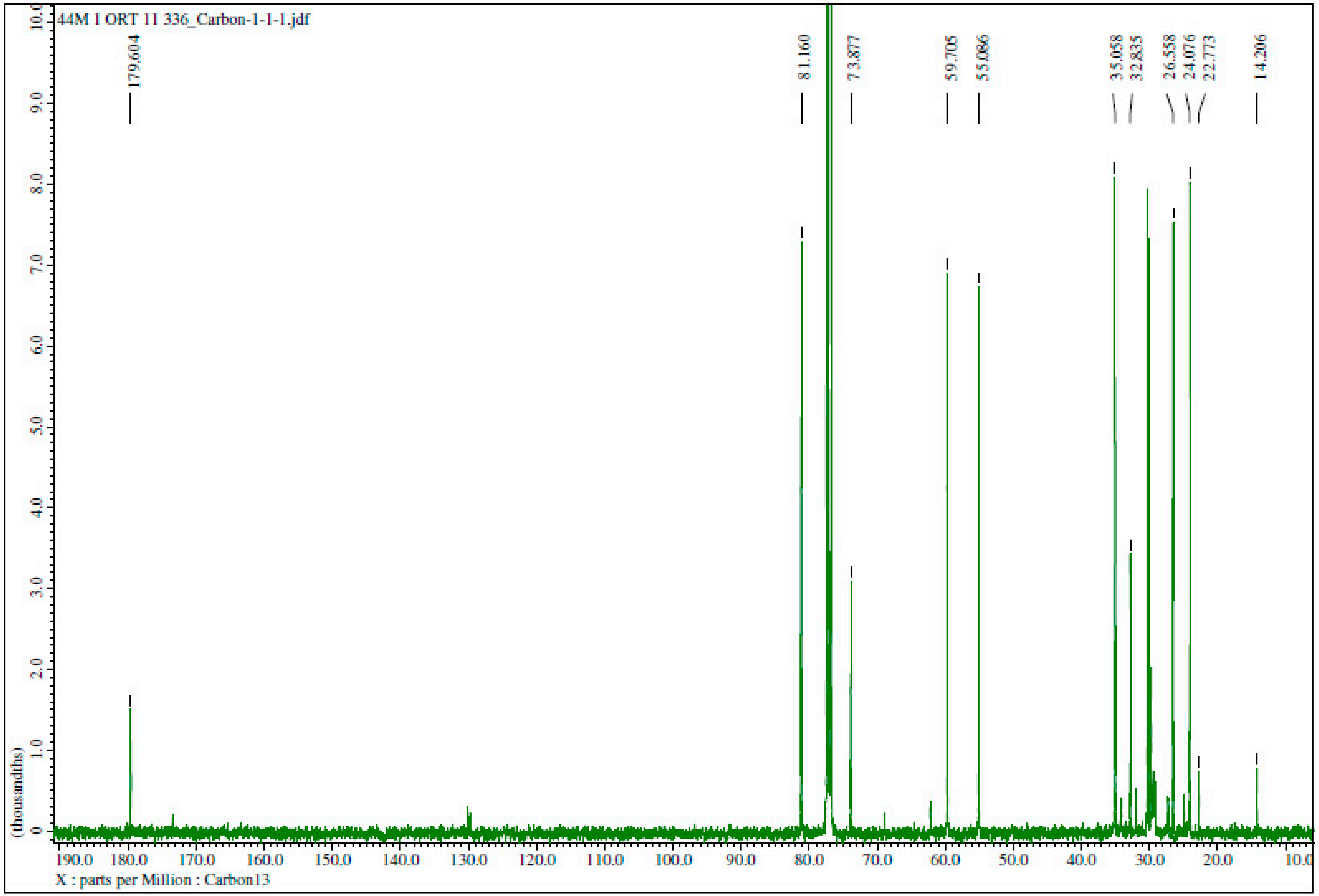

Figure S106. ^{1}H NMR (600 MHz, $CDCl_3$) spectrum of chloro-hydroxylactone **9a**

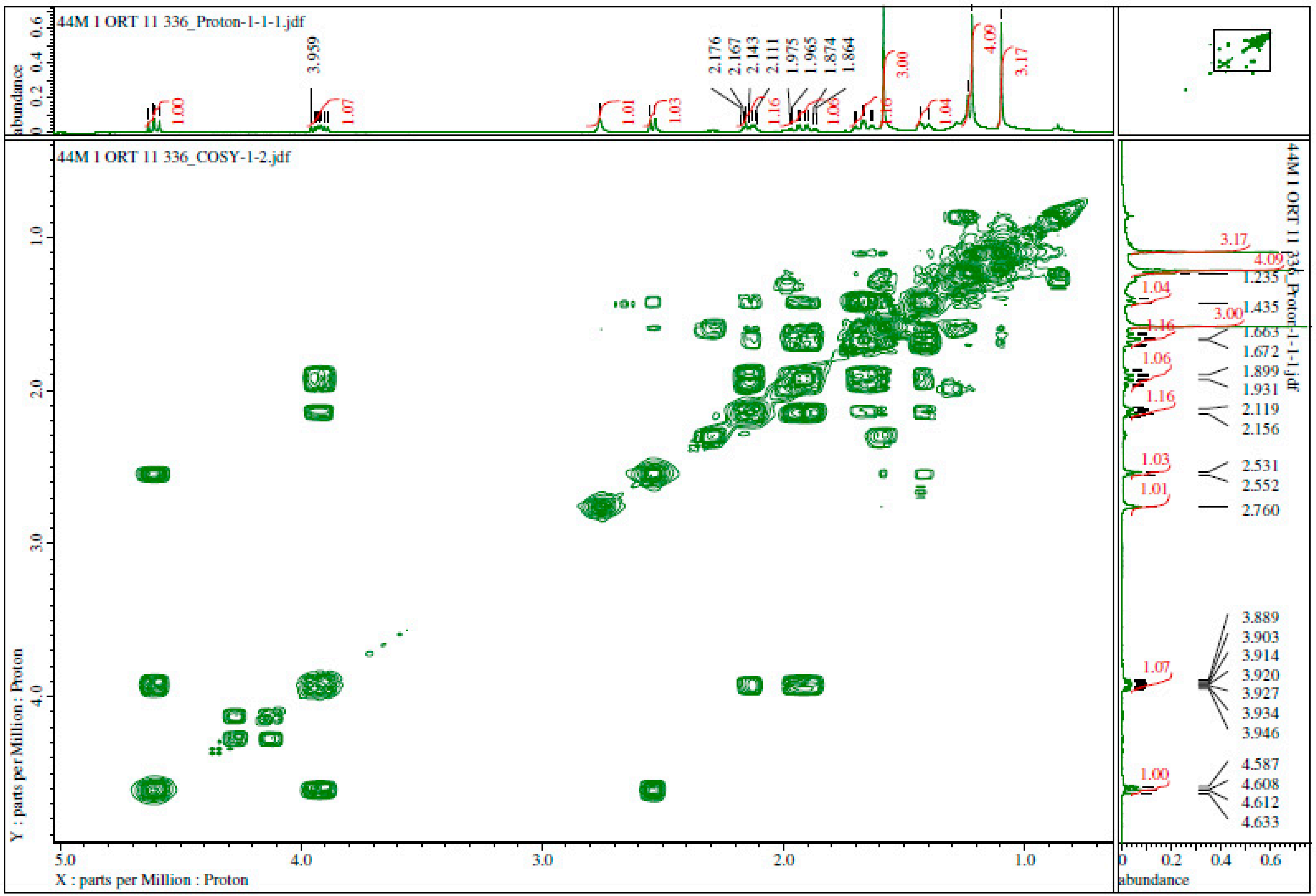

Figure S107. COSY (151 MHz, $CDCl_3$) spectrum of chloro-hydroxylactone **9a**

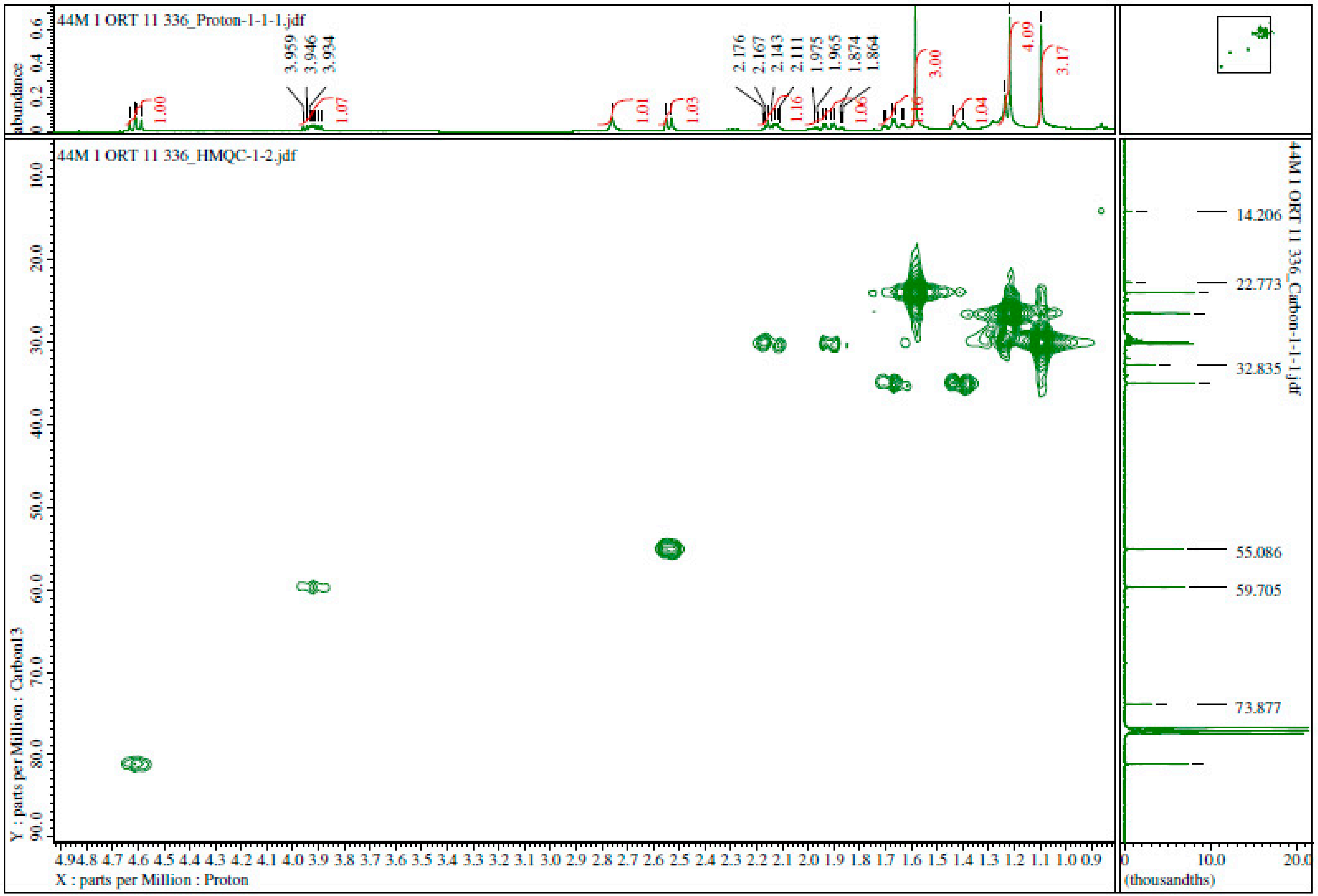

Figure S108. HMQC (151 MHz, $CDCl_3$) spectrum of chloro-hydroxylactone **9a**

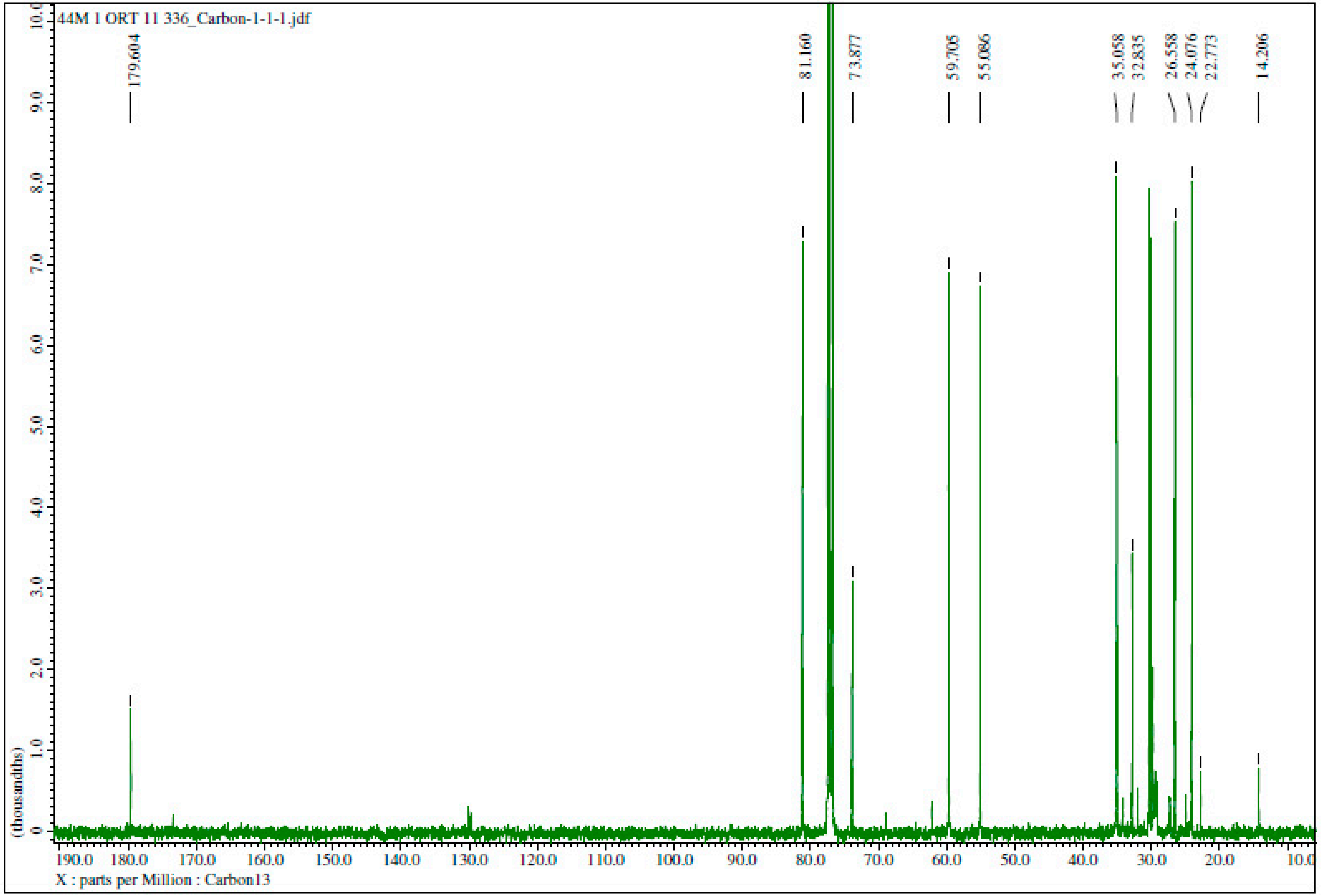

Figure S109. ^{13}C NMR (151 MHz, $CDCl_3$) spectrum of chloro-hydroxylactone **9a**

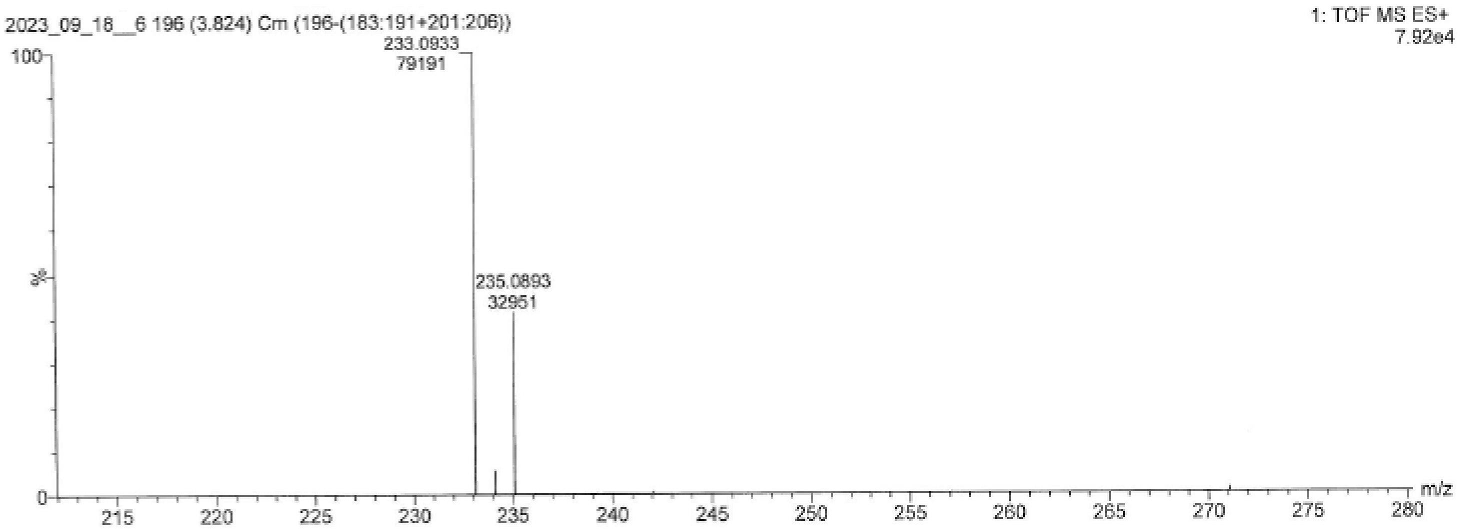

Figure S110. HRMS spectrum of chloro-hydroxylactone **9a**

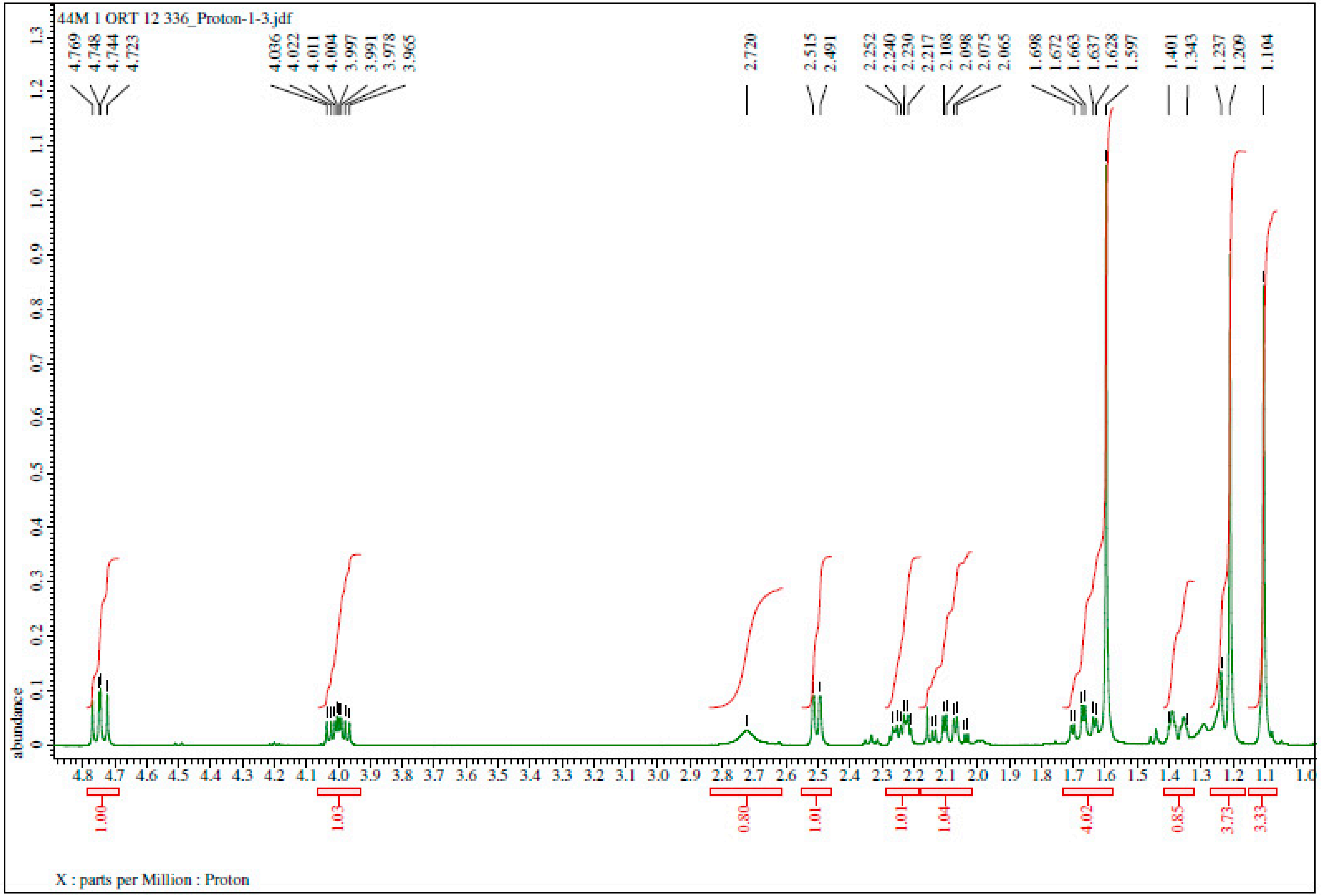

Figure S111. ^{1}H NMR (400 MHz, $CDCl_3$) spectrum of bromo-hydroxylactone **10a**

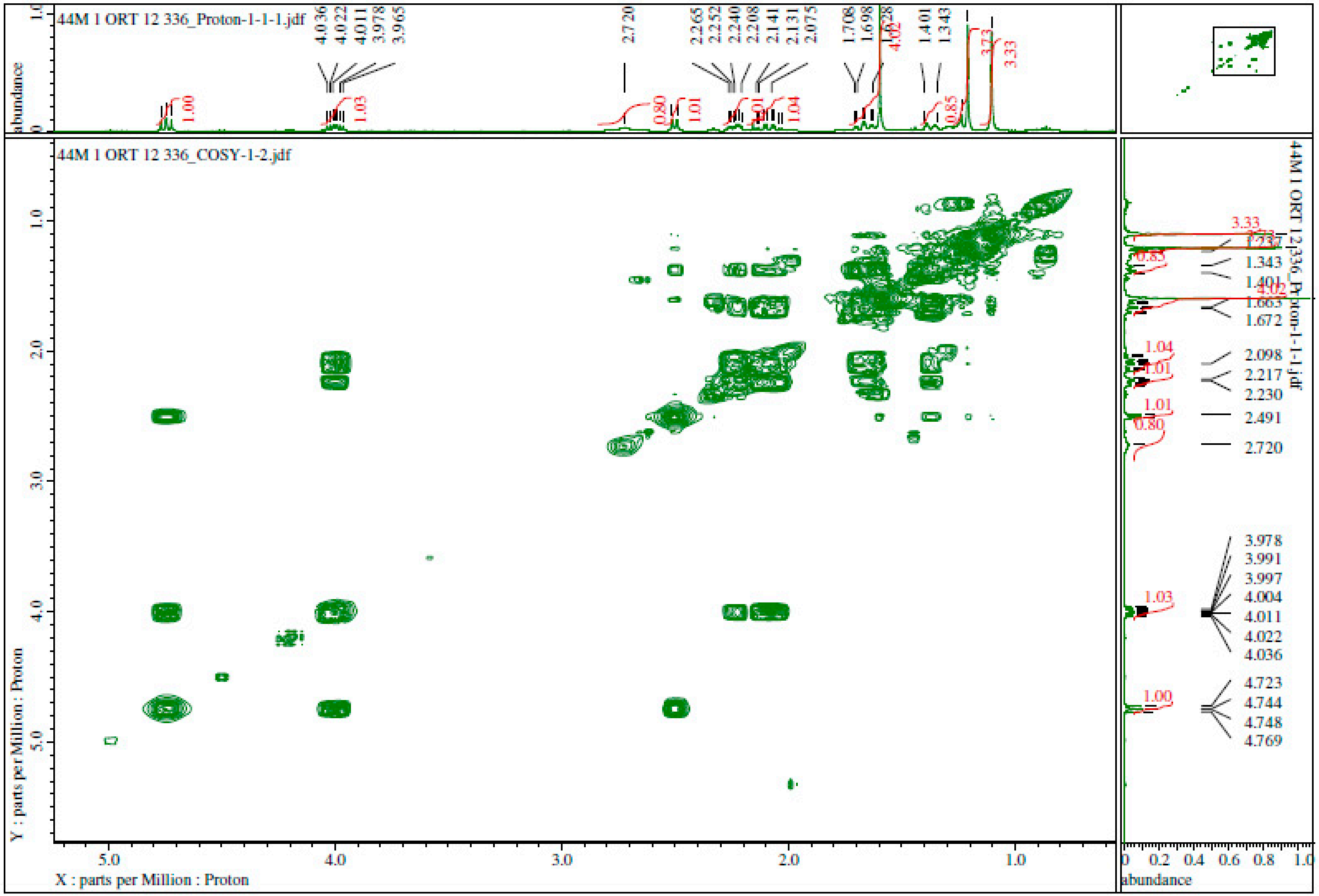

Figure S112. COSY (100 MHz, $CDCl_3$) spectrum of bromo-hydroxylactone **10a**

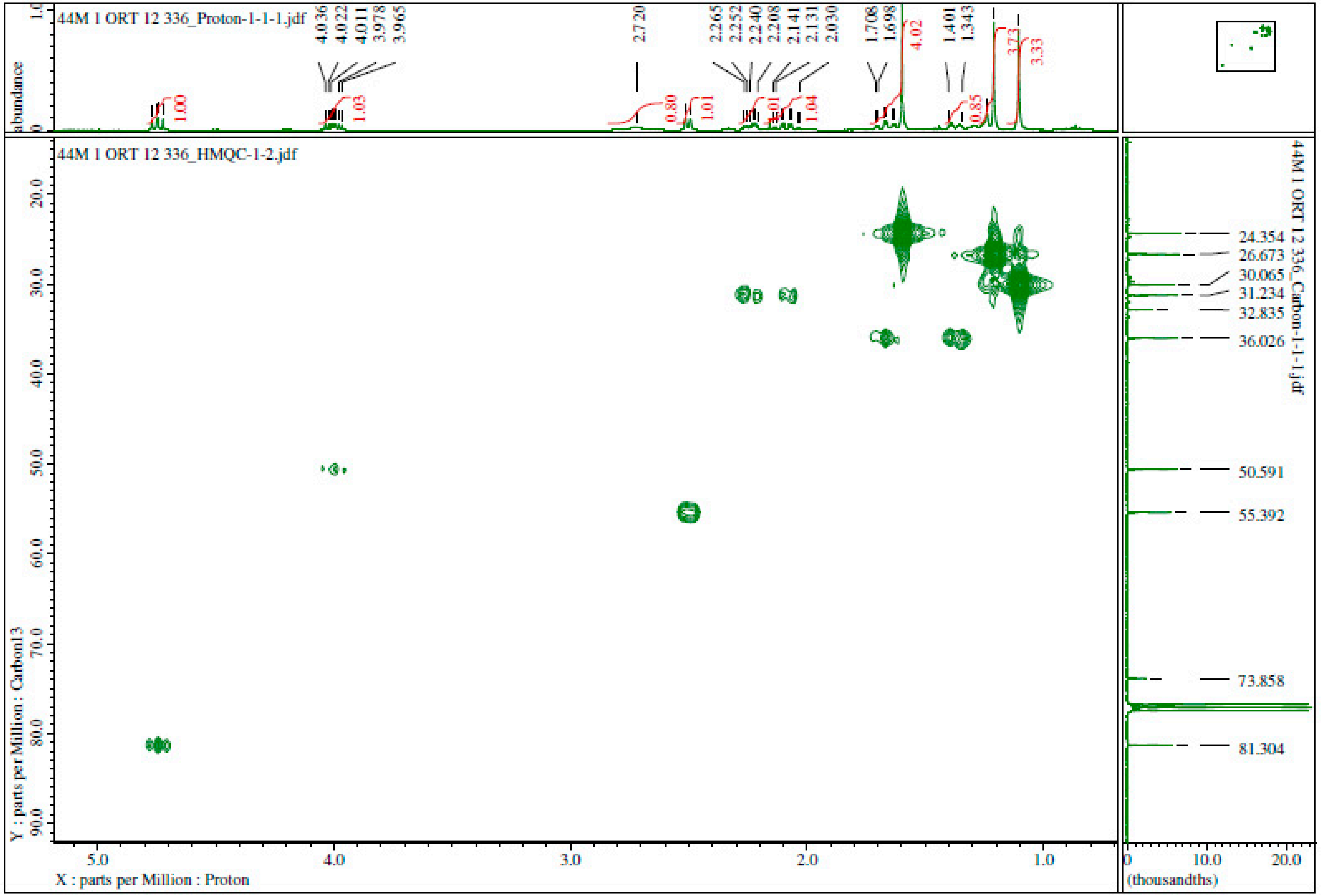

Figure S113. HMQC (100 MHz, $CDCl_3$) spectrum of bromo-hydroxylactone **10a**

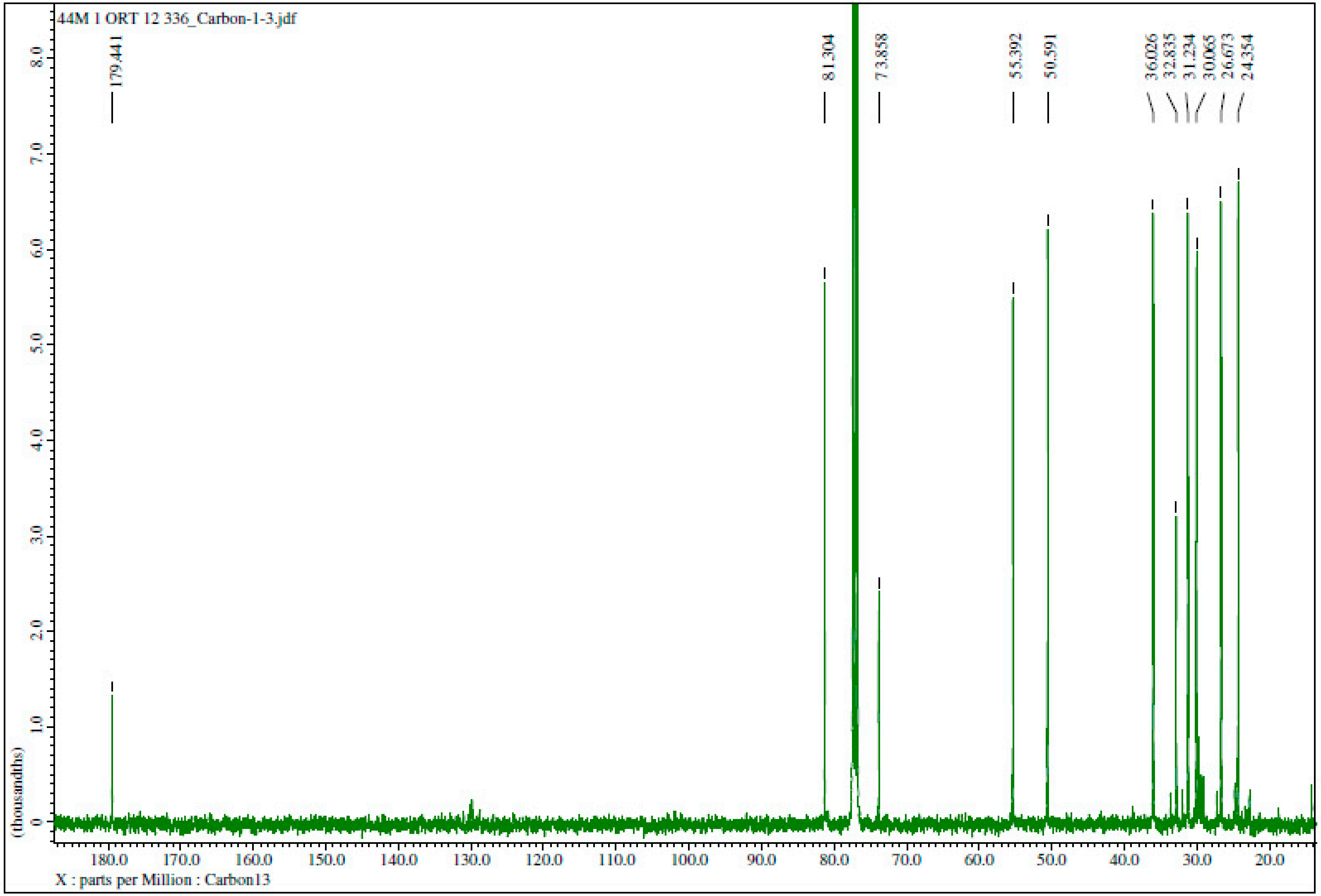

Figure S114. ^{13}C NMR (100 MHz, $CDCl_3$) spectrum of bromo-hydroxylactone **10a**

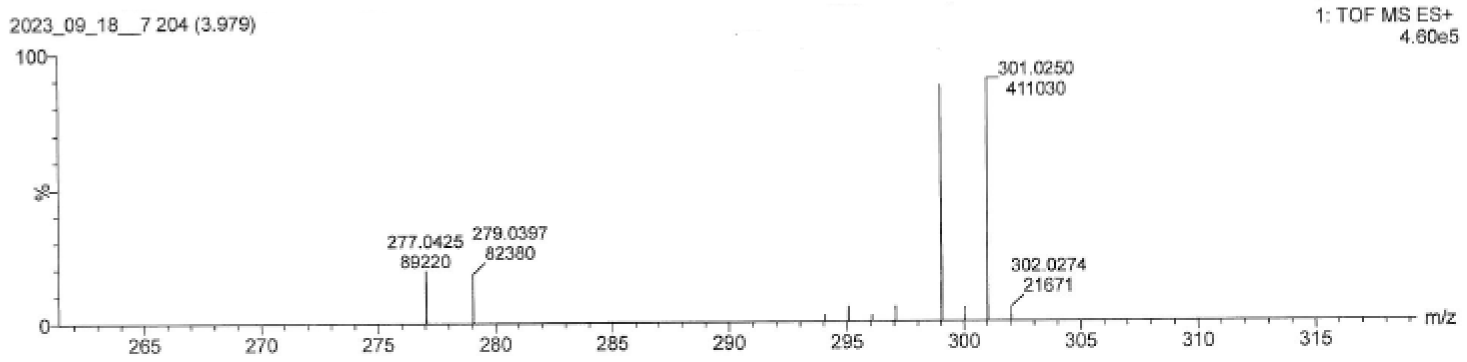

Figure S115. HRMS spectrum of bromo -hydroxylactone **10a**

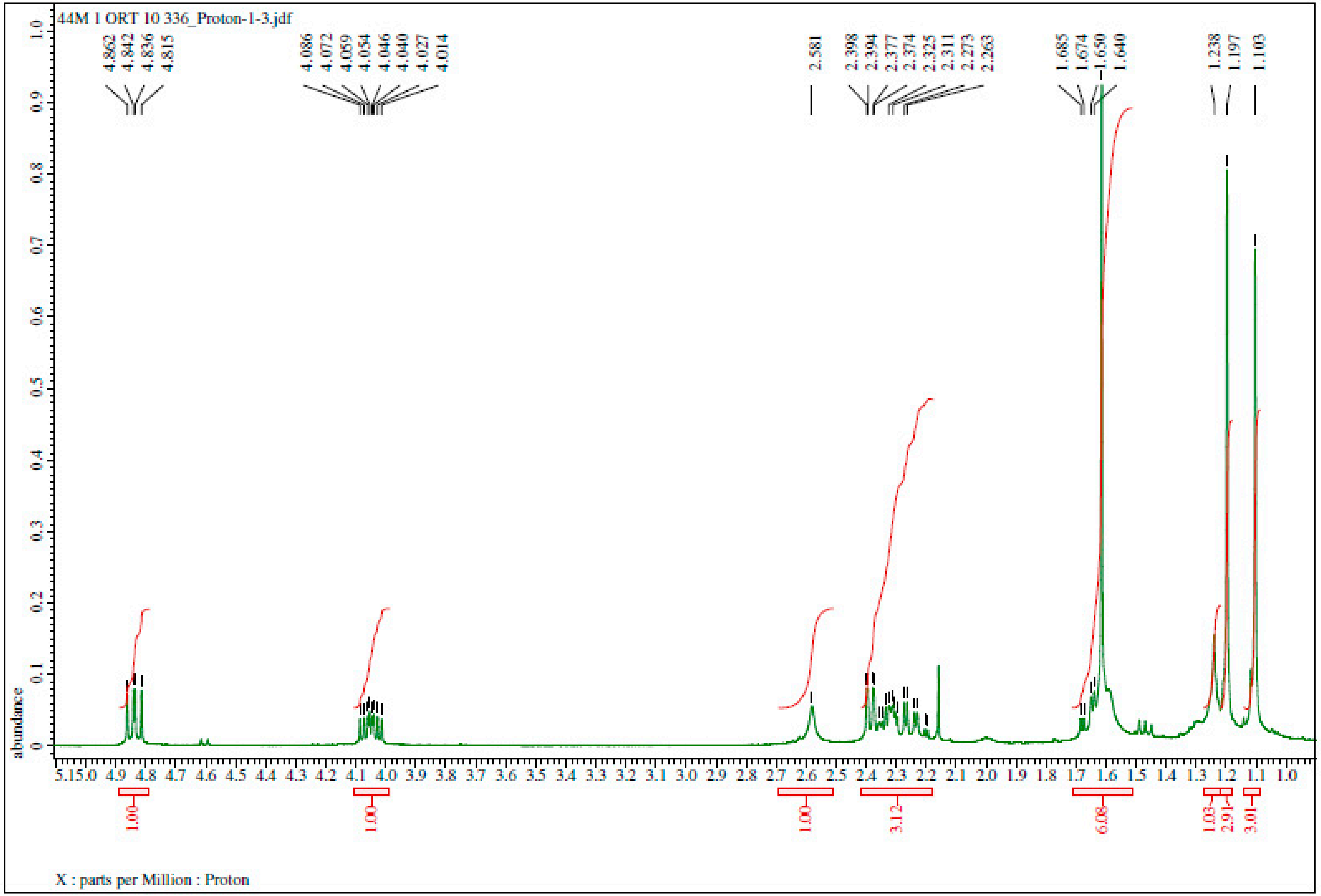

Figure S116. ^{1}H NMR (400 MHz, $CDCl_3$) spectrum of iodo-hydroxylactone **11a**

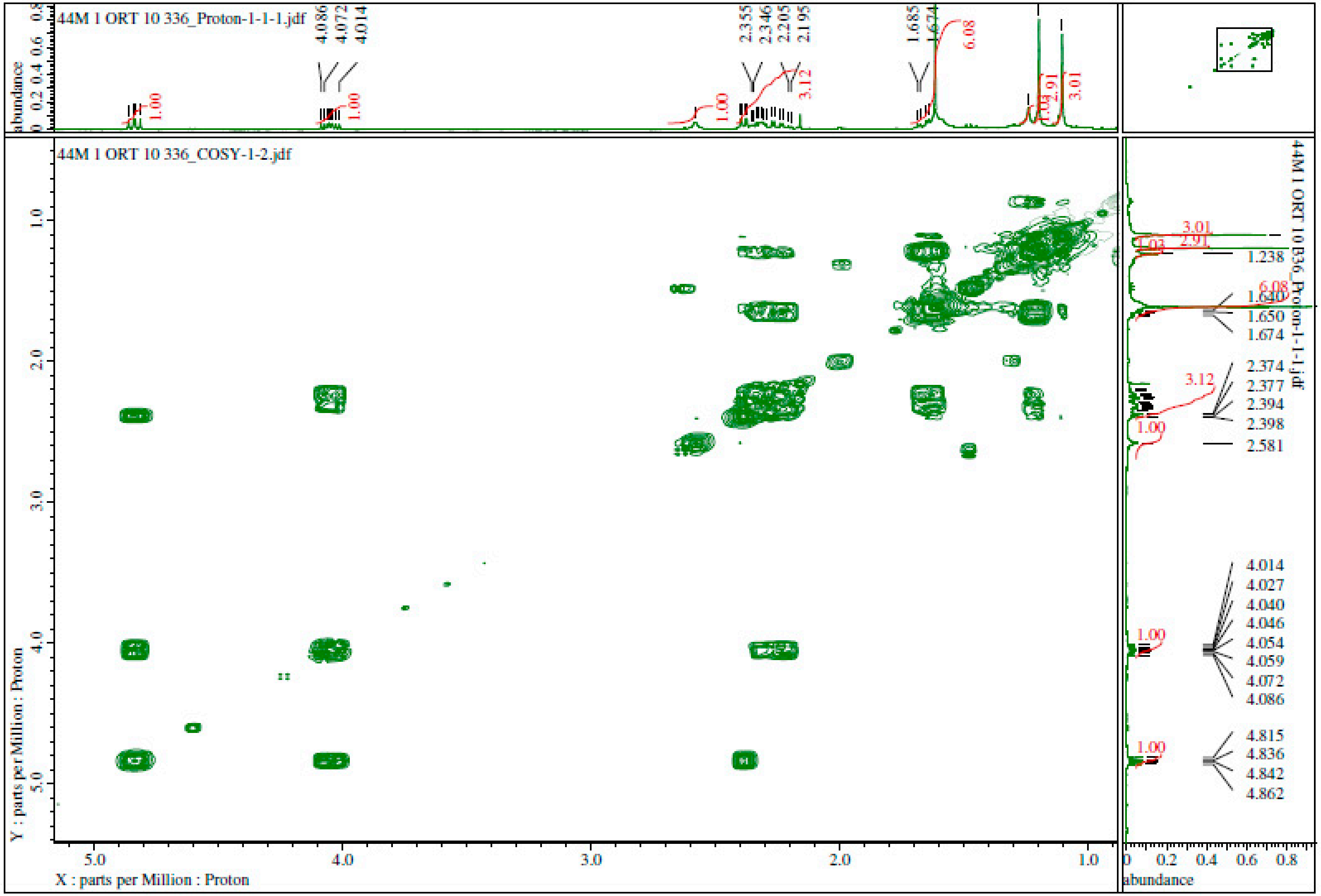

Figure S117. COSY (100 MHz, $CDCl_3$) spectrum of iodo-hydroxylactone **11a**

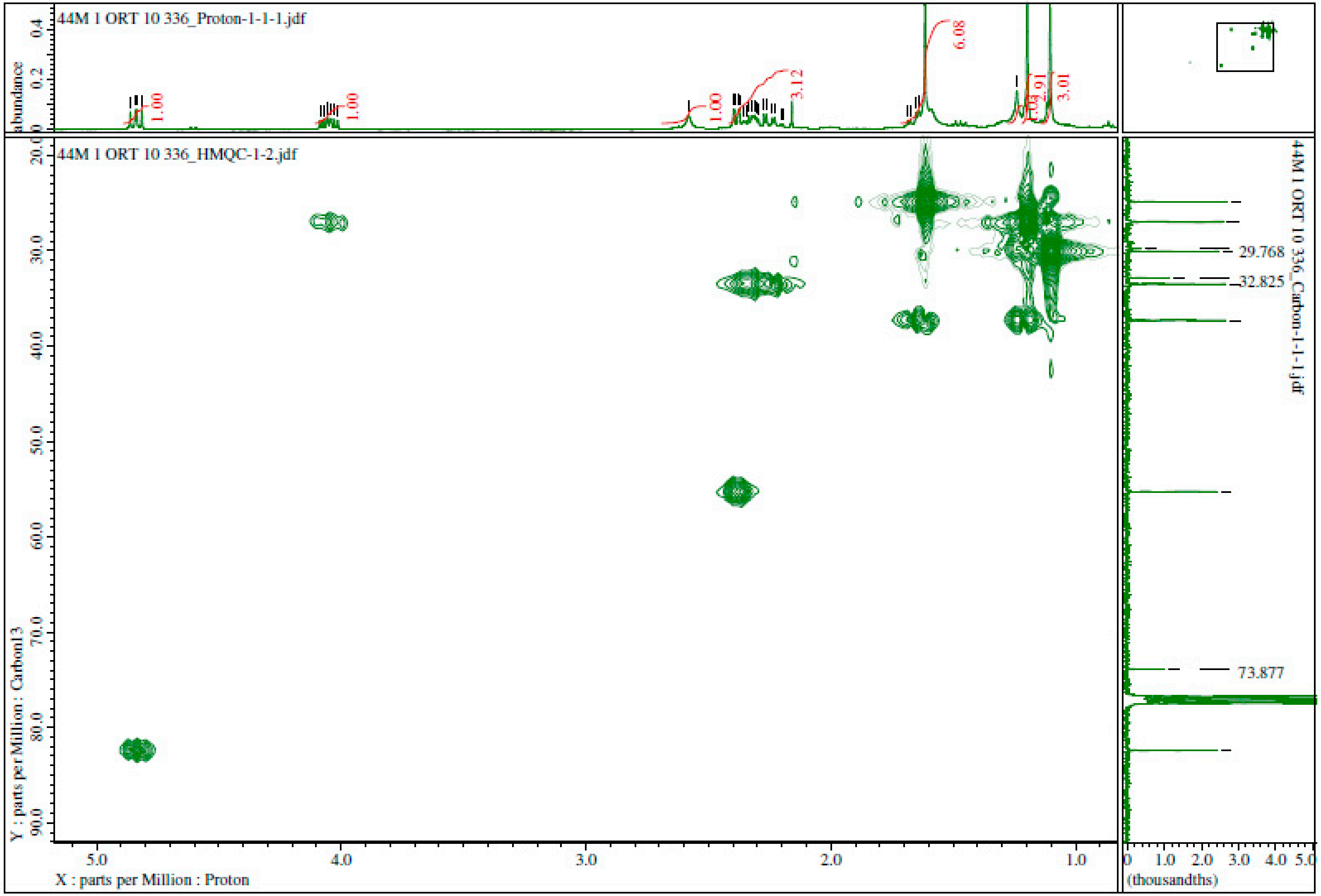

Figure S118. HMQC (100 MHz, $CDCl_3$) spectrum of iodo-hydroxylactone **11a**

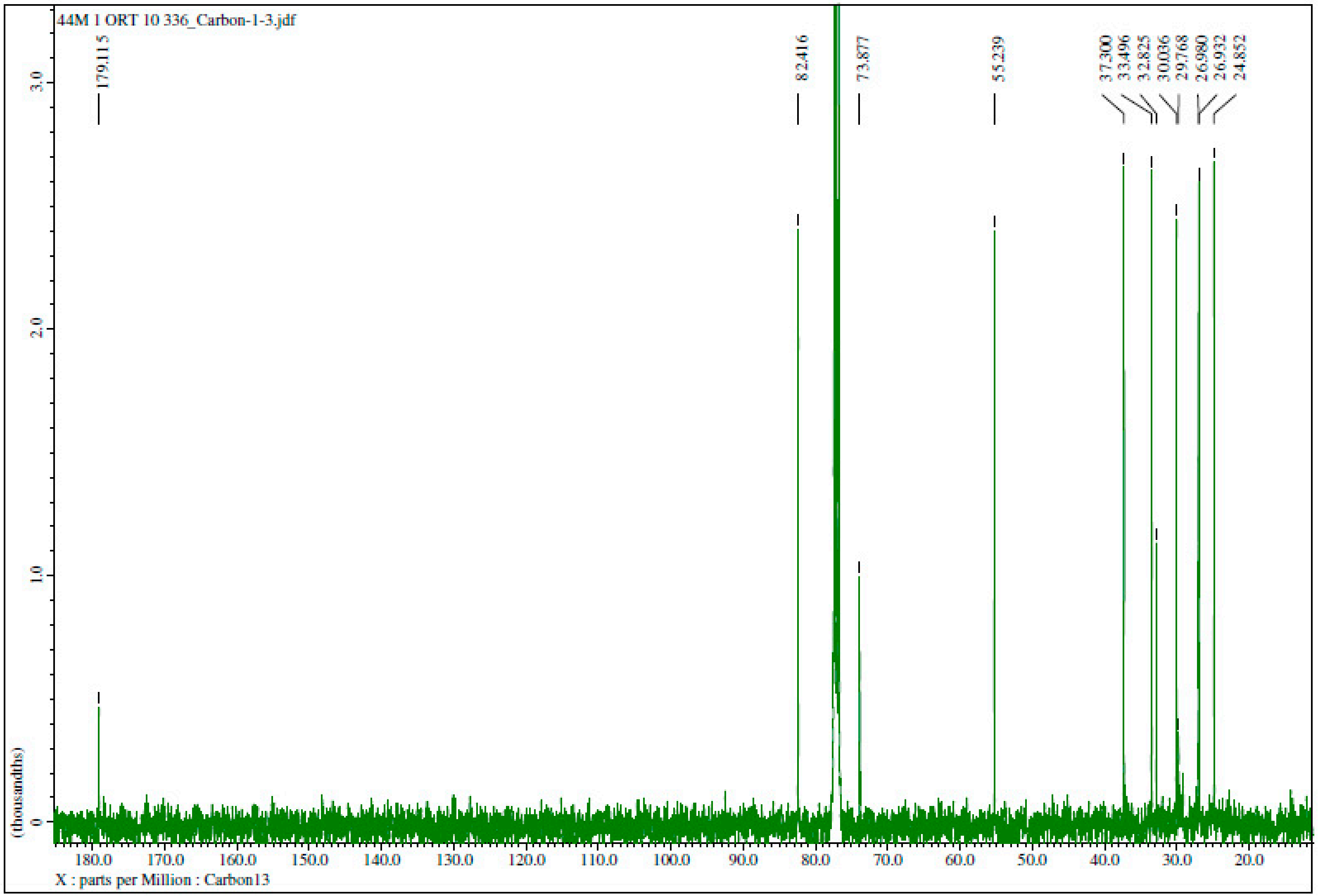

Figure S119. ^{13}C NMR (100 MHz, $CDCl_3$) spectrum of iodo-hydroxylactone **11a**

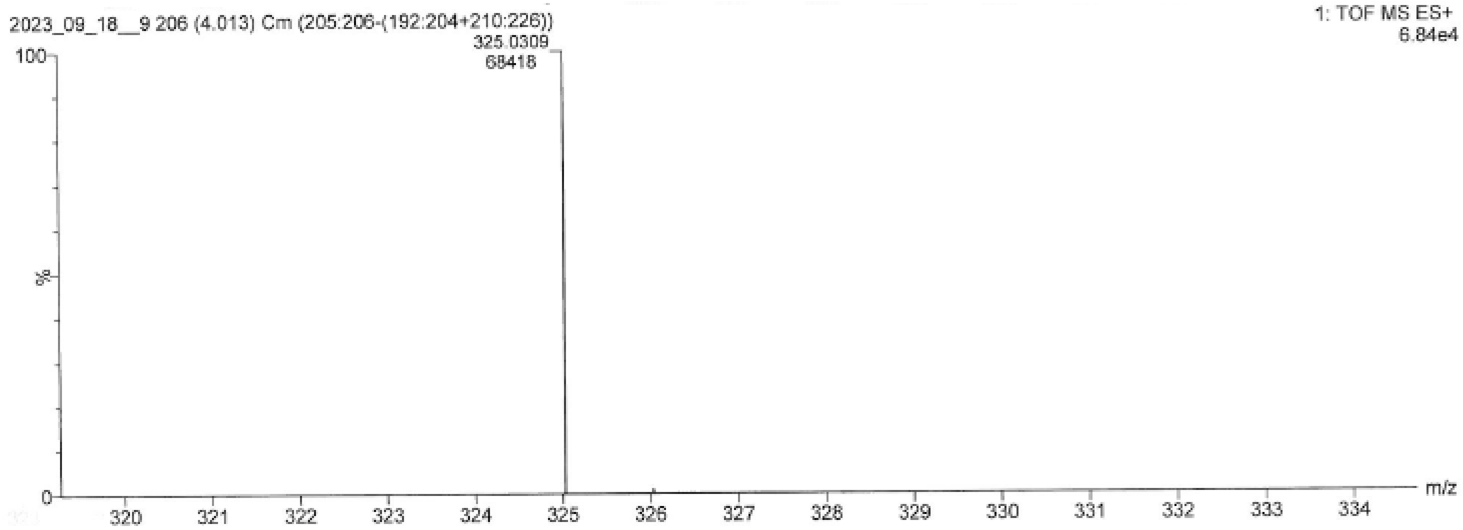

Figure S120. HRMS spectrum of iodo-hydroxylactone **11a**

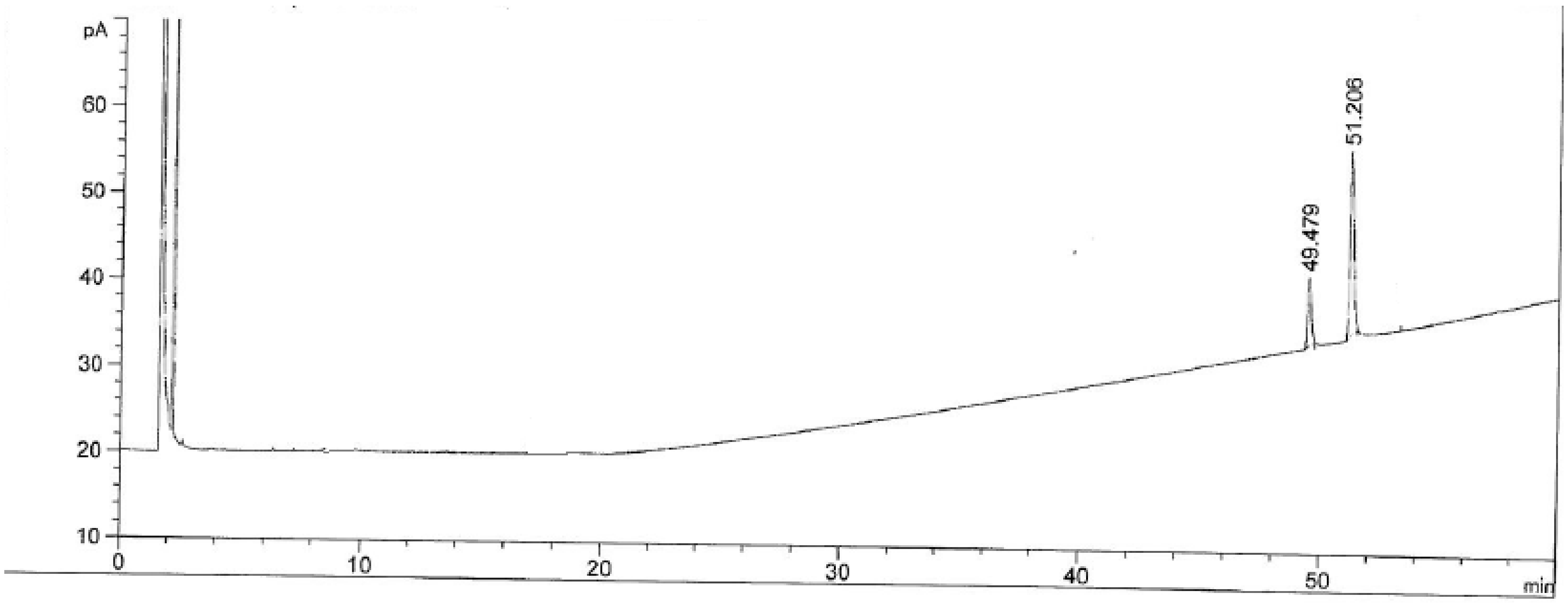

Peak #	RetTime [min]	Type	Width [min]	Area [pA*s]	Height [pA]	Area %
1	49.479	BB	0.1280	78.87131	7.86426	25.54474
2	51.206	BB	0.1290	229.88618	21.44988	74.45526

Figure S121. Chiral chromatogram of hydroxylactone **7a** obtained from bromolactone **5a**

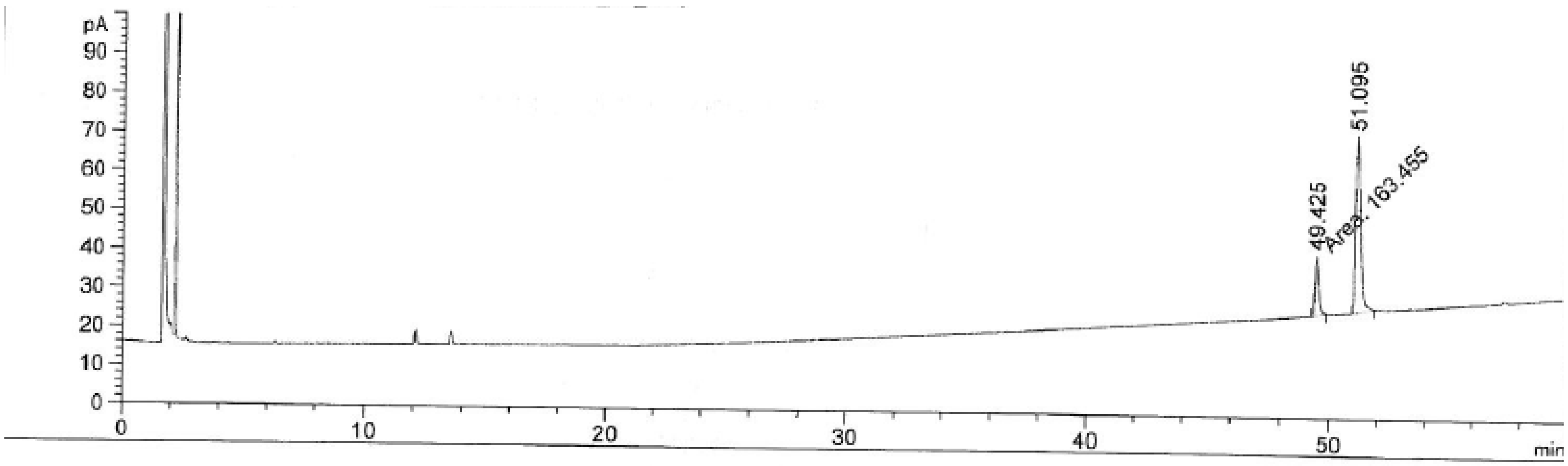

Peak #	RetTime [min]	Type	Width [min]	Area [pA*s]	Height [pA]	Area %
1	49.425	MM	0.1787	163.45482	15.24607	21.84541
2	51.095	BB	0.1592	584.77936	45.13311	78.15459

Figure S122. Chiral chromatogram of hydroxylactone **7a** obtained from iodolactone **6a**

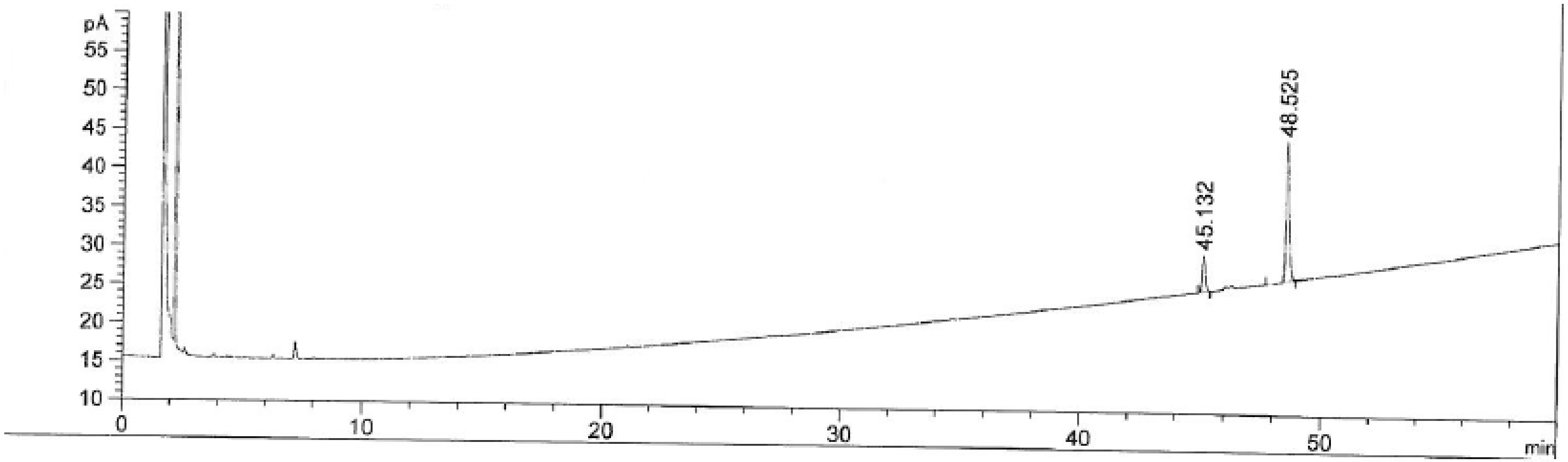

Peak #	RetTime [min]	Type	Width [min]	Area [pA*s]	Height [pA]	Area %
1	45.132	BB	0.1230	42.70560	4.65707	19.79180
2	48.525	BB	0.1229	173.06859	17.87771	80.20820

Figure S123. Chiral chromatogram of hydroxylactone **8a** obtained from iodolactone **6a**

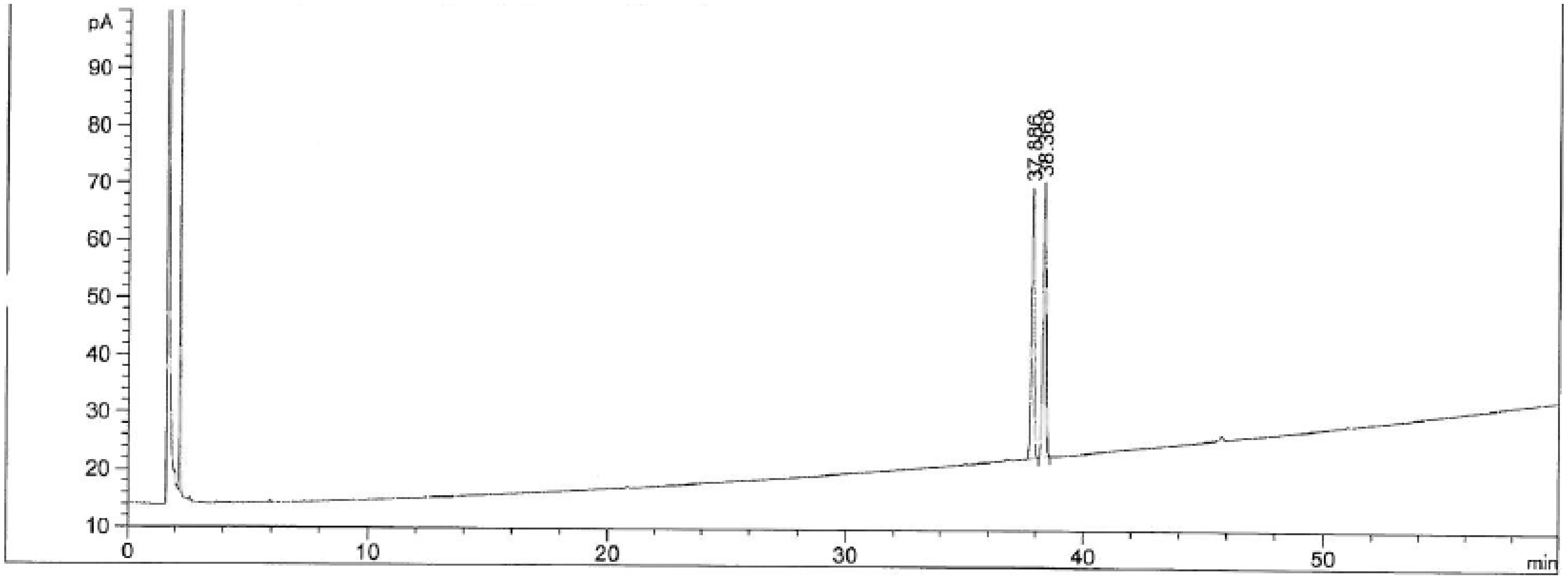

Peak #	RetTime [min]	Type	Width [min]	Area [pA*s]	Height [pA]	Area %
1	37.886	BB	0.1160	421.80237	47.29977	50.79140
2	38.368	BB	0.1166	408.65781	47.87785	49.20860

Figure S124. Chiral chromatogram of chloro-hydroxylactone **9a**

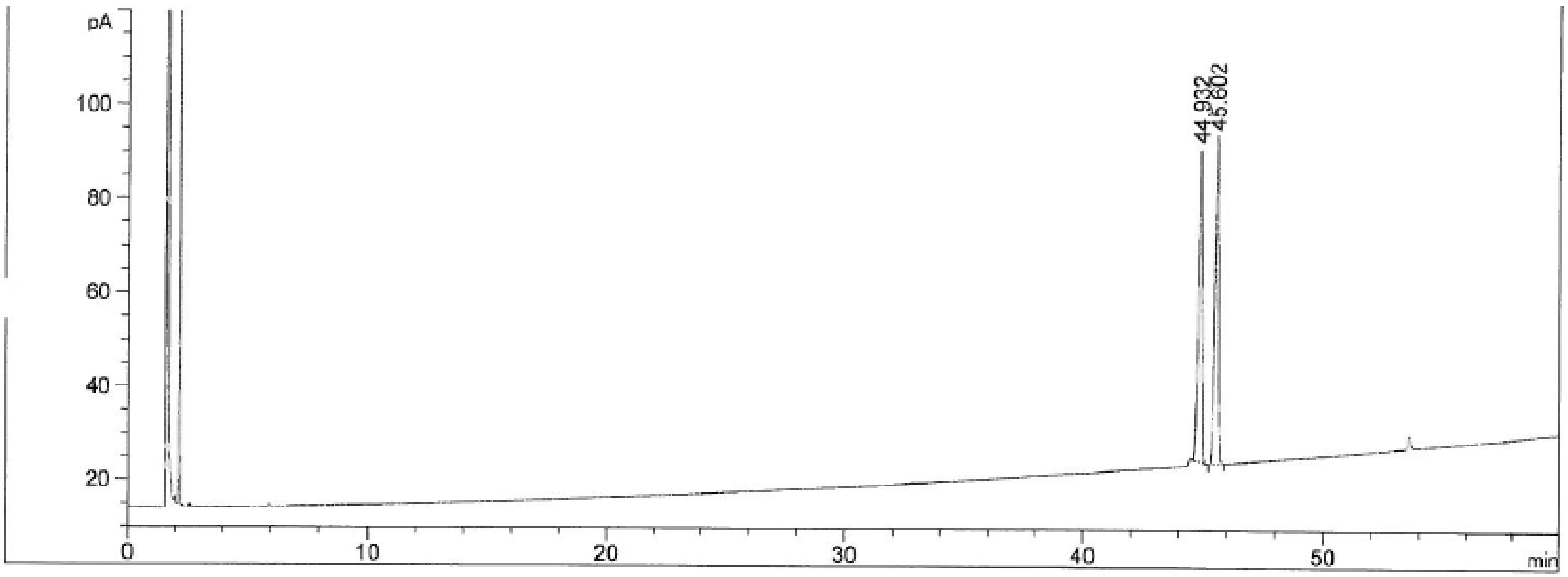

Peak #	RetTime [min]	Type	Width [min]	Area [pA*s]	Height [pA]	Area %
1	44.932	BB	0.1337	722.44250	66.53690	50.54210
2	45.602	BB	0.1354	706.94501	69.65852	49.45790

Figure S125. Chiral chromatogram of bromo-hydroxylactone **10a**

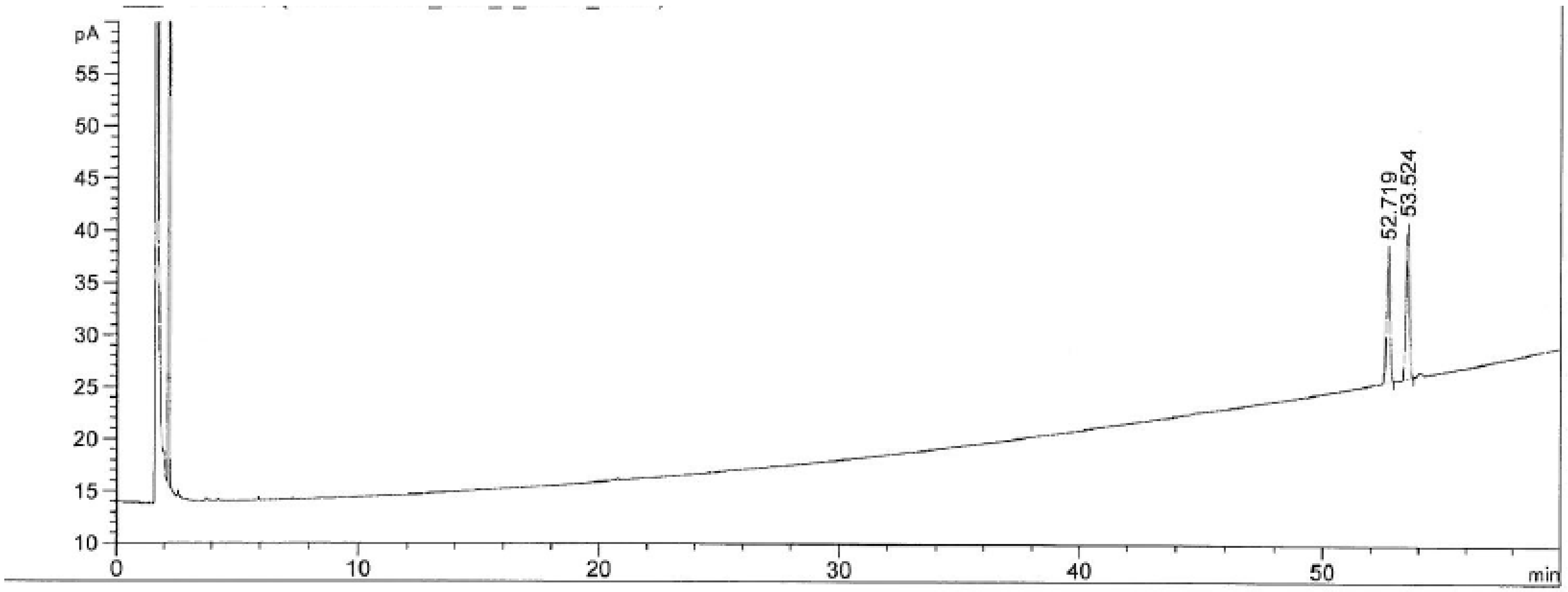

Peak #	RetTime [min]	Type	Width [min]	Area [pA*s]	Height [pA]	Area %
1	52.719	BB	0.1154	121.76060	12.98993	46.96020
2	53.524	BB	0.1174	137.52405	14.79928	53.03980

Figure S126. Chiral chromatogram of iodo-hydroxylactone **11a**
